# An In Silico Investigation of the Pathogenic G151R G Protein-Gated Inwardly Rectifying K^+^ Channel 4 Variant to Identify Small Molecule Modulators

**DOI:** 10.3390/biology13120992

**Published:** 2024-11-29

**Authors:** Eleni Pitsillou, Julia J. Liang, Noa Kino, Jessica L. Lockwood, Andrew Hung, Assam El-Osta, Asmaa S. AbuMaziad, Tom C. Karagiannis

**Affiliations:** 1 Epigenomic Medicine Laboratory at prospED Polytechnic, Melbourne, VIC 3053, Australia; 2 School of Science, STEM College, RMIT University, Melbourne, VIC 3001, Australia; 3 Epigenetics in Human Health and Disease Program, Baker Heart and Diabetes Institute, 75 Commercial Road, Melbourne, VIC 3004, Australia; 4 Department of Microbiology and Immunology, The University of Melbourne, Melbourne, VIC 3010, Australia; 5 Baker Department of Cardiometabolic Health, The University of Melbourne, Melbourne, VIC 3010, Australia; 6 Department of Diabetes, Central Clinical School, Monash University, Melbourne, VIC 3004, Australia; 7 Department of Medicine and Therapeutics, The Chinese University of Hong Kong, Sha Tin, Hong Kong SAR, China; 8 Hong Kong Institute of Diabetes and Obesity, Prince of Wales Hospital, The Chinese University of Hong Kong, 3/F Lui Che Woo Clinical Sciences Building, 30-32 Ngan Shing Street, Sha Tin, Hong Kong SAR, China; 9 Li Ka Shing Institute of Health Sciences, The Chinese University of Hong Kong, Sha Tin, Hong Kong SAR, China; 10 Biomedical Laboratory Science, Department of Technology, Faculty of Health, University College Copenhagen, 2200 Copenhagen, Denmark; 11 Department of Pediatrics, College of Medicine Tucson, The University of Arizona, Tucson, AZ 85724, USA; 12 Department of Clinical Pathology, The University of Melbourne, Melbourne, VIC 3010, Australia

**Keywords:** primary aldosteronism, G protein-gated ion channels, KCNJ5, GIRK4, G151R variant

## Abstract

Primary aldosteronism is characterised by the excessive production of aldosterone, which is a key regulator of salt metabolism, and is the most common cause of secondary hypertension. Studies have investigated the association between primary aldosteronism and genetic alterations, with pathogenic mutations being identified. This includes a glycine-to-arginine substitution at position 151 (G151R) of the G protein-activated inward rectifier potassium (K^+^) channel 4 (GIRK4), which is encoded by the *KCNJ5* gene. Mutations in GIRK4 have been found to reduce the selectivity for K^+^ ions, resulting in membrane depolarisation, the activation of voltage-gated Ca^2+^ channels, and an increase in aldosterone secretion. As a result, there is an interest in identifying and exploring the mechanisms of action of small molecule modulators of wildtype (WT) and mutant channels. In order to investigate the potential modulation of homotetrameric GIRK4^WT^ and GIRK4^G151R^ channels, homology models were generated. Molecular dynamics (MD) simulations were performed, followed by a cluster analysis to extract starting structures for molecular docking. The central cavity has been previously identified as a binding site for small molecules, including natural compounds. The OliveNet^TM^ database, which consists of over 600 compounds from *Olea europaea*, was subsequently screened against the central cavity. The binding affinities and interactions of the docked ligands against the GIRK4^WT^ and GIRK4^G151R^ channels were then examined. Based on the results, luteolin-7-O-rutinoside, pheophorbide a, and corosolic acid were identified as potential lead compounds. The modulatory activity of olive-derived compounds against the WT and mutated forms of the GIRK4 channel can be evaluated further in vitro.

## Simple Summary

Mutations in the *KCNJ5* gene, which encodes G protein-activated inward rectifier potassium (K^+^) channel 4 (GIRK4), are associated with the development of primary aldosteronism. A glycine-to-arginine amino acid substitution has been reported to occur at position 151 (G151R) of the GIRK4 channel (GIRK4^G151R^) and is among the most common somatic mutations found in aldosterone-producing adenomas. Previous studies have demonstrated the potential for small molecules to target and modulate the activity of the wildtype (WT) and mutated forms of GIRK channels. This includes the natural compound naringin, which is classified as a flavonoid. The protective properties of extra virgin olive oil, which is a key component of the Mediterranean diet and is rich in phenolic compounds, continue to be explored. We previously developed the OliveNet^TM^ library, a curated database of more than 600 compounds from *Olea europaea*. Using an in silico approach, we aimed to investigate the binding characteristics of olive-derived compounds from the OliveNet^TM^ library against the GIRK4^WT^ and GIRK4^G151R^ channels. Based on the binding affinities and interactions with the central cavity of the GIRK4^WT^ and GIRK4^G151R^ channels, lead compounds were identified. The potential modulatory activity of luteolin-7-O-rutinoside, pheophorbide a, and corosolic acid requires further investigation.

## 1. Introduction

Primary aldosteronism is a condition that is characterised by the autonomous overproduction of aldosterone, which is synthesised by the zona glomerulosa cells of the adrenal cortex [1]. Under physiological conditions, aldosterone is produced in response to sodium depletion, fluid loss, and hyperkalaemia [2,3]. Volume depletion results in the activation of the renin-angiotensin system and the production of angiotensin II (AII) [3]. Both AII signalling and hyperkalaemia cause membrane depolarisation and the opening of voltage-gated Ca^2+^ channels [3]. The increase in intracellular Ca^2+^ stimulates aldosterone production, leading to sodium and water reabsorption, increased K^+^ secretion, and blood pressure regulation [3]. In primary aldosteronism, the hypersecretion of aldosterone results in hypertension and variable hypokalaemia [3]. Primary aldosteronism is the most common cause of secondary hypertension [4,5].

Sporadic forms of primary aldosteronism are typically caused by aldosterone-producing adenomas or bilateral adrenal hyperplasia [5,6]. In addition to sporadic primary aldosteronism, several subtypes of familial hyperaldosteronism have been identified [5,6,7]. The genetic basis of primary aldosteronism continues to be investigated, as somatic and germline mutations have been detected in patients [1]. This includes genetic alterations in *KCNJ5*, which encodes G protein-activated inward rectifier potassium channel 4 (GIRK4) [1]. G protein-coupled inward rectifier K^+^ (GIRK) channels (Kir3.x) consist of four subunits (GIRK1-4) and are part of one of seven subfamilies of the inwardly-rectifying K^+^ (Kir) channel family [8,9]. GIRK channels play an important role in maintaining the resting membrane potential and regulating cellular excitability [8]. In the adrenal cortex, the GIRK4 channel exists as a homotetrameric structure [10]. Mutations in the GIRK4 channel primarily occur in or within close proximity to the selectivity filter, resulting in increased permeability to Na^+^ [1]. The influx of Na^+^ leads to the depolarisation of the cell membrane, the activation of voltage-gated Ca^2+^ channels, and an increase in aldosterone biosynthesis [1,11].

The glycine-to-arginine and leucine-to-arginine substitutions at positions 151 (G151R) and 168 (L168R), respectively, account for the majority of *KCNJ5* mutations in aldosterone-producing adenomas [3,12,13]. Aldosterone-producing adenomas with somatic *KCNJ5* mutations have been found to exhibit unique molecular characteristics including global DNA hypomethylation and transcriptomic profiles with gene expression changes [14]. Patients with the germline G151R mutation present with a severe form of primary aldosteronism with adrenocortical hyperplasia [3,15,16,17]. Moreover, patients with familial hyperaldosteronism type III with a pathogenic glycine-to-glutamic acid mutation at position 151 (G151E) display a relatively mild phenotype with no adrenal hyperplasia [18]. In comparison to G151R, the G151E mutation has been found to generate a larger Na^+^ conductance that is sufficient to induce cell death [7,18].

Treatment options for primary aldosteronism include surgical interventions, such as adrenalectomy and mineralocorticoid receptor antagonists [19]. Tauber et al. demonstrated that mutated GIRK4 channels are less sensitive to the honey bee toxin derivative tertiapin-Q and Ba^2+^, which are inhibitors of the wildtype (WT) channel [11]. There is evidence to suggest that tertiapin-Q targets the external vestibule of the ion conduction pore [20,21]. In a study by Cui et al., 3hi2one-G4 was identified as the first highly selective small molecule activator of homotetrameric GIRK4 channels [10]. The binding site was predicted to be formed by transmembrane domains 1 and 2 (TM1 and TM2) and the slide helix [10]. Furthermore, the inhibitory activity of various antidepressant drugs, volatile anaesthetics, and macrolides against WT and mutant forms of GIRK4 has been examined [11,22].

Interestingly, the biflavonoid naringin has been found to bind to the same site as tertiapin-Q and directly activates GIRK channels [10,20]. The results highlight the potential for polyphenols, including flavonoids, to directly target and modulate the activity of GIRK channels. The Mediterranean diet is characterised by the high consumption of extra virgin olive oil, which is a rich source of phenolic compounds [23]. The OliveNet^TM^ library was previously developed by our laboratory and is a comprehensive database of more than 600 compounds from *Olea europea* [24]. The anti-inflammatory and antioxidant properties of the phenolic compounds hydroxytyrosol, tyrosol, oleocanthal, and oleuropein have been well studied [24]. Nonetheless, the mechanisms of action of a vast majority of olive-derived compounds remain unexplored and approximately half are commercially available [25].

The binding sites and molecular mechanisms of action of small molecule modulators of WT and mutated homotetrameric GIRK4 channels require further elucidation. We previously evaluated the binding properties of olive-derived compounds against the pathogenic GIRK4^G151E^ channel [26]. Here, in silico methods were used to investigate the pathogenic GIRK4^G151R^ variant. Homology modelling and molecular dynamics (MD) simulations were performed to generate the starting structures of the GIRK4^WT^ and GIRK4^G151R^ channels for molecular docking. Due to the potential modulatory activity of natural compounds, such as naringin, the OliveNet^TM^ database was utilised in this study [24]. Based on the literature and ligand-binding site analysis, the central cavity region was identified as a target binding site for small molecules. Molecular docking was subsequently performed against the central cavity to identify potential modulators of the GIRK4^WT^ and GIRK4^G151R^ channels.

## 2. Materials and Methods

### 2.1. Protein Structure Preparation

The initial structure of the GIRK4 protein complex was prepared as previously described [26]. Briefly, a homology model of the homotetrameric GIRK4^WT^ channel was generated using SWISS-MODEL [27], with the human *KCNJ5* sequence (UniProt ID: P48544) as the query [28], and the cryo-EM structure of the mouse GIRK2 channel (PDB ID: 6XIT) as the template (3.3 Å resolution, 83.13% sequence identity) [29]. As described previously, PROCHECK was used to assess the stereochemical quality of the model, with 91.5% of residues in the most favoured region, 8.1% in the additionally allowed region, and 0.3% in the generously allowed region of the Ramachandran plot [26]. Coordinates of the partially resolved cofactor, phosphatidylinositol 4,5-bisphosphate (PIP_2_), were modified to generate full PIP_2_ molecules and were retained in the protein complex. To generate the GIRK4^G151R^ structure, mutations were manually introduced on all chains using the mutagenesis tool in PyMOL [30].

### 2.2. MD Simulations and Analysis

MD simulations were performed on the GIRK4^WT^ and GIRK4^G151R^ protein complexes using GROMACS version 2023 [31,32]. The CHARMM36 force field [33] was utilised, and cofactor topologies were generated using the Ligand Reader & Modeler in CHARMM-GUI [34,35]. Systems were solvated with TIP3P water [36] in a triclinic box under period boundary conditions, with a minimum distance of 1.0 nm between any protein atom to the box edge. Systems were neutralised and salted with 0.15 M NaCl and underwent energy minimisation with the steepest-descent gradient method until energies reached a maximum force of <1000 kJ/mol/nm. Equilibration was performed using the isothermal–isochoric (NVT) ensemble to 100 ps, maintaining a temperature of 310 K using a modified Berendsen thermostat [37], followed by isothermal–isobaric (NPT) equilibration at 1.0 bar with the Parrinello–Rahman barostat [38] for 1000 ps. Bond lengths were constrained with the LINCS algorithm [39], long-range electrostatic forces were calculated with the particle-mesh Ewald scheme [40] with a grid spacing of 0.16 nm, and short-range nonbonded interactions utilised cutoff ratios of 1.2 nm for Coulomb and van der Waals potentials. The final frame of the equilibrated system for GIRK4^WT^ and GIRK4^G151R^ structures were utilised for initial docking (Tables S1 and S2). Production runs were subsequently carried out for 200 ns with a time-step of 2 fs.

Trajectories were visualised and analysed using Visual Molecular Dynamics 1.9.3 [41]. The following analysis tools within GROMACS were utilised: gmx rms used for calculation of RMSD of the protein backbone; gmx gyrate to calculate the radius of gyration of the protein backbone; gmx sasa for the calculation of solvent accessible surface area of the protein surface; and gmx rmsf for the calculation of RMSF of the protein backbone. Principal components analysis (PCA) was performed to examine protein motions of the WT and G151R GIRK4 complexes. Equilibrated trajectories were used to calculate a covariance matrix using gmx covar. Using gmx anaeig, this was diagonalised to obtain a set of eigenvectors and corresponding eigenvalues. Free energy landscape (FEL) plots were calculated using gmx sham. Using gmx cluster, clusters of similar structures based on RMSD of the protein backbone was calculated on trajectories following equilibration, using the gromos clustering algorithm as described by Daura et al. [42]. With an RMSD cut-off of 0.17 nm to define two structures as neighbours, 18 clusters were identified for GIRK4^WT^ and 9 clusters for GIRK4^G151R^. The middle structure from the first three clusters for each system were written and used as structures for the screening of compounds with molecular docking. The structures of the GIRK4^WT^ and GIRK4^G151R^ channels were prepared as proteins using AutoDockTools-.1.5.7 [43].

### 2.3. Ligand Preparation

The chemical structures of verapamil, diltiazem, and roxithromycin were obtained from the National Center for Biotechnology Information PubChem database [44]. The two-dimensional structure of roxithromycin was converted to the three-dimensional format using Chem3D (v23.1.1, PerkinElmer Informatics, Shelton, CT, USA). The structures of verapamil, diltiazem, and roxithromycin were imported into PyRx, energy-minimised using the universal force field through Open Babel (v.2.2.3), and were prepared as ligands using AutoDockTools.1.5.7 [43,45,46]. The energy minimised chemical structures of the olive-derived compounds from the OliveNet^TM^ library were also utilised in this study [24].

### 2.4. Ligand-Binding Site Analysis and Molecular Docking

The representative protein structures for clusters 1, 2, and 3 of the GIRK4^WT^ and GIRK4^G151R^ channels were uploaded to the PrankWeb server [47]. Using conservation analysis, the surface of each protein structure was evaluated to identify potential ligand-binding sites [47]. Based on the ligand-binding site analysis and the literature, the receptor grids for molecular docking were centred around residues Y97, E147, T148, T149, A172, G175, and N179 of each chain [48]. The receptor grids were 20 × 20 × 20 Å in size. Molecular docking was performed at an exhaustiveness of 128 using AutoDock Vina [49]. The binding affinities (kcal/mol) were recorded. The receptor–ligand interactions were analysed using Maestro 13.2 and Visual Molecular Dynamics 1.9.3 [41,50]. GraphPad Prism 10.2.0 (Boston, MA, USA) was used to generate boxplots of the binding affinities for the OliveNet^TM^ classes (Tukey method).

## 3. Results and Discussion

GIRK subunits are composed of two transmembrane domains (TM1 and TM2) which are linked by a pore-forming region (H5) and cytoplasmic amino (N)- and carboxy (C)-terminal domains [8,9]. Functional GIRK channels consist of four subunits that form heterotetrameric or homotetrameric complexes [8,9]. The activation of G protein-coupled receptors (GPCRs) by hormones or neurotransmitter ligands results in the release of Gα and Gβγ effector molecules [9]. GIRK channels are activated by the binding of Gβγ subunits of pertussis toxin-sensitive G proteins to the cytoplasmic region, which allows K^+^ to flow into the cell and leads to membrane hyperpolarisation [9].

### 3.1. MD Simulation and Cluster Analysis of GIRK4 Protein Channels

**Figure 1:**
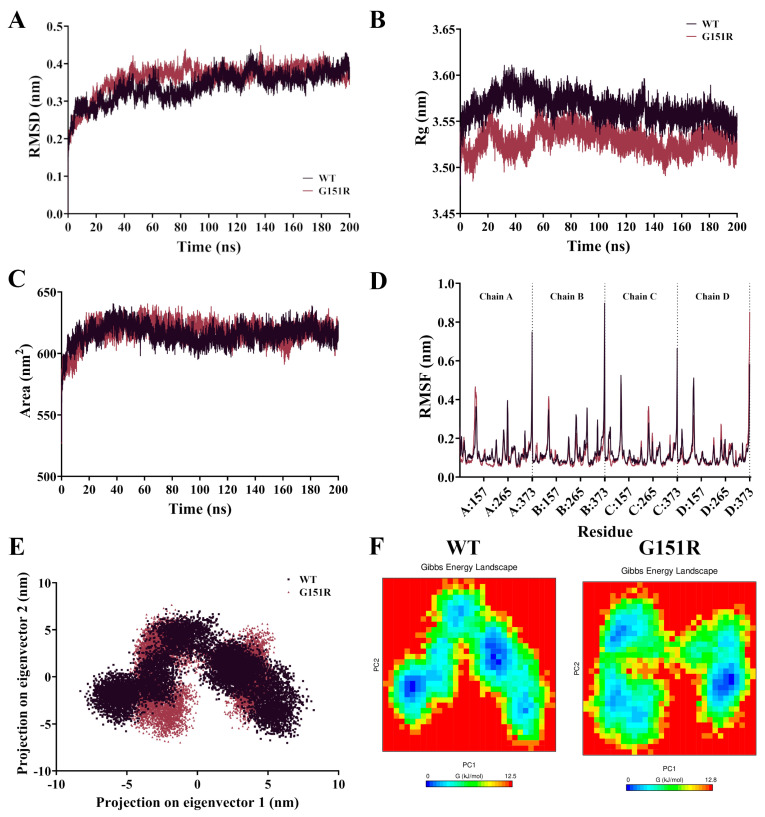
A molecular dynamics (MD) simulation of the GIRK4^WT^ and GIRK4^G151R^ protein complexes. (**A**) Root mean square deviation (RMSD) of protein backbone for WT (dark purple) and G151R (dark pink) variant GIRK4 complexes over 200 ns. **(B)** The radius of gyration (Rg) of protein backbone. (**C**) The solvent accessible surface area (SASA) of protein surface. (**D**) Root mean square fluctuation (RMSF) of protein backbone of WT and G151R GIRK4 following system equilibration. (**E**) Principal components analysis (PCA) of WT and G151R GIRK4 complex protein backbone following equilibration, showing 2D projection of the on the first two eigenvectors. (**F**) Free energy landscape plots (FEL) calculated from the first two principal components (PC1 and PC2).

Classical MD simulations were performed for the GIRK4^WT^ and GIRK4^G151R^ channels for 200 ns (Figures S1 and S2). Root mean square deviation (RMSD) of the protein backbone showed that systems equilibrated after 100 ns; thus, subsequent analysis was performed following this timepoint. Following equilibration, the GIRK4^WT^ and GIRK4^G151R^ systems displayed similar RMSD, with average values of 0.37 nm for WT and 0.38 nm for G151R (Figure 1A). Similarly, an analysis of the radius of gyration (Rg) of the protein backbone (Figure 1B) and solvent accessible surface area (SASA) of the protein (Figure 1C) showed that the protein complexes equilibrated after 100 ns with little difference between systems. Average values for Rg were 3.56 nm for WT and 3.53 nm for G151R, while the average values for SASA were 615.8 nm^2^ for WT and 616.9 nm^2^ for G151R. Principal components analysis (PCA) (Figure 1E) of the protein backbone showed that both WT and G151R occupied a similar conformation subspace, suggesting that structural rearrangements within the protein complexes are subtle between the two variants. Free energy landscape analysis (FEL) plots (Figure 1F) showed deep energy wells, suggesting the presence of energetically favourable conformations. The WT complex appeared to favour multiple conformations, compared to the G151R variant complex which had more of a tendency towards a single stable conformation.

Root mean square fluctuation (RMSF) of the protein backbone was calculated and is depicted in Figure 1D. The largest fluctuations are observed to occur at the N-terminal region of each chain. Fluctuations occurred in the distal loops, with an RMSF of ~0.50 nm for WT and ~0.40 nm for G151R at residues 120–124, and ~0.30 nm for both systems at residues 245–247. The remaining regions of the protein complexes remained relatively stable, with residues of the selectivity motif at positions 149–153 displaying minimal fluctuations indicating stability of this region of the protein (Figure S3).

**Figure 2:**
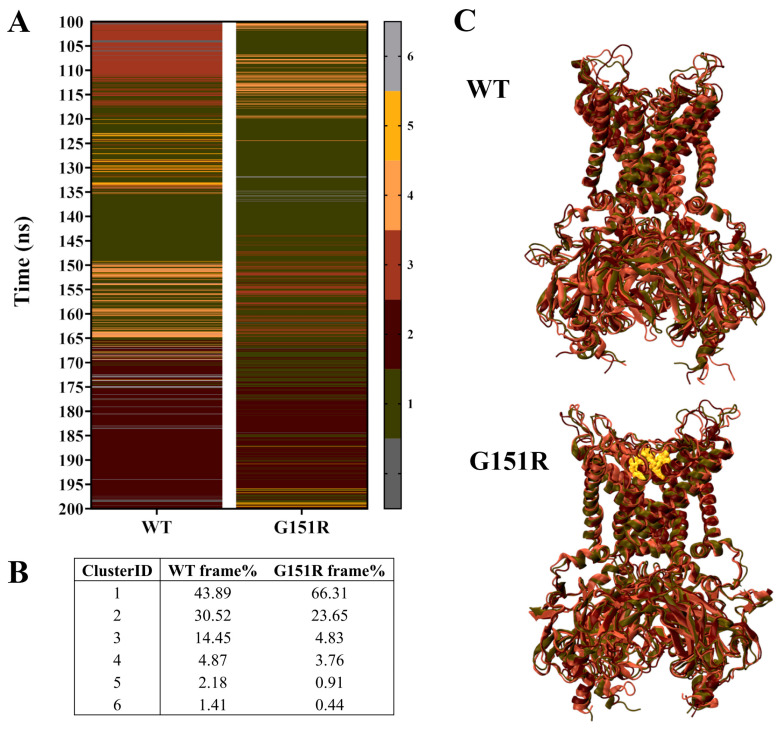
A cluster analysis of the GIRK4^WT^ and GIRK4^G151R^ protein complexes. (**A**) A cluster analysis of 10,000 frames from the equilibrated trajectories of the GIRK4 WT and G151R complexes. The top six clusters are depicted in the heatmap, with the other clusters shown in dark grey. (**B**) The percentage of frames from each trajectory allocated to the top six clusters. (**C**) A visualisation of the top three clusters overlaid for the WT and G151R GIRK4 complexes, with the G151R mutation highlighted in orange in a van der Waals representation.

A cluster analysis of the equilibrated WT and G151R trajectories utilised an RMSD cut-off of 0.17 nm to identify neighbouring structures from 10,000 frames of each complex. Given this stringent cutoff, the resulting clusters were highly similar. Nonetheless, the middle structure from the top three clusters, which collectively encompass approximately 90% of the observed conformations (Figure 2A–C), were extracted for docking. The ensemble-based approach can be used to provide further insight into ligand binding across multiple conformations of a protein [51].

Figure 2B shows the percentage of frames from each trajectory allocated to the top clusters. From the cluster analysis, the WT complex displays a more even spread of frames between the top three clusters (43.89%, 30.52%, and 14.45% allocated to clusters 1, 2, and 3, respectively), while the G151R complex has the majority of frames belonging to cluster 1 (66.31%), a smaller number to cluster 2 (23.65%), and a minimal number to cluster 3 (4.83%). This is in line with the FEL plots shown in Figure 1F, which indicated a preference for G151R to favour a single energetically favourable conformation. Structures extracted from the top three clusters are visualised in Figure 2C.

### 3.2. Identification of the Central Cavity as a Ligand-Binding Site in GIRK4^WT^ and GIRK4^G151R^ Channels

The central cavity of inwardly rectifying K^+^ channels has been investigated as a target binding site for small molecules such as proflavine, chloroquine, quinacrine, and BP-G1 [52,53,54]. In a study by Cui et al., the benzopyran derivative BP-G1 was found to selectively inhibit heteromeric GIRK1/GIRK4 channels over homomeric GIRK channels [48]. To investigate the mechanisms of action of BP-G1, a homology model of a GIRK4/GIRK4(S143F) heteromer was generated [48]. The S143F mutation was introduced to induce structural and functional changes that resembled the GIRK1 central pore [48]. The molecular modelling results demonstrated that E147 and N179 in the GIRK4(S143F) subunit contributed to the binding of BP-G1 [48]. Although BP-G1 was not an effective inhibitor of the homomeric GIRK4^WT^ channel, the binding site identified in the GIRK4/GIRK4(S143F) heteromer remains a relevant target for investigating the interactions and potential modulatory activity of small molecules against the GIRK4 channel [48].

The PrankWeb server was used to evaluate potential ligand-binding sites in the representative structures of each cluster for the GIRK4^WT^ and GIRK4^G151R^ homotetrameric channels [47,55]. In the study by Cui et al., residues Y97, E147, T148, T149, A172, G175, and N179 formed part of the central cavity region [48]. The ligand-binding site analysis revealed that the second putative binding site (pocket 2) for each cluster of the GIRK4^WT^ and GIRK4^G151R^ channels included residues Y97, E147, T148, T149, A172, G175, and N179 (Table S3). The in silico results indicate that these residues also form part of a binding site in the GIRK4^WT^ and GIRK4^G151R^ homotetrameric channels and were subsequently used for molecular docking.

### 3.3. Binding Characteristics of Verapamil, Diltiazem, and Roxithromycin Against GIRK4^WT^ and GIRK4^G151R^ Channels

The aldosterone-to-renin-ratio (ARR) is widely used as a screening test for primary aldosteronism [56]. The Endocrine Society guidelines suggest that antihypertensive medications, such as verapamil, with minimal effects on plasma aldosterone levels may be provided to maintain hypertension control during the testing process [57]. Verapamil is an antiarrhythmic drug that inhibits L-type Ca^2+^ channels [11]. In a study by Tauber et al., verapamil normalised Ca^2+^ activity in transfected NCI-H295R cells with mutant GIRK4 channels but had no effect in cells with GIRK4^WT^ channels [11]. Most notably, the GIRK4^G151R^ mutant was inhibited by verapamil, as 69 ± 12% of the Na^+^ inward current remained at 1 μM and 43 ± 9% at 10 μM [11]. Tauber et al. reported that verapamil may have a dual mechanism of action that involves directly blocking mutated GIRK4 channels and inhibiting depolarisation-activated voltage-gated Ca^2+^ channels [11]. Therefore, the effect of verapamil on the results of the screening test for patients with *KCNJ5* mutations and its use as a potential inhibitor of mutated GIRK4 channels require further investigation.

**Figure 3:**
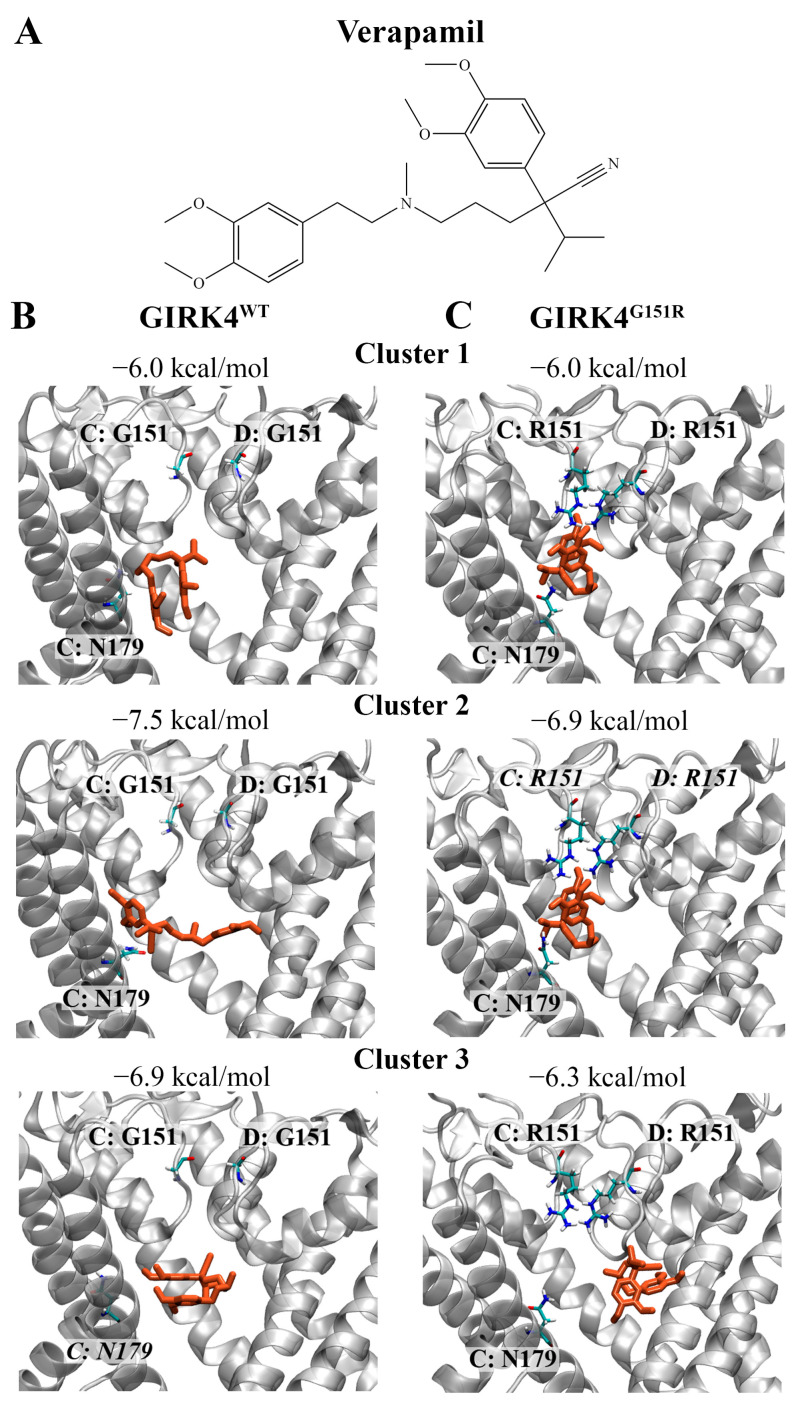
The binding characteristics of the positive control inhibitor verapamil against the central cavity of the GIRK4 channels. (**A**) The chemical structure of verapamil is shown. (**B**) The predicted non-covalent interactions of verapamil with the central cavity of the (**B**) GIRK4^WT^ and (**C**) GIRK4^G151R^ channels are provided. Verapamil is coloured red. Key residues associated with each chain of the homotetrameric structures are labelled. Residues that were predicted to form hydrogen bonds with verapamil are italicised.

Molecular docking was used to evaluate the binding characteristics of verapamil against the central cavity of the GIRK4^WT^ and GIRK4^G151R^ channels. As seen in Figure 3, verapamil was predicted to bind to the GIRK4^WT^ channel with an affinity of −6.0 kcal/mol for cluster 1, −7.5 kcal/mol for cluster 2, and −6.9 kcal/mol for cluster 3. A hydrogen bond was observed between verapamil and the polar residue N179 of cluster 3. Verapamil was predicted to bind with an affinity of −6.0, −6.9, and −6.3 kcal/mol to the cluster 1, 2, and 3 structures of the GIRK4^G151R^ channel, respectively. Hydrogen bonds were detected between verapamil and the mutated R151 residue of cluster 2. Verapamil was previously found to bind to the central cavity of the GIRK4^WT^ and GIRK4^G151E^ channels with an affinity of −7.2 and −6.4 kcal/mol, respectively [26].

Diltiazem, which is classified as a selective calcium channel blocker, is also used as an antihypertensive drug [58]. In the study by Tauber et al., diltiazem exhibited inhibitory activity against the GIRK4^L168R^ mutant channel (IC_50_ = 11 μM) [11]. Molecular docking was therefore performed to evaluate the binding characteristics of diltiazem against the GIRK4^WT^ and GIRK4^G151R^ channels. Diltiazem was predicted to bind with an affinity of −7.2 kcal/mol, −6.8 kcal/mol, and −7.5 kcal/mol to the representative structures for clusters 1, 2, and 3 of the GIRK4^WT^ channel, respectively. Similarly for the GIRK4^G151R^ channel, diltiazem was predicted to bind to the representative cluster 1, 2, and 3 structures with an affinity of −7.3 kcal/mol, −6.8 kcal/mol, and −7.0 kcal/mol, respectively. For both the GIRK4^WT^ and GIRK4^G151R^ channels, diltiazem formed hydrogen bonds with S176 of cluster 1 and N179 of cluster 3 (Figure S4).

Roxithromycin has also been previously identified as a potent inhibitor of the GIRK4^G151R^ channel [22]. The macrolide antibiotic was predicted to bind to the cluster 1, 2, and 3 structures of the GIRK4^WT^ channel with an affinity of −8.1, −8.2, and −8.1 kcal/mol, respectively (Figure S5). Similar binding affinities were obtained for the GIRK4^G151R^ channel: −8.1 kcal/mol for cluster 1, −8.3 kcal/mol for cluster 2, and −7.9 kcal/mol for cluster 3. Roxithromycin was predicted to form hydrogen bonds with N179 of the cluster 1 structure, T148 of the cluster 2 structure, as well as T149 and S176 of the cluster 3 structure for the GIRK4^WT^ channel. Like verapamil, roxithromycin was predicted to form a hydrogen bond with the mutated R151 residue of cluster 2 for the GIRK4^G151R^ channel. Hydrogen bonds were also observed between roxithromycin and residues T149, S176, and N179 of the cluster 3 structure. Based on the molecular docking results from our previous study, roxithromycin was predicted to form hydrogen bonds with N179 of the GIRK4^WT^ and GIRK4^G151E^ channels [26]. The binding affinities for the GIRK4^WT^ and GIRK4^G151E^ channels were −8.4 and −7.9 kcal/mol, respectively [26].

### 3.4. Molecular Docking of the OliveNet^TM^ Library

**Figure 4:**
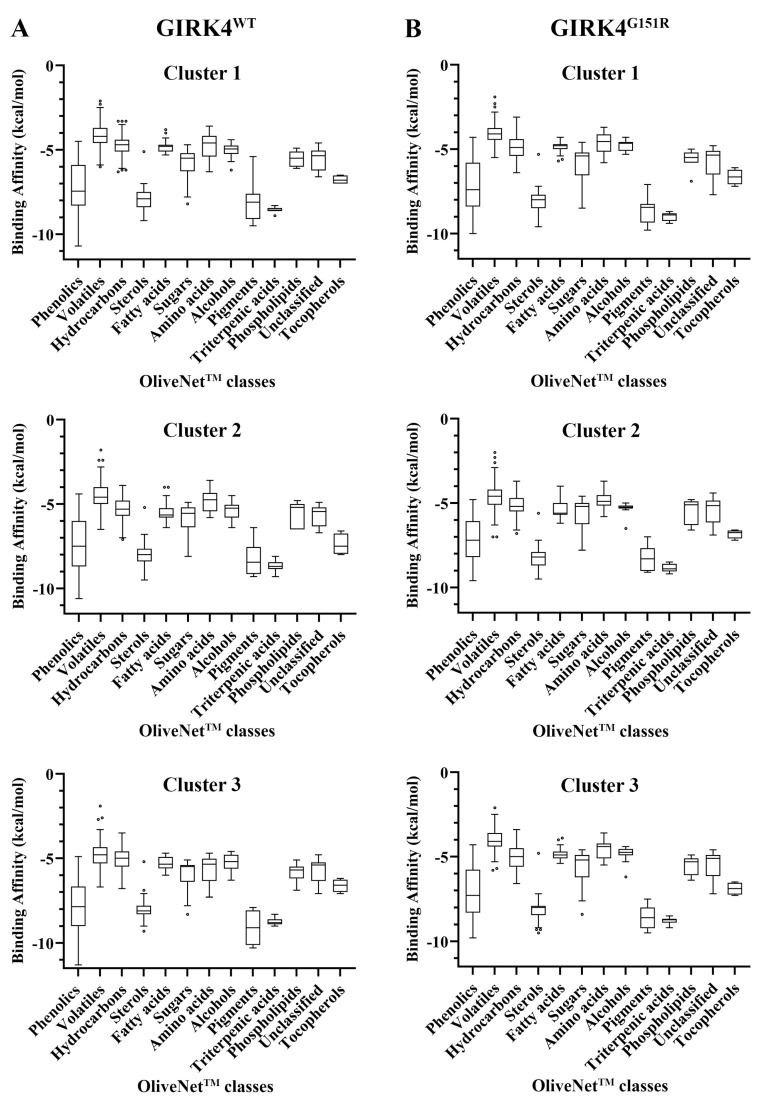
The molecular docking results of the OliveNet^TM^ classes against the GIRK4 channels. The binding affinities (kcal/mol) of the compounds within each class are provided for the representative structures of clusters 1, 2, and 3 for the (**A**) GIRK4^WT^ and (**B**) GIRK4^G151R^ channels. The boxplots are shown for the phenolic compounds, volatiles, hydrocarbons, sterols, fatty acids, sugars, amino acids, aliphatic and aromatic alcohols, pigments, triterpenic acids, phospholipids, unclassified compounds, and tocopherols. The boxplots were generated using the Tukey method (GraphPad Prism 10.2.0).

The OliveNet^TM^ library is composed of 13 classes and 47 subclasses [24]. The olive-derived compounds were screened against the central cavity of the GIRK4^WT^ and GIRK4^G151R^ channels (Tables S4–S9). As seen in Figure 4, the phenolic compounds, sterols, pigments, and triterpenic acids were the strongest binding classes for the GIRK4^WT^ and GIRK4^G151R^ channels. The phenolic compounds constitute the largest class (n = 222) and are divided into 13 subclasses [24]. The flavonoids and glucosides were predicted to be the strongest binding subclasses for the GIRK4^WT^ and GIRK4^G151R^ channels.

The 30 compounds with the strongest binding affinities for the GIRK4^WT^ and GIRK4^G151R^ channels were evaluated (Tables S10–S15). In general, the olive-derived compounds were binding to the central cavity with a greater affinity than the positive control inhibitors verapamil and roxithromycin. More than half of the top 30 compounds for the GIRK4^WT^ channel were classified as phenolics, particularly flavonoids and glucosides. The other subclasses included secoiridoids (phenolics), hydroxycinnamic acids (phenolics), triterpene alcohols (sterols), and chlorophylls (pigments). When examining the predicted non-covalent bonds that were formed between the ligands and the central cavity of the GIRK4^WT^ channel, E147 was identified as the most common interacting residue for all three clusters (Table 1). The negatively charged E147 residue mainly formed hydrogen bonds with the olive-derived compounds.

A similar trend was observed for the GIRK4^G151R^ channel, as the top 30 compounds were predominantly classified as flavonoids, glucosides, and secoiridoids. Compounds belonging to the hydroxycinnamic acid, chlorophyll (pigments), and triterpene alcohol (sterols) subclasses, as well as the triterpenic acid class also appeared in the top 30 for the GIRK4^G151R^ channel. Interestingly, several triterpenic acids were amongst the top 30 compounds for GIRK4^G151R^ but not GIRK4^WT^. They included corosolic acid, urs-2β-3β-dihydroxy-12-en-28-oic acid, maslinic acid, and pomolic acid. In our previous study, the triterpenic acids were predicted to bind to the G-loop region of the mutated GIRK4^G151E^ channel with a stronger affinity compared to the GIRK4^WT^ channel [26].

In comparison to GIRK4^WT^, the key residues of the GIRK4^G151R^ channel that were predicted to form non-covalent interactions with the ligands included E147 (negatively charged), R151 (positively charged), and N179 (polar) (Table 2). The olive-derived compounds predominantly formed hydrogen bonds with E147, R151, and N179. Salt bridges were also detected with R151. Interactions with residue 151 were only detected for the GIRK4^G151R^ channel, which suggests that the mutated residue may directly contribute to ligand binding.

The highly conserved selectivity filter of Kir channels, which is composed of the signature sequence T-X-G-Y(F)-G, creates a pathway for K^+^ ions across the membrane [9,59]. The sequence corresponds to residues T149-G153 in the GIRK4 channel, with the pathogenic R151 mutation affecting the first glycine [60]. The interaction of Kir channels with Mg^2+^ and polyamines governs the inward rectification of K^+^ influx [9]. Mg^2+^ and polyamines block K^+^ permeation by binding to residues in the transmembrane and cytoplasmic regions, thereby regulating the outward current [9]. A critical determinant of the degree of rectification is the D/N site residue in the TM2 helix [61]. Strong rectifiers contain a negatively charged residue, such as D173 in GIRK1, while weak rectifiers possess a neutral amino acid at this position [61]. In the GIRK4 channel, the site corresponds to residue N179 [9]. Furthermore, Jin et al. reported that the highly conserved G175 residue in the middle of the TM2 helix plays a critical role in the activation of GIRK channels by Gβγ [9,62].

**Figure 5:**
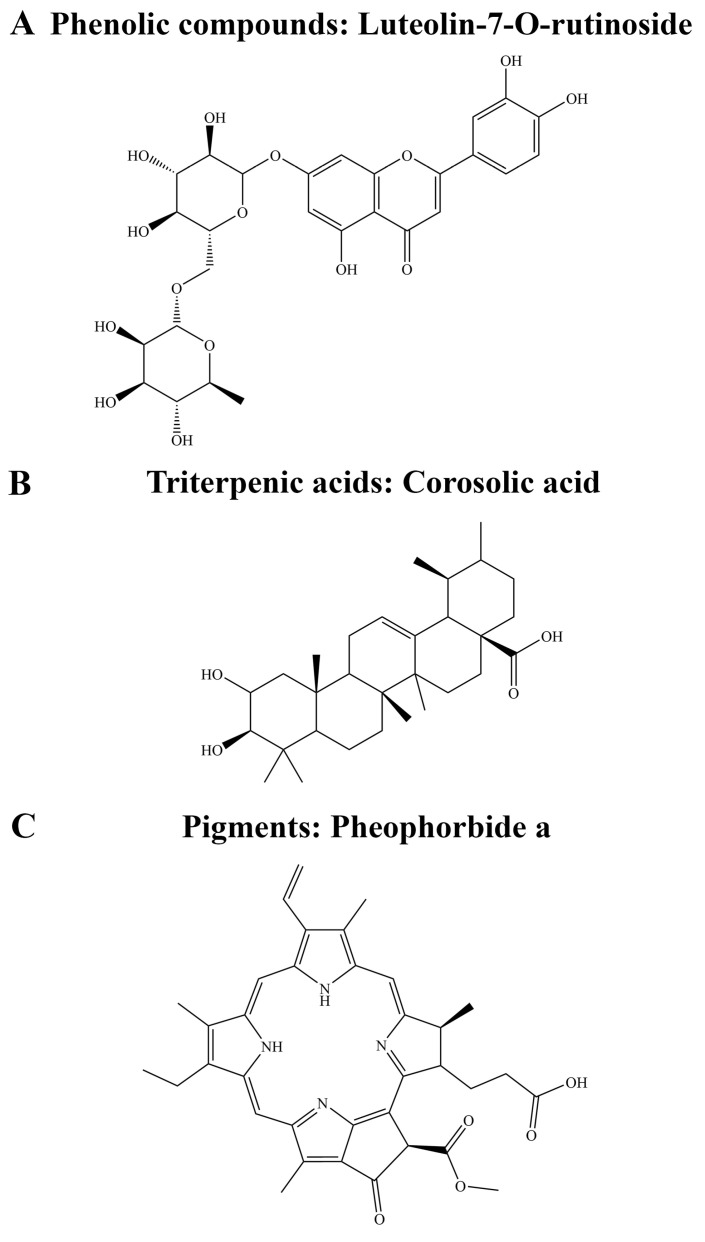
The chemical structures of potential lead compounds that were identified from molecular docking against the GIRK4^WT^ and GIRK4^G151R^ channels. The structures of (**A**) luteolin-7-O-rutinoside, (**B**) corosolic acid, and (**C**) pheophorbide a are provided.

Out of the top 30 compounds for the GIRK4^G151R^ channel, luteolin-7-O-rutinoside (phenolic compound) and pheophorbide a (pigment) were predicted to form non-covalent interactions with the mutated R151 residue in the representative structures for clusters 1, 2, and 3. Pheophorbide a appeared exclusively in the top 30 compounds for the GIRK4^G151R^ channel, while luteolin-7-O-rutinoside was in the top 30 for the GIRK4^WT^ and GIRK4^G151R^ channels. Similar results were obtained for luteolin-7-O-rutinoside from a preliminary screen of the OliveNet^TM^ library against the structures of the GIRK4^WT^ and GIRK4^G151R^ channels that were obtained from the final frame of the equilibrated system (Tables S1 and S2). Moreover, corosolic acid (triterpenic acid) was found to occur in the top 30 for clusters 1 and 2 of the GIRK4^G151R^ channel. Luteolin-7-O-rutinoside, pheophorbide, and corosolic acid were subsequently selected for further analysis (Figure 5).

### 3.5. Phenolic Compound (Flavonoid): Luteolin-7-O-Rutinoside

**Figure 6:**
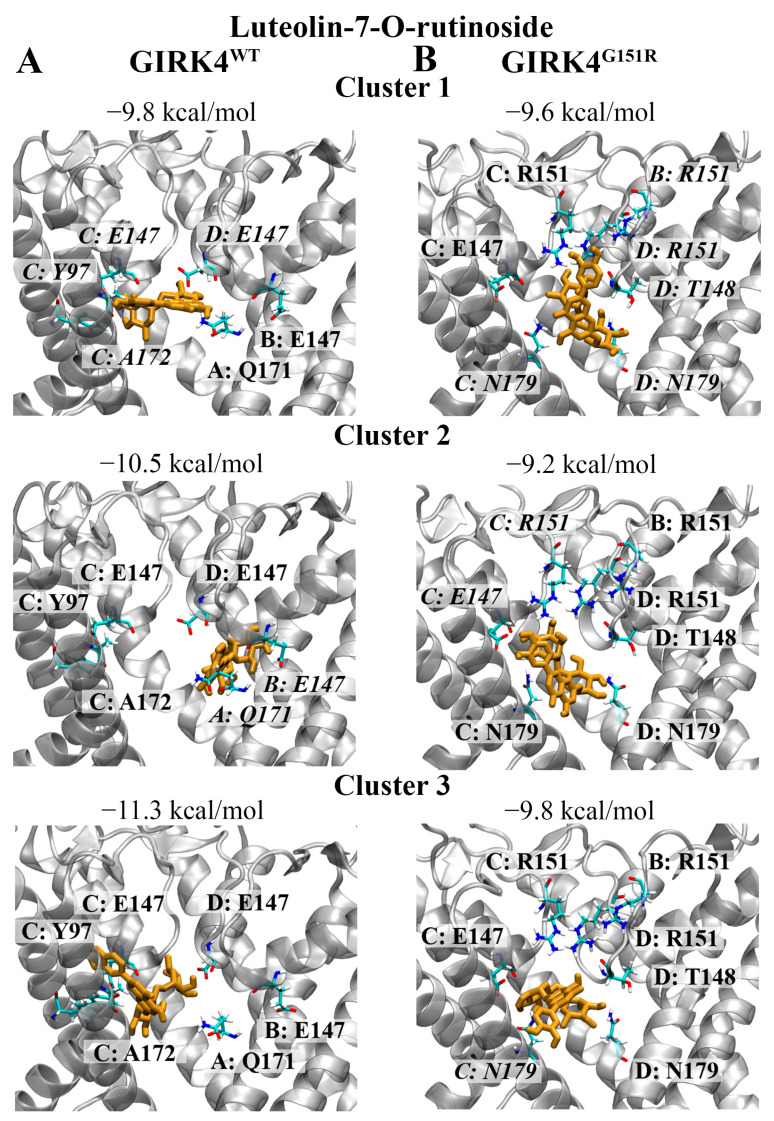
The binding characteristics of luteolin-7-O-rutinoside against the central cavity of the GIRK4 channels. The predicted non-covalent interactions of luteolin-7-O-rutinoside with the central cavity of the (**A**) GIRK4^WT^ and (**B**) GIRK4^G151R^ channels are provided. Luteolin-7-O-rutinoside is coloured orange. Key residues associated with each chain of the homotetrameric structures are labelled. Residues that were predicted to form hydrogen bonds with luteolin-7-O-rutinoside are italicised.

Luteolin-7-O-rutinoside was predicted to bind to the GIRK4^WT^ channel with an affinity of −9.8 kcal/mol for cluster 1, −10.5 kcal/mol for cluster 2, and −11.3 kcal/mol for cluster 3 (Figure 6A). Hydrogen bonds were predicted to form between the hydroxyl groups of luteolin-7-O-rutinoside and the central cavity residues including Y97, E147, and A172 of the representative structure for cluster 1. Hydrogen bonds were also predicted to occur between the hydroxyl groups of luteolin-7-O-rutinoside and residues E147 and Q171 of the representative structure for cluster 2.

For the GIRK4^G151R^ channel, luteolin-7-O-rutinoside was predicted to bind with an affinity of −9.6 kcal/mol for cluster 1, −9.2 kcal/mol for cluster 2, and −9.8 kcal/mol for cluster 3 (Figure 6B). Luteolin-7-O-rutinoside formed hydrogen bonds with T148, R151, and N179 of the representative structure for cluster 1. Residues E147 and R151 of the representative protein structure for cluster 2 were predicted to form hydrogen bonds with luteolin-7-O-rutinoside. A π-π cation and hydrogen bond were observed with residues R151 and N179 of the representative structure for cluster 3, respectively. Similar to the GIRK4^WT^ channel, the hydroxyl groups of luteolin-7-O-rutinoside were the main functional group involved in the formation of hydrogen bonds with the GIRK4^G151R^ channel.

### 3.6. Pigment (Chlorophyll): Pheophorbide a

**Figure 7:**
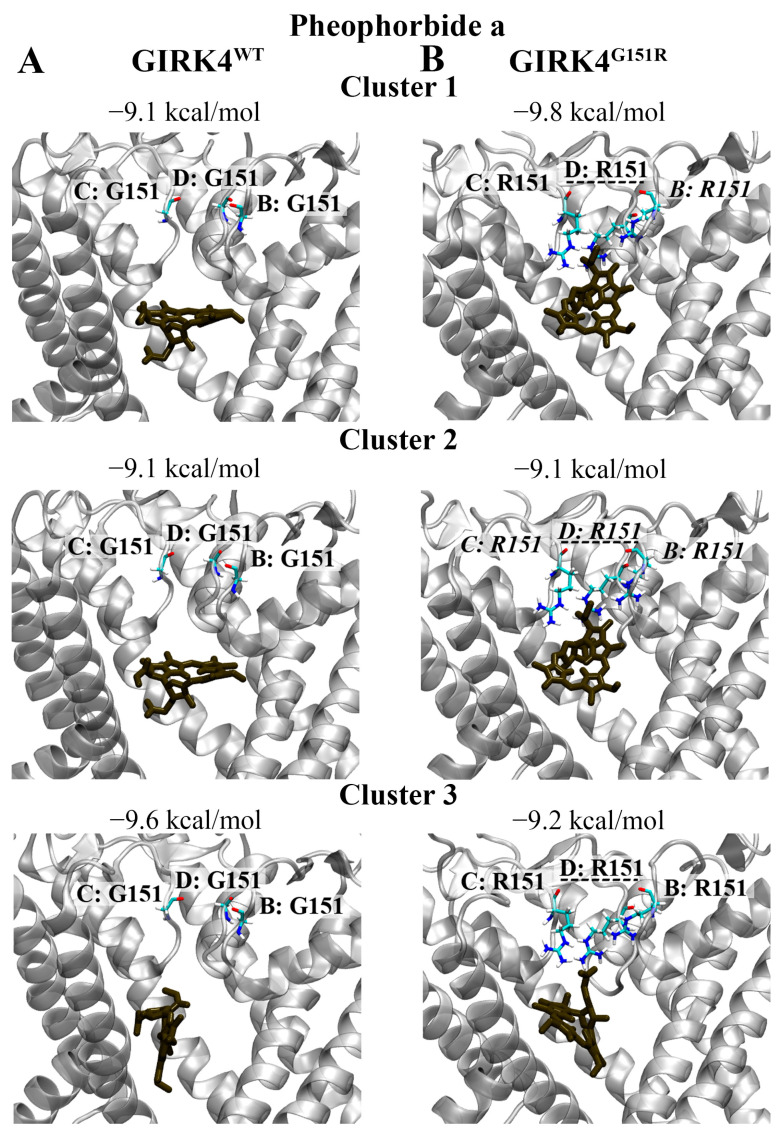
The binding characteristics of pheophorbide a against the central cavity of the GIRK4 channels. The predicted non-covalent interactions of pheophorbide a with the central cavity of the (**A**) GIRK4^WT^ and (**B**) GIRK4^G151R^ channels are provided. Pheophorbide a is coloured dark brown. Key residues associated with each chain of the homotetrameric structures are labelled. Residues that were predicted to form hydrogen bonds and salt bridges with pheophorbide a are italicised and underlined, respectively.

Pheophorbide a formed part of the top 30 ligands for clusters 1, 2, and 3 of the GIRK4^G151R^ channel. As seen in Figure 7B, the binding affinity was predicted to be −9.8 kcal/mol for cluster 1, −9.1 kcal/mol for cluster 2, and −9.2 kcal/mol for cluster 3. Hydrogen bonds and salt bridges were predicted to form between pheophorbide a and the mutated R151 residue of the representative structures for clusters 1 and 2. Pheophorbide a was also predicted to form salt bridges with R151 of the representative structure for cluster 3. The hydrogen bonds and salt bridges were primarily occurring with the oxygen atoms of pheophorbide a.

The binding affinities and interactions of pheophorbide a with the GIRK4^WT^ channel were also examined (Figure 7A). Pheophorbide a was predicted to bind to clusters 1 and 2 with an affinity of −9.1 kcal/mol, and cluster 3 with an affinity of −9.6 kcal/mol. In comparison to the GIRK4^G151R^ channel, no intermolecular bonds were predicted to occur with the central cavity residues.

### 3.7. Triterpenic Acid: Corosolic Acid

**Figure 8:**
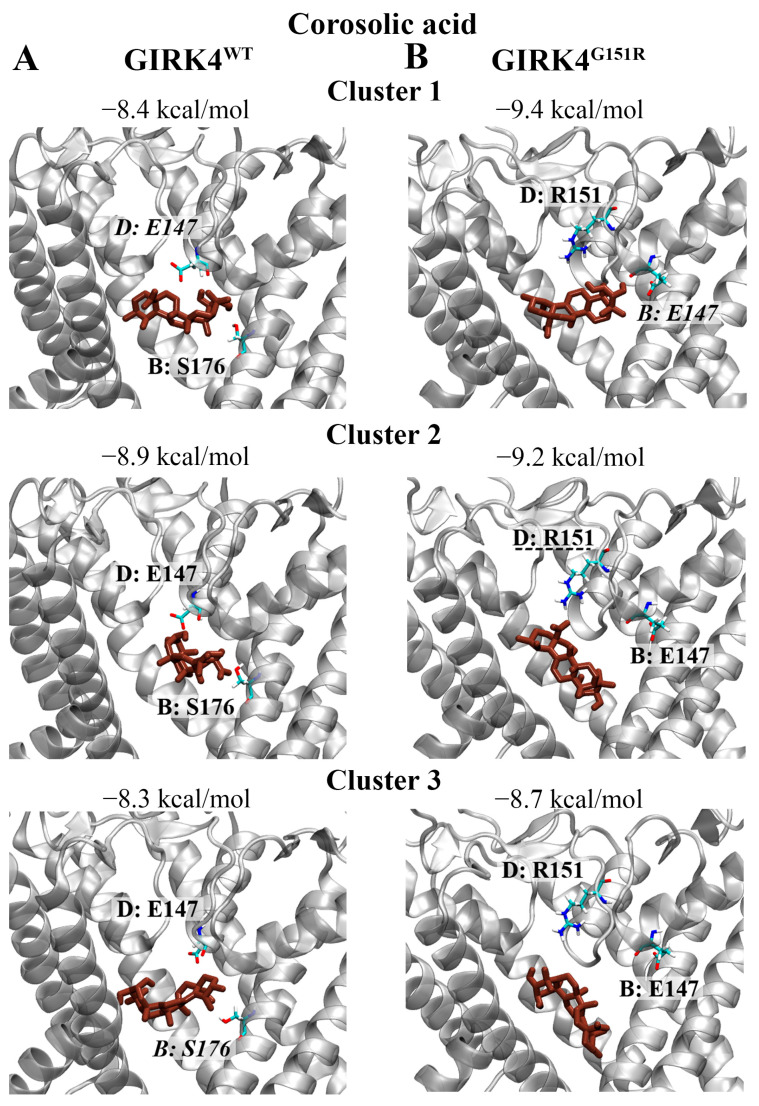
The binding characteristics of corosolic acid against the central cavity of the GIRK4 channels. The predicted non-covalent interactions of corosolic acid with the central cavity of the (**A**) GIRK4^WT^ and (**B**) GIRK4^G151R^ channels are provided. Corosolic acid is coloured purple. Key residues associated with each chain of the homotetrameric structures are labelled. Residues that were predicted to form hydrogen bonds and salt bridges with corosolic acid are italicised and underlined, respectively.

Corosolic acid was in the top 30 ligands for clusters 1 and 2 of the GIRK4^G151R^ channel (Figure 8B). The binding affinity for cluster 1 was predicted to be −9.4 kcal/mol, −9.2 kcal/mol for cluster 2, and −8.7 kcal/mol for cluster 3. Corosolic acid was predicted to form a hydrogen bond (hydroxyl group) with E147 and a salt bridge with R151 (oxygen atom) for the representative structures of cluster 1 and cluster 2, respectively. When analysing the results for the GIRK4^WT^ channel, corosolic acid was predicted to bind with an affinity of −8.4 kcal/mol for cluster 1, −8.9 kcal/mol for cluster 2, and −8.3 kcal/mol for cluster 3 (Figure 8A). Corosolic acid was predicted to form hydrogen bonds with E147 and S176 for the representative structures of cluster 1 and 3, respectively.

Luteolin-7-O-rutinoside, pheophorbide a, and corosolic acid have been found to exhibit antioxidant and anti-inflammatory properties [63,64,65,66]. The anticancer activity of pheophorbide a has also been explored, as the chlorophyll-based photosensitiser induces anti-proliferative effects against various cancer cell lines [65,67]. Several studies have demonstrated the antihypertensive properties of corosolic acid; however, the underlying mechanisms require further elucidation [66,68]. Yamamura et al. recently showed that the anti-inflammatory and anti-proliferative effects on macrophages contributed to the amelioration of pulmonary vascular remodelling in pulmonary arterial hypertension [66].

The potential modulatory activity of the lead compounds against the GIRK4^WT^ and GIRK4^G151R^ channels requires in vitro validation. For example, in the study by Tauber et al., adrenocortical carcinoma NCI-H295R cells were transfected with WT or mutant *KCNJ5* containing plasmids [11]. Whole-cell patch clamp assays were performed to measure ion channel activity in the presence or absence of inhibitors [11]. The concentration of cytosolic Ca^2+^, Na^+^, and K^+^ was also measured [11]. In general, patch clamp assays are the standard in vitro assays for the validation of our findings. Appropriate in vivo experiments using relevant knockouts can then be performed. The overall efficacy of our lead compounds will depend on their concentrations required for bioactivity and their pharmacokinetic properties.

## 4. Conclusions

Overall, in silico tools were used to evaluate the binding and interactions of small molecules with the GIRK4^WT^ and GIRK4^G151R^ channels. In accordance with structural studies on Kir channels, the central cavity was identified as a potential ligand-binding site in homotetrameric GIRK4 channels. Verapamil and roxithromycin have previously been reported to have inhibitory activity against the GIRK4^G151R^ channel and were used as positive controls in this study. Molecular docking was performed to characterise the binding properties of the positive control inhibitors and over 600 olive-derived compounds from the OliveNet^TM^ library. The phenolic compounds, sterols, pigments, and triterpenic acids were predicted to bind strongly to the representative protein structures of the GIRK4^WT^ and GIRK4^G151R^ channels. The results revealed that the triterpenic acids ranked among the top 30 compounds for GIRK4^G151R^ compared to the GIRK4^WT^ channel. Based on the binding affinities and non-covalent interactions with the central cavity region, luteolin-7-O-rutinoside, pheophorbide a, and corosolic acid were identified as potential lead compounds for further in vitro investigation.

## Figures and Tables

**Table 1:** The number of compounds from the top 30 that were predicted to form non-covalent interactions with the GIRK4^WT^ channel.

Residue	Cluster 1	Cluster 2	Cluster 3
Y97	2	-	2
T146	4	4	2
E147	19	21	13
T148	1	2	3
T149	4	5	3
Q171	8	4	1
A172	8	7	2
S176	8	2	4
N179	8	14	5

**Table 2:** The number of compounds from the top 30 that were predicted to form non-covalent interactions with the GIRK4^G151R^ channel.

Residue	Cluster 1	Cluster 2	Cluster 3
Y97	2	2	5
W101	-	-	1
F142	-	1	-
T146	3	2	2
E147	11	11	10
T148	8	3	3
T149	6	1	3
R151	11	13	9
Q171	-	-	-
A172	2	5	5
G175	1	-	-
S176	2	2	5
N179	11	11	10

## Data Availability

The data presented in this study are available on request from the corresponding author.

## Supplementary Materials

The following supporting information can be downloaded at: https://www.mdpi.com/article/10.3390/biology13120992/s1, Table S1. Predicted binding affinities (kcal/mol) of the olive-derived compounds from the OliveNet^TM^ database against the central cavity of the GIRK4^WT^ structure that was obtained from the final frame of the equilibrated system; Table S2. Predicted binding affinities (kcal/mol) of the olive-derived compounds from the OliveNet^TM^ database against the central cavity of the GIRK4^G151R^ structure that was obtained from the final frame of the equilibrated system; Table S3. Potential ligand-binding sites in the representative protein structures of the GIRK4^WT^ and GIRK4^G151R^ channels were predicted using the PrankWeb server, with pocket 2 consisting of central cavity residues. The ligandability score of the second putative binding site is provided; Table S4. Predicted binding affinities of the olive-derived compounds against the representative protein structure for cluster 1 of the GIRK4^WT^ channel; Table S5. Predicted binding affinities of the olive-derived compounds against the representative protein structure for cluster 1 of the GIRK4^G151R^ channel; Table S6. Predicted binding affinities of the olive-derived compounds against the representative protein structure for cluster 2 of the GIRK4^WT^ channel; Table S7. Predicted binding affinities of the olive-derived compounds against the representative protein structure for cluster 2 of the GIRK4^G151R^ channel; Table S8. Predicted binding affinities of the olive-derived compounds against the representative protein structure for cluster 3 of the GIRK4^WT^ channel; Table S9. Predicted binding affinities of the olive-derived compounds against the representative protein structure for cluster 3 of the GIRK4^G151R^ channel; Table S10. Predicted non-covalent interactions of the top 30 compounds with the strongest binding affinities for the representative protein structure of cluster 1 (GIRK4^WT^ channel); Table S11. Predicted non-covalent interactions of the top 30 compounds with the strongest binding affinities for the representative protein structure of cluster 1 (GIRK4^G151R^ channel); Table S12. Predicted non-covalent interactions of the top 30 compounds with the strongest binding affinities for the representative protein structure of cluster 2 (GIRK4^WT^ channel); Table S13. Predicted non-covalent interactions of the top 30 compounds with the strongest binding affinities for the representative protein structure of cluster 2 (GIRK4^G151R^ channel); Table S14. Predicted non-covalent interactions of the top 30 compounds with the strongest binding affinities for the representative protein structure of cluster 3 (GIRK4^WT^ channel); Table S15. Predicted non-covalent interactions of the top 30 compounds with the strongest binding affinities for the representative protein structure of cluster 3 (GIRK4^G151R^ channel); Figure S1. Representative structures of the GIRK4^WT^ channel extracted from molecular dynamics (MD) simulations at 20 ns intervals; Figure S2. Representative structures of the GIRK4^G151R^ channel extracted from molecular dynamics (MD) simulations at 20 ns intervals; Figure S3. Root mean square fluctuation (RMSF) of protein backbone of WT and G151R GIRK4 following system equilibration; Figure S4. Binding characteristics of diltiazem against the central cavity of the GIRK4 channels; Figure S5. Binding characteristics of roxithromycin against the central cavity of the GIRK4 channels.
