# Supplementary material for: An In Silico Investigation of the Pathogenic G151R G Protein-Gated Inwardly Rectifying K+ Channel 4 Variant to Identify Small Molecule Modulators
Source: Biology (Basel). 2024 Nov 29;13(12):992. doi: 10.3390/biology13120992 (PMC11727529; doi:10.3390/biology13120992)
Supplement: Supplementary file 1 [file biology-13-00992-s001.zip › biology-3316474-supplementary.pdf]

An In Silico Investigation of the Pathogenic G151R G Protein-Gated Inwardly Rectifying K^+ Channel 4 Variant to Identify Small Molecule Modulators

Eleni Pitsillou[1,2], Julia J. Liang[1,3], Noa Kino[1,4], Jessica L. Lockwood[1,4], Andrew Hung[2], Assam El-Osta[3,5,6,7,8,9,10], Asmaa S. AbuMaziad[11], Tom C. Karagiannis[1,3,4,5,12,*]

[1] Epigenomic Medicine Laboratory at prospED Polytechnic, Melbourne, VIC 3053, Australia

[2] School of Science, STEM College, RMIT University, Melbourne, VIC 3001, Australia

[3] Epigenetics in Human Health and Disease Program, Baker Heart and Diabetes Institute, 75 Commercial Road, Melbourne, VIC 3004, Australia

[4] Department of Microbiology and Immunology, The University of Melbourne, Melbourne, VIC 3010, Australia

[5] Baker Department of Cardiometabolic Health, The University of Melbourne, Melbourne, VIC 3010, Australia

[6] Department of Diabetes, Central Clinical School, Monash University, Melbourne, VIC 3004, Australia

[7] Department of Medicine and Therapeutics, The Chinese University of Hong Kong, Sha Tin, Hong Kong SAR, China

[8] Hong Kong Institute of Diabetes and Obesity, Prince of Wales Hospital, The Chinese University of Hong Kong, 3/F Lui Che Woo Clinical Sciences Building, 30–32 Ngan Shing Street, Sha Tin, Hong Kong SAR, China

[9] Li Ka Shing Institute of Health Sciences, The Chinese University of Hong Kong, Sha Tin, Hong Kong SAR, China

[10] Biomedical Laboratory Science, Department of Technology, Faculty of Health, University College Copenhagen, 2200 Copenhagen, Denmark

[11] Department of Pediatrics, College of Medicine Tucson, The University of Arizona, Tucson, AZ 85724, USA

[12] Department of Clinical Pathology, The University of Melbourne, Melbourne, VIC 3010, Australia

* Author for Correspondence:
Dr Tom Karagiannis
Epigenomic in Human Health and Disease Program
Baker Heart and Diabetes Institute
75 Commercial Road, Prahran, VIC 3004, Australia
Email: karat@unimelb.edu.au Phone: +613 8532 1290 Fax: +613 8532 1100

Table S1. Predicted binding affinities (kcal/mol) of the olive-derived compounds from the OliveNet^{TM} database against the central cavity of the GIRK4^{WT} structure that was obtained from the final frame of the equilibrated system……………………………………………......3
Table S2. Predicted binding affinities (kcal/mol) of the olive-derived compounds from the OliveNet^{TM} database against the central cavity of the GIRK4^{G151R} structure that was obtained from the final frame of the equilibrated system……………………………………….......20
Table S3. Potential ligand-binding sites in the representative protein structures of the GIRK4^{WT} and GIRK4^{G151R} channels were predicted using the PrankWeb server, with pocket 2 consisting of central cavity residues. The ligandability score of the second putative binding site is provided……………………………....……………………………………….......37
Table S4. Predicted binding affinities of the olive-derived compounds against the representative protein structure for cluster 1 of the GIRK4^{WT} channel……………………..39
Table S5. Predicted binding affinities of the olive-derived compounds against the representative protein structure for cluster 1 of the GIRK4^{G151R} channel……………………56
Table S6. Predicted binding affinities of the olive-derived compounds against the representative protein structure for cluster 2 of the GIRK4^{WT} channel………………………73
Table S7. Predicted binding affinities of the olive-derived compounds against the representative protein structure for cluster 2 of the GIRK4^{G151R} channel……………………90
Table S8. Predicted binding affinities of the olive-derived compounds against the representative protein structure for cluster 3 of the GIRK4^{WT} channel……………..……..107
Table S9. Predicted binding affinities of the olive-derived compounds against the representative protein structure for cluster 3 of the GIRK4^{G151R} channel……………….…..124
Table S10. Predicted non-covalent interactions of the top 30 compounds with the strongest binding affinities for the representative protein structure of cluster 1 (GIRK4^{WT} channel)…141
Table S11. Predicted non-covalent interactions of the top 30 compounds with the strongest binding affinities for the representative protein structure of cluster 1 (GIRK4^{G151R} channel)..143
Table S12. Predicted non-covalent interactions of the top 30 compounds with the strongest binding affinities for the representative protein structure of cluster 2 (GIRK4^{WT} channel)…145
Table S13. Predicted non-covalent interactions of the top 30 compounds with the strongest binding affinities for the representative protein structure of cluster 2 (GIRK4^{G151R} channel).147
Table S14. Predicted non-covalent interactions of the top 30 compounds with the strongest binding affinities for the representative protein structure of cluster 3 (GIRK4^{WT} channel)…149
Table S15. Predicted non-covalent interactions of the top 30 compounds with the strongest binding affinities for the representative protein structure of cluster 3 (GIRK4^{G151R} channel)……………………………………………………………………………..151
Figure S1. Representative structures of the GIRK4^{WT} channel extracted from molecular dynamics (MD) simulations at 20 ns intervals……………………………………………153
Figure S2. Representative structures of the GIRK4^{G151R} channel extracted from molecular dynamics (MD) simulations at 20 ns intervals……………………………………………154
Figure S3. Root mean square fluctuation (RMSF) of protein backbone of WT and G151R GIRK4 following system equilibration…………………………………………………..155
Figure S4. Binding characteristics of diltiazem against the central cavity of GIRK4 channels. …………………………………………………………………………………... 156
Figure S5. Binding characteristics of roxithromycin against the central cavity of GIRK4 channels……………………………………………………………………………..157

Table S1. Predicted binding affinities (kcal/mol) of the olive-derived compounds from the OliveNet™ database against the central cavity of the GIRK4^{WT} structure that was obtained from the final frame of the equilibrated system.

OliveNet™ class	Compound	Binding Affinity (kcal/mol)
Pigments	Pheophytin b	-11.5
Phenolics	Luteolin-7-O-rutinoside	-11.1
Pigments	Chlorophyllide a	-11.1
Phenolics	Luteolin-4'-O-rutinoside	-10.9
Pigments	Chlorophyllide b	-10.9
Pigments	Chlorophyll a	-10.8
Phenolics	Hesperidin	-10.7
Phenolics	β-Hydroxy-acetoside	-10.7
Phenolics	Apigenin-7-O-rutinoside	-10.6
Phenolics	Isorhoifolin	-10.6
Phenolics	Ligstroside derivative 4	-10.6
Phenolics	Ligstroside derivative 5	-10.6
Phenolics	Nüzhenide 11-Methyl oleoside	-10.6
Phenolics	Orbanchoside	-10.6
Phenolics	Rutin	-10.6
Phenolics	Scolymoside	-10.6
Phenolics	Suspensaside	-10.6
Phenolics	Verbascoside	-10.6
Phenolics	β-Hydroxy verbascoside	-10.6
Phenolics	Acetoside	-10.5
Phenolics	Isoacteoside	-10.5
Phenolics	Luteolin-3′,7-O-diglucoside	-10.5
Phenolics	Cyanidin-3-O-rutinoside	-10.3
Phenolics	Oxidized isoverbascoside	-10.3
Phenolics	Luteolin-7,4-O-diglucoside	-10.2
Phenolics	Nüzhenide oleoside	-10.2
Phenolics	Chrysoeriol-7-O-glucoside	-10.1
Phenolics	Hellicoside	-10.1
Phenolics	Jaspolyanoside	-10.1
Phenolics	Luteolin-4’-O-glucoside	-10.1
Phenolics	Oxidized verbascoside	-10.1
Phenolics	Quercetin-7-O-glucoside	-10.1
Phenolics	Apigenin-7-O-glucoside	-10.0
Phenolics	Isojaspolyoside A	-10.0
Phenolics	Isoverbascoside	-10.0
Phenolics	Jaspolyoside	-10.0
Phenolics	Oleuropein dimer	-10.0
Phenolics	Quercetin 3-O-rutinoside	-10.0
Phenolics	Luteolin-7-O-glucoside	-9.9
Phenolics	Oleuricine A	-9.9
Pigments	Chlorophyll b	-9.9
Pigments	Pheophorbide a	-9.9

Pigments	Pheophorbide b	-9.8
Phenolics	Cyanidin-3-O-glucoside	-9.7
Phenolics	Delphinidin-3-O-glucoside	-9.7
Phenolics	Luteolin-8-C-glucoside	-9.6
Phenolics	Nüzhenide	-9.6
Phenolics	Oleuropein diglucoside	-9.6
Pigments	Pheophytin α	-9.6
Phenolics	(+)-1-Acetoxypinoresinol-4'-β-D-glucopyranoside	-9.5
Phenolics	4'-O-β-D-Glucosyl-9-O-(6"-deoxysaccharosyl)olivil	-9.5
Phenolics	Luteolin-6-C-glucoside	-9.5
Phenolics	Wedelosin	-9.5
Phenolics	Ligstroside derivative 3	-9.4
Phenolics	Neo-nüzhenide	-9.4
Phenolics	Oleuropein	-9.4
Pigments	Pyropheophytin α	-9.4
Sterols	α-amyrin	-9.4
Phenolics	6'-Rhamnopyranosyl oleoside	-9.3
Phenolics	Caffeoyl-6'-secologanoside	-9.3
Phenolics	Elenolic acid diglucoside	-9.3
Phenolics	Ligstroside	-9.3
Triterpenic acids	Pomolic acid	-9.3
Triterpenic acids	Urs-2β,3β-dihydroxy-12-en-28-oic acid	-9.3
Triterpenic acids	Ursolic acid	-9.3
Sterols	28-isocitrostadienol	-9.3
Sterols	28-nor-β-amyrin	-9.3
Sterols	Taraxasterol	-9.3
Phenolics	(+)-1-Acetoxypinoresinol-4"-O-methyl ether	-9.2
Phenolics	(+)-1-Acetoxypinoresinol-4'-β-D-glucopyranoside-4"-O-methyl ether	-9.2
Phenolics	Vicenin-2	-9.2
Sterols	Stigmastanol	-9.2
Sterols	Δ-5,24-Stigmastadienol	-9.2
Sterols	24-Ethyl-E-23-dehydrolophenol	-9.2
Sterols	24-methyl-(E)-23-dehydrolophenol	-9.2
Sterols	24-methyl-24(25)-dehydrolophenol	-9.2
Sterols	β-amyrone	-9.2
Sterols	Ψ-taraxasterol	-9.2
Phenolics	10-Hydroxyoleuropein	-9.1
Phenolics	Quercetin-3-rhamnoside	-9.1
Sterols	24-ethyllophenol	-9.1
Sterols	Lupenone	-9.1
Phenolics	(+)-1-Acetoxypinoresinol-4'-β-D-glucopyranoside	-9.0
Phenolics	6'-β-D-Glucopyranosyl oleoside	-9.0
Phenolics	Ligstroside derivative 1	-9.0

Phenolics	Oleuricine B	-9.0
Phenolics	Quercitrin	-9.0
Triterpenic acids	Corosolic acid	-9.0
Triterpenic acids	Oleanolic acid demethyl	-9.0
Sterols	Brassicasterol	-9.0
Sterols	Ergosterol	-9.0
Sterols	4α,14α-Dimethylstigmasta-8,24(24)-dien-3β-ol	-9.0
Phenolics	Lucidumoside C	-8.9
Phenolics	Oleuropein-3"-Methyl ether	-8.9
Phenolics	Oleuropein-3'-O-β-D-glucopyranoside	-8.9
Phenolics	Syringaresinol	-8.9
Sterols	Δ-5-Avenasterol	-8.9
Phenolics	(+)-Fraxiresinol-1-β-D-glucopyranoside	-8.8
Phenolics	Oleuroside-10-carboxylic acid	-8.8
Phenolics	Quercetin-3-O-glucoside	-8.8
Triterpenic acids	3-epi-betulinic acid	-8.8
Triterpenic acids	Betulinic acid	-8.8
Sterols	24-Methylene-cholesterol	-8.8
Sterols	Campesterol	-8.8
Sterols	Δ7,22-Ergostadienol	-8.8
Sterols	28-nor-α-amyrin	-8.8
Sterols	4,4-dimethyl-5α-stigmast-7-en-3β-ol	-8.8
Sterols	Bacchar-12,21-dien-3β-ol	-8.8
Sterols	Uvaol	-8.8
Phenolics	Demethyloleuropein	-8.7
Phenolics	Hydroxypinoresinol	-8.7
Sterols	β-sitosterol	-8.7
Sterols	Δ-5,23-Stigmastadienol	-8.7
Sterols	28-hydroxytaraxerol	-8.7
Sterols	3-epi-lupeol	-8.7
Sterols	Lupeol	-8.7
Sterols	β-amyrin	-8.7
Phenolics	(+)-1-Acetoxypinoresinol-4-O-methyl ether	-8.6
Phenolics	Demethylligstroside	-8.6
Phenolics	Methoxyluteolin	-8.6
Phenolics	Pinoresinol	-8.6
Triterpenic acids	Oleanolic acid	-8.6
Sterols	22,23- Dihydrobrassicasterol	-8.6
Sterols	Δ7,24-Ergostadienol	-8.6
Sterols	Δ-7-Avenasterol	-8.6
Sterols	24-methylene-24-dihydroparkeol	-8.6
Sterols	Cyclosadol	-8.6
Sterols	Methyl 2α,3β-diacetoxyolean-12-en-28-oate	-8.6
Sterols	Methyl 3β-acetoxyolean-12-en-28-oate	-8.6
Sterols	δ-amyrin	-8.6

Phenolics	1-Acetoxypinoresinol	-8.5
Phenolics	6'-O-[(2E)-2,6-Dimethyl-8-hydroxy- 2-octenoyloxy]-secologanoside	-8.5
Phenolics	Esculin	-8.5
Sugars	D-(+)-raffinose	-8.5
Triterpenic acids	Maslinic acid	-8.5
Sterols	3-epi-betulin	-8.5
Sterols	Cycloartenol	-8.5
Sterols	Taraxerol	-8.5
Phenolics	1-(3’-Methoxy-4’-hydroxy)- phenyl-6,7-dihydroxyisochroman	-8.4
Phenolics	Chrysoeriol	-8.4
Phenolics	Demethyloleuropein aglycone	-8.4
Phenolics	Dihydro-oleuropein	-8.4
Phenolics	Diosmetin	-8.4
Phenolics	Ligstroside derivative 2	-8.4
Phenolics	Quercetin	-8.4
Phenolics	Taxifolin	-8.4
Phenolics	Verucosin	-8.4
Sterols	Stigmasterol	-8.4
Sterols	Δ-7-Stigmastenol	-8.4
Sterols	24-Methylenelophenol	-8.4
Sterols	Gramisterol	-8.4
Sterols	Erythrodiol	-8.4
Phenolics	Comselogoside	-8.3
Phenolics	Eriodictyol	-8.3
Phenolics	Hesperitin	-8.3
Phenolics	Hydroxytyrosol diglucoside	-8.3
Phenolics	Ligstroside-3'-O-β-D-glucopyranoside	-8.3
Phenolics	Luteolin	-8.3
Phenolics	Oleuroside	-8.3
Sugars	Mannan	-8.3
Sterols	24-methylene-cycloartenol	-8.3
Sterols	Germanicol	-8.3
Phenolics	(-)-Olivil	-8.2
Phenolics	3-Acetyloxy berchemol	-8.2
Phenolics	Chlorogenic acid	-8.2
Phenolics	Delphinidin	-8.2
Phenolics	Ligstroside aglycone methyl acetal	-8.2
Phenolics	Oleoside-11-Methylester	-8.2
Phenolics	Oleuropein aglycone (3,4-DHPEA-EA)	-8.2
Sterols	Cholesterol	-8.2
Sterols	Citrostadienol	-8.2
Sterols	24-methylene-24-dihydroparkenol	-8.2
Sterols	Agrostophyllinol	-8.2
Phenolics	10-Hydroxy oleuropein aglycone	-8.1
Phenolics	Apigenin	-8.1
Phenolics	Berchemol	-8.1

Phenolics	Cyanidin (cation)	-8.1
Phenolics	Oleoside	-8.1
Phenolics	Rosmarinic acid	-8.1
Sterols	24-methylene-31-nor-9(11)-lanostenol	-8.1
Sterols	Cyclobranol	-8.1
Sterols	Dammaradienol	-8.1
Phenolics	Ligstroside aglycone	-8.0
Phenolics	Monoaldehydic form of Oleuropein aglycon	-8.0
Phenolics	Oleoside dimethylester	-8.0
Phenolics	Oleuropeindial - Lactone (Cannizzaro-like product of oleuropeindial)	-8.0
Sugars	Maltotriose	-8.0
Tocopherols	α-tocopherol	-8.0
Tocopherols	β-tocopherol	-8.0
Sterols	24-methyl-31-nor-9(11)-lanostenol	-8.0
Sterols	7, 24-tirucalladienol	-8.0
Sterols	Butyrospermol	-8.0
Sterols	Parkeol	-8.0
Phenolics	10-Hydroxy oleuropein aglycone decarboxymethyl	-7.9
Phenolics	10-Hydroxyoleuropein	-7.9
Phenolics	Caffeoylglucose	-7.9
Phenolics	Scopolin	-7.9
Other	Poly-unsaturated di-galactoside glycerol diester	-7.9
Sterols	(24Z)-24-ethylidene-dihydrolanosterol	-7.9
Sterols	24-methylene-24-dihydrolanosterol	-7.9
Sterols	4,4-dimethyl-5α-stigmasta-7,24Z(241)-dien-3β-ol	-7.9
Phenolics	7-Deoxyloganic acid	-7.8
Phenolics	Loganic acid	-7.8
Phenolics	Loganin	-7.8
Phenolics	Oleuropeindial (enol form)	-7.8
Sterols	24-Ethylidenelophenol	-7.8
Sterols	Tirucallol	-7.8
Phenolics	Elenolic acid glucoside	-7.7
Phenolics	Hydroxytyrosil-elenolate	-7.7
Phenolics	Hydroxytyrosol-1′-β-glucoside	-7.7
Phenolics	Monoaldehydic form of Ligstroside aglycon	-7.7
Phenolics	Secologanin	-7.7
Sterols	Clerosterol	-7.7
Phenolics	10-Hydroxy-10-methyl oleuropein aglycone	-7.6
Phenolics	Secologanoside	-7.6
Tocopherols	γ-tocopherol	-7.6
Sterols	Cycloeucalenol	-7.6

Phenolics	Cornoside	-7.5
Phenolics	(+)-Cycloolivil	-7.4
Phenolics	1-Phenyl-6,7-dihydroxyisochroman	-7.4
Phenolics	3,4-DHPEA-DETA	-7.4
Phenolics	Caftaric acid	-7.4
Phenolics	Oleuropeindial (Cannizzaro-like product of oleuropeindial)	-7.4
Phenolics	Oleuropeindial (keto form)	-7.4
Phenolics	Secologanol	-7.4
Sugars	Galactinol	-7.4
Sterols	Campestanol	-7.4
Phenolics	DHPEA-DEDA	-7.3
Phenolics	Hydroxytyrosol rhamnoside	-7.3
Phenolics	Hydroxytyrosol-3-β-glucoside	-7.3
Phenolics	Hydroxytyrosol-4-β-glucoside	-7.3
Phenolics	Oleacein (Dialdehydic form of decarboxymethyl Oleuropein aglycon)	-7.3
Phenolics	Salidroside	-7.3
Hydrocarbons	Squalene	-7.3
Sterols	Obtusifoliol	-7.3
Phenolics	3,4-DHPEA-EDA (Oleuropein-aglycone di-aldehyde)	-7.2
Phenolics	Demethyloleuropein aglycone dialdehyde	-7.2
Phenolics	Secologanic acid	-7.2
Sugars	D-(+)-sucrose	-7.2
Phenolics	Hydroxytyrosol acyclodihydroelenolate	-7.1
Sugars	α-Cellulose	-7.1
Phenolics	demethyloleuropein aglycone (enol form)	-7.0
Phenolics	DHPEA-DEDA (acetal)	-7.0
Phenolics	Oleocanthal (Dialdehydic form of decarboxymethyl Ligstroside aglycon)	-7.0
Hydrocarbons	Geranylgeranyl C24:0	-7.0
Sugars	D-(+)-lactose	-7.0
Phospholipids	Phosphatidylinositol	-6.9
Aliphatic and aromatic	Geranylgeraniol	-6.8

Hydrocarbons	α-Selinene	-6.7
Tocopherols	δ-tocopherol	-6.7
Phenolics	D-(+)-Erythro-1-(4-hydroxy-3-methoxy)-214 - phenyl-1,2,3-propantriol	-6.6
Phenolics	Hemiacetal of dialdehydic ligstroside aglycone decarboxymethyl	-6.6
Phenolics	Methyl malate-hydroxytyrosol ester	-6.6
Hydrocarbons	Geranylgeranyl C20:1	-6.6
Hydrocarbons	Geranylgeranyl C22:0	-6.6
Phospholipids	Lysophosphatidylethanolamine	-6.6
Phenolics	1-oleyltyrosol	-6.5
Phenolics	Decarboxymethyl ligstroside aglycone	-6.5
Phenolics	Demethyl elenolic acid	-6.5
Phenolics	Deoxyloganic acid lauryl ester	-6.5
Phenolics	Esculetin	-6.5
Phenolics	Hemiacetal of dialdehydic oleuropein aglycone decarboxymethyl	-6.5
Hydrocarbons	β-cubebene	-6.5
Hydrocarbons	Phytyl oleate C18:1	-6.5
Hydrocarbons	(E)-caryophyllene	-6.4
Hydrocarbons	Alloaromadendrene	-6.4
Hydrocarbons	Eremophyllene	-6.4
Phenolics	Caffeic acid	-6.3
Phenolics	Hydroxycaffeic acid	-6.3
Hydrocarbons	γ-Muurolene	-6.3
Hydrocarbons	β-Curcumene	-6.3
Hydrocarbons	Phytyl C20:1	-6.3
Phenolics	Ferulic acid	-6.2
Phenolics	Tyrosol acetate	-6.2
Hydrocarbons	α-Zingiberene	-6.2
Hydrocarbons	Geranylgeranyl C20:0	-6.2
Hydrocarbons	Geranylgeranyl oleate C18:0	-6.2
Hydrocarbons	Geranylgeranyl oleate C18:1	-6.2
Hydrocarbons	Phytyl C22:0	-6.2
Phospholipids	Lysophosphatidic acid	-6.2
Sugars	Pectin	-6.2
Phenolics	Hydroxytyrosol acetate	-6.1
Phenolics	p-HPEA-EDA	-6.1

Phenolics	Scopoletin	-6.1
Hydrocarbons	(Z)2,(E)4,(E)6-Allofarnesene	-6.1
Hydrocarbons	Longicyclene	-6.1
Hydrocarbons	Drima-7,9(11)-diene	-6.1
Hydrocarbons	α-copaene	-6.1
Hydrocarbons	β- Sesquiphellandrene	-6.1
Hydrocarbons	β-acoradiene	-6.1
Hydrocarbons	β-elemene	-6.1
Hydrocarbons	Phytyl C20:0	-6.1
Volatiles	trans-β-Damascenone	-6.1
Phenolics	Gallic acid	-6.0
Phenolics	Homovanillic acid	-6.0
Phenolics	Quinic acid	-6.0
Phenolics	Sinapic acid	-6.0
Aliphatic and aromatic	Phytol	-6.0
Fatty acids	Linolenic	-6.0
Hydrocarbons	Triacontane	-6.0
Hydrocarbons	γ-curcumene	-6.0
Hydrocarbons	(E)-β-farnesene	-6.0
Hydrocarbons	Eremophilone	-6.0
Other	3-[1-(hydroxymethyl)-(E)-1-propenyl] glutaric acid	-6.0
Phenolics	2,3-dihydrocaffeic acid	-5.9
Phenolics	4-O-methyl-D-glucuronic acid	-5.9
Phenolics	Elenolic acid	-5.9
Phenolics	Elenolic acid dialdehyde	-5.9
Phenolics	Elenolic acid methylester	-5.9
Phenolics	*p*-Coumaric acid	-5.9
Phenolics	Protocatechuic acid	-5.9
Hydrocarbons	Calarene	-5.9
Hydrocarbons	Cyclosativene	-5.9
Hydrocarbons	α-trans-bergamotene	-5.9
Hydrocarbons	Phytyl oleate C18:0	-5.9
Hydrocarbons	Wax ester 46:0 (22:0-24:0)	-5.9
Phenolics	3,4-Dihydroxyphenylacetic acid	-5.8
Phenolics	3,4-Dihydroxyphenylglycol	-5.8
Phenolics	3,4-Dimethoxybenzoic acid	-5.8
Phenolics	Isoeugenol	-5.8
Phenolics	Vanillic acid	-5.8
Amino acids	Tyrosine	-5.8
Fatty acids	Ethyl linoleate	-5.8
Fatty acids	Ethyl oleate	-5.8

Hydrocarbons	Wax ester 42:1 (16:1-26:0)	-5.8
Hydrocarbons	Wax ester 44:1 (20:1-24:0)	-5.8
Hydrocarbons	Wax ester 46:0 (20:0-26:0)	-5.8
Sugars	D-(+)-galacturonic acid	-5.8
Sugars	D-glucuronic acid	-5.8
Volatiles	cis-3-Hexenyl acetate	-5.8
Phenolics	4-Hydroxy-3-methoxy-phenylacetic acid	-5.7
Phenolics	Dihydro-*p*-coumaric acid	-5.7
Phenolics	Homovanillin	-5.7
Phenolics	*m*-Coumaric acid	-5.7
Phenolics	*o*-Coumaric acid	-5.7
Phenolics	Phloretic acid	-5.7
Phenolics	Shikimic acid	-5.7
Fatty acids	Methyl heptadecanoate	-5.7
Fatty acids	11-cis-vaccenic	-5.7
Fatty acids	Behenic	-5.7
Fatty acids	Oleic	-5.7
Fatty acids	Trans-palmitoleic	-5.7
Hydrocarbons	(E)2,(Z)4,(E)6-Allofarnesene	-5.7
Hydrocarbons	δ-cadinene	-5.7
Sugars	Galacturonan	-5.7
Volatiles	Ethyl cinnamate	-5.7
Phenolics	2,5-Dihydroxyphenylacetic acid	-5.6
Phenolics	2,6-Dimethoxybenzoic acid	-5.6
Phenolics	3,4,5-Trimethoxybenzoic acid	-5.6
Phenolics	Gentisic acid	-5.6
Phenolics	Homoveratric acid	-5.6
Phenolics	p-Hydroxyphenylacetic acid	-5.6
Phenolics	Syringic acid	-5.6
Amino acids	Phenylalanine	-5.6
Fatty acids	Methyl linoleate	-5.6
Fatty acids	Elaidic	-5.6
Fatty acids	Linoelaidic	-5.6
Fatty acids	Palmitic	-5.6
Hydrocarbons	Wax ester 40:1 (18:1-22:0)	-5.6
Hydrocarbons	Wax ester 46:0 (14:0-32:0)	-5.6
Sugars	myo-inositol	-5.6
Volatiles	Phenethyl acetate	-5.6
Phenolics	2,4 dihydroxybenzoic acid	-5.5
Phenolics	DEDA acetal	-5.5
Phenolics	Homovanillyl alcohol	-5.5
Fatty acids	Ethyl stearate	-5.5

Fatty acids	Eicosenoic	-5.5
Fatty acids	Gadoleic	-5.5
Fatty acids	Linoleic	-5.5
Fatty acids	Margaric acid	-5.5
Fatty acids	Petroselinic	-5.5
Hydrocarbons	Tetratriacontane	-5.5
Hydrocarbons	Methyl oleate	-5.5
Hydrocarbons	Methyl palmitate	-5.5
Hydrocarbons	Wax ester 40:0 (16:0-24:0)	-5.5
Hydrocarbons	Wax ester 44:0 (16:0-28:0)	-5.5
Hydrocarbons	Wax ester 44:0 (18:0-26:0)	-5.5
Hydrocarbons	Wax ester 44:0 (22:0-22:0)	-5.5
Hydrocarbons	Wax ester 46:0 (16:0-30:0)	-5.5
Other	3-(1-Hydroxymethyl-1-propenyl)pentanedioic acid	-5.5
Phospholipids	Phosphatidylcholine	-5.5
Sugars	D-(+)-mannose	-5.5
Sugars	Sedoheptulose	-5.5
Volatiles	2-Ethylphenyl acetate	-5.5
Phenolics	Cinnamic acid	-5.4
Phenolics	Hydroxytyrosol	-5.4
Fatty acids	Methyl stearate	-5.4
Fatty acids	Erucic	-5.4
Fatty acids	Palmitoleic	-5.4
Fatty acids	Stearic	-5.4
Hydrocarbons	Ethyl oleate	-5.4
Hydrocarbons	Wax ester 40:0 (18:0-22:0)	-5.4
Hydrocarbons	Wax ester 40:0 (20:0-20:0)	-5.4
Other	Halleridone	-5.4
Sugars	D-(-)-fructose	-5.4
Sugars	D-(+)-glucose	-5.4
Sugars	D-(+)-chiro-inositol	-5.4
Sugars	D-Fucose	-5.4
Volatiles	Terpineol	-5.4
Volatiles	Vanillin	-5.4
Phenolics	2,6-Dihydroxybenzoic acid	-5.3
Phenolics	4-Ethylguaiacol	-5.3
Phenolics	4-hydroxybenzoic acid	-5.3
Phenolics	Syringaldehyde	-5.3
Amino acids	Arginine	-5.3

Fatty acids	Arachidic	-5.3
Fatty acids	Cis-Heptadecenoic	-5.3
Hydrocarbons	Tricosane	-5.3
Hydrocarbons	9-Docosene	-5.3
Hydrocarbons	Wax ester 38:0 (18:0-20:0)	-5.3
Hydrocarbons	Wax ester 40:1 (16:1-24:0)	-5.3
Hydrocarbons	Wax ester 42:0 (20:0-22:0)	-5.3
Hydrocarbons	Wax ester 42:0 (24:0-18:0)	-5.3
Hydrocarbons	Wax ester 44:1 (18:1-26:0)	-5.3
Hydrocarbons	Wax ester 46:0 (18:0-28:0)	-5.3
Hydrocarbons	Wax ester 46:1 (18:1-28:0)	-5.3
Other	3-[1-(formyl)-(E)-1-propenyl] glutaric acid	-5.3
Phospholipids	Phosphatidylglycerol	-5.3
Sugars	D-(-)-galactose	-5.3
Volatiles	Ethyl cyclohexylcarboxylate	-5.3
Volatiles	3-Methyl-2-pentylfuran	-5.3
Volatiles	2-Ethyl-5-hexylthiophene	-5.3
Phenolics	2-Methoxy-4-vinylphenol	-5.2
Phenolics	4-Vinylguaiacol	-5.2
Aliphatic and aromatic	Eicosanol	-5.2
Aliphatic and aromatic	Hexacosanol	-5.2
Aliphatic and aromatic	Pentacosanol	-5.2
Fatty acids	Methyl palmitate	-5.2
Fatty acids	Lignoceric	-5.2
Fatty acids	Myristic	-5.2
Hydrocarbons	Docosane	-5.2
Hydrocarbons	Heptacosane	-5.2
Hydrocarbons	9-Heptacosene	-5.2
Hydrocarbons	9-Tetracosene	-5.2
Hydrocarbons	Phytyl C24:0	-5.2
Hydrocarbons	Wax ester 38:0 (12:0-26:0)	-5.2
Hydrocarbons	Wax ester 38:0 (16:0-22:0)	-5.2
Hydrocarbons	Wax ester 42:0 (14:0-28:0)	-5.2
Hydrocarbons	Wax ester 42:0 (16:0-26:0)	-5.2
Hydrocarbons	Wax ester 46:0 (24:0-22:0)	-5.2
Phospholipids	Phosphatidylethanolamine	-5.2
Sugars	1,6-anhydro-β-D-glucose	-5.2

Sugars	L-rhamnose	-5.2
Sugars	L-Fucose	-5.2
Volatiles	Benzyl acetate	-5.2
Phenolics	DEDA	-5.1
Phenolics	Dialdehydic elenolic acid decarboxymethl	-5.1
Phenolics	Dialdehydic elenolic ester decarboxymethyl	-5.1
Phenolics	Tyrosol	-5.1
Aliphatic and aromatic	Tetracosanol	-5.1
Amino acids	Glutamine	-5.1
Hydrocarbons	Dotriacontane	-5.1
Hydrocarbons	Hentriacontane	-5.1
Hydrocarbons	Heptadecane	-5.1
Hydrocarbons	Nonacosane	-5.1
Hydrocarbons	Octacosane	-5.1
Hydrocarbons	Pentacosane	-5.1
Hydrocarbons	8-Heptadecene	-5.1
Hydrocarbons	9-Tricosene	-5.1
Hydrocarbons	Wax ester 38:0 (14:0-24:0)	-5.1
Hydrocarbons	Wax ester 42:1 (18:1-24:0)	-5.1
Hydrocarbons	Wax ester 44:1 (16:0-28:0)	-5.1
Volatiles	1,8-Cineole	-5.1
Volatiles	Citric acid	-5.1
Sterols	Linalool	-5.1
Aliphatic and aromatic	Docosanol	-5.0
Aliphatic and aromatic	Heptacosanol	-5.0
Aliphatic and aromatic	Tricosanol	-5.0
Fatty acids	Methyl oleate	-5.0
Fatty acids	Ethyl palmitate	-5.0
Hydrocarbons	Icosane	-5.0
Hydrocarbons	Tetracosane	-5.0
Hydrocarbons	6,10-dimethyl-1-undecene	-5.0
Hydrocarbons	9-Hexacosene	-5.0
Hydrocarbons	9-Pentacosene	-5.0
Phospholipids	Phosphatidic acid	-5.0
Sugars	D-(-)-arabinose	-5.0
Volatiles	2,4-Decadienal	-5.0
Volatiles	trans-4,5-Epoxy-trans-2-decenal	-5.0
Volatiles	Acetophenone	-5.0

Volatiles	Gluconic acid	-5.0
Phenolics	4-Ethylphenol	-4.9
Phenolics	4-Methylcatechol	-4.9
Phenolics	4-Vinylphenol	-4.9
Aliphatic and aromatic	Octacosanol	-4.9
Hydrocarbons	Heneicosane	-4.9
Hydrocarbons	Hexacosane	-4.9
Hydrocarbons	Nonadecane	-4.9
Hydrocarbons	Octadecane	-4.9
Hydrocarbons	Pentadecane	-4.9
Hydrocarbons	Tetradecane	-4.9
Hydrocarbons	(Z)-β-farnesene	-4.9
Hydrocarbons	Wax ester 40:0 (14:0-26:0)	-4.9
Hydrocarbons	Wax ester 42:0 (18:0-24:0)	-4.9
Sugars	D-(+)-xylose	-4.9
Volatiles	Lavendulol	-4.9
Volatiles	trans,trans-2,4-Nonadienal	-4.9
Volatiles	3,4-methyl-3-pentenyl furan	-4.9
Phenolics	4-Hydroxybenzaldehyde	-4.8
Hydrocarbons	Tridecane	-4.8
Hydrocarbons	Tritriacontane	-4.8
Hydrocarbons	6,10-Dimethyl-l-undecane	-4.8
Hydrocarbons	Methyl stearate	-4.8
Hydrocarbons	Wax ester 38:0 (20:0-18:0)	-4.8
Volatiles	2-Phenylethanol	-4.8
Volatiles	cis-1,5-Octadien-3-one	-4.8
Phenolics	Guaiacol	-4.7
Sugars	D-Mannitol	-4.7
Volatiles	6-Methyl-5-hepten-3-ol	-4.7
Volatiles	trans,trans-2,4-Decadienal	-4.7
Volatiles	trans-2-Nonenal	-4.7
Volatiles	2-Hexenyl acetate	-4.7
Volatiles	Octyl acetate	-4.7
Volatiles	6-Methyl-5-hepten-2-one	-4.7
Phenolics	p-cresol	-4.6
Amino acids	Glutamic acid	-4.6
Fatty acids	Lauric	-4.6
Other	1,5-anhydroxylitol	-4.6
Volatiles	2-Methylpropyl butanoate	-4.6
Volatiles	3-Hexenyl acetate	-4.6
Volatiles	Ethyl 2-methylbutanoate	-4.6
Volatiles	Ethyl hexanoate	-4.6

Volatiles	Ethyl octanoate	-4.6
Volatiles	2-Octanone	-4.6
Phenolics	Catechol	-4.5
Phenolics	m-cresol	-4.5
Phenolics	o-cresol	-4.5
Amino acids	Isoleucine	-4.5
Amino acids	Leucine	-4.5
Amino acids	Valine	-4.5
Fatty acids	1,3-diacylglycerol	-4.5
Fatty acids	2,3-diacylglycerol	-4.5
Sugars	L-(-)-arabitol	-4.5
Volatiles	3-Octenol	-4.5
Volatiles	Phenylacetaldehyde	-4.5
Volatiles	2-Methylbutyl acetate	-4.5
Volatiles	Ethyl 3-methylbutanoate	-4.5
Volatiles	Methyl 2-methylbutanoate	-4.5
Volatiles	Propyl 2-methylpropanoate	-4.5
Volatiles	3-Octanone	-4.5
Volatiles	l-Octen-3-one	-4.5
Volatiles	2,5-Diethylthiophene	-4.5
Amino acids	Aspargine	-4.4
Amino acids	Aspartic acid	-4.4
Fatty acids	1,2-diacylglycerol	-4.4
Hydrocarbons	Styrene	-4.4
Sugars	Adonitol	-4.4
Sugars	Xylitol	-4.4
Volatiles	2-Octanol	-4.4
Volatiles	Benzyl alcohol	-4.4
Volatiles	Benzaldehyde	-4.4
Volatiles	Ethyl isobutyrate	-4.4
Volatiles	Ethyl-2-methypropanoate	-4.4
Volatiles	Hexyl acetate	-4.4
Volatiles	Isopentyl acetate	-4.4
Volatiles	Methyl 3-methylbutanoate	-4.4
Volatiles	Methyl heptanoate	-4.4
Volatiles	Methyl octanoate	-4.4
Volatiles	Propyl butanoate	-4.4
Volatiles	2-Heptanone	-4.4
Volatiles	Heptan-2-one	-4.4
Volatiles	Octan-2-one	-4.4
Volatiles	Malic acid	-4.4
Volatiles	Decanol	-4.3
Volatiles	Decanal	-4.3

Volatiles	Nonanal	-4.3
Volatiles	trans,cis-2,4-Decadienal	-4.3
Volatiles	trans-2-Decenal	-4.3
Volatiles	trans-2-Octenal	-4.3
Volatiles	Octanoic acid	-4.3
Volatiles	Ethyl butanoate	-4.3
Volatiles	Methyl hexanoate	-4.3
Volatiles	2-Nonanone	-4.3
Volatiles	4-Methyl-2-pentanone	-4.3
Hydrocarbons	Methyl benzene	-4.2
Hydrocarbons	Tridecane	-4.2
Volatiles	2-Heptanol	-4.2
Volatiles	Octanol	-4.2
Volatiles	2,4 Hexadienal	-4.2
Volatiles	3-Hexanal	-4.2
Volatiles	cis-2-Heptenal	-4.2
Volatiles	cis-2-Nonenal	-4.2
Volatiles	3-Methyl-butanoic acid	-4.2
Volatiles	3-Methylbutyric acid	-4.2
Volatiles	Heptanoic acid	-4.2
Volatiles	Hexanoic acid	-4.2
Volatiles	Butyl acetate	-4.2
Volatiles	2-Propylfuran	-4.2
Volatiles	3-Propylfuran	-4.2
Volatiles	3-Isopropenylthiophene	-4.2
Phenolics	Phenol	-4.1
Fatty acids	1-monoacylglycerol	-4.1
Hydrocarbons	Dodecane	-4.1
Hydrocarbons	Hexadecane	-4.1
Volatiles	cis-3-Hexenol	-4.1
Volatiles	Nonanol	-4.1
Volatiles	2,4-Heptadienal	-4.1
Volatiles	Octanal	-4.1
Hydrocarbons	Nonane	-4.0
Volatiles	Heptan-2-ol	-4.0
Volatiles	Heptanol	-4.0
Volatiles	Ethyl propanoate	-4.0
Volatiles	Methyl pentanoate	-4.0
Volatiles	Propyl propanoate	-4.0
Volatiles	2-Hexanone	-4.0
Volatiles	Succinic acid	-4.0
Fatty acids	3-monoacylglycerol	-3.9
Hydrocarbons	Octane	-3.9

Volatiles	4-Hexenol	-3.9
Volatiles	cis-2-Hexenol	-3.9
Volatiles	Hexanol	-3.9
Volatiles	trans-2-Hexenol	-3.9
Volatiles	trans-3-Hexenol	-3.9
Volatiles	2-Methyl-2-butenal	-3.9
Volatiles	cis-2-Hexenal	-3.9
Volatiles	cis-3-Hexenal	-3.9
Volatiles	trans-2-Heptenal	-3.9
Volatiles	Methyl butanoate	-3.9
Volatiles	1-Penten-3-one	-3.9
Volatiles	3-Methyl-2-butanone	-3.9
Volatiles	3-Pentanone	-3.9
Volatiles	l-Penten-3-one	-3.9
Fatty acids	2-monoacylglycerol	-3.8
Volatiles	3-Pentanol	-3.8
Volatiles	3-Penten-2-ol	-3.8
Volatiles	Heptanal	-3.8
Volatiles	Hexanal	-3.8
Volatiles	trans-2-Hexenal	-3.8
Volatiles	Butanoic acid	-3.8
Volatiles	Isobutyric acid	-3.8
Volatiles	Pentanoic acid	-3.8
Volatiles	2-Ethylfuran	-3.8
Amino acids	Alanine	-3.7
Amino acids	Serine	-3.7
Hydrocarbons	Heptane	-3.7
Volatiles	1-Penten-3-ol	-3.7
Volatiles	2-Penten-1-ol	-3.7
Volatiles	2-Methylbutanal	-3.7
Volatiles	3-Methylbutanal	-3.7
Hydrocarbons	2-Methylpentane	-3.6
Hydrocarbons	3-Methylpentane	-3.6
Volatiles	2-Methyl-1-butanol	-3.6
Volatiles	2-Methyl-3-butenol	-3.6
Volatiles	3-Methyl-1-butanol	-3.6
Volatiles	Pentanol	-3.6
Volatiles	cis-2-Pentenal	-3.6
Volatiles	Ethyl acetate	-3.6
Volatiles	Pentanal	-3.5
Volatiles	Propanal	-3.5
Volatiles	trans-2-Pentenal	-3.5
Volatiles	2-Butanone	-3.5

Volatiles	Butan-2-one	-3.5
Volatiles	4-Methoxy-2-methyl-2-butanethiol	-3.5
Hydrocarbons	Hexene	-3.4
Volatiles	Oxalic acid	-3.4
Volatiles	3-Methyl-2-butenethiol	-3.4
Hydrocarbons	2-Methylbutane	-3.3
Hydrocarbons	Hexane	-3.3
Volatiles	Propanoic acid	-3.3
Volatiles	Butan-1-ol	-3.2
Volatiles	Butan-2-ol	-3.2
Volatiles	Methyl acetate	-3.1
Volatiles	1-Propanol	-2.8
Volatiles	Acetic acid	-2.8
Volatiles	Diethyl ether	-2.8
Volatiles	Acetaldehyde	-2.4
Volatiles	Ethanol	-2.3
Volatiles	Methanol	-1.9
Pigments	Neoxanthin	-0.9

Table S2. Predicted binding affinities (kcal/mol) of the olive-derived compounds from the OliveNet™ database against the central cavity of the GIRK4^{G151R} structure that was obtained from the final frame of the equilibrated system.

OliveNet™ class	Compound	Binding Affinity (kcal/mol)
Pigments	Chlorophyllide a	-11.5
Phenolics	Luteolin-4'-O-rutinoside	-11.4
Phenolics	Nüzhenide 11-Methyl oleoside	-11.3
Pigments	Chlorophyllide b	-11.3
Phenolics	Isorhoifolin	-11.2
Phenolics	Scolymoside	-11.2
Phenolics	Apigenin-7-O-rutinoside	-11.1
Phenolics	Luteolin-3′,7-O-diglucoside	-11.1
Phenolics	Jaspolyoside	-10.9
Phenolics	Luteolin-6-C-glucoside	-10.9
Phenolics	Isojaspolyoside A	-10.8
Phenolics	Luteolin-7-O-rutinoside	-10.8
Pigments	Chlorophyll a	-10.8
Pigments	Pyropheophytin α	-10.8
Phenolics	Acetoside	-10.7
Phenolics	Cyanidin-3-O-rutinoside	-10.6
Phenolics	Ligstroside derivative 5	-10.6
Phenolics	Luteolin-4’-O-glucoside	-10.6
Phenolics	Nüzhenide oleoside	-10.6
Phenolics	Oleuropein dimer	-10.6
Phenolics	Quercetin 3-O-rutinoside	-10.6
Phenolics	β-Hydroxy verbascoside	-10.6
Phenolics	β-Hydroxy-acetoside	-10.6
Phenolics	Hesperidin	-10.5
Phenolics	Luteolin-7-O-glucoside	-10.5
Phenolics	Apigenin-7-O-glucoside	-10.4
Phenolics	Chrysoeriol-7-O-glucoside	-10.4
Phenolics	Orbanchoside	-10.4
Phenolics	Oxidized verbascoside	-10.4
Phenolics	Quercetin-7-O-glucoside	-10.4
Phenolics	Rutin	-10.4
Phenolics	Suspensaside	-10.4
Phenolics	Verbascoside	-10.4
Phenolics	Hellicoside	-10.3
Phenolics	Isoacteoside	-10.2
Phenolics	Isoverbascoside	-10.2
Phenolics	Ligstroside derivative 4	-10.2
Phenolics	Luteolin-7,4-O-diglucoside	-10.2
Phenolics	4'-O-β-D-Glucosyl-9-O-(6''-deoxysaccharosyl)olivil	-10.1
Phenolics	Oxidized isoverbascoside	-10.1
Pigments	Pheophorbide b	-10.1

Phenolics	Quercetin-3-rhamnoside	-10.0
Phenolics	(+)-1-Acetoxypinoresinol-4'-β-D-glucopyranoside	-9.9
Phenolics	Demethyloleuropein	-9.9
Phenolics	Oleuropein diglucoside	-9.9
Pigments	Pheophorbide a	-9.9
Sterols	Taraxasterol	-9.8
Phenolics	Oleuropein-3'-O-β-D-glucopyranoside	-9.7
Phenolics	Demethylligstroside	-9.6
Phenolics	Jaspolyanoside	-9.6
Phenolics	Luteolin-8-C-glucoside	-9.6
Phenolics	Oleuricine A	-9.6
Phenolics	Quercitrin	-9.6
Sterols	Δ-5,24-Stigmastadienol	-9.6
Phenolics	(+)-1-Acetoxypinoresinol-4'-β-D-glucopyranoside	-9.5
Phenolics	10-Hydroxyoleuropein	-9.5
Phenolics	Ligstroside	-9.5
Phenolics	Wedelosin	-9.5
Sterols	Ergosterol	-9.5
Phenolics	Elenolic acid diglucoside	-9.4
Phenolics	Nüzhenide	-9.4
Sterols	Stigmasterol	-9.4
Sterols	3-epi-lupeol	-9.4
Phenolics	Hydroxypinoresinol	-9.3
Phenolics	Hydroxytyrosol diglucoside	-9.3
Phenolics	Neo-nüzhenide	-9.3
Phenolics	Oleuroside	-9.3
Pigments	Pheophytin α	-9.3
Sterols	24-Ethyl-E-23-dehydrolophenol	-9.3
Sterols	α-amyrin	-9.3
Sterols	Ψ-taraxasterol	-9.3
Phenolics	6'-β-D-Glucopyranosyl oleoside	-9.2
Phenolics	Oleuropein-3"-Methyl ether	-9.2
Pigments	Pheophytin b	-9.2
Sterols	24-Methylene-cholesterol	-9.2
Sterols	Δ-5-Avenasterol	-9.2
Triterpenic acids	Corosolic acid	-9.2
Phenolics	(+)-1-Acetoxypinoresinol-4'-β-D-glucopyranoside-4"-O-methyl ether	-9.1
Phenolics	(+)-Fraxiresinol-1-β-D-glucopyranoside	-9.1
Phenolics	6'-Rhamnopyranosyl oleoside	-9.1
Phenolics	Cyanidin-3-O-glucoside	-9.1
Phenolics	Quercetin-3-O-glucoside	-9.1
Pigments	Chlorophyll b	-9.1
Sterols	Brassicasterol	-9.1
Sterols	Campesterol	-9.1
Sterols	β-sitosterol	-9.1
Sterols	Δ7,24-Ergostadienol	-9.1
Sterols	28-nor-β-amyrin	-9.1

Sterols	Lupenone	-9.1
Triterpenic acids	Pomolic acid	-9.1
Phenolics	(+)-1-Acetoxypinoresinol-4"-O-methyl ether	-9.0
Phenolics	Caffeoyl-6'-secologanoside	-9.0
Phenolics	Comselogoside	-9.0
Phenolics	Oleuropein	-9.0
Phenolics	Oleuroside-10-carboxylic acid	-9.0
Phenolics	Quercetin	-9.0
Phenolics	Vicenin-2	-9.0
Sterols	Δ-5,23-Stigmastadienol	-9.0
Sterols	24-methyl-24(25)-dehydrolophenol	-9.0
Sterols	28-isocitrostadienol	-9.0
Sterols	Methyl 3β-acetoxyolean-12-en-28-oate	-9.0
Triterpenic acids	Ursolic acid	-9.0
Phenolics	Delphinidin-3-O-glucoside	-8.9
Phenolics	Ligstroside derivative 3	-8.9
Phenolics	Methoxyluteolin	-8.9
Phenolics	Oleuropeindial - Lactone (Cannizzaro-like product of oleuropeindial)	-8.9
Phenolics	Syringaresinol	-8.9
Phenolics	Taxifolin	-8.9
Sterols	22,23- Dihydrobrassicasterol	-8.9
Sterols	Δ7,22-Ergostadienol	-8.9
Sterols	24-ethyllophenol	-8.9
Sterols	24-methyl-(E)-23-dehydrolophenol	-8.9
Sterols	(24Z)-24-ethylidene-dihydrolanosterol	-8.9
Sterols	24-methylene-24-dihydrolanosterol	-8.9
Sterols	Germanicol	-8.9
Sterols	Taraxerol	-8.9
Sterols	β-amyrin	-8.9
Phenolics	Chrysoeriol	-8.8
Phenolics	Eriodictyol	-8.8
Phenolics	Lucidumoside C	-8.8
Sterols	4α,14α-Dimethylstigmasta-8,24(24)-dien-3β-ol	-8.8
Sterols	Bacchar-12,21-dien-3β-ol	-8.8
Phenolics	10-Hydroxyoleuropein	-8.7
Phenolics	Delphinidin	-8.7
Phenolics	Luteolin	-8.7
Phenolics	Oleuricine B	-8.7
Sterols	Cholesterol	-8.7
Sterols	28-nor-α-amyrin	-8.7
Sterols	Uvaol	-8.7
Sugars	D-(+)-raffinose	-8.7
Triterpenic acids	3-epi-betulinic acid	-8.7
Triterpenic acids	Betulinic acid	-8.7
Triterpenic acids	Oleanolic acid demethyl	-8.7
Phenolics	1-Acetoxypinoresinol	-8.6
Phenolics	Cyanidin (cation)	-8.6

Phenolics	Demethyloleuropein aglycone	-8.6
Phenolics	Oleoside	-8.6
Phenolics	Pinoresinol	-8.6
Sterols	24-methylene-24-dihydroparkeol	-8.6
Sterols	24-methylene-cycloartenol	-8.6
Sterols	Erythrodiol	-8.6
Phenolics	3-Acetyloxy berchemol	-8.5
Phenolics	Apigenin	-8.5
Phenolics	Diosmetin	-8.5
Phenolics	Esculin	-8.5
Phenolics	Ligstroside aglycone methyl acetal	-8.5
Phenolics	Rosmarinic acid	-8.5
Sterols	Δ-7-Avenasterol	-8.5
Sterols	Δ-7-Stigmastenol	-8.5
Sterols	Citrostadienol	-8.5
Sterols	4,4-dimethyl-5α-stigmast-7-en-3β-ol	-8.5
Sterols	Cyclobranol	-8.5
Triterpenic acids	Urs-2β,3β-dihydroxy-12-en-28-oic acid	-8.5
Phenolics	(+)-1-Acetoxypinoresinol-4-O-methyl ether	-8.4
Phenolics	Hesperitin	-8.4
Phenolics	Verucosin	-8.4
Sterols	24-Methylenelophenol	-8.4
Sterols	Gramisterol	-8.4
Sterols	28-hydroxytaraxerol	-8.4
Sterols	Lupeol	-8.4
Sterols	β-amyrone	-8.4
Sterols	δ-amyrin	-8.4
Phenolics	1-(3'-Methoxy-4'-hydroxy)- phenyl-6,7-dihydroxyisochroman	-8.3
Phenolics	6'-O-[(2E)-2,6-Dimethyl-8-hydroxy- 2-octenoyloxy]-secologanoside	-8.3
Phenolics	Chlorogenic acid	-8.3
Phenolics	Dihydro-oleuropein	-8.3
Phenolics	Ligstroside-3'-O-β-D-glucopyranoside	-8.3
Phenolics	Oleoside-11-Methylester	-8.3
Phenolics	Secologanoside	-8.3
Sterols	Agrostophyllinol	-8.3
Sterols	Methyl 2α,3β-diacetoxyolean-12-en-28-oate	-8.3
Sugars	Mannan	-8.3
Triterpenic acids	Maslinic acid	-8.3
Phenolics	Berchemol	-8.2
Phenolics	Hydroxytyrosil-elenolate	-8.2
Phenolics	Loganin	-8.2
Phenolics	Monoaldehydic form of Oleuropein aglycon	-8.2
Phenolics	Oleuropein aglycone (3,4-DHPEA-EA)	-8.2
Phenolics	Scopolin	-8.2
Sterols	24-methyl-31-nor-9(11)-lanostenol	-8.2
Sterols	24-methylene-31-nor-9(11)-lanostenol	-8.2

Sterols	4,4-dimethyl-5α-stigmasta-7,24Z(241)-dien-3β-ol	-8.2
Sterols	Butyrospermol	-8.2
Sterols	Cyclosadol	-8.2
Sugars	Maltotriose	-8.2
Tocopherols	α-tocopherol	-8.2
Triterpenic acids	Oleanolic acid	-8.2
Phenolics	Elenolic acid glucoside	-8.1
Phenolics	Ligstroside aglycone	-8.1
Phenolics	Secologanin	-8.1
Sterols	Campestanol	-8.1
Sterols	24-Ethylidenelophenol	-8.1
Sterols	24-methylene-24-dihydroparkenol	-8.1
Sterols	3-epi-betulin	-8.1
Sterols	7, 24-tirucalladienol	-8.1
Tocopherols	β-tocopherol	-8.1
Hydrocarbons	Squalene	-8.0
Other	Poly-unsaturated di-galactoside glycerol diester	-8.0
Phenolics	(-)-Olivil	-8.0
Phenolics	3,4-DHPEA-DETA	-8.0
Phenolics	Caffeoylglucose	-8.0
Phenolics	Hydroxytyrosol-4-β-glucoside	-8.0
Phenolics	Ligstroside derivative 1	-8.0
Phenolics	Secologanol	-8.0
Sterols	Cycloeucalenol	-8.0
Sterols	Cycloartenol	-8.0
Phenolics	10-Hydroxy-10-methyl oleuropein aglycone	-7.9
Phenolics	Monoaldehydic form of Ligstroside aglycon	-7.9
Phenolics	Oleuropeindial (enol form)	-7.9
Sterols	Dammaradienol	-7.9
Sterols	Parkeol	-7.9
Tocopherols	γ-tocopherol	-7.9
Phenolics	10-Hydroxy oleuropein aglycone	-7.8
Phenolics	Loganic acid	-7.8
Phenolics	10-Hydroxy oleuropein aglycone decarboxymethyl	-7.7
Phenolics	7-Deoxyloganic acid	-7.7
Phenolics	Oleoside dimethylester	-7.7
Sterols	Tirucallol	-7.7
Sugars	Galactinol	-7.7
Phenolics	(+)-Cycloolivil	-7.6
Phenolics	Cornoside	-7.6
Phenolics	Demethyloleuropein aglycone dialdehyde	-7.6
Phenolics	Hydroxytyrosol rhamnoside	-7.6
Phenolics	Hydroxytyrosol-1′-β-glucoside	-7.6
Phenolics	Ligstroside derivative 2	-7.6

Phenolics	Oleuropeindial (keto form)	-7.5
Phenolics	Salidroside	-7.5
Sterols	Clerosterol	-7.5
Sterols	Stigmastanol	-7.5
Tocopherols	δ-tocopherol	-7.5
Aliphatic and aromatic	Geranylgeraniol	-7.4
Phenolics	DHPEA-DEDA	-7.4
Phenolics	Hydroxytyrosol-3-β-glucoside	-7.4
Phenolics	3,4-DHPEA-EDA (Oleuropein-aglycone di-aldehyde)	-7.3
Phenolics	Caftaric acid	-7.3
Phenolics	DHPEA-DEDA (acetal)	-7.3
Phenolics	Hemiacetal of dialdehydic oleuropein aglycone decarboxymethyl	-7.3
Phenolics	Oleuropeindial (Cannizzaro-like product of oleuropeindial)	-7.3
Phenolics	Secologanic acid	-7.3
Sugars	α-Cellulose	-7.3
Hydrocarbons	Geranylgeranyl oleate C18:1	-7.2
Phenolics	Oleocanthal (Dialdehydic form of decarboxymethyl Ligstroside aglycon)	-7.2
Sterols	Obtusifoliol	-7.2
Sugars	D-(+)-lactose	-7.2
Phenolics	1-Phenyl-6,7-dihydroxyisochroman	-7.1
Phenolics	demethyloleuropein aglycone (enol form)	-7.1
Phenolics	Hemiacetal of dialdehydic ligstroside aglycone decarboxymethyl	-7.1
Hydrocarbons	Alloaromadendrene	-7.0
Hydrocarbons	Geranylgeranyl C20:0	-7.0
Phenolics	Hydroxytyrosol acyclodihydroelenolate	-7.0
Sugars	D-(+)-sucrose	-7.0
Hydrocarbons	γ-Muurolene	-6.9

Phenolics	Decarboxymethyl ligstroside aglycone	-6.9
Phenolics	Methyl malate-hydroxytyrosol ester	-6.9
Phospholipids	Phosphatidylinositol	-6.9
Phenolics	1-oleyltyrosol	-6.8
Hydrocarbons	γ-curcumene	-6.7
Hydrocarbons	β- Sesquiphellandrene	-6.7
Hydrocarbons	Geranylgeranyl oleate C18:0	-6.7
Phenolics	Demethyl elenolic acid	-6.7
Phenolics	p-HPEA-EDA	-6.7
Phospholipids	Lysophosphatidylethanolamine	-6.7
Hydrocarbons	Calarene	-6.6
Hydrocarbons	Eremophyllene	-6.6
Hydrocarbons	β-acoradiene	-6.6
Hydrocarbons	β-cubebene	-6.6
Hydrocarbons	β-elemene	-6.6
Hydrocarbons	Geranylgeranyl C20:1	-6.6
Hydrocarbons	Phytyl oleate C18:1	-6.6
Phenolics	Esculetin	-6.6
Hydrocarbons	(Z)2,(E)4,(E)6-Allofarnesene	-6.5
Hydrocarbons	(E)-caryophyllene	-6.5
Hydrocarbons	Geranylgeranyl C22:0	-6.5
Hydrocarbons	Geranylgeranyl C24:0	-6.5
Fatty acids	Linolenic	-6.4
Hydrocarbons	α-copaene	-6.4
Hydrocarbons	Wax ester 46:0 (16:0-30:0)	-6.4
Phenolics	D-(+)-Erythro-1-(4-hydroxy-3-methoxy)- 214 - phenyl-1,2,3-propantriol	-6.4
Phenolics	Hydroxycaffeic acid	-6.4
Phenolics	Scopoletin	-6.4
Phenolics	Tyrosol acetate	-6.4
Hydrocarbons	(E)-β-farnesene	-6.3
Hydrocarbons	Drima-7,9(11)-diene	-6.3
Hydrocarbons	α-Selinene	-6.3
Hydrocarbons	α-Zingiberene	-6.3
Hydrocarbons	β-Curcumene	-6.3
Hydrocarbons	Wax ester 40:0 (14:0-26:0)	-6.3
Hydrocarbons	Wax ester 40:0 (16:0-24:0)	-6.3
Hydrocarbons	Wax ester 42:0 (18:0-24:0)	-6.3
Hydrocarbons	Wax ester 44:1 (20:1-24:0)	-6.3
Phenolics	Caffeic acid	-6.3

Phenolics	Deoxyloganic acid lauryl ester	-6.3
Phenolics	Ferulic acid	-6.3
Volatiles	cis-3-Hexenyl acetate	-6.3
Volatiles	trans-β-Damascenone	-6.3
Fatty acids	Ethyl linoleate	-6.2
Fatty acids	Linoelaidic	-6.2
Hydrocarbons	Phytyl C20:1	-6.2
Hydrocarbons	Phytyl oleate C18:0	-6.2
Hydrocarbons	Wax ester 38:0 (14:0-24:0)	-6.2
Hydrocarbons	Wax ester 42:1 (16:1-26:0)	-6.2
Hydrocarbons	Wax ester 46:0 (18:0-28:0)	-6.2
Other	3-[1-(hydroxymethyl)-(E)-1-propenyl] glutaric acid	-6.2
Phenolics	2,3-dihydrocaffeic acid	-6.2
Phenolics	Sinapic acid	-6.2
Phospholipids	Lysophosphatidic acid	-6.2
Amino acids	Tyrosine	-6.1
Fatty acids	Methyl linoleate	-6.1
Hydrocarbons	Hexacosane	-6.1
Hydrocarbons	α-trans-bergamotene	-6.1
Hydrocarbons	Phytyl C22:0	-6.1
Hydrocarbons	Wax ester 38:0 (12:0-26:0)	-6.1
Hydrocarbons	Wax ester 42:0 (14:0-28:0)	-6.1
Hydrocarbons	Wax ester 42:0 (20:0-22:0)	-6.1
Hydrocarbons	Wax ester 44:0 (16:0-28:0)	-6.1
Hydrocarbons	Wax ester 44:0 (18:0-26:0)	-6.1
Hydrocarbons	Wax ester 46:0 (20:0-26:0)	-6.1
Hydrocarbons	Wax ester 46:0 (24:0-22:0)	-6.1
Hydrocarbons	Wax ester 46:1 (18:1-28:0)	-6.1
Phenolics	3,4-Dihydroxyphenylglycol	-6.1
Phenolics	4-Hydroxy-3-methoxy-phenylacetic acid	-6.1
Phenolics	4-O-methyl-D-glucuronic acid	-6.1
Phenolics	Elenolic acid	-6.1
Phenolics	Hydroxytyrosol acetate	-6.1
Phenolics	*m*-Coumaric acid	-6.1
Sugars	Pectin	-6.1
Fatty acids	Eicosenoic	-6.0
Fatty acids	Elaidic	-6.0
Fatty acids	Oleic	-6.0
Fatty acids	Trans-palmitoleic	-6.0
Hydrocarbons	Cyclosativene	-6.0

Hydrocarbons	Methyl oleate	-6.0
Hydrocarbons	Phytyl C24:0	-6.0
Hydrocarbons	Wax ester 40:1 (16:1-24:0)	-6.0
Hydrocarbons	Wax ester 42:0 (16:0-26:0)	-6.0
Hydrocarbons	Wax ester 44:1 (16:0-28:0)	-6.0
Phenolics	Homovanillic acid	-6.0
Phenolics	Isoeugenol	-6.0
Phenolics	*p*-Coumaric acid	-6.0
Sugars	D-glucuronic acid	-6.0
Aliphatic and aromatic	Phytol	-5.9
Aliphatic and aromatic	Eicosanol	-5.9
Aliphatic and aromatic	Tetracosanol	-5.9
Fatty acids	11-cis-vaccenic	-5.9
Fatty acids	Cis-Heptadecenoic	-5.9
Fatty acids	Gadoleic	-5.9
Hydrocarbons	9-Heptacosene	-5.9
Hydrocarbons	9-Pentacosene	-5.9
Hydrocarbons	(E)2,(Z)4,(E)6-Allofarnesene	-5.9
Hydrocarbons	Ethyl oleate	-5.9
Hydrocarbons	Wax ester 38:0 (18:0-20:0)	-5.9
Hydrocarbons	Wax ester 40:0 (18:0-22:0)	-5.9
Hydrocarbons	Wax ester 40:0 (20:0-20:0)	-5.9
Hydrocarbons	Wax ester 42:0 (24:0-18:0)	-5.9
Hydrocarbons	Wax ester 46:0 (22:0-24:0)	-5.9
Phenolics	2,6-Dimethoxybenzoic acid	-5.9
Phenolics	3,4-Dihydroxyphenylacetic acid	-5.9
Phenolics	Homovanillyl alcohol	-5.9
Phenolics	*o*-Coumaric acid	-5.9
Aliphatic and aromatic	Pentacosanol	-5.8
Amino acids	Phenylalanine	-5.8
Fatty acids	Methyl palmitate	-5.8
Fatty acids	Linoleic	-5.8
Fatty acids	Petroselinic	-5.8
Fatty acids	Stearic	-5.8
Hydrocarbons	Longicyclene	-5.8
Hydrocarbons	Methyl stearate	-5.8
Hydrocarbons	Wax ester 42:1 (18:1-24:0)	-5.8
Hydrocarbons	Wax ester 46:0 (14:0-32:0)	-5.8
Phenolics	Dihydro-*p*-coumaric acid	-5.8
Phenolics	Elenolic acid methylester	-5.8

Phenolics	Gallic acid	-5.8
Phenolics	Hydroxytyrosol	-5.8
Phenolics	Phloretic acid	-5.8
Phenolics	Protocatechuic acid	-5.8
Sugars	Sedoheptulose	-5.8
Volatiles	Terpineol	-5.8
Volatiles	Ethyl cinnamate	-5.8
Aliphatic and aromatic	Octacosanol	-5.7
Aliphatic and aromatic	Tricosanol	-5.7
Fatty acids	Methyl heptadecanoate	-5.7
Fatty acids	Methyl oleate	-5.7
Fatty acids	Methyl stearate	-5.7
Fatty acids	Ethyl oleate	-5.7
Fatty acids	Margaric acid	-5.7
Fatty acids	Palmitic	-5.7
Fatty acids	Palmitoleic	-5.7
Hydrocarbons	Heptadecane	-5.7
Hydrocarbons	9-Tetracosene	-5.7
Hydrocarbons	Wax ester 44:0 (22:0-22:0)	-5.7
Hydrocarbons	Wax ester 44:1 (18:1-26:0)	-5.7
Phenolics	2,5-Dihydroxyphenylacetic acid	-5.7
Phenolics	3,4-Dimethoxybenzoic acid	-5.7
Phenolics	Homovanillin	-5.7
Phenolics	Quinic acid	-5.7
Phenolics	Shikimic acid	-5.7
Phenolics	Syringic acid	-5.7
Phenolics	Vanillic acid	-5.7
Sugars	Galacturonan	-5.7
Volatiles	2-Ethylphenyl acetate	-5.7
Volatiles	Phenethyl acetate	-5.7
Aliphatic and aromatic	Heptacosanol	-5.6
Aliphatic and aromatic	Hexacosanol	-5.6
Fatty acids	Ethyl palmitate	-5.6
Fatty acids	Ethyl stearate	-5.6
Fatty acids	Lignoceric	-5.6
Fatty acids	Myristic	-5.6

Hydrocarbons	Nonadecane	-5.6
Hydrocarbons	Triacontane	-5.6
Hydrocarbons	Tritriacontane	-5.6
Hydrocarbons	9-Hexacosene	-5.6
Hydrocarbons	9-Tricosene	-5.6
Hydrocarbons	δ-cadinene	-5.6
Hydrocarbons	Phytyl C20:0	-5.6
Phenolics	2,4 dihydroxybenzoic acid	-5.6
Phenolics	Cinnamic acid	-5.6
Phenolics	Elenolic acid dialdehyde	-5.6
Phenolics	Gentisic acid	-5.6
Phenolics	Homoveratric acid	-5.6
Phenolics	p-Hydroxyphenylacetic acid	-5.6
Sugars	D-(+)-mannose	-5.6
Sugars	D-(+)-galacturonic acid	-5.6
Volatiles	Gluconic acid	-5.6
Aliphatic and aromatic	Docosanol	-5.5
Hydrocarbons	Icosane	-5.5
Hydrocarbons	Tricosane	-5.5
Hydrocarbons	8-Heptadecene	-5.5
Hydrocarbons	Wax ester 38:0 (20:0-18:0)	-5.5
Hydrocarbons	Wax ester 40:1 (18:1-22:0)	-5.5
Other	3-(1-Hydroxymethyl-1-propenyl)pentanedioic acid	-5.5
Phenolics	2,6-Dihydroxybenzoic acid	-5.5
Phenolics	2-Methoxy-4-vinylphenol	-5.5
Phenolics	3,4,5-Trimethoxybenzoic acid	-5.5
Phenolics	4-Ethylguaiacol	-5.5
Phenolics	4-Vinylguaiacol	-5.5
Phenolics	DEDA acetal	-5.5
Phenolics	Syringaldehyde	-5.5
Phenolics	Tyrosol	-5.5
Phospholipids	Phosphatidylcholine	-5.5
Phospholipids	Phosphatidylglycerol	-5.5
Sugars	D-(-)-fructose	-5.5
Sugars	D-(-)-galactose	-5.5
Sugars	myo-inositol	-5.5
Amino acids	Arginine	-5.4
Fatty acids	Erucic	-5.4
Fatty acids	Lauric	-5.4

Hydrocarbons	Docosane	-5.4
Hydrocarbons	Dotriacontane	-5.4
Hydrocarbons	Heneicosane	-5.4
Hydrocarbons	Hentriacontane	-5.4
Hydrocarbons	Hexadecane	-5.4
Hydrocarbons	Octacosane	-5.4
Hydrocarbons	Octadecane	-5.4
Hydrocarbons	Pentacosane	-5.4
Hydrocarbons	Tetracosane	-5.4
Hydrocarbons	9-Docosene	-5.4
Hydrocarbons	Methyl palmitate	-5.4
Hydrocarbons	Wax ester 38:0 (16:0-22:0)	-5.4
Other	3-[1-(formyl)-(E)-1-propenyl] glutaric acid	-5.4
Other	Halleridone	-5.4
Phenolics	4-hydroxybenzoic acid	-5.4
Sugars	1,6-anhydro-β-D-glucose	-5.4
Sugars	D-(+)-glucose	-5.4
Sugars	D-(+)-chiro-inositol	-5.4
Sugars	D-Fucose	-5.4
Volatiles	Lavendulol	-5.4
Volatiles	Vanillin	-5.4
Volatiles	3,4-methyl-3-pentenyl furan	-5.4
Volatiles	2-Ethyl-5-hexylthiophene	-5.4
Fatty acids	Arachidic	-5.3
Hydrocarbons	Heptacosane	-5.3
Hydrocarbons	Nonacosane	-5.3
Hydrocarbons	Pentadecane	-5.3
Hydrocarbons	(Z)-β-farnesene	-5.3
Phenolics	Dialdehydic elenolic ester decarboxymethyl	-5.3
Phospholipids	Phosphatidylethanolamine	-5.3
Sugars	L-rhamnose	-5.3
Volatiles	Ethyl cyclohexylcarboxylate	-5.3
Volatiles	Acetophenone	-5.3
Volatiles	Citric acid	-5.3
Hydrocarbons	Tetratriacontane	-5.2
Hydrocarbons	Eremophilone	-5.2
Phenolics	4-Ethylphenol	-5.2
Phenolics	4-Methylcatechol	-5.2
Phenolics	DEDA	-5.2
Phenolics	Dialdehydic elenolic acid decarboxymethl	-5.2
Sugars	D-Mannitol	-5.2

Sugars	L-Fucose	-5.2
Volatiles	trans-4,5-Epoxy-trans-2-decenal	-5.2
Volatiles	Benzyl acetate	-5.2
Hydrocarbons	Tridecane	-5.1
Phenolics	4-Vinylphenol	-5.1
Sterols	Linalool	-5.1
Volatiles	2-Phenylethanol	-5.1
Volatiles	1,8-Cineole	-5.1
Volatiles	3-Methyl-2-pentylfuran	-5.1
Amino acids	Glutamine	-5.0
Phenolics	4-Hydroxybenzaldehyde	-5.0
Sugars	D-(-)-arabinose	-5.0
Volatiles	2,4-Decadienal	-5.0
Fatty acids	1,3-diacylglycerol	-4.9
Fatty acids	2,3-diacylglycerol	-4.9
Hydrocarbons	Tetradecane	-4.9
Hydrocarbons	6,10-dimethyl-1-undecene	-4.9
Other	1,5-anhydroxylitol	-4.9
Phenolics	Guaiacol	-4.9
Phenolics	o-cresol	-4.9
Phenolics	p-cresol	-4.9
Sugars	D-(+)-xylose	-4.9
Volatiles	6-Methyl-5-hepten-3-ol	-4.9
Volatiles	Phenylacetaldehyde	-4.9
Volatiles	trans,trans-2,4-Decadienal	-4.9
Volatiles	2-Methylpropyl butanoate	-4.9
Volatiles	cis-1,5-Octadien-3-one	-4.9
Amino acids	Aspargine	-4.8
Amino acids	Aspartic acid	-4.8
Fatty acids	1,2-diacylglycerol	-4.8
Phenolics	m-cresol	-4.8
Phospholipids	Phosphatidic acid	-4.8
Volatiles	Benzyl alcohol	-4.8
Volatiles	Benzaldehyde	-4.8
Volatiles	trans,cis-2,4-Decadienal	-4.8
Volatiles	Ethyl 2-methylbutanoate	-4.8
Volatiles	Octyl acetate	-4.8
Volatiles	Propyl 2-methylpropanoate	-4.8
Volatiles	6-Methyl-5-hepten-2-one	-4.8
Volatiles	Malic acid	-4.8
Amino acids	Glutamic acid	-4.7
Fatty acids	1-monoacylglycerol	-4.7
Hydrocarbons	Styrene	-4.7

Sugars	L-(-)-arabitol	-4.7
Volatiles	Decanal	-4.7
Volatiles	trans,trans-2,4-Nonadienal	-4.7
Volatiles	Ethyl 3-methylbutanoate	-4.7
Volatiles	Ethyl octanoate	-4.7
Volatiles	3-Octanone	-4.7
Volatiles	l-Octen-3-one	-4.7
Volatiles	2,5-Diethylthiophene	-4.7
Amino acids	Isoleucine	-4.6
Fatty acids	2-monoacylglycerol	-4.6
Hydrocarbons	6,10-Dimethyl-l-undecane	-4.6
Phenolics	Catechol	-4.6
Volatiles	2-Octanol	-4.6
Volatiles	3-Hexenyl acetate	-4.6
Volatiles	Ethyl hexanoate	-4.6
Volatiles	Hexyl acetate	-4.6
Volatiles	Isopentyl acetate	-4.6
Volatiles	Methyl octanoate	-4.6
Volatiles	3-Propylfuran	-4.6
Amino acids	Leucine	-4.5
Hydrocarbons	Dodecane	-4.5
Hydrocarbons	Methyl benzene	-4.5
Sugars	Adonitol	-4.5
Sugars	Xylitol	-4.5
Volatiles	3-Octenol	-4.5
Volatiles	Nonanal	-4.5
Volatiles	2-Hexenyl acetate	-4.5
Volatiles	2-Methylbutyl acetate	-4.5
Volatiles	Ethyl isobutyrate	-4.5
Volatiles	Ethyl-2-methypropanoate	-4.5
Volatiles	Methyl 2-methylbutanoate	-4.5
Volatiles	Methyl 3-methylbutanoate	-4.5
Volatiles	Methyl heptanoate	-4.5
Volatiles	Methyl hexanoate	-4.5
Volatiles	Propyl butanoate	-4.5
Volatiles	2-Nonanone	-4.5
Amino acids	Valine	-4.4
Hydrocarbons	Tridecane	-4.4
Volatiles	Decanol	-4.4
Volatiles	Nonanol	-4.4
Volatiles	Octanol	-4.4
Volatiles	2,4-Heptadienal	-4.4
Volatiles	cis-2-Nonenal	-4.4

Volatiles	trans-2-Decenal	-4.4
Volatiles	trans-2-Nonenal	-4.4
Volatiles	Octanoic acid	-4.4
Volatiles	Propyl propanoate	-4.4
Volatiles	2-Propylfuran	-4.4
Volatiles	2-Octanone	-4.4
Volatiles	4-Methyl-2-pentanone	-4.4
Volatiles	Octan-2-one	-4.4
Volatiles	3-Isopropenylthiophene	-4.4
Volatiles	2-Heptanol	-4.3
Volatiles	cis-2-Hexenol	-4.3
Volatiles	Heptan-2-ol	-4.3
Volatiles	Heptanol	-4.3
Volatiles	3-Hexanal	-4.3
Volatiles	Octanal	-4.3
Volatiles	trans-2-Octenal	-4.3
Volatiles	Heptanoic acid	-4.3
Volatiles	Ethyl butanoate	-4.3
Volatiles	Methyl pentanoate	-4.3
Fatty acids	3-monoacylglycerol	-4.2
Hydrocarbons	Nonane	-4.2
Phenolics	Phenol	-4.2
Volatiles	4-Hexenol	-4.2
Volatiles	cis-3-Hexenol	-4.2
Volatiles	trans-2-Hexenol	-4.2
Volatiles	trans-3-Hexenol	-4.2
Volatiles	2,4 Hexadienal	-4.2
Volatiles	cis-2-Heptenal	-4.2
Volatiles	trans-2-Heptenal	-4.2
Volatiles	3-Methyl-butanoic acid	-4.2
Volatiles	3-Methylbutyric acid	-4.2
Volatiles	Hexanoic acid	-4.2
Volatiles	2-Heptanone	-4.2
Volatiles	Heptan-2-one	-4.2
Amino acids	Serine	-4.1
Hydrocarbons	Octane	-4.1
Volatiles	1-Penten-3-ol	-4.1
Volatiles	Ethyl propanoate	-4.1
Volatiles	1-Penten-3-one	-4.1
Volatiles	2-Hexanone	-4.1
Volatiles	3-Pentanone	-4.1
Volatiles	l-Penten-3-one	-4.1
Volatiles	Succinic acid	-4.1

Volatiles	3-Pentanol	-4.0
Volatiles	Hexanol	-4.0
Volatiles	2-Methyl-2-butenal	-4.0
Volatiles	cis-2-Hexenal	-4.0
Volatiles	Heptanal	-4.0
Volatiles	trans-2-Hexenal	-4.0
Volatiles	Butyl acetate	-4.0
Volatiles	Methyl butanoate	-4.0
Volatiles	2-Ethylfuran	-4.0
Volatiles	3-Methyl-2-butanone	-4.0
Hydrocarbons	Heptane	-3.9
Volatiles	2-Penten-1-ol	-3.9
Volatiles	3-Penten-2-ol	-3.9
Volatiles	cis-3-Hexenal	-3.9
Volatiles	Hexanal	-3.9
Volatiles	Pentanoic acid	-3.9
Volatiles	2-Methyl-1-butanol	-3.8
Volatiles	3-Methyl-1-butanol	-3.8
Volatiles	2-Methylbutanal	-3.8
Volatiles	3-Methylbutanal	-3.8
Volatiles	Isobutyric acid	-3.8
Amino acids	Alanine	-3.7
Hydrocarbons	2-Methylpentane	-3.7
Hydrocarbons	3-Methylpentane	-3.7
Hydrocarbons	Hexene	-3.7
Volatiles	2-Methyl-3-butenol	-3.7
Volatiles	Pentanol	-3.7
Volatiles	cis-2-Pentenal	-3.7
Volatiles	trans-2-Pentenal	-3.7
Volatiles	Butanoic acid	-3.7
Volatiles	4-Methoxy-2-methyl-2-butanethiol	-3.7
Hydrocarbons	Hexane	-3.6
Volatiles	Pentanal	-3.6
Volatiles	Propanal	-3.6
Volatiles	Ethyl acetate	-3.6
Volatiles	2-Butanone	-3.6
Volatiles	Butan-2-one	-3.6
Volatiles	Butan-2-ol	-3.5
Volatiles	Oxalic acid	-3.5
Volatiles	3-Methyl-2-butenethiol	-3.5
Volatiles	Butan-1-ol	-3.4
Volatiles	Propanoic acid	-3.4

Hydrocarbons	2-Methylbutane	-3.2
Volatiles	Methyl acetate	-3.2
Volatiles	Diethyl ether	-3.0
Volatiles	1-Propanol	-2.9
Volatiles	Acetic acid	-2.8
Volatiles	Ethanol	-2.5
Volatiles	Acetaldehyde	-2.4
Volatiles	Methanol	-2.3
Pigments	Neoxanthin	-1.1

Table S3. Potential ligand-binding sites in the representative protein structures of the GIRK4^{WT} and GIRK4^{G151R} channels were predicted using the PrankWeb server, with pocket 2 consisting of central cavity residues. The ligandability score of the second putative binding site is provided.

	**GIRK4^{WT}**	**GIRK4^{G151R}**
**Cluster 1**	Pocket 2: 22.18	Pocket 2: 38.94
	Chain A: 146, 147, 148, 149, 150, 168, 169, 172, 173, 176, 179<br>Chain B: 101, 146, 147, 148, 149, 171, 172, 176, 179, 182, 183, 186, 97<br>Chain C: 146, 147, 148, 149, 168, 172, 176, 147, 148, 149, 150	Chain A: 146, 147, 148, 149, 168, 169, 172, 173, 176, 179<br>Chain B: 101, 146, 147, 148, 149, 151, 172, 175, 176, 179, 182, 97<br>Chain C: 146, 147, 148, 149, 150, 151, 172, 175, 176, 179, 182, 183, 97<br>Chain D: 145, 146, 147, 148, 149, 150, 151, 168, 169, 172, 176, 179
**Cluster 2**	Pocket 2: 41.59	Pocket 2: 142.83
	Chain A: 101, 108, 146, 147, 148, 149, 168, 171, 172, 175, 176, 179, 183, 97<br>Chain B: 101, 146, 147, 148, 149, 172, 175, 176, 179, 97<br>Chain C: 141, 144, 145, 146, 147, 148, 149, 150, 152, 154, 155, 172, 176, 179<br>Chain D: 101, 142, 146, 147, 148, 149, 156, 157, 168, 171, 172, 175, 176, 179, 183, 97	Chain A: 146, 147, 148, 149, 150, 151, 152, 153, 168, 169, 172, 176, 179, 183, 184, 187, 188, 191, 192, 193, 312, 314<br>Chain B: 101, 147, 148, 149, 151, 152, 153, 156, 172, 173, 175, 176, 179, 180, 182, 183, 184, 187, 188, 191, 192, 194, 311, 312, 314, 72, 75, 76, 78, 79, 80, 82, 83, 97<br>Chain C: 100, 101, 146, 147, 148, 149, 150, 151, 152, 168, 172, 174, 175, 176, 178, 179, 180, 182, 183, 184, 187, 188, 191, 193, 312, 314, 78, 96, 97<br>Chain D: 101, 142, 145, 146, 147, 148, 149, 151, 152, 153, 154, 155, 157, 168, 169, 172, 173, 176, 179, 180, 183, 184, 187, 188, 190, 191, 192, 193, 195, 196, 225, 227, 309, 310, 311, 312, 314, 78, 79, 97
**Cluster 3**	Pocket 2: 49.79	Pocket 2: 44.49
	Chain A: 101, 104, 142, 145, 146, 147, 148, 149, 153, 155, 156, 157, 159, 165, 168, 169, 171, 172, 173, 175, 176, 179, 97	Chain A: 146, 147, 148, 149, 150, 151, 168, 169, 172, 176, 179

	Chain B: 101, 141, 144, 146, 147, 148, 149, 150, 152, 154, 155, 171, 172, 175, 176, 179, 97<br>Chain C: 146, 147, 148, 149, 171, 172, 175, 176, 179, 180, 183, 97<br>Chain D: 146, 147, 148, 149, 171, 172, 175, 176, 179	Chain B: 100, 101, 104, 147, 148, 149, 151, 171, 172, 174, 175, 176, 178, 179, 183, 97<br>Chain C: 101, 147, 148, 149, 150, 151, 175, 176, 179, 182, 183, 97<br>Chain D: 145, 146, 147, 148, 149, 151, 168, 169, 172, 176, 179

Table S4. Predicted binding affinities of the olive-derived compounds against the representative protein structure for cluster 1 of the GIRK4^{WT} channel.

Compound	Binding Affinity (kcal/mol)	OliveNet^{TM} Class
Geranylgeraniol	-6.2	Aliphatic and aromatic
Phytol	-5.7	Aliphatic and aromatic
Tetracosanol	-5.1	Aliphatic and aromatic
Octacosanol	-5.1	Aliphatic and aromatic
Docosanol	-5.0	Aliphatic and aromatic
Hexacosanol	-4.9	Aliphatic and aromatic
Eicosanol	-4.8	Aliphatic and aromatic
Pentacosanol	-4.8	Aliphatic and aromatic
Tricosanol	-4.6	Aliphatic and aromatic
Heptacosanol	-4.4	Aliphatic and aromatic
Tyrosine	-6.3	Amino acids
Arginine	-5.8	Amino acids
Phenylalanine	-5.6	Amino acids
Asparagine	-4.8	Amino acids
Glutamine	-4.8	Amino acids
Glutamic Acid	-4.7	Amino acids
Aspartic Acid	-4.5	Amino acids
Leucine	-4.5	Amino acids
Isoleucine	-4.4	Amino acids
Valine	-4.1	Amino acids
Serine	-4.0	Amino acids
Alanine	-3.6	Amino acids
Trans-palmitoleic	-5.3	Fatty acids
Lignoceric	-5.3	Fatty acids
Methyl heptadecanoate	-5.2	Fatty acids
Linoelaidic	-5.2	Fatty acids
Linoleic	-5.2	Fatty acids
Gadoleic	-5.2	Fatty acids
Ethyl linoleate	-5.1	Fatty acids
11-cis-vaccenic	-5.1	Fatty acids
Petroselinic	-5.1	Fatty acids
Linolenic	-5.1	Fatty acids
Ethyl oleate	-5.0	Fatty acids
Methyl oleate	-5.0	Fatty acids
Elaidic	-5.0	Fatty acids
Eicosenoic	-5.0	Fatty acids
Oleic	-4.9	Fatty acids
Behenic	-4.9	Fatty acids

Methyl linoleate	-4.8	Fatty acids
Methyl stearate	-4.8	Fatty acids
Stearic	-4.8	Fatty acids
Arachidic	-4.8	Fatty acids
Erucic	-4.8	Fatty acids
Lauric	-4.8	Fatty acids
2,3-diacylglycerol	-4.7	Fatty acids
1,3-diacylglycerol	-4.7	Fatty acids
Ethyl palmitate	-4.7	Fatty acids
Ethyl stearate	-4.7	Fatty acids
Methyl palmitate	-4.7	Fatty acids
Palmitic	-4.7	Fatty acids
Palmitoleic	-4.7	Fatty acids
Cis-10-Heptadecenoic	-4.7	Fatty acids
1,2-diacylglycerol	-4.6	Fatty acids
Margaric acid	-4.6	Fatty acids
Myristic	-4.5	Fatty acids
1-monoacylglycerol	-4.3	Fatty acids
3-monoacylglycerol	-4.0	Fatty acids
2-monoacylglycerol	-3.8	Fatty acids
Cyclosativene	-6.3	Hydrocarbons
Drima-7,9(11)-diene	-6.2	Hydrocarbons
β- Sesquiphellandrene	-6.2	Hydrocarbons
Alloaromadendrene	-6.1	Hydrocarbons
(Z)2,(E)4,(E)6-Allofarnesene	-6.0	Hydrocarbons
(E)2,(Z)4,(E)6-Allofarnesene	-6.0	Hydrocarbons
β-Curcumene	-5.9	Hydrocarbons
Eremophilone	-5.8	Hydrocarbons
α-copaene	-5.8	Hydrocarbons
α-Zingiberene	-5.8	Hydrocarbons
β-cubebene	-5.7	Hydrocarbons
β-elemene	-5.7	Hydrocarbons
(E)-caryophyllene	-5.7	Hydrocarbons
γ-Muurolene	-5.7	Hydrocarbons
Eremophyllene	-5.7	Hydrocarbons
α-Selinene	-5.7	Hydrocarbons
γ-curcumene	-5.6	Hydrocarbons
Calarene	-5.6	Hydrocarbons
Geranylgeranyl C20:1	-5.6	Hydrocarbons
Longicyclene	-5.5	Hydrocarbons
δ-cadinene	-5.5	Hydrocarbons
Phytyl oleate C18:0	-5.4	Hydrocarbons
β-acoradiene	-5.3	Hydrocarbons

Wax ester 44:0 (18:0-26:0)	-5.3	Hydrocarbons
Squalene	-5.2	Hydrocarbons
(Z)-β-farnesene	-5.2	Hydrocarbons
(E)-β-farnesene	-5.1	Hydrocarbons
Geranylgeranyl oleate C18:0	-5.1	Hydrocarbons
Phytyl C24:0	-5.1	Hydrocarbons
Geranylgeranyl C24:0	-5.1	Hydrocarbons
Geranylgeranyl oleate C18:1	-5.0	Hydrocarbons
Phytyl C20:1	-5.0	Hydrocarbons
Wax ester 42:0 (20:0-22:0)	-5.0	Hydrocarbons
Wax ester 46:0 (22:0-24:0)	-5.0	Hydrocarbons
Tricosane	-4.9	Hydrocarbons
9-tricosene	-4.9	Hydrocarbons
α-trans-bergamotene	-4.9	Hydrocarbons
Methyl oleate	-4.9	Hydrocarbons
Methyl stearate	-4.9	Hydrocarbons
Ethyl oleate	-4.9	Hydrocarbons
Geranylgeranyl C20:0	-4.9	Hydrocarbons
Geranylgeranyl C22:0	-4.9	Hydrocarbons
Wax ester 40:0 (14:0-26:0)	-4.9	Hydrocarbons
Wax ester 40:0 (20:0-20:0)	-4.9	Hydrocarbons
Wax ester 44:0 (16:0-28:0)	-4.9	Hydrocarbons
Wax ester 44:1 (20:1-24:0)	-4.9	Hydrocarbons
Wax ester 46:0 (20:0-26:0)	-4.9	Hydrocarbons
Heneicosane	-4.8	Hydrocarbons
Hexacpsane	-4.8	Hydrocarbons
9-docosene	-4.8	Hydrocarbons
6,10-dimethyl-1-undecane	-4.8	Hydrocarbons
Phytyl C22:0	-4.8	Hydrocarbons
Wax ester 40:0 (18:0-22:0)	-4.8	Hydrocarbons
9-pentacosene	-4.7	Hydrocarbons
Wax ester 38:0 (12:0-26:0)	-4.7	Hydrocarbons
Wax ester 38:0 (14:0-24:0)	-4.7	Hydrocarbons
Wax ester 42:0 (16:0-26:0)	-4.7	Hydrocarbons
Wax ester 44:0 (22:0-22:0)	-4.7	Hydrocarbons
Wax ester 46:0 (16:0-30:0)	-4.7	Hydrocarbons
Wax ester 46:0 (24:0-22:0)	-4.7	Hydrocarbons
Heptadecane	-4.6	Hydrocarbons
Icosane	-4.6	Hydrocarbons
Tetracosane	-4.6	Hydrocarbons
Nonacosane	-4.6	Hydrocarbons
Styrene	-4.6	Hydrocarbons
8-heptdecene	-4.6	Hydrocarbons

9-hexacosene	-4.6	Hydrocarbons
Methyl palmitate	-4.6	Hydrocarbons
Phytyl C20:0	-4.6	Hydrocarbons
Wax ester 42:1 (18:1-24:0)	-4.6	Hydrocarbons
Tridecane	-4.5	Hydrocarbons
Tetradecane	-4.5	Hydrocarbons
Pentadecane	-4.5	Hydrocarbons
Hexadecane	-4.5	Hydrocarbons
Docosane	-4.5	Hydrocarbons
Pentacosane	-4.5	Hydrocarbons
Tritriacontane	-4.5	Hydrocarbons
6,10-dimethyl-1-undecene	-4.5	Hydrocarbons
9-heptacosene	-4.5	Hydrocarbons
Wax ester 38:0 (16:0-22:0)	-4.5	Hydrocarbons
Wax ester 38:0 (18:0-20:0)	-4.5	Hydrocarbons
Wax ester 40:0 (16:0-24:0)	-4.5	Hydrocarbons
Wax ester 40:1 (18:1-22:0)	-4.5	Hydrocarbons
Wax ester 46:1 (18:1-28:0)	-4.5	Hydrocarbons
Dodecane	-4.4	Hydrocarbons
Octadecane	-4.4	Hydrocarbons
Nonadecane	-4.4	Hydrocarbons
Heptacosane	-4.4	Hydrocarbons
Tricontane	-4.4	Hydrocarbons
Tridecane	-4.4	Hydrocarbons
9-tetracosene	-4.4	Hydrocarbons
Phytyl oleate C18:1	-4.4	Hydrocarbons
Wax ester 40:1 (16:1-24:0)	-4.4	Hydrocarbons
Wax ester 42:0 (14:0-28:0)	-4.4	Hydrocarbons
Wax ester 42:0 (18:0-24:0)	-4.4	Hydrocarbons
Wax ester 42:0 (24:0-18:0)	-4.4	Hydrocarbons
Wax ester 44:1 (18:1-26:0)	-4.4	Hydrocarbons
Wax ester 46:0 (18:0-28:0)	-4.4	Hydrocarbons
Methyl benzene	-4.3	Hydrocarbons
Nonane	-4.2	Hydrocarbons
Wax ester 38:0 (20:0-18:0)	-4.2	Hydrocarbons
Octacosane	-4.1	Hydrocarbons
Hentriacontane	-4.1	Hydrocarbons
Wax ester 42:1 (16:1-26:0)	-4.1	Hydrocarbons
Wax ester 44:1 (16:0-28:0)	-4.1	Hydrocarbons
Tetratriacontane	-4.0	Hydrocarbons
Octane	-3.9	Hydrocarbons
Heptane	-3.8	Hydrocarbons
Wax ester 46:0 (14:0-32:0)	-3.8	Hydrocarbons

Dotriacotane	-3.7	Hydrocarbons
2-methylpentane	-3.6	Hydrocarbons
3-methylpentane	-3.5	Hydrocarbons
2-Methylbutane	-3.3	Hydrocarbons
Hexane	-3.3	Hydrocarbons
Hexene	-3.3	Hydrocarbons
Poly-unsaturated di-galactoside glycerol diester	-6.6	Other
3-[1-(hydroxymethyl)-(E)-1-propenyl] glutaric acid	-6.1	Other
3-(1-Hydroxymethyl-1-propenyl)pentanedioic acid	-5.4	Other
3-[1-(formyl)-(E)-1-propenyl] glutaric acid	-5.3	Other
Halleridone	-5.2	Other
1,5-anhydroxylitol	-4.6	Other
β-Hydroxy verbascoside	-10.7	Phenolic compounds
Acetoside	-10.4	Phenolic compounds
Verbascoside	-10.2	Phenolic compounds
Isoverbascoside	-10.1	Phenolic compounds
Isorhoifolin	-10.0	Phenolic compounds
Oxidized verbascoside	-10.0	Phenolic compounds
Luteolin-4'-O-rutinoside	-9.8	Phenolic compounds
Luteolin-7-O-rutinoside	-9.8	Phenolic compounds
β-Hydroxy-acetoside	-9.8	Phenolic compounds
4'-O-β-D-Glucosyl-9-O-(6''-deoxysaccharosyl)olivil	-9.8	Phenolic compounds
Hesperidin	-9.7	Phenolic compounds
Apigenin-7-O-rutinoside	-9.7	Phenolic compounds
Suspensaside	-9.7	Phenolic compounds
Rutin	-9.6	Phenolic compounds
Cyanidin-3-O-rutinoside	-9.6	Phenolic compounds
Scolymoside	-9.6	Phenolic compounds
Orbanchoside	-9.6	Phenolic compounds
Quercetin 3-O-rutinoside	-9.5	Phenolic compounds
Nüzhenide oleoside	-9.5	Phenolic compounds
Oxidized isoverbascoside	-9.5	Phenolic compounds
Hellicoside	-9.5	Phenolic compounds
Ligstroside derivative 5	-9.4	Phenolic compounds
Isoacteoside	-9.3	Phenolic compounds
Quercetin-3-rhamnoside	-9.3	Phenolic compounds

Oleuricine A	-9.2	Phenolic compounds
Luteolin-3′,7-O-diglucoside	-9.1	Phenolic compounds
Quercitrin	-9.1	Phenolic compounds
Neo-nüzhenide	-9.1	Phenolic compounds
Jaspolyoside	-9.1	Phenolic compounds
Luteolin-7,4-O-diglucoside	-9.0	Phenolic compounds
Luteolin-4'-O-glucoside	-9.0	Phenolic compounds
Oleuropein dimer	-9.0	Phenolic compounds
Wedelosin	-9.0	Phenolic compounds
Cyanidin-3-O-glucoside	-8.9	Phenolic compounds
Luteolin-6-C-glucoside	-8.9	Phenolic compounds
Nüzhenide 11-Methyl oleoside	-8.9	Phenolic compounds
Nüzhenide	-8.9	Phenolic compounds
Demethyloleuropein	-8.9	Phenolic compounds
Luteolin-7-O-glucoside	-8.8	Phenolic compounds
Ligstroside derivative 4	-8.8	Phenolic compounds
Caffeoyl-6'-secologanoside	-8.8	Phenolic compounds
Vicenin-2	-8.7	Phenolic compounds
Ligstroside	-8.7	Phenolic compounds
Quercetin-7-O-glucoside	-8.7	Phenolic compounds
Lucidumoside C	-8.6	Phenolic compounds
Chrysoeriol-7-O-glucoside	-8.6	Phenolic compounds
7''-S-Hydroxyoleuropein	-8.6	Phenolic compounds
Comselogoside	-8.6	Phenolic compounds
Apigenin-7-O-glucoside	-8.4	Phenolic compounds
(+)-1-Acetoxypinoresinol-4'-β-D-glucopyranoside-4''-O-methyl ether	-8.4	Phenolic compounds
Oleuropein	-8.4	Phenolic compounds
Elenolic acid diglucoside	-8.4	Phenolic compounds
Isojaspolyoside A	-8.4	Phenolic compounds
Luteolin-8-C-glucoside	-8.3	Phenolic compounds
Pinoresinol	-8.3	Phenolic compounds
(+)-1-Hydroxypinoresinol-4'-β-D-glucopyranoside	-8.3	Phenolic compounds
Ligstroside-3'-O-β-D-glucopyranoside	-8.3	Phenolic compounds
Jaspolyanoside	-8.3	Phenolic compounds
Oleuropein-3''-Methyl ether	-8.3	Phenolic compounds
Hydroxytyrosol diglucoside	-8.3	Phenolic compounds
6'-Rhamnopyranosyl oleoside	-8.3	Phenolic compounds
Quercetin-3-O-glucoside	-8.2	Phenolic compounds
Hydroxypinoresinol	-8.2	Phenolic compounds
(+)-Fraxiresinol-1-β-D-glucopyranoside	-8.2	Phenolic compounds
Oleuropein diglucoside	-8.2	Phenolic compounds
10-Hydroxyoleuropein	-8.2	Phenolic compounds

Esculin	-8.2	Phenolic compounds
6'-β-D-Glucopyranosyl oleoside	-8.2	Phenolic compounds
Luteolin	-8.1	Phenolic compounds
Delphinidin-3-O-glucoside	-8.1	Phenolic compounds
Rosmarinic acid	-8.1	Phenolic compounds
Dihydro-oleuropein	-8.1	Phenolic compounds
Oleuroside-10-carboxylic acid	-8.1	Phenolic compounds
Oleuricine B	-8.1	Phenolic compounds
Chrysoeriol	-8.0	Phenolic compounds
Eriodictyol	-8.0	Phenolic compounds
1-Acetoxypinoresinol	-8.0	Phenolic compounds
Taxifolin	-7.9	Phenolic compounds
Cyanidin (cation)	-7.9	Phenolic compounds
(+)-1-Hydroxypinoresinol-4"-O-methyl ether	-7.9	Phenolic compounds
(+)-1-Acetoxypinoresinol-4'-β-D-glucopyranoside	-7.9	Phenolic compounds
Demethyloleuropein aglycone	-7.9	Phenolic compounds
Ligstroside derivative 3	-7.9	Phenolic compounds
Verucosin	-7.9	Phenolic compounds
Quercetin	-7.8	Phenolic compounds
Caffeoylglucose	-7.8	Phenolic compounds
Demethylligstroside	-7.8	Phenolic compounds
6'-O-[(2E)-2,6-Dimethyl-8-hydroxy- 2-octenoyloxy]-secologanoside	-7.8	Phenolic compounds
Diosmetin	-7.7	Phenolic compounds
Methoxyluteolin	-7.7	Phenolic compounds
Apigenin	-7.7	Phenolic compounds
Delphinidin	-7.7	Phenolic compounds
Hesperitin	-7.7	Phenolic compounds
Chlorogenic acid	-7.7	Phenolic compounds
Oleuropein-3'-O-β-D-glucopyranoside	-7.7	Phenolic compounds
10-Hydroxy oleuropein aglycone	-7.7	Phenolic compounds
Ligstroside derivative 1	-7.7	Phenolic compounds
Scopolin	-7.7	Phenolic compounds
Oleuroside	-7.6	Phenolic compounds
Oleuropein aglycone (3,4-DHPEA-EA)	-7.6	Phenolic compounds
Loganic Acid	-7.6	Phenolic compounds
Oleoside-11-Methylester	-7.6	Phenolic compounds
Syringaresinol	-7.5	Phenolic compounds
(+)-1-Acetoxypinoresinol-4"-O-methyl ether	-7.5	Phenolic compounds
(-)-Olivil	-7.5	Phenolic compounds

10-Hydroxy oleuropein aglycone decarboxymethyl	-7.5	Phenolic compounds
10-Hydroxy-10-methyl oleuropein aglycone	-7.5	Phenolic compounds
Ligstroside aglycone methyl acetal	-7.5	Phenolic compounds
Oleoside	-7.5	Phenolic compounds
7-Deoxyloganic acid	-7.4	Phenolic compounds
Oleuropeindial - Lactone (Cannizzaro-like product of oleuropeindial)	-7.4	Phenolic compounds
Ligstroside aglycone	-7.4	Phenolic compounds
Hydroxytyrosol-4-β-glucoside	-7.4	Phenolic compounds
Berchemol	-7.3	Phenolic compounds
Elenolic acid glucoside	-7.3	Phenolic compounds
Hydroxytyrosol-1′-β-glucoside	-7.3	Phenolic compounds
1-(3’-Methoxy-4’-hydroxy)- phenyl-6,7-dihydroxyisochroman	-7.2	Phenolic compounds
3,4-DHPEA-DETA	-7.2	Phenolic compounds
Secologanin	-7.2	Phenolic compounds
Cornoside	-7.2	Phenolic compounds
Hydroxytyrosil-elenolate	-7.2	Phenolic compounds
Monoaldehydic form of Oleuropein aglycon	-7.2	Phenolic compounds
(+)-Cycloolivil	-7.2	Phenolic compounds
Hydroxytyrosol rhamnoside	-7.2	Phenolic compounds
Caftaric acid	-7.1	Phenolic compounds
Oleuropeindial (Cannizzaro-like product of oleuropeindial)	-7.1	Phenolic compounds
Secologanoside	-7.1	Phenolic compounds
3-Acetyloxy berchemol	-7.0	Phenolic compounds
Oleoside dimethylester	-7.0	Phenolic compounds
Secologanol	-7.0	Phenolic compounds
demethyloleuropein aglycone (enol form)	-7.0	Phenolic compounds
Demethyloleuropein aglycone dialdehyde	-7.0	Phenolic compounds
Oleuropeindial (keto form)	-7.0	Phenolic compounds
Loganin	-7.0	Phenolic compounds
1-Phenyl-6,7-dihydroxyisochroman	-6.9	Phenolic compounds
Methyl malate-hydroxytyrosol ester	-6.9	Phenolic compounds
Oleuropeindial (enol form)	-6.8	Phenolic compounds
Ligstroside derivative 2	-6.8	Phenolic compounds
Hemiacetal of dialdehydic oleuropein aglycone decarboxymethyl	-6.8	Phenolic compounds
Salidroside	-6.8	Phenolic compounds
Secologanic acid	-6.7	Phenolic compounds
3,4-DHPEA-EDA (Oleuropein-aglycone di-aldehyde)	-6.7	Phenolic compounds

Hydroxytyrosol acetate	-6.6	Phenolic compounds
Oleacein (Dialdehydic form of decarboxymethyl Oleuropein aglycon)	-6.6	Phenolic compounds
Monoaldehydic form of Ligstroside aglycon	-6.6	Phenolic compounds
3,4-DHPEA-DEDA (Oleuropein aglycone decarboxymethyl dialdehyde form)	-6.6	Phenolic compounds
3,4-DHPEA-DEDA (acetal)	-6.6	Phenolic compounds
Hydroxytyrosol acyclodihydroelenolate	-6.6	Phenolic compounds
p-HPEA-EDA	-6.5	Phenolic compounds
Decarboxymethyl ligstroside aglycone	-6.5	Phenolic compounds
Hydroxytyrosol-3-β-glucoside	-6.4	Phenolic compounds
D-(+)-Erythro-1-(4-hydroxy-3-methoxy)-214 - phenyl-1,2,3-propantriol	-6.3	Phenolic compounds
Oleocanthal (Dialdehydic form of decarboxymethyl Ligstroside aglycon)	-6.3	Phenolic compounds
Demethyl elenolic acid	-6.3	Phenolic compounds
Hemiacetal of dialdehydic ligstroside aglycone decarboxymethyl	-6.3	Phenolic compounds
Esculetin	-6.3	Phenolic compounds
Hydroxycaffeic acid	-6.1	Phenolic compounds
Deoxyloganic acid lauryl ester	-6.1	Phenolic compounds
2,3-dihydrocaffeic acid	-6.0	Phenolic compounds
Phloretic acid	-6.0	Phenolic compounds
Caffeic acid	-6.0	Phenolic compounds
Dihydro-p-coumaric acid	-6.0	Phenolic compounds
Tyrosol acetate	-6.0	Phenolic compounds
Quinic acid	-5.9	Phenolic compounds
Gallic acid	-5.9	Phenolic compounds
Protocatechuic acid	-5.9	Phenolic compounds
3,4-Dihydroxyphenylacetic acid	-5.9	Phenolic compounds
p-Coumaric acid	-5.9	Phenolic compounds
Scopoletin	-5.9	Phenolic compounds
3,4-Dihydroxyphenylglycol	-5.8	Phenolic compounds
Shikimic acid	-5.8	Phenolic compounds
2,5-Dihydroxyphenylacetic acid	-5.8	Phenolic compounds
Elenolic acid	-5.8	Phenolic compounds
Gentisic acid	-5.7	Phenolic compounds
o-Coumaric acid	-5.7	Phenolic compounds
Hydroxytyrosol	-5.6	Phenolic compounds
Syringic acid	-5.6	Phenolic compounds
4-Hydroxy-3-methoxy-phenylacetic acid	-5.6	Phenolic compounds
m-Coumaric acid	-5.6	Phenolic compounds
Elenolic acid dialdehyde	-5.6	Phenolic compounds

1-oleyltyrosol	-5.6	Phenolic compounds
2,4 dihydroxybenzoic acid	-5.5	Phenolic compounds
p-Hydroxyphenylacetic acid	-5.5	Phenolic compounds
Homovanillic acid	-5.5	Phenolic compounds
Elenolic acid methylester	-5.5	Phenolic compounds
Homovanillyl alcohol	-5.4	Phenolic compounds
3,4-Dimethoxybenzoic acid	-5.4	Phenolic compounds
4-hydroxybenzoic acid	-5.4	Phenolic compounds
2,6-Dihydroxybenzoic acid	-5.4	Phenolic compounds
Homoveratric acid	-5.4	Phenolic compounds
Sinapic acid	-5.4	Phenolic compounds
Ferulic acid	-5.4	Phenolic compounds
Cinnamic acid	-5.4	Phenolic compounds
Syringaldehyde	-5.3	Phenolic compounds
4-Methylcatechol	-5.3	Phenolic compounds
3,4,5-Trimethoxybenzoic acid	-5.3	Phenolic compounds
4-O-methyl-D-glucuronic acid	-5.3	Phenolic compounds
Isoeugenol	-5.3	Phenolic compounds
Homovanillin	-5.3	Phenolic compounds
Tyrosol	-5.2	Phenolic compounds
Vanillic acid	-5.2	Phenolic compounds
DEDA acetal	-5.2	Phenolic compounds
Catechol	-5.1	Phenolic compounds
4-Ethylguaiacol	-5.1	Phenolic compounds
2,6-Dimethoxybenzoic acid	-5.1	Phenolic compounds
DEDA (Decarboxymethyl elenolic acid dialdehyde)	-5.1	Phenolic compounds
4-Vinylguaiacol	-5.0	Phenolic compounds
2-Methoxy-4-vinylphenol	-5.0	Phenolic compounds
o-cresol	-4.9	Phenolic compounds
4-Vinylphenol	-4.9	Phenolic compounds
4-Hydroxybenzaldehyde	-4.9	Phenolic compounds
Dialdehydic elenolic acid decarboxymethyl	-4.9	Phenolic compounds
Guaiacol	-4.9	Phenolic compounds
m-cresol	-4.8	Phenolic compounds
4-Ethylphenol	-4.8	Phenolic compounds
Dialdehydic elenolic ester decarboxymethyl	-4.8	Phenolic compounds
p-cresol	-4.6	Phenolic compounds
Phenol	-4.5	Phenolic compounds
Phosphatidylinositol	-6.1	Phospholipids
Lysophosphatidylethanolamine	-6.0	Phospholipids
Lysophosphatidic acid	-5.6	Phospholipids

Phosphatidylglycerol	-5.5	Phospholipids
Phosphatidylcholine	-5.3	Phospholipids
Phosphatidylethanolamine	-5.1	Phospholipids
Phosphatidic acid	-4.9	Phospholipids
Chlorophyllide a	-9.5	Pigments
Chlorophyllide b	-9.1	Pigments
Pheophorbide a	-9.1	Pigments
Pheophorbide b	-8.9	Pigments
Pyropheophytin α	-8.1	Pigments
Pheophytin b	-8.1	Pigments
Chlorophyll b	-7.8	Pigments
Pheophytin α	-7.6	Pigments
Chlorophyll a	-7.6	Pigments
Neoxanthin	-5.4	Pigments
28-nor-β-amyrin	-9.2	Sterols
Lupenone	-9.0	Sterols
Germanicol	-8.8	Sterols
Taraxasterol	-8.8	Sterols
δ-amyrin	-8.8	Sterols
β-amyrone	-8.7	Sterols
28-hydroxytaraxerol	-8.7	Sterols
28-nor-α-amyrin	-8.7	Sterols
24-Ethyl-E-23-dehydrolophenol	-8.6	Sterols
α-amyrin	-8.6	Sterols
24-methyl-(E)-23-dehydrolophenol	-8.5	Sterols
24-methyl-24(25)-dehydrolophenol	-8.5	Sterols
Lupeol	-8.5	Sterols
Taraxerol	-8.5	Sterols
4α,14α-Dimethylstigmasta-8,24(24)-dien-3β-ol	-8.5	Sterols
Δ7,22-Ergostadienol	-8.4	Sterols
Ψ-taraxasterol	-8.4	Sterols
Uvaol	-8.4	Sterols
Ergosterol	-8.3	Sterols
Butyrospermol	-8.3	Sterols
3-epi-lupeol	-8.3	Sterols
Methyl 2α,3β-diacetoxyolean-12-en-28-oate	-8.3	Sterols
Methyl 3β-acetoxyolean-12-en-28-oate	-8.3	Sterols
Δ-7-Avenasterol	-8.2	Sterols
Δ-5,23-Stigmastadienol	-8.2	Sterols
Δ-5,24-Stigmastadienol	-8.2	Sterols
Cyclosadol	-8.2	Sterols
β-amyrin	-8.2	Sterols

Brassicasterol	-8.1	Sterols
Cyclobranol	-8.1	Sterols
Bacchar-12,21-dien-3β-ol	-8.1	Sterols
28-isocitrostadienol	-8.0	Sterols
Stigmasterol	-8.0	Sterols
Dammaradienol	-8.0	Sterols
β-sitosterol	-7.9	Sterols
Δ-7-Stigmastenol	-7.9	Sterols
Δ7,24-Ergostadienol	-7.9	Sterols
Cycloartenol	-7.9	Sterols
3-epi-betulin	-7.9	Sterols
Tirucallol	-7.9	Sterols
Δ-5-Avenasterol	-7.8	Sterols
4,4-dimethyl-5α-stigmasta-7,24Z(241)-dien-3β-ol	-7.8	Sterols
Erythrodiol	-7.8	Sterols
24-ethyllophenol	-7.7	Sterols
Campesterol	-7.7	Sterols
(24Z)-24-ethylidene-dihydrolanosterol	-7.7	Sterols
Citrostadienol	-7.6	Sterols
24-Methylene-cholesterol	-7.6	Sterols
24-methylene-24-dihydroparkeol	-7.6	Sterols
24-methylene-24-dihydrolanosterol	-7.6	Sterols
4,4-dimethyl-5α-stigmast-7-en-3β-ol	-7.6	Sterols
Gramisterol	-7.5	Sterols
24-Methylenelophenol	-7.5	Sterols
Cholesterol	-7.5	Sterols
22,23- Dihydrobrassicasterol	-7.5	Sterols
24-methylene-cycloartenol	-7.5	Sterols
Parkeol	-7.5	Sterols
7, 24-tirucalladienol	-7.5	Sterols
24-methylene-31-nor-9(11)-lanostenol	-7.4	Sterols
24-methylene-24-dihydroparkenol	-7.4	Sterols
24-methyl-31-nor-9(11)-lanostenol	-7.3	Sterols
Agrostophyllinol	-7.3	Sterols
Campestanol	-7.2	Sterols
Obtusifoliol	-7.1	Sterols
Cycloeucalenol	-7.0	Sterols
24-Ethylidenelophenol	-7.0	Sterols
Clerosterol	-7.0	Sterols
Stigmastanol	-7.0	Sterols
Linalool	-5.1	Sterols
Mannan	-8.2	Sugars

Maltotriose	-7.8	Sugars
Galactinol	-7.8	Sugars
D-(+)-raffinose	-7.7	Sugars
D-(+)-sucrose	-6.7	Sugars
D-(+)-lactose	-6.5	Sugars
α-Cellulose	-6.4	Sugars
D-(+)-chiro-inositol	-5.9	Sugars
Sedoheptulose	-5.8	Sugars
D-(+)-galacturonic acid	-5.8	Sugars
D-(+)-mannose	-5.7	Sugars
D-(+)-glucose	-5.6	Sugars
Galacturonan	-5.6	Sugars
D-(-)-galactose	-5.5	Sugars
L-rhamnose	-5.5	Sugars
L-Fucose	-5.5	Sugars
myo-inositol	-5.5	Sugars
Pectin	-5.5	Sugars
D-Fucose	-5.4	Sugars
D-(-)-arabinose	-5.2	Sugars
D-(-)-fructose	-5.2	Sugars
D-glucuronic acid	-5.2	Sugars
D-Mannitol	-5.0	Sugars
D-(+)-xylose	-4.9	Sugars
1,6-anhydro-β-D-glucose	-4.9	Sugars
Xylitol	-4.8	Sugars
Adonitol	-4.8	Sugars
L-(-)-arabitol	-4.7	Sugars
β-tocopherol	-7.0	Tocopherols
γ-tocopherol	-7.0	Tocopherols
α-tocopherol	-6.6	Tocopherols
δ-tocopherol	-6.5	Tocopherols
Pomolic acid	-8.9	Triterpenic acids
Oleanolic acid demethyl	-8.6	Triterpenic acids
Ursolic acid	-8.6	Triterpenic acids
Betulinic acid	-8.6	Triterpenic acids
3-epi-betulinic acid	-8.6	Triterpenic acids
Maslinic acid	-8.5	Triterpenic acids
Urs-2β,3β-dihydroxy-12-en-28-oic acid	-8.5	Triterpenic acids
Corosolic acid	-8.4	Triterpenic acids
Oleanolic acid	-8.3	Triterpenic acids
trans-β-Damascenone	-6.0	Volatiles
cis-3-Hexenyl acetate	-5.9	Volatiles
Phenethyl acetate	-5.7	Volatiles

Benzyl acetate	-5.6	Volatiles
Terpineol	-5.4	Volatiles
Ethyl cinnamate	-5.4	Volatiles
1,8-Cineole	-5.3	Volatiles
Gluconic acid	-5.3	Volatiles
Vanillin	-5.2	Volatiles
2-Ethylphenyl acetate	-5.2	Volatiles
3-Methyl-2-pentylfuran	-5.1	Volatiles
3,4-methyl-3-pentenyl furan	-5.1	Volatiles
Citric acid	-5.1	Volatiles
2-Ethyl-5-hexylthiophene	-5.0	Volatiles
2-Hexenyl acetate	-4.9	Volatiles
3-Hexenyl acetate	-4.9	Volatiles
Ethyl cyclohexylcarboxylate	-4.9	Volatiles
Phenylacetaldehyde	-4.8	Volatiles
trans,cis-2,4-Decadienal	-4.8	Volatiles
2-Phenylethanol	-4.8	Volatiles
Lavendulol	-4.8	Volatiles
trans,trans-2,4-Nonadienal	-4.7	Volatiles
2,4-Decadienal	-4.7	Volatiles
trans-4,5-Epoxy-trans-2-decenal	-4.7	Volatiles
2-Octanol	-4.7	Volatiles
Benzyl alcohol	-4.7	Volatiles
Ethyl octanoate	-4.7	Volatiles
6-Methyl-5-hepten-2-one	-4.7	Volatiles
Benzaldehyde	-4.6	Volatiles
trans-2-Decenal	-4.6	Volatiles
3-Octenol	-4.6	Volatiles
Nonanol	-4.6	Volatiles
Decanol	-4.6	Volatiles
Octanoic acid	-4.6	Volatiles
Octyl acetate	-4.6	Volatiles
2-Nonanone	-4.6	Volatiles
Acetophenone	-4.6	Volatiles
Malic acid	-4.6	Volatiles
cis-2-Nonenal	-4.5	Volatiles
trans-2-Nonenal	-4.5	Volatiles
Decanal	-4.5	Volatiles
trans,trans-2,4-Decadienal	-4.5	Volatiles
6-Methyl-5-hepten-3-ol	-4.5	Volatiles
Hexyl acetate	-4.5	Volatiles
Ethyl 3-methylbutanoate	-4.5	Volatiles
Methyl heptanoate	-4.5	Volatiles

Methyl octanoate	-4.5	Volatiles
2-Octanone	-4.5	Volatiles
cis-1,5-Octadien-3-one	-4.5	Volatiles
trans-2-Octenal	-4.4	Volatiles
Heptan-2-ol	-4.4	Volatiles
Octanol	-4.4	Volatiles
Heptanoic acid	-4.4	Volatiles
Isopentyl acetate	-4.4	Volatiles
2-Methylpropyl butanoate	-4.4	Volatiles
Octan-2-one	-4.4	Volatiles
trans-2-Heptenal	-4.3	Volatiles
2,4-Heptadienal	-4.3	Volatiles
Octanal	-4.3	Volatiles
Nonanal	-4.3	Volatiles
2-Heptanol	-4.3	Volatiles
Heptanol	-4.3	Volatiles
2-Methylbutyl acetate	-4.3	Volatiles
3-Octanone	-4.3	Volatiles
l-Octen-3-one	-4.3	Volatiles
2,5-Diethylthiophene	-4.3	Volatiles
cis-2-Heptenal	-4.2	Volatiles
cis-2-Hexenol	-4.2	Volatiles
Hexanoic acid	-4.2	Volatiles
Propyl butanoate	-4.2	Volatiles
Methyl 3-methylbutanoate	-4.2	Volatiles
Ethyl hexanoate	-4.2	Volatiles
3-Propylfuran	-4.2	Volatiles
Heptan-2-one	-4.2	Volatiles
2-Heptanone	-4.2	Volatiles
Heptanal	-4.1	Volatiles
4-Hexenol	-4.1	Volatiles
Butyl acetate	-4.1	Volatiles
Propyl 2-methylpropanoate	-4.1	Volatiles
Methyl hexanoate	-4.1	Volatiles
2-Propylfuran	-4.1	Volatiles
Succinic acid	-4.1	Volatiles
3-Isopropenylthiophene	-4.1	Volatiles
Hexanol	-4.0	Volatiles
cis-3-Hexenol	-4.0	Volatiles
3-Methyl-butanoic acid	-4.0	Volatiles
Ethyl isobutyrate	-4.0	Volatiles
Ethyl 2-methylbutanoate	-4.0	Volatiles
Ethyl-2-methypropanoate	-4.0	Volatiles

Methyl 2-methylbutanoate	-4.0	Volatiles
2,4 Hexadienal	-3.9	Volatiles
cis-3-Hexenal	-3.9	Volatiles
trans-2-Hexenal	-3.9	Volatiles
2-Methyl-3-butenol	-3.9	Volatiles
trans-2-Hexenol	-3.9	Volatiles
trans-3-Hexenol	-3.9	Volatiles
Pentanoic acid	-3.9	Volatiles
Ethyl butanoate	-3.9	Volatiles
4-Methyl-2-pentanone	-3.9	Volatiles
2-Hexanone	-3.9	Volatiles
Hexanal	-3.8	Volatiles
2-Methyl-1-butanol	-3.8	Volatiles
3-Methyl-1-butanol	-3.8	Volatiles
Pentanol	-3.8	Volatiles
Propyl propanoate	-3.8	Volatiles
Methyl pentanoate	-3.8	Volatiles
2-Ethylfuran	-3.8	Volatiles
2-Methyl-2-butenal	-3.7	Volatiles
3-Hexanal	-3.7	Volatiles
cis-2-Hexenal	-3.7	Volatiles
1-Penten-3-ol	-3.7	Volatiles
3-Methylbutyric acid	-3.7	Volatiles
Pentanal	-3.6	Volatiles
3-Pentanol	-3.6	Volatiles
3-Penten-2-ol	-3.6	Volatiles
2-Penten-1-ol	-3.6	Volatiles
Isobutyric acid	-3.6	Volatiles
Butanoic acid	-3.6	Volatiles
Ethyl propanoate	-3.6	Volatiles
Methyl butanoate	-3.6	Volatiles
3-Methyl-2-butanone	-3.6	Volatiles
2-Methylbutanal	-3.5	Volatiles
trans-2-Pentenal	-3.5	Volatiles
cis-2-Pentenal	-3.5	Volatiles
Oxalic acid	-3.5	Volatiles
3-Methylbutanal	-3.4	Volatiles
Butan-1-ol	-3.4	Volatiles
Butan-2-ol	-3.4	Volatiles
l-Penten-3-one	-3.4	Volatiles
3-Pentanone	-3.4	Volatiles
1-Penten-3-one	-3.4	Volatiles
4-Methoxy-2-methyl-2-butanethiol	-3.4	Volatiles

Ethyl acetate	-3.3	Volatiles
3-Methyl-2-butenethiol	-3.3	Volatiles
Propanal	-3.2	Volatiles
Propanoic acid	-3.1	Volatiles
Butan-2-one	-3.1	Volatiles
2-Butanone	-3.1	Volatiles
1-Propanol	-3.0	Volatiles
Methyl acetate	-2.9	Volatiles
Acetic acid	-2.7	Volatiles
Diethyl ether	-2.7	Volatiles
Ethanol	-2.5	Volatiles
Acetaldehyde	-2.3	Volatiles
Methanol	-2.1	Volatiles

Table S5. Predicted binding affinities of the olive-derived compounds against the representative protein structure for cluster 1 of the GIRK4^{G151R} channel.

Compound	Binding Affinity (kcal/mol)	OliveNet^{TM} Class
Geranylgeraniol	-5.3	Aliphatic and aromatic
Phytol	-5.1	Aliphatic and aromatic
Tetracosanol	-5.1	Aliphatic and aromatic
Hexacosanol	-4.8	Aliphatic and aromatic
Pentacosanol	-4.7	Aliphatic and aromatic
Eicosanol	-4.6	Aliphatic and aromatic
Heptacosanol	-4.6	Aliphatic and aromatic
Octacosanol	-4.6	Aliphatic and aromatic
Tricosanol	-4.5	Aliphatic and aromatic
Docosanol	-4.3	Aliphatic and aromatic
Tyrosine	-5.8	Amino acids
Arginine	-5.7	Amino acids
Phenylalanine	-5.3	Amino acids
Glutamine	-4.7	Amino acids
Aspartic Acid	-4.6	Amino acids
Asparagine	-4.6	Amino acids
Glutamic Acid	-4.5	Amino acids
Isoleucine	-4.3	Amino acids
Leucine	-4.2	Amino acids
Serine	-4.1	Amino acids
Valine	-4.1	Amino acids
Alanine	-3.7	Amino acids
Linoelaidic	-5.7	Fatty acids
Linolenic	-5.6	Fatty acids
Trans-palmitoleic	-5.4	Fatty acids
Palmitic	-5.1	Fatty acids
Oleic	-5.1	Fatty acids
Arachidic	-5.1	Fatty acids
Gadoleic	-5.1	Fatty acids
Erucic	-5.1	Fatty acids
Methyl linoleate	-5.0	Fatty acids
Palmitoleic	-5.0	Fatty acids
11-cis-vaccenic	-5.0	Fatty acids
Linoleic	-5.0	Fatty acids
1,2-diacylglycerol	-4.9	Fatty acids
Ethyl linoleate	-4.9	Fatty acids
Cis-10-Heptadecenoic	-4.9	Fatty acids
Petroselinic	-4.9	Fatty acids

Behenic	-4.9	Fatty acids
2,3-diacylglycerol	-4.8	Fatty acids
1,3-diacylglycerol	-4.8	Fatty acids
Methyl palmitate	-4.8	Fatty acids
Methyl oleate	-4.8	Fatty acids
Margaric acid	-4.8	Fatty acids
Stearic	-4.8	Fatty acids
Ethyl palmitate	-4.7	Fatty acids
Ethyl stearate	-4.7	Fatty acids
Methyl stearate	-4.7	Fatty acids
Elaidic	-4.7	Fatty acids
Eicosenoic	-4.7	Fatty acids
Ethyl oleate	-4.6	Fatty acids
Myristic	-4.6	Fatty acids
Lignoceric	-4.6	Fatty acids
Lauric	-4.6	Fatty acids
Methyl heptadecanoate	-4.5	Fatty acids
1-monoacylglycerol	-4.3	Fatty acids
2-monoacylglycerol	-4.3	Fatty acids
3-monoacylglycerol	-4.3	Fatty acids
α-copaene	-6.4	Hydrocarbons
(E)-caryophyllene	-6.4	Hydrocarbons
Calarene	-6.4	Hydrocarbons
Geranylgeranyl C20:1	-6.3	Hydrocarbons
γ-Muurolene	-6.1	Hydrocarbons
Cyclosativene	-6.0	Hydrocarbons
Alloaromadendrene	-6.0	Hydrocarbons
α-Selinene	-6.0	Hydrocarbons
δ-cadinene	-6.0	Hydrocarbons
Eremophilone	-5.9	Hydrocarbons
β-cubebene	-5.9	Hydrocarbons
Phytyl oleate C18:1	-5.9	Hydrocarbons
Phytyl C22:0	-5.9	Hydrocarbons
Geranylgeranyl C22:0	-5.9	Hydrocarbons
Squalene	-5.8	Hydrocarbons
Longicyclene	-5.8	Hydrocarbons
α-trans-bergamotene	-5.8	Hydrocarbons
Drima-7,9(11)-diene	-5.8	Hydrocarbons
Eremophyllene	-5.7	Hydrocarbons
Geranylgeranyl oleate C18:0	-5.7	Hydrocarbons
Phytyl C20:1	-5.7	Hydrocarbons
Geranylgeranyl C20:0	-5.7	Hydrocarbons
β-acoradiene	-5.6	Hydrocarbons

Wax ester 44:1 (16:0-28:0)	-5.6	Hydrocarbons
Phytyl C20:0	-5.5	Hydrocarbons
β-elemene	-5.4	Hydrocarbons
Phytyl C24:0	-5.4	Hydrocarbons
Geranylgeranyl C24:0	-5.4	Hydrocarbons
Wax ester 42:1 (18:1-24:0)	-5.4	Hydrocarbons
β- Sesquiphellandrene	-5.3	Hydrocarbons
(Z)2,(E)4,(E)6-Allofarnesene	-5.3	Hydrocarbons
Wax ester 40:0 (16:0-24:0)	-5.3	Hydrocarbons
Wax ester 40:1 (18:1-22:0)	-5.3	Hydrocarbons
γ-curcumene	-5.2	Hydrocarbons
Wax ester 44:1 (20:1-24:0)	-5.2	Hydrocarbons
Wax ester 46:0 (18:0-28:0)	-5.2	Hydrocarbons
Wax ester 46:0 (24:0-22:0)	-5.2	Hydrocarbons
α-Zingiberene	-5.1	Hydrocarbons
β-Curcumene	-5.1	Hydrocarbons
(E)2,(Z)4,(E)6-Allofarnesene	-5.1	Hydrocarbons
Ethyl oleate	-5.1	Hydrocarbons
Geranylgeranyl oleate C18:1	-5.1	Hydrocarbons
Wax ester 38:0 (16:0-22:0)	-5.1	Hydrocarbons
Wax ester 38:0 (18:0-20:0)	-5.1	Hydrocarbons
Wax ester 40:0 (14:0-26:0)	-5.1	Hydrocarbons
Wax ester 40:0 (20:0-20:0)	-5.1	Hydrocarbons
Wax ester 42:0 (16:0-26:0)	-5.1	Hydrocarbons
Wax ester 46:0 (22:0-24:0)	-5.1	Hydrocarbons
Phytyl oleate C18:0	-5.0	Hydrocarbons
Wax ester 44:1 (18:1-26:0)	-5.0	Hydrocarbons
Wax ester 46:0 (16:0-30:0)	-5.0	Hydrocarbons
Methyl palmitate	-4.9	Hydrocarbons
Methyl oleate	-4.9	Hydrocarbons
Wax ester 38:0 (14:0-24:0)	-4.9	Hydrocarbons
Wax ester 40:0 (18:0-22:0)	-4.9	Hydrocarbons
Wax ester 42:0 (18:0-24:0)	-4.9	Hydrocarbons
Wax ester 42:0 (20:0-22:0)	-4.9	Hydrocarbons
Wax ester 42:0 (24:0-18:0)	-4.9	Hydrocarbons
Wax ester 42:1 (16:1-26:0)	-4.9	Hydrocarbons
Wax ester 44:0 (22:0-22:0)	-4.9	Hydrocarbons
Wax ester 46:1 (18:1-28:0)	-4.9	Hydrocarbons
Tetratriacontane	-4.8	Hydrocarbons
9-pentacosene	-4.8	Hydrocarbons
(Z)-β-farnesene	-4.8	Hydrocarbons
Wax ester 38:0 (20:0-18:0)	-4.8	Hydrocarbons
Wax ester 40:1 (16:1-24:0)	-4.8	Hydrocarbons

Wax ester 42:0 (14:0-28:0)	-4.8	Hydrocarbons
Wax ester 46:0 (14:0-32:0)	-4.8	Hydrocarbons
Dotriacotane	-4.7	Hydrocarbons
9-docosene	-4.7	Hydrocarbons
9-tetracosene	-4.7	Hydrocarbons
(E)-β-farnesene	-4.7	Hydrocarbons
Methyl stearate	-4.7	Hydrocarbons
Wax ester 44:0 (18:0-26:0)	-4.7	Hydrocarbons
Heneicosane	-4.6	Hydrocarbons
Wax ester 38:0 (12:0-26:0)	-4.6	Hydrocarbons
Docosane	-4.5	Hydrocarbons
Hexacpsane	-4.5	Hydrocarbons
6,10-dimethyl-1-undecene	-4.5	Hydrocarbons
9-hexacosene	-4.5	Hydrocarbons
6,10-dimethyl-1-undecane	-4.5	Hydrocarbons
Wax ester 44:0 (16:0-28:0)	-4.5	Hydrocarbons
Wax ester 46:0 (20:0-26:0)	-4.5	Hydrocarbons
Tricosane	-4.4	Hydrocarbons
Octacosane	-4.4	Hydrocarbons
Tricontane	-4.4	Hydrocarbons
Hentriacontane	-4.4	Hydrocarbons
Tritriacontane	-4.4	Hydrocarbons
9-heptacosene	-4.4	Hydrocarbons
Pentadecane	-4.3	Hydrocarbons
Tetracosane	-4.3	Hydrocarbons
Styrene	-4.3	Hydrocarbons
9-tricosene	-4.3	Hydrocarbons
Dodecane	-4.2	Hydrocarbons
Tridecane	-4.2	Hydrocarbons
Tetradecane	-4.2	Hydrocarbons
Icosane	-4.2	Hydrocarbons
Pentacosane	-4.2	Hydrocarbons
Heptacosane	-4.2	Hydrocarbons
Nonacosane	-4.2	Hydrocarbons
Nonane	-4.2	Hydrocarbons
Tridecane	-4.1	Hydrocarbons
8-heptdecene	-4.1	Hydrocarbons
Hexadecane	-4.0	Hydrocarbons
Heptadecane	-4.0	Hydrocarbons
Octadecane	-4.0	Hydrocarbons
Nonadecane	-4.0	Hydrocarbons
Methyl benzene	-4.0	Hydrocarbons
Octane	-3.9	Hydrocarbons

Heptane	-3.8	Hydrocarbons
Hexene	-3.7	Hydrocarbons
Hexane	-3.6	Hydrocarbons
3-methylpentane	-3.4	Hydrocarbons
2-methylpentane	-3.3	Hydrocarbons
2-Methylbutane	-3.1	Hydrocarbons
Poly-unsaturated di-galactoside glycerol diester	-7.7	Other
3-[1-(hydroxymethyl)-(E)-1-propenyl] glutaric acid	-6.1	Other
Halleridone	-5.4	Other
3-[1-(formyl)-(E)-1-propenyl] glutaric acid	-5.3	Other
3-(1-Hydroxymethyl-1-propenyl)pentanedioic acid	-5.2	Other
1,5-anhydroxylitol	-4.8	Other
Apigenin-7-O-rutinoside	-10.0	Phenolic compounds
Isorhoifolin	-9.8	Phenolic compounds
Oxidized isoverbascoside	-9.8	Phenolic compounds
Scolymoside	-9.7	Phenolic compounds
Isoverbascoside	-9.7	Phenolic compounds
Hesperidin	-9.6	Phenolic compounds
Luteolin-7-O-rutinoside	-9.6	Phenolic compounds
Luteolin-3′,7-O-diglucoside	-9.6	Phenolic compounds
Quercetin 3-O-rutinoside	-9.6	Phenolic compounds
Nüzhenide 11-Methyl oleoside	-9.6	Phenolic compounds
4'-O-β-D-Glucosyl-9-O-(6''-deoxysaccharosyl)olivil	-9.6	Phenolic compounds
Nüzhenide oleoside	-9.5	Phenolic compounds
Neo-nüzhenide	-9.4	Phenolic compounds
Isojaspolyoside A	-9.4	Phenolic compounds
Acetoside	-9.4	Phenolic compounds
Isoacteoside	-9.3	Phenolic compounds
Luteolin-7,4-O-diglucoside	-9.2	Phenolic compounds
Luteolin-4'-O-rutinoside	-9.2	Phenolic compounds
Nüzhenide	-9.2	Phenolic compounds
Oleuricine B	-9.2	Phenolic compounds
Verbascoside	-9.2	Phenolic compounds
Rutin	-9.1	Phenolic compounds
Jaspolyanoside	-9.1	Phenolic compounds
Hellicoside	-9.1	Phenolic compounds

β-Hydroxy-acetoside	-9.0	Phenolic compounds
Oxidized verbascoside	-9.0	Phenolic compounds
Suspensaside	-9.0	Phenolic compounds
Luteolin-7-O-glucoside	-8.9	Phenolic compounds
(+)-1-Hydroxypinoresinol-4'-β-D-glucopyranoside	-8.9	Phenolic compounds
Ligstroside derivative 4	-8.9	Phenolic compounds
Cyanidin-3-O-rutinoside	-8.8	Phenolic compounds
(+)-1-Acetoxypinoresinol-4'-β-D-glucopyranoside-4"-O-methyl ether	-8.8	Phenolic compounds
Elenolic acid diglucoside	-8.8	Phenolic compounds
Jaspolyoside	-8.8	Phenolic compounds
Ligstroside derivative 5	-8.8	Phenolic compounds
Orbanchoside	-8.8	Phenolic compounds
Caffeoyl-6'-secologanoside	-8.8	Phenolic compounds
Comselogoside	-8.8	Phenolic compounds
Vicenin-2	-8.7	Phenolic compounds
Chrysoeriol-7-O-glucoside	-8.7	Phenolic compounds
Luteolin-4'-O-glucoside	-8.7	Phenolic compounds
Wedelosin	-8.7	Phenolic compounds
Cyanidin-3-O-glucoside	-8.6	Phenolic compounds
Apigenin-7-O-glucoside	-8.6	Phenolic compounds
Oleuropein diglucoside	-8.6	Phenolic compounds
Oleuroside-10-carboxylic acid	-8.6	Phenolic compounds
Oleuricine A	-8.6	Phenolic compounds
Quercetin-7-O-glucoside	-8.6	Phenolic compounds
Oleuropein dimer	-8.5	Phenolic compounds
Demethyloleuropein	-8.5	Phenolic compounds
Ligstroside	-8.5	Phenolic compounds
Ligstroside derivative 3	-8.5	Phenolic compounds
Lucidumoside C	-8.4	Phenolic compounds
Luteolin-8-C-glucoside	-8.4	Phenolic compounds
β-Hydroxy verbascoside	-8.4	Phenolic compounds
Ligstroside-3'-O-β-D-glucopyranoside	-8.4	Phenolic compounds
6'-Rhamnopyranosyl oleoside	-8.4	Phenolic compounds
7"-S-Hydroxyoleuropein	-8.3	Phenolic compounds
Chlorogenic acid	-8.2	Phenolic compounds
Caffeoylglucose	-8.2	Phenolic compounds
(+)-Fraxiresinol-1-β-D-glucopyranoside	-8.2	Phenolic compounds
Dihydro-oleuropein	-8.2	Phenolic compounds
Hydroxytyrosol diglucoside	-8.2	Phenolic compounds
6'-β-D-Glucopyranosyl oleoside	-8.2	Phenolic compounds
Quercetin-3-O-glucoside	-8.1	Phenolic compounds
Luteolin-6-C-glucoside	-8.1	Phenolic compounds

Quercitrin	-8.1	Phenolic compounds
Delphinidin-3-O-glucoside	-8.1	Phenolic compounds
10-Hydroxyoleuropein	-8.1	Phenolic compounds
Oleuropein-3'-O-β-D-glucopyranoside	-8.1	Phenolic compounds
Quercetin-3-rhamnoside	-8.1	Phenolic compounds
(+)-1-Acetoxypinoresinol-4'-β-D-glucopyranoside	-8.0	Phenolic compounds
Oleuropein	-8.0	Phenolic compounds
Oleuropein aglycone (3,4-DHPEA-EA)	-8.0	Phenolic compounds
Quercetin	-7.9	Phenolic compounds
Delphinidin	-7.9	Phenolic compounds
Syringaresinol	-7.9	Phenolic compounds
1-Acetoxypinoresinol	-7.9	Phenolic compounds
(+)-1-Hydroxypinoresinol-4"-O-methyl ether	-7.9	Phenolic compounds
(+)-1-Acetoxypinoresinol-4"-O-methyl ether	-7.9	Phenolic compounds
Demethyloleuropein aglycone	-7.9	Phenolic compounds
Verucosin	-7.9	Phenolic compounds
Taxifolin	-7.8	Phenolic compounds
Methoxyluteolin	-7.8	Phenolic compounds
Oleuroside	-7.8	Phenolic compounds
10-Hydroxy oleuropein aglycone	-7.8	Phenolic compounds
Ligstroside aglycone	-7.8	Phenolic compounds
Demethylligstroside	-7.8	Phenolic compounds
Esculin	-7.8	Phenolic compounds
Diosmetin	-7.7	Phenolic compounds
Hesperitin	-7.7	Phenolic compounds
(-)-Olivil	-7.7	Phenolic compounds
Oleuropeindial - Lactone (Cannizzaro-like product of oleuropeindial)	-7.7	Phenolic compounds
Oleuropein-3"-Methyl ether	-7.7	Phenolic compounds
6'-O-[(2E)-2,6-Dimethyl-8-hydroxy- 2-octenoyloxy]-secologanoside	-7.7	Phenolic compounds
Cyanidin (cation)	-7.6	Phenolic compounds
Rosmarinic acid	-7.6	Phenolic compounds
Pinoresinol	-7.6	Phenolic compounds
Elenolic acid glucoside	-7.6	Phenolic compounds
Hydroxytyrosil-elenolate	-7.6	Phenolic compounds
Ligstroside derivative 1	-7.6	Phenolic compounds
Eriodictyol	-7.5	Phenolic compounds
Luteolin	-7.5	Phenolic compounds
Hydroxypinoresinol	-7.5	Phenolic compounds
3-Acetyloxy berchemol	-7.5	Phenolic compounds

Oleoside dimethylester	-7.5	Phenolic compounds
10-Hydroxy oleuropein aglycone decarboxymethyl	-7.5	Phenolic compounds
Oleoside	-7.5	Phenolic compounds
Chrysoeriol	-7.4	Phenolic compounds
7-Deoxyloganic acid	-7.4	Phenolic compounds
Oleuropeindial (Cannizzaro-like product of oleuropeindial)	-7.4	Phenolic compounds
(+)-Cycloolivil	-7.4	Phenolic compounds
Ligstroside aglycone methyl acetal	-7.4	Phenolic compounds
Loganic Acid	-7.4	Phenolic compounds
Oleoside-11-Methylester	-7.4	Phenolic compounds
Hydroxytyrosol-1′-β-glucoside	-7.4	Phenolic compounds
Berchemol	-7.3	Phenolic compounds
1-(3’-Methoxy-4’-hydroxy)- phenyl-6,7-dihydroxyisochroman	-7.3	Phenolic compounds
3,4-DHPEA-DETA	-7.3	Phenolic compounds
Scopolin	-7.3	Phenolic compounds
demethyloleuropein aglycone (enol form)	-7.2	Phenolic compounds
Apigenin	-7.1	Phenolic compounds
Caftaric acid	-7.1	Phenolic compounds
10-Hydroxy-10-methyl oleuropein aglycone	-7.1	Phenolic compounds
Monoaldehydic form of Ligstroside aglycon	-7.1	Phenolic compounds
Loganin	-7.1	Phenolic compounds
1-Phenyl-6,7-dihydroxyisochroman	-7.0	Phenolic compounds
Secologanin	-7.0	Phenolic compounds
Cornoside	-7.0	Phenolic compounds
Hydroxytyrosol acyclodihydroelenolate	-7.0	Phenolic compounds
Hydroxytyrosol rhamnoside	-7.0	Phenolic compounds
Salidroside	-7.0	Phenolic compounds
Secologanic acid	-6.9	Phenolic compounds
3,4-DHPEA-DEDA (Oleuropein aglycone decarboxymethyl dialdehyde form)	-6.9	Phenolic compounds
Secologanoside	-6.9	Phenolic compounds
Hydroxytyrosol-4-β-glucoside	-6.9	Phenolic compounds
Secologanol	-6.8	Phenolic compounds
Monoaldehydic form of Oleuropein aglycon	-6.8	Phenolic compounds
Ligstroside derivative 2	-6.8	Phenolic compounds
Hydroxytyrosol-3-β-glucoside	-6.8	Phenolic compounds
Oleuropeindial (enol form)	-6.7	Phenolic compounds

Hydroxycaffeic acid	-6.6	Phenolic compounds
p-HPEA-EDA	-6.5	Phenolic compounds
Hemiacetal of dialdehydic ligstroside aglycone decarboxymethyl	-6.5	Phenolic compounds
Demethyloleuropein aglycone dialdehyde	-6.4	Phenolic compounds
Oleuropeindial (keto form)	-6.4	Phenolic compounds
Oleocanthal (Dialdehydic form of decarboxymethyl Ligstroside aglycon)	-6.3	Phenolic compounds
3,4-DHPEA-DEDA (acetal)	-6.3	Phenolic compounds
Hemiacetal of dialdehydic oleuropein aglycone decarboxymethyl	-6.3	Phenolic compounds
2,3-dihydrocaffeic acid	-6.2	Phenolic compounds
Methyl malate-hydroxytyrosol ester	-6.2	Phenolic compounds
Oleacein (Dialdehydic form of decarboxymethyl Oleuropein aglycon)	-6.2	Phenolic compounds
Caffeic acid	-6.1	Phenolic compounds
D-(+)-Erythro-1-(4-hydroxy-3-methoxy)- 214 - phenyl-1,2,3-propantriol	-6.1	Phenolic compounds
3,4-DHPEA-EDA (Oleuropein-aglycone di-aldehyde)	-6.1	Phenolic compounds
Deoxyloganic acid lauryl ester	-6.1	Phenolic compounds
Sinapic acid	-6.0	Phenolic compounds
Ferulic acid	-6.0	Phenolic compounds
Hydroxytyrosol acetate	-6.0	Phenolic compounds
o-Coumaric acid	-5.9	Phenolic compounds
Elenolic acid methylester	-5.9	Phenolic compounds
Esculetin	-5.9	Phenolic compounds
2,4 dihydroxybenzoic acid	-5.8	Phenolic compounds
m-Coumaric acid	-5.8	Phenolic compounds
Demethyl elenolic acid	-5.8	Phenolic compounds
Syringic acid	-5.7	Phenolic compounds
Gallic acid	-5.7	Phenolic compounds
p-Coumaric acid	-5.7	Phenolic compounds
Tyrosol acetate	-5.7	Phenolic compounds
Elenolic acid	-5.7	Phenolic compounds
Decarboxymethyl ligstroside aglycone	-5.7	Phenolic compounds
Scopoletin	-5.7	Phenolic compounds
Shikimic acid	-5.6	Phenolic compounds
Isoeugenol	-5.6	Phenolic compounds
3,4,5-Trimethoxybenzoic acid	-5.5	Phenolic compounds
Quinic acid	-5.5	Phenolic compounds
Vanillic acid	-5.5	Phenolic compounds
Phloretic acid	-5.5	Phenolic compounds

2,5-Dihydroxyphenylacetic acid	-5.5	Phenolic compounds
Dihydro-p-coumaric acid	-5.5	Phenolic compounds
Elenolic acid dialdehyde	-5.5	Phenolic compounds
Hydroxytyrosol	-5.4	Phenolic compounds
Homovanillyl alcohol	-5.4	Phenolic compounds
Syringaldehyde	-5.4	Phenolic compounds
3,4-Dihydroxyphenylglycol	-5.4	Phenolic compounds
4-Vinylguaiacol	-5.4	Phenolic compounds
3,4-Dimethoxybenzoic acid	-5.4	Phenolic compounds
Protocatechuic acid	-5.4	Phenolic compounds
Gentisic acid	-5.4	Phenolic compounds
4-O-methyl-D-glucuronic acid	-5.4	Phenolic compounds
3,4-Dihydroxyphenylacetic acid	-5.4	Phenolic compounds
4-Hydroxy-3-methoxy-phenylacetic acid	-5.4	Phenolic compounds
2-Methoxy-4-vinylphenol	-5.4	Phenolic compounds
1-oleyltyrosol	-5.4	Phenolic compounds
4-Ethylguaiacol	-5.3	Phenolic compounds
Homoveratric acid	-5.3	Phenolic compounds
Homovanillic acid	-5.3	Phenolic compounds
Cinnamic acid	-5.3	Phenolic compounds
DEDA acetal	-5.3	Phenolic compounds
Homovanillin	-5.3	Phenolic compounds
2,6-Dihydroxybenzoic acid	-5.2	Phenolic compounds
Tyrosol	-5.1	Phenolic compounds
4-Methylcatechol	-5.1	Phenolic compounds
4-hydroxybenzoic acid	-5.1	Phenolic compounds
p-Hydroxyphenylacetic acid	-5.1	Phenolic compounds
Dialdehydic elenolic ester decarboxymethyl	-5.1	Phenolic compounds
DEDA (Decarboxymethyl elenolic acid dialdehyde)	-5.1	Phenolic compounds
4-Ethylphenol	-4.9	Phenolic compounds
4-Vinylphenol	-4.9	Phenolic compounds
2,6-Dimethoxybenzoic acid	-4.9	Phenolic compounds
Dialdehydic elenolic acid decarboxymethyl	-4.9	Phenolic compounds
p-cresol	-4.8	Phenolic compounds
4-Hydroxybenzaldehyde	-4.8	Phenolic compounds
Guaiacol	-4.8	Phenolic compounds
Catechol	-4.7	Phenolic compounds
m-cresol	-4.7	Phenolic compounds
o-cresol	-4.7	Phenolic compounds
Phenol	-4.3	Phenolic compounds
Phosphatidylinositol	-6.9	Phospholipids

Lysophosphatidylethanolamine	-5.8	Phospholipids
Lysophosphatidic acid	-5.5	Phospholipids
Phosphatidylglycerol	-5.5	Phospholipids
Phosphatidylcholine	-5.3	Phospholipids
Phosphatidylethanolamine	-5.2	Phospholipids
Phosphatidic acid	-5.0	Phospholipids
Pheophorbide a	-9.8	Pigments
Pheophorbide b	-9.5	Pigments
Chlorophyllide a	-9.3	Pigments
Chlorophyllide b	-9.2	Pigments
Chlorophyll a	-8.5	Pigments
Pyropheophytin α	-8.4	Pigments
Chlorophyll b	-8.4	Pigments
Pheophytin b	-8.3	Pigments
Pheophytin α	-8.1	Pigments
Neoxanthin	-7.1	Pigments
Germanicol	-9.6	Sterols
Lupenone	-9.6	Sterols
Ψ-taraxasterol	-9.3	Sterols
β-amyrin	-9.2	Sterols
β-amyrone	-8.8	Sterols
α-amyrin	-8.8	Sterols
Uvaol	-8.8	Sterols
Taraxasterol	-8.7	Sterols
Taraxerol	-8.7	Sterols
28-nor-β-amyrin	-8.7	Sterols
Cyclobranol	-8.6	Sterols
28-hydroxytaraxerol	-8.6	Sterols
Erythrodiol	-8.6	Sterols
Parkeol	-8.5	Sterols
3-epi-betulin	-8.5	Sterols
Agrostophyllinol	-8.5	Sterols
δ-amyrin	-8.5	Sterols
28-nor-α-amyrin	-8.5	Sterols
Lupeol	-8.4	Sterols
3-epi-lupeol	-8.4	Sterols
24-methylene-cycloartenol	-8.3	Sterols
Bacchar-12,21-dien-3β-ol	-8.3	Sterols
24-methylene-24-dihydroparkeol	-8.3	Sterols
24-methyl-(E)-23-dehydrolophenol	-8.2	Sterols
Methyl 2α,3β-diacetoxyolean-12-en-28-oate	-8.2	Sterols
Methyl 3β-acetoxyolean-12-en-28-oate	-8.2	Sterols

4,4-dimethyl-5α-stigmast-7-en-3β-ol	-8.2	Sterols
24-methyl-24(25)-dehydrolophenol	-8.1	Sterols
24-Ethylidenelophenol	-8.1	Sterols
Clerosterol	-8.1	Sterols
Butyrospermol	-8.1	Sterols
Dammaradienol	-8.1	Sterols
Tirucallol	-8.1	Sterols
24-Ethyl-E-23-dehydrolophenol	-8.0	Sterols
Ergosterol	-8.0	Sterols
Δ-5,23-Stigmastadienol	-8.0	Sterols
Cycloartenol	-8.0	Sterols
Cyclosadol	-8.0	Sterols
Citrostadienol	-7.9	Sterols
24-methylene-31-nor-9(11)-lanostenol	-7.9	Sterols
Brassicasterol	-7.9	Sterols
24-methylene-24-dihydroparkenol	-7.9	Sterols
7, 24-tirucalladienol	-7.9	Sterols
Obtusifoliol	-7.8	Sterols
24-methyl-31-nor-9(11)-lanostenol	-7.8	Sterols
Campesterol	-7.8	Sterols
Campestanol	-7.8	Sterols
4α,14α-Dimethylstigmasta-8,24(24)-dien-3β-ol	-7.8	Sterols
28-isocitrostadienol	-7.7	Sterols
β-sitosterol	-7.7	Sterols
Stigmasterol	-7.7	Sterols
Stigmastanol	-7.7	Sterols
Δ7,22-Ergostadienol	-7.7	Sterols
24-methylene-24-dihydrolanosterol	-7.7	Sterols
4,4-dimethyl-5α-stigmasta-7,24Z(241)-dien-3β-ol	-7.7	Sterols
Gramisterol	-7.6	Sterols
24-Methylenelophenol	-7.6	Sterols
Δ-7-Avenasterol	-7.6	Sterols
Δ-5-Avenasterol	-7.6	Sterols
Δ7,24-Ergostadienol	-7.6	Sterols
22,23- Dihydrobrassicasterol	-7.6	Sterols
(24Z)-24-ethylidene-dihydrolanosterol	-7.6	Sterols
Cycloeucalenol	-7.5	Sterols
24-ethyllophenol	-7.5	Sterols
Cholesterol	-7.5	Sterols
Δ-5,24-Stigmastadienol	-7.5	Sterols
24-Methylene-cholesterol	-7.3	Sterols
Δ-7-Stigmastenol	-7.2	Sterols

Linalool	-5.3	Sterols
Mannan	-8.5	Sugars
D-(+)-raffinose	-7.7	Sugars
Maltotriose	-7.7	Sugars
α-Cellulose	-7.4	Sugars
Galactinol	-7.0	Sugars
D-(+)-lactose	-6.8	Sugars
D-(+)-sucrose	-6.7	Sugars
Pectin	-6.1	Sugars
D-(+)-galacturonic acid	-5.9	Sugars
D-(-)-galactose	-5.6	Sugars
Galacturonan	-5.6	Sugars
D-(-)-fructose	-5.5	Sugars
D-(+)-glucose	-5.4	Sugars
D-(+)-mannose	-5.4	Sugars
Sedoheptulose	-5.4	Sugars
D-glucuronic acid	-5.4	Sugars
D-Mannitol	-5.3	Sugars
L-rhamnose	-5.2	Sugars
D-(+)-chiro-inositol	-5.2	Sugars
L-Fucose	-5.2	Sugars
D-Fucose	-5.2	Sugars
myo-inositol	-5.2	Sugars
1,6-anhydro-β-D-glucose	-5.1	Sugars
D-(-)-arabinose	-4.8	Sugars
D-(+)-xylose	-4.8	Sugars
Xylitol	-4.6	Sugars
Adonitol	-4.6	Sugars
L-(-)-arabitol	-4.6	Sugars
α-tocopherol	-7.2	Tocopherols
γ-tocopherol	-6.7	Tocopherols
β-tocopherol	-6.6	Tocopherols
δ-tocopherol	-6.1	Tocopherols
Corosolic acid	-9.4	Triterpenic acids
Urs-2β,3β-dihydroxy-12-en-28-oic acid	-9.4	Triterpenic acids
Maslinic acid	-9.1	Triterpenic acids
Pomolic acid	-9.1	Triterpenic acids
Ursolic acid	-8.9	Triterpenic acids
Oleanolic acid demethyl	-8.8	Triterpenic acids
Betulinic acid	-8.8	Triterpenic acids
3-epi-betulinic acid	-8.8	Triterpenic acids
Oleanolic acid	-8.7	Triterpenic acids
Ethyl cinnamate	-5.5	Volatiles

3,4-methyl-3-pentenyl furan	-5.4	Volatiles
trans-β-Damascenone	-5.4	Volatiles
Vanillin	-5.3	Volatiles
Citric acid	-5.2	Volatiles
Gluconic acid	-5.2	Volatiles
Terpineol	-5.1	Volatiles
Phenethyl acetate	-5.1	Volatiles
1,8-Cineole	-5.1	Volatiles
cis-3-Hexenyl acetate	-5.0	Volatiles
Benzyl acetate	-5.0	Volatiles
trans,cis-2,4-Decadienal	-4.8	Volatiles
2-Ethylphenyl acetate	-4.8	Volatiles
Ethyl cyclohexylcarboxylate	-4.8	Volatiles
3-Methyl-2-pentylfuran	-4.8	Volatiles
Malic acid	-4.8	Volatiles
trans,trans-2,4-Nonadienal	-4.7	Volatiles
2,4-Decadienal	-4.7	Volatiles
trans,trans-2,4-Decadienal	-4.7	Volatiles
trans-4,5-Epoxy-trans-2-decenal	-4.7	Volatiles
Lavendulol	-4.7	Volatiles
2-Phenylethanol	-4.6	Volatiles
Decanol	-4.6	Volatiles
trans-2-Nonenal	-4.5	Volatiles
Decanal	-4.5	Volatiles
6-Methyl-5-hepten-3-ol	-4.5	Volatiles
Nonanol	-4.5	Volatiles
Octanoic acid	-4.5	Volatiles
2-Hexenyl acetate	-4.5	Volatiles
3-Hexenyl acetate	-4.5	Volatiles
Octyl acetate	-4.5	Volatiles
Ethyl octanoate	-4.5	Volatiles
6-Methyl-5-hepten-2-one	-4.5	Volatiles
2-Nonanone	-4.5	Volatiles
Acetophenone	-4.5	Volatiles
2-Ethyl-5-hexylthiophene	-4.5	Volatiles
Phenylacetaldehyde	-4.4	Volatiles
trans-2-Octenal	-4.4	Volatiles
Nonanal	-4.4	Volatiles
cis-2-Nonenal	-4.4	Volatiles
trans-2-Decenal	-4.4	Volatiles
Octanol	-4.4	Volatiles
2-Octanol	-4.4	Volatiles
Heptanoic acid	-4.4	Volatiles

Hexyl acetate	-4.4	Volatiles
Methyl octanoate	-4.4	Volatiles
2-Octanone	-4.4	Volatiles
Octan-2-one	-4.4	Volatiles
Octanal	-4.3	Volatiles
3-Octenol	-4.3	Volatiles
Hexanoic acid	-4.3	Volatiles
Ethyl 3-methylbutanoate	-4.3	Volatiles
Methyl heptanoate	-4.3	Volatiles
Succinic acid	-4.3	Volatiles
Benzaldehyde	-4.2	Volatiles
2,4-Heptadienal	-4.2	Volatiles
cis-2-Hexenol	-4.2	Volatiles
Benzyl alcohol	-4.2	Volatiles
Isopentyl acetate	-4.2	Volatiles
2-Methylpropyl butanoate	-4.2	Volatiles
Methyl 3-methylbutanoate	-4.2	Volatiles
l-Octen-3-one	-4.2	Volatiles
cis-1,5-Octadien-3-one	-4.2	Volatiles
trans-2-Heptenal	-4.1	Volatiles
trans-2-Hexenol	-4.1	Volatiles
Heptanol	-4.1	Volatiles
Pentanoic acid	-4.1	Volatiles
2-Methylbutyl acetate	-4.1	Volatiles
Propyl 2-methylpropanoate	-4.1	Volatiles
Methyl hexanoate	-4.1	Volatiles
Ethyl hexanoate	-4.1	Volatiles
2-Propylfuran	-4.1	Volatiles
3-Octanone	-4.1	Volatiles
2,5-Diethylthiophene	-4.1	Volatiles
trans-2-Hexenal	-4.0	Volatiles
Heptanal	-4.0	Volatiles
2-Heptanol	-4.0	Volatiles
4-Hexenol	-4.0	Volatiles
Heptan-2-ol	-4.0	Volatiles
3-Methylbutyric acid	-4.0	Volatiles
3-Methyl-butanoic acid	-4.0	Volatiles
Propyl butanoate	-4.0	Volatiles
Methyl pentanoate	-4.0	Volatiles
3-Propylfuran	-4.0	Volatiles
Heptan-2-one	-4.0	Volatiles
2-Heptanone	-4.0	Volatiles
2,4 Hexadienal	-3.9	Volatiles

Hexanal	-3.9	Volatiles
cis-2-Heptenal	-3.9	Volatiles
Hexanol	-3.9	Volatiles
trans-3-Hexenol	-3.9	Volatiles
Ethyl isobutyrate	-3.9	Volatiles
Ethyl-2-methypropanoate	-3.9	Volatiles
Oxalic acid	-3.9	Volatiles
Pentanol	-3.8	Volatiles
1-Penten-3-ol	-3.8	Volatiles
cis-3-Hexenol	-3.8	Volatiles
Isobutyric acid	-3.8	Volatiles
Butanoic acid	-3.8	Volatiles
Butyl acetate	-3.8	Volatiles
Ethyl butanoate	-3.8	Volatiles
Propyl propanoate	-3.8	Volatiles
Ethyl 2-methylbutanoate	-3.8	Volatiles
Methyl butanoate	-3.8	Volatiles
Methyl 2-methylbutanoate	-3.8	Volatiles
2-Ethylfuran	-3.8	Volatiles
4-Methyl-2-pentanone	-3.8	Volatiles
2-Hexanone	-3.8	Volatiles
3-Isopropenylthiophene	-3.8	Volatiles
2-Methyl-2-butenal	-3.7	Volatiles
cis-3-Hexenal	-3.7	Volatiles
cis-2-Hexenal	-3.7	Volatiles
3-Pentanol	-3.7	Volatiles
2-Penten-1-ol	-3.7	Volatiles
Ethyl propanoate	-3.7	Volatiles
Pentanal	-3.6	Volatiles
trans-2-Pentenal	-3.6	Volatiles
cis-2-Pentenal	-3.6	Volatiles
3-Hexanal	-3.6	Volatiles
2-Methyl-1-butanol	-3.6	Volatiles
3-Methyl-1-butanol	-3.6	Volatiles
3-Methyl-2-butanone	-3.6	Volatiles
3-Pentanone	-3.6	Volatiles
2-Methylbutanal	-3.5	Volatiles
Butan-1-ol	-3.5	Volatiles
2-Methyl-3-butenol	-3.5	Volatiles
3-Penten-2-ol	-3.5	Volatiles
Propanoic acid	-3.5	Volatiles
l-Penten-3-one	-3.5	Volatiles
1-Penten-3-one	-3.5	Volatiles

3-Methylbutanal	-3.4	Volatiles
Ethyl acetate	-3.4	Volatiles
4-Methoxy-2-methyl-2-butanethiol	-3.4	Volatiles
Butan-2-ol	-3.3	Volatiles
Methyl acetate	-3.3	Volatiles
Propanal	-3.2	Volatiles
Butan-2-one	-3.2	Volatiles
2-Butanone	-3.2	Volatiles
3-Methyl-2-butenethiol	-3.2	Volatiles
Acetic acid	-3.1	Volatiles
1-Propanol	-2.9	Volatiles
Diethyl ether	-2.8	Volatiles
Ethanol	-2.5	Volatiles
Acetaldehyde	-2.3	Volatiles
Methanol	-1.9	Volatiles

Table S6. Predicted binding affinities of the olive-derived compounds against the representative protein structure for cluster 2 of the GIRK4^{WT} channel.

Compound	Binding Affinity (kcal/mol)	OliveNet™ Class
Phytol	-6.4	Aliphatic and aromatic
Geranylgeraniol	-5.8	Aliphatic and aromatic
Heptacosanol	-5.8	Aliphatic and aromatic
Tricosanol	-5.5	Aliphatic and aromatic
Tetracosanol	-5.3	Aliphatic and aromatic
Octacosanol	-5.2	Aliphatic and aromatic
Eicosanol	-5.1	Aliphatic and aromatic
Docosanol	-5.1	Aliphatic and aromatic
Hexacosanol	-4.9	Aliphatic and aromatic
Pentacosanol	-4.5	Aliphatic and aromatic
Tyrosine	-5.8	Amino acids
Phenylalanine	-5.7	Amino acids
Arginine	-5.6	Amino acids
Leucine	-4.9	Amino acids
Glutamic Acid	-4.8	Amino acids
Glutamine	-4.8	Amino acids
Asparagine	-4.7	Amino acids
Valine	-4.6	Amino acids
Isoleucine	-4.5	Amino acids
Aspartic Acid	-4.3	Amino acids
Serine	-3.7	Amino acids
Alanine	-3.6	Amino acids
Linolenic	-6.4	Fatty acids
Trans-palmitoleic	-6.2	Fatty acids
Palmitic	-6.0	Fatty acids
Linoleic	-6.0	Fatty acids
Petroselinic	-5.9	Fatty acids
Linoelaidic	-5.9	Fatty acids
Erucic	-5.9	Fatty acids
Ethyl linoleate	-5.8	Fatty acids
Methyl heptadecanoate	-5.8	Fatty acids
Margaric acid	-5.8	Fatty acids
Cis-10-Heptadecenoic	-5.8	Fatty acids
11-cis-vaccenic	-5.8	Fatty acids
Elaidic	-5.8	Fatty acids
Gadoleic	-5.8	Fatty acids
Eicosenoic	-5.8	Fatty acids
Myristic	-5.7	Fatty acids
Palmitoleic	-5.7	Fatty acids

Oleic	-5.7	Fatty acids
Methyl linoleate	-5.6	Fatty acids
Stearic	-5.6	Fatty acids
Arachidic	-5.6	Fatty acids
Behenic	-5.6	Fatty acids
Lauric	-5.6	Fatty acids
Methyl oleate	-5.5	Fatty acids
Ethyl palmitate	-5.4	Fatty acids
Methyl palmitate	-5.4	Fatty acids
Methyl stearate	-5.4	Fatty acids
Lignoceric	-5.2	Fatty acids
Ethyl stearate	-5.1	Fatty acids
Ethyl oleate	-5.0	Fatty acids
2,3-diacylglycerol	-4.9	Fatty acids
1,3-diacylglycerol	-4.9	Fatty acids
1,2-diacylglycerol	-4.6	Fatty acids
3-monoacylglycerol	-4.5	Fatty acids
1-monoacylglycerol	-4.0	Fatty acids
2-monoacylglycerol	-4.0	Fatty acids
(E)2,(Z)4,(E)6-Allofarnesene	-7.1	Hydrocarbons
(Z)2,(E)4,(E)6-Allofarnesene	-7.0	Hydrocarbons
(E)-β-farnesene	-6.9	Hydrocarbons
β-Curcumene	-6.8	Hydrocarbons
β- Sesquiphellandrene	-6.6	Hydrocarbons
α-copaene	-6.5	Hydrocarbons
γ-curcumene	-6.4	Hydrocarbons
(Z)-β-farnesene	-6.4	Hydrocarbons
α-Zingiberene	-6.3	Hydrocarbons
Longicyclene	-6.2	Hydrocarbons
(E)-caryophyllene	-6.1	Hydrocarbons
α-trans-bergamotene	-6.1	Hydrocarbons
γ-Muurolene	-6.1	Hydrocarbons
Nonacosane	-6.0	Hydrocarbons
9-tricosene	-6.0	Hydrocarbons
β-acoradiene	-6.0	Hydrocarbons
α-Selinene	-6.0	Hydrocarbons
8-heptdecene	-5.9	Hydrocarbons
9-pentacosene	-5.9	Hydrocarbons
Alloaromadendrene	-5.9	Hydrocarbons
Ethyl oleate	-5.9	Hydrocarbons
9-tetracosene	-5.8	Hydrocarbons
β-cubebene	-5.8	Hydrocarbons
β-elemene	-5.8	Hydrocarbons

Drima-7,9(11)-diene	-5.8	Hydrocarbons
Geranylgeranyl oleate C18:1	-5.8	Hydrocarbons
Geranylgeranyl oleate C18:0	-5.8	Hydrocarbons
Tricosane	-5.7	Hydrocarbons
Hentriacontane	-5.7	Hydrocarbons
6,10-dimethyl-1-undecene	-5.7	Hydrocarbons
9-hexacosene	-5.7	Hydrocarbons
6,10-dimethyl-1-undecane	-5.7	Hydrocarbons
Phytyl C22:0	-5.7	Hydrocarbons
Octadecane	-5.6	Hydrocarbons
Docosane	-5.6	Hydrocarbons
Cyclosativene	-5.6	Hydrocarbons
Eremophyllene	-5.6	Hydrocarbons
δ-cadinene	-5.6	Hydrocarbons
Calarene	-5.6	Hydrocarbons
Methyl stearate	-5.6	Hydrocarbons
Phytyl oleate C18:0	-5.6	Hydrocarbons
Geranylgeranyl C20:1	-5.6	Hydrocarbons
Tetradecane	-5.5	Hydrocarbons
Nonadecane	-5.5	Hydrocarbons
Icosane	-5.5	Hydrocarbons
Tetracosane	-5.5	Hydrocarbons
9-docosene	-5.5	Hydrocarbons
Squalene	-5.5	Hydrocarbons
Methyl palmitate	-5.5	Hydrocarbons
Tridecane	-5.4	Hydrocarbons
Pentadecane	-5.4	Hydrocarbons
Dotriacotane	-5.4	Hydrocarbons
Tridecane	-5.4	Hydrocarbons
Eremophilone	-5.4	Hydrocarbons
Wax ester 44:1 (16:0-28:0)	-5.4	Hydrocarbons
Hexadecane	-5.3	Hydrocarbons
Heptadecane	-5.3	Hydrocarbons
Pentacosane	-5.3	Hydrocarbons
Hexacpsane	-5.3	Hydrocarbons
Methyl oleate	-5.3	Hydrocarbons
Phytyl C20:1	-5.3	Hydrocarbons
Geranylgeranyl C20:0	-5.3	Hydrocarbons
Wax ester 42:0 (18:0-24:0)	-5.3	Hydrocarbons
Heptacosane	-5.2	Hydrocarbons
Styrene	-5.2	Hydrocarbons
Phytyl C20:0	-5.2	Hydrocarbons
Wax ester 46:1 (18:1-28:0)	-5.2	Hydrocarbons

Methyl benzene	-5.1	Hydrocarbons
9-heptacosene	-5.1	Hydrocarbons
Phytyl C24:0	-5.1	Hydrocarbons
Wax ester 40:0 (18:0-22:0)	-5.1	Hydrocarbons
Dodecane	-5.0	Hydrocarbons
Heneicosane	-5.0	Hydrocarbons
Phytyl oleate C18:1	-5.0	Hydrocarbons
Geranylgeranyl C22:0	-5.0	Hydrocarbons
Wax ester 44:1 (20:1-24:0)	-5.0	Hydrocarbons
Wax ester 46:0 (20:0-26:0)	-5.0	Hydrocarbons
Tritriacontane	-4.9	Hydrocarbons
Nonane	-4.9	Hydrocarbons
Wax ester 38:0 (18:0-20:0)	-4.9	Hydrocarbons
Wax ester 40:0 (16:0-24:0)	-4.9	Hydrocarbons
Wax ester 42:0 (16:0-26:0)	-4.9	Hydrocarbons
Wax ester 42:0 (24:0-18:0)	-4.9	Hydrocarbons
Wax ester 46:0 (24:0-22:0)	-4.9	Hydrocarbons
Wax ester 38:0 (20:0-18:0)	-4.8	Hydrocarbons
Wax ester 42:1 (18:1-24:0)	-4.8	Hydrocarbons
Wax ester 44:0 (16:0-28:0)	-4.8	Hydrocarbons
Wax ester 44:0 (22:0-22:0)	-4.8	Hydrocarbons
Wax ester 44:1 (18:1-26:0)	-4.8	Hydrocarbons
Wax ester 46:0 (22:0-24:0)	-4.8	Hydrocarbons
Octane	-4.7	Hydrocarbons
Wax ester 38:0 (14:0-24:0)	-4.7	Hydrocarbons
Wax ester 40:0 (14:0-26:0)	-4.7	Hydrocarbons
Wax ester 40:1 (16:1-24:0)	-4.7	Hydrocarbons
Wax ester 42:0 (20:0-22:0)	-4.7	Hydrocarbons
Wax ester 42:1 (16:1-26:0)	-4.7	Hydrocarbons
Octacosane	-4.6	Hydrocarbons
Wax ester 38:0 (12:0-26:0)	-4.6	Hydrocarbons
Wax ester 38:0 (16:0-22:0)	-4.6	Hydrocarbons
Wax ester 40:1 (18:1-22:0)	-4.6	Hydrocarbons
Wax ester 42:0 (14:0-28:0)	-4.6	Hydrocarbons
Wax ester 44:0 (18:0-26:0)	-4.6	Hydrocarbons
Wax ester 46:0 (14:0-32:0)	-4.6	Hydrocarbons
Wax ester 46:0 (18:0-28:0)	-4.6	Hydrocarbons
Geranylgeranyl C24:0	-4.5	Hydrocarbons
Tetratriacontane	-4.4	Hydrocarbons
Heptane	-4.4	Hydrocarbons
Tricontane	-4.3	Hydrocarbons
3-methylpentane	-4.3	Hydrocarbons
2-methylpentane	-4.2	Hydrocarbons

Wax ester 46:0 (16:0-30:0)	-4.2	Hydrocarbons
Hexene	-4.1	Hydrocarbons
Hexane	-4.0	Hydrocarbons
Wax ester 40:0 (20:0-20:0)	-4.0	Hydrocarbons
2-Methylbutane	-3.9	Hydrocarbons
Poly-unsaturated di-galactoside glycerol diester	-6.7	Other
3-[1-(hydroxymethyl)-(E)-1-propenyl] glutaric acid	-6.2	Other
Halleridone	-5.5	Other
3-[1-(formyl)-(E)-1-propenyl] glutaric acid	-5.4	Other
3-(1-Hydroxymethyl-1-propenyl)pentanedioic acid	-5.3	Other
1,5-anhydroxylitol	-4.9	Other
Luteolin-4'-O-rutinoside	-10.6	Phenolic compounds
Luteolin-7-O-rutinoside	-10.5	Phenolic compounds
Scolymoside	-10.4	Phenolic compounds
Oleuropein dimer	-10.4	Phenolic compounds
Hesperidin	-10.3	Phenolic compounds
Luteolin-3′,7-O-diglucoside	-10.3	Phenolic compounds
Apigenin-7-O-rutinoside	-10.2	Phenolic compounds
Verbascoside	-10.2	Phenolic compounds
Cyanidin-3-O-rutinoside	-10.1	Phenolic compounds
Isorhoifolin	-10.1	Phenolic compounds
Isoverbascoside	-9.9	Phenolic compounds
Oxidized isoverbascoside	-9.9	Phenolic compounds
Quercetin 3-O-rutinoside	-9.7	Phenolic compounds
Oxidized verbascoside	-9.7	Phenolic compounds
Isoacteoside	-9.7	Phenolic compounds
Luteolin-7,4-O-diglucoside	-9.6	Phenolic compounds
β-Hydroxy verbascoside	-9.6	Phenolic compounds
Acetoside	-9.6	Phenolic compounds
Suspensaside	-9.6	Phenolic compounds
Rutin	-9.5	Phenolic compounds
Luteolin-7-O-glucoside	-9.5	Phenolic compounds
Nüzhenide 11-Methyl oleoside	-9.5	Phenolic compounds
Orbanchoside	-9.5	Phenolic compounds
Quercitrin	-9.4	Phenolic compounds
Ligstroside derivative 4	-9.4	Phenolic compounds
β-Hydroxy-acetoside	-9.4	Phenolic compounds
Chrysoeriol-7-O-glucoside	-9.3	Phenolic compounds
Luteolin-8-C-glucoside	-9.3	Phenolic compounds

Jaspolyoside	-9.3	Phenolic compounds
Hellicoside	-9.3	Phenolic compounds
Quercetin-7-O-glucoside	-9.3	Phenolic compounds
Apigenin-7-O-glucoside	-9.2	Phenolic compounds
(+)-1-Acetoxypinoresinol-4'-β-D-glucopyranoside-4''-O-methyl ether	-9.2	Phenolic compounds
Oleuropein diglucoside	-9.2	Phenolic compounds
Isojaspolyoside A	-9.2	Phenolic compounds
Luteolin-4'-O-glucoside	-9.1	Phenolic compounds
(+)-1-Hydroxypinoresinol-4'-β-D-glucopyranoside	-9.1	Phenolic compounds
Quercetin-3-rhamnoside	-9.1	Phenolic compounds
Vicenin-2	-9.0	Phenolic compounds
Oleuricine A	-9.0	Phenolic compounds
Jaspolyanoside	-9.0	Phenolic compounds
Ligstroside derivative 3	-9.0	Phenolic compounds
Hydroxytyrosol diglucoside	-9.0	Phenolic compounds
Luteolin-6-C-glucoside	-8.9	Phenolic compounds
Oleuricine B	-8.9	Phenolic compounds
6'-Rhamnopyranosyl oleoside	-8.9	Phenolic compounds
Delphinidin-3-O-glucoside	-8.8	Phenolic compounds
Neo-nüzhenide	-8.8	Phenolic compounds
Nüzhenide	-8.8	Phenolic compounds
Ligstroside derivative 1	-8.8	Phenolic compounds
4'-O-β-D-Glucosyl-9-O-(6''-deoxysaccharosyl)olivil	-8.8	Phenolic compounds
Caffeoyl-6'-secologanoside	-8.8	Phenolic compounds
Comselogoside	-8.8	Phenolic compounds
Lucidumoside C	-8.7	Phenolic compounds
Quercetin-3-O-glucoside	-8.7	Phenolic compounds
(+)-Fraxiresinol-1-β-D-glucopyranoside	-8.7	Phenolic compounds
Nüzhenide oleoside	-8.7	Phenolic compounds
Demethyloleuropein	-8.7	Phenolic compounds
Demethylligstroside	-8.7	Phenolic compounds
Cyanidin-3-O-glucoside	-8.6	Phenolic compounds
Rosmarinic acid	-8.6	Phenolic compounds
Oleuropein-3''-Methyl ether	-8.6	Phenolic compounds
Ligstroside derivative 5	-8.6	Phenolic compounds
Quercetin	-8.5	Phenolic compounds
(+)-1-Hydroxypinoresinol-4''-O-methyl ether	-8.5	Phenolic compounds
(+)-1-Acetoxypinoresinol-4'-β-D-glucopyranoside	-8.5	Phenolic compounds
Wedelosin	-8.5	Phenolic compounds

Taxifolin	-8.4	Phenolic compounds
Luteolin	-8.4	Phenolic compounds
Oleuropein-3'-O-β-D-glucopyranoside	-8.4	Phenolic compounds
Ligstroside	-8.4	Phenolic compounds
Eriodictyol	-8.3	Phenolic compounds
Chlorogenic acid	-8.3	Phenolic compounds
Oleuroside-10-carboxylic acid	-8.3	Phenolic compounds
Elenolic acid diglucoside	-8.3	Phenolic compounds
Chrysoeriol	-8.2	Phenolic compounds
Hesperitin	-8.2	Phenolic compounds
1-Acetoxypinoresinol	-8.2	Phenolic compounds
Dihydro-oleuropein	-8.2	Phenolic compounds
Ligstroside-3'-O-β-D-glucopyranoside	-8.2	Phenolic compounds
7''-S-Hydroxyoleuropein	-8.2	Phenolic compounds
6'-β-D-Glucopyranosyl oleoside	-8.2	Phenolic compounds
Diosmetin	-8.1	Phenolic compounds
Apigenin	-8.1	Phenolic compounds
Delphinidin	-8.1	Phenolic compounds
10-Hydroxyoleuropein	-8.1	Phenolic compounds
Demethyloleuropein aglycone	-8.1	Phenolic compounds
Methoxyluteolin	-8.0	Phenolic compounds
Cyanidin (cation)	-8.0	Phenolic compounds
Oleuroside	-8.0	Phenolic compounds
Caffeoylglucose	-7.9	Phenolic compounds
Oleuropein	-7.9	Phenolic compounds
10-Hydroxy oleuropein aglycone	-7.9	Phenolic compounds
10-Hydroxy-10-methyl oleuropein aglycone	-7.9	Phenolic compounds
(+)-Cycloolivil	-7.9	Phenolic compounds
Esculin	-7.9	Phenolic compounds
Syringaresinol	-7.8	Phenolic compounds
Pinoresinol	-7.8	Phenolic compounds
Hydroxypinoresinol	-7.8	Phenolic compounds
Ligstroside derivative 2	-7.8	Phenolic compounds
6'-O-[(2E)-2,6-Dimethyl-8-hydroxy- 2-octenoyloxy]-secologanoside	-7.8	Phenolic compounds
Oleuropeindial - Lactone (Cannizzaro-like product of oleuropeindial)	-7.7	Phenolic compounds
Hydroxytyrosol-1′-β-glucoside	-7.7	Phenolic compounds
(-)-Olivil	-7.6	Phenolic compounds
Berchemol	-7.6	Phenolic compounds
1-(3'-Methoxy-4'-hydroxy)- phenyl-6,7-dihydroxyisochroman	-7.6	Phenolic compounds
3,4-DHPEA-DETA	-7.6	Phenolic compounds

10-Hydroxy oleuropein aglycone decarboxymethyl	-7.6	Phenolic compounds
(+)-1-Acetoxypinoresinol-4"-O-methyl ether	-7.5	Phenolic compounds
3-Acetyloxy berchemol	-7.5	Phenolic compounds
Hydroxytyrosil-elenolate	-7.5	Phenolic compounds
Scopolin	-7.5	Phenolic compounds
Loganin	-7.5	Phenolic compounds
Verucosin	-7.5	Phenolic compounds
Oleoside dimethylester	-7.4	Phenolic compounds
Oleuropein aglycone (3,4-DHPEA-EA)	-7.4	Phenolic compounds
Loganic Acid	-7.4	Phenolic compounds
Oleoside-11-Methylester	-7.4	Phenolic compounds
Oleoside	-7.4	Phenolic compounds
Elenolic acid glucoside	-7.3	Phenolic compounds
Cornoside	-7.3	Phenolic compounds
Monoaldehydic form of Oleuropein aglycon	-7.3	Phenolic compounds
Hydroxytyrosol rhamnoside	-7.3	Phenolic compounds
Hydroxytyrosol-4-β-glucoside	-7.3	Phenolic compounds
Secologanin	-7.2	Phenolic compounds
Caftaric acid	-7.1	Phenolic compounds
7-Deoxyloganic acid	-7.1	Phenolic compounds
Ligstroside aglycone	-7.1	Phenolic compounds
1-Phenyl-6,7-dihydroxyisochroman	-7.0	Phenolic compounds
Monoaldehydic form of Ligstroside aglycon	-7.0	Phenolic compounds
Ligstroside aglycone methyl acetal	-7.0	Phenolic compounds
Secologanoside	-7.0	Phenolic compounds
Salidroside	-7.0	Phenolic compounds
Secologanic acid	-6.9	Phenolic compounds
Secologanol	-6.9	Phenolic compounds
Oleuropeindial (Cannizzaro-like product of oleuropeindial)	-6.9	Phenolic compounds
1-oleyltyrosol	-6.9	Phenolic compounds
Methyl malate-hydroxytyrosol ester	-6.8	Phenolic compounds
Demethyloleuropein aglycone dialdehyde	-6.8	Phenolic compounds
Oleuropeindial (keto form)	-6.8	Phenolic compounds
Hydroxytyrosol acyclodihydroelenolate	-6.8	Phenolic compounds
Deoxyloganic acid lauryl ester	-6.8	Phenolic compounds
Hydroxytyrosol-3-β-glucoside	-6.8	Phenolic compounds
demethyloleuropein aglycone (enol form)	-6.7	Phenolic compounds
Hemiacetal of dialdehydic ligstroside aglycone decarboxymethyl	-6.7	Phenolic compounds

3,4-DHPEA-DEDA (acetal)	-6.6	Phenolic compounds
Esculetin	-6.6	Phenolic compounds
Hydroxycaffeic acid	-6.5	Phenolic compounds
Oleacein (Dialdehydic form of decarboxymethyl Oleuropein aglycon)	-6.5	Phenolic compounds
Oleuropeindial (enol form)	-6.5	Phenolic compounds
Hemiacetal of dialdehydic oleuropein aglycone decarboxymethyl	-6.5	Phenolic compounds
Demethyl elenolic acid	-6.4	Phenolic compounds
Oleocanthal (Dialdehydic form of decarboxymethyl Ligstroside aglycon)	-6.3	Phenolic compounds
2,3-dihydrocaffeic acid	-6.2	Phenolic compounds
Sinapic acid	-6.2	Phenolic compounds
D-(+)-Erythro-1-(4-hydroxy-3-methoxy)- 214 - phenyl-1,2,3-propantriol	-6.2	Phenolic compounds
3,4-DHPEA-EDA (Oleuropein-aglycone di-aldehyde)	-6.2	Phenolic compounds
Decarboxymethyl ligstroside aglycone	-6.2	Phenolic compounds
3,4,5-Trimethoxybenzoic acid	-6.1	Phenolic compounds
Cinnamic acid	-6.1	Phenolic compounds
Hydroxytyrosol acetate	-6.1	Phenolic compounds
Tyrosol acetate	-6.1	Phenolic compounds
Syringic acid	-6.0	Phenolic compounds
Shikimic acid	-6.0	Phenolic compounds
Homovanillic acid	-6.0	Phenolic compounds
Ferulic acid	-6.0	Phenolic compounds
Caffeic acid	-6.0	Phenolic compounds
3,4-DHPEA-DEDA (Oleuropein aglycone decarboxymethyl dialdehyde form)	-6.0	Phenolic compounds
Scopoletin	-6.0	Phenolic compounds
Gallic acid	-5.9	Phenolic compounds
3,4-Dihydroxyphenylacetic acid	-5.9	Phenolic compounds
m-Coumaric acid	-5.9	Phenolic compounds
3,4-Dihydroxyphenylglycol	-5.8	Phenolic compounds
4-O-methyl-D-glucuronic acid	-5.8	Phenolic compounds
4-Hydroxy-3-methoxy-phenylacetic acid	-5.8	Phenolic compounds
p-HPEA-EDA	-5.8	Phenolic compounds
Elenolic acid	-5.8	Phenolic compounds
Syringaldehyde	-5.7	Phenolic compounds
3,4-Dimethoxybenzoic acid	-5.7	Phenolic compounds
Quinic acid	-5.7	Phenolic compounds
Vanillic acid	-5.7	Phenolic compounds
Phloretic acid	-5.7	Phenolic compounds
2,6-Dihydroxybenzoic acid	-5.7	Phenolic compounds

Dihydro-p-coumaric acid	-5.7	Phenolic compounds
Elenolic acid methylester	-5.7	Phenolic compounds
Hydroxytyrosol	-5.6	Phenolic compounds
2,6-Dimethoxybenzoic acid	-5.6	Phenolic compounds
Protocatechuic acid	-5.6	Phenolic compounds
2,4 dihydroxybenzoic acid	-5.6	Phenolic compounds
p-Coumaric acid	-5.6	Phenolic compounds
o-Coumaric acid	-5.6	Phenolic compounds
Homovanillin	-5.6	Phenolic compounds
Homovanillyl alcohol	-5.5	Phenolic compounds
Homoveratric acid	-5.5	Phenolic compounds
2,5-Dihydroxyphenylacetic acid	-5.5	Phenolic compounds
Elenolic acid dialdehyde	-5.5	Phenolic compounds
Isoeugenol	-5.5	Phenolic compounds
m-cresol	-5.4	Phenolic compounds
Gentisic acid	-5.4	Phenolic compounds
p-Hydroxyphenylacetic acid	-5.4	Phenolic compounds
4-Ethylguaiacol	-5.3	Phenolic compounds
4-Vinylguaiacol	-5.3	Phenolic compounds
4-Methylcatechol	-5.2	Phenolic compounds
DEDA acetal	-5.2	Phenolic compounds
2-Methoxy-4-vinylphenol	-5.2	Phenolic compounds
DEDA (Decarboxymethyl elenolic acid dialdehyde)	-5.1	Phenolic compounds
Tyrosol	-4.9	Phenolic compounds
Catechol	-4.9	Phenolic compounds
4-hydroxybenzoic acid	-4.9	Phenolic compounds
Guaiacol	-4.9	Phenolic compounds
Dialdehydic elenolic acid decarboxymethyl	-4.8	Phenolic compounds
Dialdehydic elenolic ester decarboxymethyl	-4.8	Phenolic compounds
Phenol	-4.7	Phenolic compounds
4-Ethylphenol	-4.7	Phenolic compounds
4-Vinylphenol	-4.7	Phenolic compounds
4-Hydroxybenzaldehyde	-4.7	Phenolic compounds
o-cresol	-4.6	Phenolic compounds
p-cresol	-4.4	Phenolic compounds
Phosphatidylinositol	-6.5	Phospholipids
Lysophosphatidic acid	-6.5	Phospholipids
Lysophosphatidylethanolamine	-5.9	Phospholipids
Phosphatidylcholine	-5.2	Phospholipids
Phosphatidylethanolamine	-5.2	Phospholipids
Phosphatidylglycerol	-5.0	Phospholipids

Phosphatidic acid	-4.8	Phospholipids
Chlorophyllide a	-9.3	Pigments
Chlorophyllide b	-9.3	Pigments
Pheophorbide a	-9.1	Pigments
Pyropheophytin α	-9.0	Pigments
Pheophorbide b	-9.0	Pigments
Pheophytin α	-7.9	Pigments
Pheophytin b	-7.7	Pigments
Chlorophyll a	-7.6	Pigments
Chlorophyll b	-7.4	Pigments
Neoxanthin	-6.4	Pigments
β-amyrone	-9.5	Sterols
Germanicol	-9.1	Sterols
Ergosterol	-8.9	Sterols
Lupenone	-8.9	Sterols
24-Ethyl-E-23-dehydrolophenol	-8.8	Sterols
Brassicasterol	-8.8	Sterols
Taraxasterol	-8.8	Sterols
α-amyrin	-8.8	Sterols
Δ-5,23-Stigmastadienol	-8.7	Sterols
Stigmasterol	-8.6	Sterols
Taraxerol	-8.6	Sterols
28-nor-β-amyrin	-8.6	Sterols
28-isocitrostadienol	-8.5	Sterols
Tirucallol	-8.5	Sterols
Ψ-taraxasterol	-8.5	Sterols
28-nor-α-amyrin	-8.5	Sterols
Cyclosadol	-8.4	Sterols
3-epi-lupeol	-8.4	Sterols
28-hydroxytaraxerol	-8.4	Sterols
Uvaol	-8.4	Sterols
Citrostadienol	-8.3	Sterols
24-ethyllophenol	-8.3	Sterols
24-methyl-(E)-23-dehydrolophenol	-8.3	Sterols
24-methyl-24(25)-dehydrolophenol	-8.3	Sterols
Δ-5-Avenasterol	-8.3	Sterols
Δ-5,24-Stigmastadienol	-8.2	Sterols
β-amyrin	-8.2	Sterols
Δ-7-Avenasterol	-8.1	Sterols
22,23- Dihydrobrassicasterol	-8.1	Sterols
24-methylene-cycloartenol	-8.1	Sterols
Parkeol	-8.1	Sterols

Methyl 2α,3β-diacetoxyolean-12-en-28-oate	-8.1	Sterols
Methyl 3β-acetoxyolean-12-en-28-oate	-8.1	Sterols
Erythrodiol	-8.1	Sterols
Campesterol	-8.0	Sterols
Δ7,22-Ergostadienol	-8.0	Sterols
Δ7,24-Ergostadienol	-8.0	Sterols
Cyclobranol	-8.0	Sterols
24-methylene-24-dihydroparkenol	-8.0	Sterols
δ-amyrin	-8.0	Sterols
4α,14α-Dimethylstigmasta-8,24(24)-dien-3β-ol	-8.0	Sterols
Gramisterol	-7.9	Sterols
24-methylene-31-nor-9(11)-lanostenol	-7.9	Sterols
β-sitosterol	-7.9	Sterols
24-Methylene-cholesterol	-7.9	Sterols
Lupeol	-7.9	Sterols
24-Methylenelophenol	-7.8	Sterols
24-methylene-24-dihydroparkeol	-7.8	Sterols
(24Z)-24-ethylidene-dihydrolanosterol	-7.8	Sterols
Obtusifoliol	-7.7	Sterols
Δ-7-Stigmastenol	-7.7	Sterols
Bacchar-12,21-dien-3β-ol	-7.7	Sterols
Cholesterol	-7.6	Sterols
Dammaradienol	-7.6	Sterols
7, 24-tirucalladienol	-7.6	Sterols
24-methylene-24-dihydrolanosterol	-7.6	Sterols
4,4-dimethyl-5α-stigmasta-7,24Z(241)-dien-3β-ol	-7.6	Sterols
Cycloartenol	-7.5	Sterols
24-methyl-31-nor-9(11)-lanostenol	-7.4	Sterols
Butyrospermol	-7.4	Sterols
3-epi-betulin	-7.4	Sterols
Agrostophyllinol	-7.4	Sterols
4,4-dimethyl-5α-stigmast-7-en-3β-ol	-7.4	Sterols
Stigmastanol	-7.2	Sterols
24-Ethylidenelophenol	-6.9	Sterols
Cycloeucalenol	-6.8	Sterols
Clerosterol	-6.8	Sterols
Campestanol	-6.8	Sterols
Linalool	-5.2	Sterols
D-(+)-raffinose	-8.1	Sugars
Maltotriose	-8.1	Sugars
Mannan	-7.8	Sugars

Galactinol	-7.1	Sugars
α-Cellulose	-7.0	Sugars
D-(+)-lactose	-6.9	Sugars
D-(+)-sucrose	-6.5	Sugars
Sedoheptulose	-6.0	Sugars
Galacturonan	-5.9	Sugars
Pectin	-5.8	Sugars
D-(-)-fructose	-5.7	Sugars
D-(+)-galacturonic acid	-5.7	Sugars
D-(+)-chiro-inositol	-5.7	Sugars
D-glucuronic acid	-5.6	Sugars
D-(+)-mannose	-5.5	Sugars
1,6-anhydro-β-D-glucose	-5.5	Sugars
myo-inositol	-5.5	Sugars
D-(+)-glucose	-5.4	Sugars
D-(-)-galactose	-5.3	Sugars
L-Fucose	-5.3	Sugars
D-(-)-arabinose	-5.2	Sugars
D-Fucose	-5.2	Sugars
L-rhamnose	-5.1	Sugars
D-Mannitol	-5.1	Sugars
D-(+)-xylose	-4.9	Sugars
Xylitol	-4.9	Sugars
Adonitol	-4.9	Sugars
L-(-)-arabitol	-4.9	Sugars
β-tocopherol	-8.0	Tocopherols
α-tocopherol	-7.8	Tocopherols
δ-tocopherol	-7.2	Tocopherols
γ-tocopherol	-6.6	Tocopherols
Pomolic acid	-9.3	Triterpenic acids
Corosolic acid	-8.9	Triterpenic acids
Betulinic acid	-8.8	Triterpenic acids
3-epi-betulinic acid	-8.8	Triterpenic acids
Urs-2β,3β-dihydroxy-12-en-28-oic acid	-8.7	Triterpenic acids
Oleanolic acid demethyl	-8.5	Triterpenic acids
Ursolic acid	-8.5	Triterpenic acids
Oleanolic acid	-8.4	Triterpenic acids
Maslinic acid	-8.1	Triterpenic acids
cis-3-Hexenyl acetate	-6.5	Volatiles
Ethyl cinnamate	-6.4	Volatiles
Benzyl acetate	-6.0	Volatiles
6-Methyl-5-hepten-2-one	-5.7	Volatiles
3,4-methyl-3-pentenyl furan	-5.6	Volatiles

2,4-Decadienal	-5.5	Volatiles
trans,trans-2,4-Decadienal	-5.5	Volatiles
trans-β-Damascenone	-5.5	Volatiles
trans,trans-2,4-Nonadienal	-5.4	Volatiles
Decanol	-5.4	Volatiles
trans-2-Decenal	-5.3	Volatiles
trans,cis-2,4-Decadienal	-5.3	Volatiles
Vanillin	-5.3	Volatiles
2-Ethylphenyl acetate	-5.3	Volatiles
Phenethyl acetate	-5.3	Volatiles
Gluconic acid	-5.3	Volatiles
Decanal	-5.2	Volatiles
trans-4,5-Epoxy-trans-2-decenal	-5.2	Volatiles
3-Methyl-2-pentylfuran	-5.2	Volatiles
2-Octanone	-5.2	Volatiles
cis-1,5-Octadien-3-one	-5.2	Volatiles
Acetophenone	-5.2	Volatiles
2-Ethyl-5-hexylthiophene	-5.2	Volatiles
cis-2-Nonenal	-5.1	Volatiles
2-Phenylethanol	-5.1	Volatiles
6-Methyl-5-hepten-3-ol	-5.1	Volatiles
Terpineol	-5.1	Volatiles
Octyl acetate	-5.1	Volatiles
Octan-2-one	-5.1	Volatiles
trans-2-Nonenal	-5.0	Volatiles
Nonanol	-5.0	Volatiles
Lavendulol	-5.0	Volatiles
Octanoic acid	-5.0	Volatiles
Isopentyl acetate	-5.0	Volatiles
2-Hexenyl acetate	-5.0	Volatiles
3-Hexenyl acetate	-5.0	Volatiles
Ethyl hexanoate	-5.0	Volatiles
Methyl heptanoate	-5.0	Volatiles
Ethyl octanoate	-5.0	Volatiles
Citric acid	-5.0	Volatiles
trans-2-Heptenal	-4.9	Volatiles
cis-2-Heptenal	-4.9	Volatiles
trans-2-Octenal	-4.9	Volatiles
Nonanal	-4.9	Volatiles
Benzyl alcohol	-4.9	Volatiles
Heptanoic acid	-4.9	Volatiles
Methyl octanoate	-4.9	Volatiles
Ethyl cyclohexylcarboxylate	-4.9	Volatiles

2-Propylfuran	-4.9	Volatiles
2-Heptanone	-4.9	Volatiles
3-Octanone	-4.9	Volatiles
l-Octen-3-one	-4.9	Volatiles
Benzaldehyde	-4.8	Volatiles
Octanol	-4.8	Volatiles
2-Octanol	-4.8	Volatiles
3-Octenol	-4.8	Volatiles
2-Methylpropyl butanoate	-4.8	Volatiles
Methyl hexanoate	-4.8	Volatiles
Heptan-2-one	-4.8	Volatiles
2-Nonanone	-4.8	Volatiles
2,4-Heptadienal	-4.7	Volatiles
Octanal	-4.7	Volatiles
2-Heptanol	-4.7	Volatiles
Heptan-2-ol	-4.7	Volatiles
Heptanol	-4.7	Volatiles
2-Methylbutyl acetate	-4.7	Volatiles
Hexyl acetate	-4.7	Volatiles
Ethyl 3-methylbutanoate	-4.7	Volatiles
1,8-Cineole	-4.7	Volatiles
3-Propylfuran	-4.7	Volatiles
2,4 Hexadienal	-4.6	Volatiles
Heptanal	-4.6	Volatiles
Phenylacetaldehyde	-4.6	Volatiles
Hexanoic acid	-4.6	Volatiles
Propyl butanoate	-4.6	Volatiles
Ethyl 2-methylbutanoate	-4.6	Volatiles
cis-3-Hexenal	-4.5	Volatiles
cis-2-Hexenal	-4.5	Volatiles
trans-2-Hexenol	-4.5	Volatiles
4-Hexenol	-4.5	Volatiles
Butyl acetate	-4.5	Volatiles
Ethyl isobutyrate	-4.5	Volatiles
Ethyl-2-methypropanoate	-4.5	Volatiles
Propyl 2-methylpropanoate	-4.5	Volatiles
Methyl 3-methylbutanoate	-4.5	Volatiles
trans-2-Hexenal	-4.4	Volatiles
cis-2-Hexenol	-4.4	Volatiles
Pentanoic acid	-4.4	Volatiles
Ethyl butanoate	-4.4	Volatiles
Methyl pentanoate	-4.4	Volatiles
2-Ethylfuran	-4.4	Volatiles

4-Methyl-2-pentanone	-4.4	Volatiles
2-Hexanone	-4.4	Volatiles
Malic acid	-4.4	Volatiles
Succinic acid	-4.4	Volatiles
2-Methyl-2-butenal	-4.3	Volatiles
Hexanal	-4.3	Volatiles
3-Hexanal	-4.3	Volatiles
Hexanol	-4.3	Volatiles
trans-3-Hexenol	-4.3	Volatiles
cis-3-Hexenol	-4.3	Volatiles
3-Methylbutyric acid	-4.3	Volatiles
3-Methyl-butanoic acid	-4.3	Volatiles
Propyl propanoate	-4.3	Volatiles
2,5-Diethylthiophene	-4.3	Volatiles
cis-2-Pentenal	-4.2	Volatiles
2-Methyl-1-butanol	-4.2	Volatiles
2-Penten-1-ol	-4.1	Volatiles
3-Methylbutanal	-4.0	Volatiles
2-Methylbutanal	-4.0	Volatiles
Pentanal	-4.0	Volatiles
trans-2-Pentenal	-4.0	Volatiles
2-Methyl-3-butenol	-4.0	Volatiles
3-Methyl-1-butanol	-4.0	Volatiles
Butanoic acid	-4.0	Volatiles
Ethyl propanoate	-4.0	Volatiles
Methyl 2-methylbutanoate	-4.0	Volatiles
l-Penten-3-one	-4.0	Volatiles
3-Methyl-2-butanone	-4.0	Volatiles
1-Penten-3-one	-4.0	Volatiles
3-Isopropenylthiophene	-4.0	Volatiles
Pentanol	-3.9	Volatiles
3-Pentanol	-3.9	Volatiles
3-Penten-2-ol	-3.9	Volatiles
Isobutyric acid	-3.9	Volatiles
Methyl butanoate	-3.9	Volatiles
3-Pentanone	-3.9	Volatiles
1-Penten-3-ol	-3.8	Volatiles
3-Methyl-2-butenethiol	-3.8	Volatiles
Propanal	-3.7	Volatiles
Ethyl acetate	-3.7	Volatiles
Butan-2-one	-3.7	Volatiles
2-Butanone	-3.7	Volatiles
4-Methoxy-2-methyl-2-butanethiol	-3.6	Volatiles

Butan-1-ol	-3.5	Volatiles
Butan-2-ol	-3.5	Volatiles
Propanoic acid	-3.4	Volatiles
Oxalic acid	-3.4	Volatiles
1-Propanol	-3.0	Volatiles
Methyl acetate	-3.0	Volatiles
Diethyl ether	-3.0	Volatiles
Acetic acid	-2.8	Volatiles
Acetaldehyde	-2.4	Volatiles
Ethanol	-2.4	Volatiles
Methanol	-1.8	Volatiles

Table S7. Predicted binding affinities of the olive-derived compounds against the representative protein structure for cluster 2 of the GIRK4^{G151R} channel.

Compound	Binding Affinity (kcal/mol)	Class
Geranylgeraniol	-6.5	Aliphatic and aromatic
Tetracosanol	-5.4	Aliphatic and aromatic
Eicosanol	-5.3	Aliphatic and aromatic
Tricosanol	-5.3	Aliphatic and aromatic
Hexacosanol	-5.3	Aliphatic and aromatic
Phytol	-5.2	Aliphatic and aromatic
Docosanol	-5.2	Aliphatic and aromatic
Octacosanol	-5.2	Aliphatic and aromatic
Pentacosanol	-5.0	Aliphatic and aromatic
Heptacosanol	-5.0	Aliphatic and aromatic
Tyrosine	-5.8	Amino acids
Phenylalanine	-5.7	Amino acids
Arginine	-5.2	Amino acids
Leucine	-5.0	Amino acids
Glutamic Acid	-4.9	Amino acids
Asparagine	-4.9	Amino acids
Glutamine	-4.9	Amino acids
Isoleucine	-4.8	Amino acids
Aspartic Acid	-4.6	Amino acids
Valine	-4.5	Amino acids
Serine	-3.9	Amino acids
Alanine	-3.7	Amino acids
Linolenic	-6.2	Fatty acids
Linoelaidic	-6.1	Fatty acids
Methyl oleate	-6.0	Fatty acids
Trans-palmitoleic	-5.9	Fatty acids
Petroselinic	-5.9	Fatty acids
Eicosenoic	-5.9	Fatty acids
Erucic	-5.9	Fatty acids
Palmitic	-5.8	Fatty acids
Stearic	-5.7	Fatty acids
Oleic	-5.7	Fatty acids
11-cis-vaccenic	-5.7	Fatty acids
Elaidic	-5.7	Fatty acids
Ethyl linoleate	-5.6	Fatty acids
Methyl palmitate	-5.6	Fatty acids
Methyl heptadecanoate	-5.6	Fatty acids
Margaric acid	-5.6	Fatty acids
Cis-10-Heptadecenoic	-5.6	Fatty acids

Arachidic	-5.6	Fatty acids
Gadoleic	-5.6	Fatty acids
Ethyl palmitate	-5.5	Fatty acids
Ethyl oleate	-5.5	Fatty acids
Lignoceric	-5.4	Fatty acids
Lauric	-5.4	Fatty acids
Palmitoleic	-5.3	Fatty acids
Behenic	-5.3	Fatty acids
Ethyl stearate	-5.0	Fatty acids
Methyl stearate	-5.0	Fatty acids
Linoleic	-5.0	Fatty acids
2,3-diacylglycerol	-4.9	Fatty acids
1,3-diacylglycerol	-4.9	Fatty acids
1,2-diacylglycerol	-4.8	Fatty acids
Methyl linoleate	-4.7	Fatty acids
Myristic	-4.7	Fatty acids
1-monoacylglycerol	-4.6	Fatty acids
3-monoacylglycerol	-4.5	Fatty acids
2-monoacylglycerol	-4.0	Fatty acids
Geranylgeranyl oleate C18:1	-6.8	Hydrocarbons
Longicyclene	-6.6	Hydrocarbons
(E)-β-farnesene	-6.5	Hydrocarbons
Calarene	-6.4	Hydrocarbons
β-cubebene	-6.3	Hydrocarbons
(Z)2,(E)4,(E)6-Allofarnesene	-6.3	Hydrocarbons
Eremophilone	-6.2	Hydrocarbons
Alloaromadendrene	-6.2	Hydrocarbons
δ-cadinene	-6.2	Hydrocarbons
Cyclosativene	-6.1	Hydrocarbons
α-copaene	-6.1	Hydrocarbons
Drima-7,9(11)-diene	-6.1	Hydrocarbons
γ-Muurolene	-6.1	Hydrocarbons
α-Zingiberene	-6.1	Hydrocarbons
(Z)-β-farnesene	-6.1	Hydrocarbons
8-heptdecene	-5.9	Hydrocarbons
(E)-caryophyllene	-5.9	Hydrocarbons
Eremophyllene	-5.9	Hydrocarbons
α-Selinene	-5.9	Hydrocarbons
Squalene	-5.8	Hydrocarbons
β-acoradiene	-5.8	Hydrocarbons
Geranylgeranyl C20:0	-5.8	Hydrocarbons
Phytyl oleate C18:0	-5.7	Hydrocarbons
α-trans-bergamotene	-5.6	Hydrocarbons

Methyl oleate	-5.6	Hydrocarbons
Geranylgeranyl oleate C18:0	-5.6	Hydrocarbons
Tridecane	-5.5	Hydrocarbons
Tetradecane	-5.5	Hydrocarbons
Pentadecane	-5.5	Hydrocarbons
Hexadecane	-5.5	Hydrocarbons
Nonadecane	-5.5	Hydrocarbons
Methyl palmitate	-5.5	Hydrocarbons
Methyl stearate	-5.5	Hydrocarbons
Geranylgeranyl C24:0	-5.5	Hydrocarbons
Wax ester 42:0 (16:0-26:0)	-5.5	Hydrocarbons
Heptadecane	-5.4	Hydrocarbons
Icosane	-5.4	Hydrocarbons
Heneicosane	-5.4	Hydrocarbons
Tetracosane	-5.4	Hydrocarbons
Nonacosane	-5.4	Hydrocarbons
Tridecane	-5.4	Hydrocarbons
β-elemene	-5.4	Hydrocarbons
β-Curcumene	-5.4	Hydrocarbons
Phytyl oleate C18:1	-5.4	Hydrocarbons
Dodecane	-5.3	Hydrocarbons
Octadecane	-5.3	Hydrocarbons
6,10-dimethyl-1-undecene	-5.3	Hydrocarbons
9-pentacosene	-5.3	Hydrocarbons
9-hexacosene	-5.3	Hydrocarbons
6,10-dimethyl-1-undecane	-5.3	Hydrocarbons
γ-curcumene	-5.3	Hydrocarbons
Ethyl oleate	-5.3	Hydrocarbons
Phytyl C20:1	-5.3	Hydrocarbons
Phytyl C22:0	-5.3	Hydrocarbons
Wax ester 40:0 (18:0-22:0)	-5.3	Hydrocarbons
Docosane	-5.2	Hydrocarbons
Hexacpsane	-5.2	Hydrocarbons
Heptacosane	-5.2	Hydrocarbons
Methyl benzene	-5.2	Hydrocarbons
9-tetracosene	-5.2	Hydrocarbons
Phytyl C24:0	-5.2	Hydrocarbons
Geranylgeranyl C20:1	-5.1	Hydrocarbons
Tricontane	-5.0	Hydrocarbons
Hentriacontane	-5.0	Hydrocarbons
9-docosene	-5.0	Hydrocarbons
9-heptacosene	-5.0	Hydrocarbons
(E)2,(Z)4,(E)6-Allofarnesene	-5.0	Hydrocarbons

Phytyl C20:0	-5.0	Hydrocarbons
Wax ester 40:0 (14:0-26:0)	-5.0	Hydrocarbons
Wax ester 42:0 (20:0-22:0)	-5.0	Hydrocarbons
Wax ester 44:1 (18:1-26:0)	-5.0	Hydrocarbons
β- Sesquiphellandrene	-4.9	Hydrocarbons
Wax ester 40:0 (16:0-24:0)	-4.9	Hydrocarbons
Wax ester 40:0 (20:0-20:0)	-4.9	Hydrocarbons
Wax ester 42:1 (18:1-24:0)	-4.9	Hydrocarbons
Wax ester 44:0 (22:0-22:0)	-4.9	Hydrocarbons
Wax ester 44:1 (20:1-24:0)	-4.9	Hydrocarbons
Wax ester 46:0 (14:0-32:0)	-4.9	Hydrocarbons
Wax ester 46:0 (18:0-28:0)	-4.9	Hydrocarbons
Wax ester 46:0 (22:0-24:0)	-4.9	Hydrocarbons
Styrene	-4.8	Hydrocarbons
Wax ester 38:0 (20:0-18:0)	-4.8	Hydrocarbons
Wax ester 42:1 (16:1-26:0)	-4.8	Hydrocarbons
Wax ester 46:0 (20:0-26:0)	-4.8	Hydrocarbons
Wax ester 46:0 (24:0-22:0)	-4.8	Hydrocarbons
Octacosane	-4.7	Hydrocarbons
Nonane	-4.7	Hydrocarbons
9-tricosene	-4.7	Hydrocarbons
Geranylgeranyl C22:0	-4.7	Hydrocarbons
Wax ester 38:0 (14:0-24:0)	-4.7	Hydrocarbons
Wax ester 40:1 (18:1-22:0)	-4.7	Hydrocarbons
Wax ester 42:0 (18:0-24:0)	-4.7	Hydrocarbons
Wax ester 42:0 (24:0-18:0)	-4.7	Hydrocarbons
Wax ester 46:0 (16:0-30:0)	-4.7	Hydrocarbons
Wax ester 46:1 (18:1-28:0)	-4.7	Hydrocarbons
Wax ester 38:0 (12:0-26:0)	-4.6	Hydrocarbons
Wax ester 38:0 (18:0-20:0)	-4.6	Hydrocarbons
Wax ester 40:1 (16:1-24:0)	-4.6	Hydrocarbons
Wax ester 42:0 (14:0-28:0)	-4.6	Hydrocarbons
Wax ester 44:0 (18:0-26:0)	-4.6	Hydrocarbons
Tritriacontane	-4.5	Hydrocarbons
Tetratriacontane	-4.5	Hydrocarbons
Octane	-4.5	Hydrocarbons
Wax ester 44:0 (16:0-28:0)	-4.5	Hydrocarbons
Wax ester 44:1 (16:0-28:0)	-4.5	Hydrocarbons
Tricosane	-4.4	Hydrocarbons
Pentacosane	-4.4	Hydrocarbons
Wax ester 38:0 (16:0-22:0)	-4.4	Hydrocarbons
Dotriacotane	-4.2	Hydrocarbons
Heptane	-4.2	Hydrocarbons

2-methylpentane	-4.1	Hydrocarbons
3-methylpentane	-4.1	Hydrocarbons
Hexane	-3.9	Hydrocarbons
Hexene	-3.9	Hydrocarbons
2-Methylbutane	-3.7	Hydrocarbons
Poly-unsaturated di-galactoside glycerol diester	-6.9	Other
3-[1-(hydroxymethyl)-(E)-1-propenyl] glutaric acid	-5.9	Other
3-(1-Hydroxymethyl-1-propenyl)pentanedioic acid	-5.2	Other
Halleridone	-5.1	Other
3-[1-(formyl)-(E)-1-propenyl] glutaric acid	-5.0	Other
1,5-anhydroxylitol	-4.4	Other
Isorhoifolin	-9.6	Phenolic compounds
Rutin	-9.5	Phenolic compounds
Hellicoside	-9.4	Phenolic compounds
4'-O-β-D-Glucosyl-9-O-(6''-deoxysaccharosyl)olivil	-9.4	Phenolic compounds
Cyanidin-3-O-rutinoside	-9.3	Phenolic compounds
Oxidized isoverbascoside	-9.3	Phenolic compounds
Luteolin-7-O-rutinoside	-9.2	Phenolic compounds
Apigenin-7-O-rutinoside	-9.2	Phenolic compounds
Demethylligstroside	-9.2	Phenolic compounds
Ligstroside derivative 4	-9.2	Phenolic compounds
Luteolin-3′,7-O-diglucoside	-9.1	Phenolic compounds
Nüzhenide 11-Methyl oleoside	-9.1	Phenolic compounds
Demethyloleuropein	-9.1	Phenolic compounds
Hesperidin	-9.0	Phenolic compounds
Nüzhenide oleoside	-9.0	Phenolic compounds
Oleuropein-3'-O-β-D-glucopyranoside	-9.0	Phenolic compounds
Scolymoside	-8.9	Phenolic compounds
Luteolin-4'-O-rutinoside	-8.9	Phenolic compounds
Luteolin-7-O-glucoside	-8.9	Phenolic compounds
Quercetin 3-O-rutinoside	-8.9	Phenolic compounds
Oleuricine A	-8.9	Phenolic compounds
Verbascoside	-8.9	Phenolic compounds
Luteolin-7,4-O-diglucoside	-8.8	Phenolic compounds
Chrysoeriol-7-O-glucoside	-8.8	Phenolic compounds
Jaspolyanoside	-8.8	Phenolic compounds
Wedelosin	-8.8	Phenolic compounds
Apigenin-7-O-glucoside	-8.7	Phenolic compounds
Jaspolyoside	-8.7	Phenolic compounds

Ligstroside	-8.7	Phenolic compounds
Orbanchoside	-8.6	Phenolic compounds
10-Hydroxyoleuropein	-8.5	Phenolic compounds
Isojaspolyoside A	-8.5	Phenolic compounds
Ligstroside derivative 3	-8.5	Phenolic compounds
Ligstroside derivative 5	-8.5	Phenolic compounds
β-Hydroxy-acetoside	-8.5	Phenolic compounds
Isoverbascoside	-8.5	Phenolic compounds
Suspensaside	-8.5	Phenolic compounds
Oleuropein dimer	-8.4	Phenolic compounds
Oleuroside-10-carboxylic acid	-8.4	Phenolic compounds
Isoacteoside	-8.4	Phenolic compounds
Comselogoside	-8.4	Phenolic compounds
6'-Rhamnopyranosyl oleoside	-8.4	Phenolic compounds
Vicenin-2	-8.3	Phenolic compounds
Luteolin-6-C-glucoside	-8.3	Phenolic compounds
β-Hydroxy verbascoside	-8.3	Phenolic compounds
Nüzhenide	-8.3	Phenolic compounds
Demethyloleuropein aglycone	-8.3	Phenolic compounds
Oxidized verbascoside	-8.3	Phenolic compounds
Acetoside	-8.3	Phenolic compounds
Quercetin-3-rhamnoside	-8.3	Phenolic compounds
Quercetin-7-O-glucoside	-8.3	Phenolic compounds
Quercitrin	-8.2	Phenolic compounds
(+)-1-Hydroxypinoresinol-4'-β-D-glucopyranoside	-8.2	Phenolic compounds
(+)-1-Acetoxypinoresinol-4'-β-D-glucopyranoside-4"-O-methyl ether	-8.2	Phenolic compounds
Oleuroside	-8.2	Phenolic compounds
Cyanidin-3-O-glucoside	-8.1	Phenolic compounds
Chlorogenic acid	-8.1	Phenolic compounds
Pinoresinol	-8.1	Phenolic compounds
(+)-1-Hydroxypinoresinol-4"-O-methyl ether	-8.1	Phenolic compounds
Neo-nüzhenide	-8.1	Phenolic compounds
7"-S-Hydroxyoleuropein	-8.1	Phenolic compounds
Hydroxytyrosol diglucoside	-8.1	Phenolic compounds
Luteolin-8-C-glucoside	-8.0	Phenolic compounds
(+)-1-Acetoxypinoresinol-4'-β-D-glucopyranoside	-8.0	Phenolic compounds
Oleuropein-3"-Methyl ether	-8.0	Phenolic compounds
Ligstroside derivative 1	-8.0	Phenolic compounds
6'-β-D-Glucopyranosyl oleoside	-8.0	Phenolic compounds

Lucidumoside C	-7.9	Phenolic compounds
Luteolin-4’-O-glucoside	-7.9	Phenolic compounds
Hydroxypinoresinol	-7.9	Phenolic compounds
(+)-Fraxiresinol-1-β-D-glucopyranoside	-7.9	Phenolic compounds
Oleuropein	-7.9	Phenolic compounds
Elenolic acid diglucoside	-7.9	Phenolic compounds
Quercetin-3-O-glucoside	-7.8	Phenolic compounds
Delphinidin-3-O-glucoside	-7.8	Phenolic compounds
Rosmarinic acid	-7.8	Phenolic compounds
Caffeoylglucose	-7.8	Phenolic compounds
1-Acetoxypinoresinol	-7.7	Phenolic compounds
Monoaldehydic form of Ligstroside aglycon	-7.7	Phenolic compounds
Quercetin	-7.6	Phenolic compounds
Syringaresinol	-7.6	Phenolic compounds
(+)-1-Acetoxypinoresinol-4"-O-methyl ether	-7.6	Phenolic compounds
Oleuropein diglucoside	-7.6	Phenolic compounds
Hydroxytyrosil-elenolate	-7.6	Phenolic compounds
10-Hydroxy oleuropein aglycone	-7.6	Phenolic compounds
Esculin	-7.6	Phenolic compounds
Salidroside	-7.6	Phenolic compounds
Methoxyluteolin	-7.5	Phenolic compounds
Hesperitin	-7.5	Phenolic compounds
Oleuropeindial - Lactone (Cannizzaro-like product of oleuropeindial)	-7.5	Phenolic compounds
Hydroxytyrosol rhamnoside	-7.5	Phenolic compounds
Diosmetin	-7.4	Phenolic compounds
Eriodictyol	-7.4	Phenolic compounds
Luteolin	-7.4	Phenolic compounds
(-)-Olivil	-7.4	Phenolic compounds
Oleuropein aglycone (3,4-DHPEA-EA)	-7.4	Phenolic compounds
7-Deoxyloganic acid	-7.4	Phenolic compounds
Oleuricine B	-7.4	Phenolic compounds
Scopolin	-7.4	Phenolic compounds
Taxifolin	-7.3	Phenolic compounds
Cyanidin (cation)	-7.3	Phenolic compounds
Delphinidin	-7.3	Phenolic compounds
Dihydro-oleuropein	-7.3	Phenolic compounds
Loganic Acid	-7.3	Phenolic compounds
Oleoside dimethylester	-7.2	Phenolic compounds
Ligstroside aglycone	-7.2	Phenolic compounds
(+)-Cycloolivil	-7.2	Phenolic compounds

Loganin	-7.2	Phenolic compounds
Oleoside-11-Methylester	-7.2	Phenolic compounds
Verucosin	-7.2	Phenolic compounds
Caffeoyl-6'-secologanoside	-7.2	Phenolic compounds
Apigenin	-7.1	Phenolic compounds
3-Acetyloxy berchemol	-7.1	Phenolic compounds
Berchemol	-7.1	Phenolic compounds
1-(3'-Methoxy-4'-hydroxy)- phenyl-6,7-dihydroxyisochroman	-7.1	Phenolic compounds
Ligstroside-3'-O-β-D-glucopyranoside	-7.1	Phenolic compounds
Oleuropeindial (keto form)	-7.1	Phenolic compounds
6'-O-[(2E)-2,6-Dimethyl-8-hydroxy- 2-octenoyloxy]-secologanoside	-7.1	Phenolic compounds
Oleoside	-7.1	Phenolic compounds
Secologanoside	-7.1	Phenolic compounds
Chrysoeriol	-7.0	Phenolic compounds
3,4-DHPEA-DETA	-7.0	Phenolic compounds
Secologanic acid	-7.0	Phenolic compounds
Cornoside	-7.0	Phenolic compounds
Hydroxytyrosol-4-β-glucoside	-7.0	Phenolic compounds
10-Hydroxy-10-methyl oleuropein aglycone	-6.9	Phenolic compounds
Monoaldehydic form of Oleuropein aglycon	-6.9	Phenolic compounds
o-Coumaric acid	-6.8	Phenolic compounds
Caftaric acid	-6.8	Phenolic compounds
Methyl malate-hydroxytyrosol ester	-6.8	Phenolic compounds
Elenolic acid glucoside	-6.8	Phenolic compounds
Secologanol	-6.8	Phenolic compounds
Secologanin	-6.8	Phenolic compounds
p-HPEA-EDA	-6.8	Phenolic compounds
3,4-DHPEA-DEDA (Oleuropein aglycone decarboxymethyl dialdehyde form)	-6.8	Phenolic compounds
Decarboxymethyl ligstroside aglycone	-6.8	Phenolic compounds
Hydroxytyrosol-1′-β-glucoside	-6.8	Phenolic compounds
m-Coumaric acid	-6.7	Phenolic compounds
Cinnamic acid	-6.7	Phenolic compounds
1-Phenyl-6,7-dihydroxyisochroman	-6.7	Phenolic compounds
Oleuropeindial (enol form)	-6.7	Phenolic compounds
demethyloleuropein aglycone (enol form)	-6.7	Phenolic compounds
Hemiacetal of dialdehydic oleuropein aglycone decarboxymethyl	-6.7	Phenolic compounds

10-Hydroxy oleuropein aglycone decarboxymethyl	-6.6	Phenolic compounds
Demethyloleuropein aglycone dialdehyde	-6.6	Phenolic compounds
Hydroxytyrosol-3-β-glucoside	-6.6	Phenolic compounds
Hydroxytyrosol acetate	-6.5	Phenolic compounds
Oleuropeindial (Cannizzaro-like product of oleuropeindial)	-6.5	Phenolic compounds
Ligstroside derivative 2	-6.5	Phenolic compounds
Hemiacetal of dialdehydic ligstroside aglycone decarboxymethyl	-6.5	Phenolic compounds
Hydroxytyrosol acyclodihydroelenolate	-6.4	Phenolic compounds
Ligstroside aglycone methyl acetal	-6.4	Phenolic compounds
Hydroxytyrosol	-6.3	Phenolic compounds
3,4-Dihydroxyphenylacetic acid	-6.3	Phenolic compounds
Caffeic acid	-6.3	Phenolic compounds
Oleacein (Dialdehydic form of decarboxymethyl Oleuropein aglycon)	-6.3	Phenolic compounds
3,4-DHPEA-DEDA (acetal)	-6.3	Phenolic compounds
Deoxyloganic acid lauryl ester	-6.3	Phenolic compounds
Tyrosol acetate	-6.2	Phenolic compounds
Tyrosol	-6.1	Phenolic compounds
Phloretic acid	-6.1	Phenolic compounds
p-Hydroxyphenylacetic acid	-6.1	Phenolic compounds
Esculetin	-6.1	Phenolic compounds
Scopoletin	-6.1	Phenolic compounds
2,5-Dihydroxyphenylacetic acid	-6.0	Phenolic compounds
Oleocanthal (Dialdehydic form of decarboxymethyl Ligstroside aglycon)	-6.0	Phenolic compounds
4-Ethylphenol	-5.9	Phenolic compounds
Protocatechuic acid	-5.9	Phenolic compounds
2,4 dihydroxybenzoic acid	-5.9	Phenolic compounds
D-(+)-Erythro-1-(4-hydroxy-3-methoxy)- 214 - phenyl-1,2,3-propantriol	-5.9	Phenolic compounds
Demethyl elenolic acid	-5.9	Phenolic compounds
m-cresol	-5.8	Phenolic compounds
4-Ethylguaiacol	-5.8	Phenolic compounds
4-Vinylguaiacol	-5.8	Phenolic compounds
4-Vinylphenol	-5.8	Phenolic compounds
2,3-dihydrocaffeic acid	-5.8	Phenolic compounds
Gallic acid	-5.8	Phenolic compounds
Gentisic acid	-5.8	Phenolic compounds
4-hydroxybenzoic acid	-5.8	Phenolic compounds
p-Coumaric acid	-5.8	Phenolic compounds

Hydroxycaffeic acid	-5.8	Phenolic compounds
Elenolic acid	-5.8	Phenolic compounds
Elenolic acid methylester	-5.8	Phenolic compounds
2-Methoxy-4-vinylphenol	-5.8	Phenolic compounds
4-Hydroxybenzaldehyde	-5.7	Phenolic compounds
Vanillic acid	-5.7	Phenolic compounds
Ferulic acid	-5.7	Phenolic compounds
Isoeugenol	-5.7	Phenolic compounds
4-Methylcatechol	-5.6	Phenolic compounds
Sinapic acid	-5.6	Phenolic compounds
3,4-DHPEA-EDA (Oleuropein-aglycone di-aldehyde)	-5.6	Phenolic compounds
Guaiacol	-5.6	Phenolic compounds
p-cresol	-5.5	Phenolic compounds
o-cresol	-5.5	Phenolic compounds
Homovanillic acid	-5.5	Phenolic compounds
Dihydro-p-coumaric acid	-5.5	Phenolic compounds
Homoveratric acid	-5.4	Phenolic compounds
1-oleyltyrosol	-5.4	Phenolic compounds
3,4,5-Trimethoxybenzoic acid	-5.3	Phenolic compounds
3,4-Dimethoxybenzoic acid	-5.3	Phenolic compounds
4-Hydroxy-3-methoxy-phenylacetic acid	-5.3	Phenolic compounds
Elenolic acid dialdehyde	-5.3	Phenolic compounds
DEDA acetal	-5.3	Phenolic compounds
3,4-Dihydroxyphenylglycol	-5.2	Phenolic compounds
Catechol	-5.2	Phenolic compounds
Syringic acid	-5.2	Phenolic compounds
Quinic acid	-5.2	Phenolic compounds
Shikimic acid	-5.2	Phenolic compounds
Homovanillyl alcohol	-5.1	Phenolic compounds
Phenol	-5.1	Phenolic compounds
2,6-Dimethoxybenzoic acid	-5.1	Phenolic compounds
4-O-methyl-D-glucuronic acid	-5.1	Phenolic compounds
Homovanillin	-5.1	Phenolic compounds
2,6-Dihydroxybenzoic acid	-5.0	Phenolic compounds
Syringaldehyde	-4.9	Phenolic compounds
DEDA (Decarboxymethyl elenolic acid dialdehyde)	-4.9	Phenolic compounds
Dialdehydic elenolic acid decarboxymethyl	-4.8	Phenolic compounds
Dialdehydic elenolic ester decarboxymethyl	-4.8	Phenolic compounds
Lysophosphatidylethanolamine	-6.6	Phospholipids

Phosphatidylinositol	-6.3	Phospholipids
Lysophosphatidic acid	-6.2	Phospholipids
Phosphatidylcholine	-5.1	Phospholipids
Phosphatidic acid	-5.0	Phospholipids
Phosphatidylglycerol	-4.9	Phospholipids
Phosphatidylethanolamine	-4.8	Phospholipids
Pheophorbide a	-9.1	Pigments
Pheophorbide b	-9.1	Pigments
Chlorophyllide a	-9.0	Pigments
Chlorophyllide b	-8.7	Pigments
Chlorophyll a	-8.4	Pigments
Neoxanthin	-8.2	Pigments
Pyropheophytin α	-8.1	Pigments
Chlorophyll b	-7.9	Pigments
Pheophytin α	-7.0	Pigments
Pheophytin b	-7.0	Pigments
Taraxasterol	-9.5	Sterols
Dammaradienol	-9.4	Sterols
Taraxerol	-9.2	Sterols
28-nor-β-amyrin	-9.2	Sterols
β-amyrone	-9.0	Sterols
28-hydroxytaraxerol	-9.0	Sterols
Germanicol	-8.9	Sterols
24-methylene-24-dihydrolanosterol	-8.9	Sterols
3-epi-lupeol	-8.8	Sterols
δ-amyrin	-8.8	Sterols
28-nor-α-amyrin	-8.8	Sterols
4α,14α-Dimethylstigmasta-8,24(24)-dien-3β-ol	-8.8	Sterols
Methyl 3β-acetoxyolean-12-en-28-oate	-8.8	Sterols
β-amyrin	-8.7	Sterols
Methyl 2α,3β-diacetoxyolean-12-en-28-oate	-8.7	Sterols
Bacchar-12,21-dien-3β-ol	-8.7	Sterols
(24Z)-24-ethylidene-dihydrolanosterol	-8.7	Sterols
4,4-dimethyl-5α-stigmast-7-en-3β-ol	-8.7	Sterols
Lupenone	-8.6	Sterols
α-amyrin	-8.6	Sterols
Ψ-taraxasterol	-8.6	Sterols
7, 24-tirucalladienol	-8.5	Sterols
Tirucallol	-8.5	Sterols
4,4-dimethyl-5α-stigmasta-7,24Z(241)-dien-3β-ol	-8.5	Sterols
24-methylene-31-nor-9(11)-lanostenol	-8.4	Sterols

24-methyl-(E)-23-dehydrolophenol	-8.4	Sterols
24-methylene-24-dihydroparkenol	-8.4	Sterols
Citrostadienol	-8.3	Sterols
24-methyl-31-nor-9(11)-lanostenol	-8.3	Sterols
Brassicasterol	-8.3	Sterols
Clerosterol	-8.3	Sterols
Ergosterol	-8.3	Sterols
24-methylene-24-dihydroparkeol	-8.3	Sterols
Gramisterol	-8.2	Sterols
24-Methylenelophenol	-8.2	Sterols
Δ7,22-Ergostadienol	-8.2	Sterols
Butyrospermol	-8.2	Sterols
Cycloartenol	-8.2	Sterols
Lupeol	-8.2	Sterols
Uvaol	-8.2	Sterols
24-methyl-24(25)-dehydrolophenol	-8.1	Sterols
Campesterol	-8.1	Sterols
Stigmasterol	-8.1	Sterols
Δ-7-Avenasterol	-8.1	Sterols
28-isocitrostadienol	-8.0	Sterols
24-Ethyl-E-23-dehydrolophenol	-8.0	Sterols
β-sitosterol	-8.0	Sterols
Parkeol	-8.0	Sterols
3-epi-betulin	-8.0	Sterols
Erythrodiol	-8.0	Sterols
24-ethyllophenol	-7.9	Sterols
24-Methylene-cholesterol	-7.9	Sterols
Δ-7-Stigmastenol	-7.9	Sterols
24-methylene-cycloartenol	-7.9	Sterols
Campestanol	-7.8	Sterols
Cyclobranol	-7.8	Sterols
Obtusifoliol	-7.7	Sterols
Δ-5-Avenasterol	-7.7	Sterols
Δ-5,23-Stigmastadienol	-7.7	Sterols
Δ7,24-Ergostadienol	-7.7	Sterols
22,23- Dihydrobrassicasterol	-7.7	Sterols
24-Ethylidenelophenol	-7.6	Sterols
Δ-5,24-Stigmastadienol	-7.6	Sterols
Cyclosadol	-7.6	Sterols
Agrostophyllinol	-7.6	Sterols
Cholesterol	-7.5	Sterols
Stigmastanol	-7.5	Sterols
Cycloeucalenol	-7.2	Sterols

Linalool	-5.6	Sterols
D-(+)-raffinose	-7.8	Sugars
Mannan	-7.7	Sugars
Maltotriose	-7.3	Sugars
Galactinol	-7.1	Sugars
D-(+)-sucrose	-6.8	Sugars
D-(+)-lactose	-6.6	Sugars
α-Cellulose	-6.5	Sugars
L-Fucose	-5.5	Sugars
myo-inositol	-5.4	Sugars
D-Mannitol	-5.3	Sugars
Pectin	-5.3	Sugars
Galacturonan	-5.3	Sugars
D-(+)-mannose	-5.2	Sugars
Sedoheptulose	-5.2	Sugars
D-(+)-galacturonic acid	-5.2	Sugars
D-glucuronic acid	-5.2	Sugars
Xylitol	-5.2	Sugars
D-(+)-glucose	-5.1	Sugars
D-(-)-fructose	-5.1	Sugars
D-(-)-galactose	-5.0	Sugars
L-(-)-arabitol	-5.0	Sugars
D-(+)-chiro-inositol	-5.0	Sugars
D-(-)-arabinose	-4.9	Sugars
L-rhamnose	-4.9	Sugars
D-Fucose	-4.9	Sugars
1,6-anhydro-β-D-glucose	-4.8	Sugars
Adonitol	-4.8	Sugars
D-(+)-xylose	-4.6	Sugars
β-tocopherol	-7.2	Tocopherols
α-tocopherol	-6.8	Tocopherols
δ-tocopherol	-6.7	Tocopherols
γ-tocopherol	-6.6	Tocopherols
Corosolic acid	-9.2	Triterpenic acids
Maslinic acid	-9.1	Triterpenic acids
Urs-2β,3β-dihydroxy-12-en-28-oic acid	-9.0	Triterpenic acids
Oleanolic acid demethyl	-8.9	Triterpenic acids
Pomolic acid	-8.9	Triterpenic acids
Oleanolic acid	-8.6	Triterpenic acids
Betulinic acid	-8.6	Triterpenic acids
3-epi-betulinic acid	-8.6	Triterpenic acids
Ursolic acid	-8.5	Triterpenic acids
cis-3-Hexenyl acetate	-7.0	Volatiles

Ethyl cinnamate	-7.0	Volatiles
Benzyl acetate	-6.3	Volatiles
Acetophenone	-6.2	Volatiles
2-Phenylethanol	-6.0	Volatiles
Ethyl cyclohexylcarboxylate	-5.9	Volatiles
Phenylacetaldehyde	-5.8	Volatiles
trans-4,5-Epoxy-trans-2-decenal	-5.8	Volatiles
6-Methyl-5-hepten-3-ol	-5.7	Volatiles
Benzaldehyde	-5.6	Volatiles
Benzyl alcohol	-5.6	Volatiles
Phenethyl acetate	-5.6	Volatiles
3,4-methyl-3-pentenyl furan	-5.6	Volatiles
trans-β-Damascenone	-5.6	Volatiles
trans,trans-2,4-Decadienal	-5.5	Volatiles
trans,trans-2,4-Nonadienal	-5.4	Volatiles
2,4-Decadienal	-5.4	Volatiles
Vanillin	-5.4	Volatiles
Ethyl octanoate	-5.4	Volatiles
2-Ethyl-5-hexylthiophene	-5.4	Volatiles
trans-2-Decenal	-5.3	Volatiles
Methyl octanoate	-5.3	Volatiles
2-Nonanone	-5.3	Volatiles
3-Octenol	-5.2	Volatiles
Terpineol	-5.2	Volatiles
2-Hexenyl acetate	-5.2	Volatiles
3-Hexenyl acetate	-5.2	Volatiles
3-Methyl-2-pentylfuran	-5.2	Volatiles
Gluconic acid	-5.2	Volatiles
trans,cis-2,4-Decadienal	-5.1	Volatiles
2-Heptanol	-5.1	Volatiles
Heptan-2-ol	-5.1	Volatiles
2-Octanol	-5.1	Volatiles
Octanoic acid	-5.1	Volatiles
Isopentyl acetate	-5.1	Volatiles
Octyl acetate	-5.1	Volatiles
2-Ethylphenyl acetate	-5.1	Volatiles
Methyl heptanoate	-5.1	Volatiles
trans-2-Octenal	-5.0	Volatiles
cis-2-Nonenal	-5.0	Volatiles
trans-2-Nonenal	-5.0	Volatiles
Decanal	-5.0	Volatiles
Decanol	-5.0	Volatiles
2-Methylbutyl acetate	-5.0	Volatiles

2-Methylpropyl butanoate	-5.0	Volatiles
1,8-Cineole	-5.0	Volatiles
6-Methyl-5-hepten-2-one	-5.0	Volatiles
2-Octanone	-5.0	Volatiles
Octan-2-one	-5.0	Volatiles
cis-1,5-Octadien-3-one	-5.0	Volatiles
2,4-Heptadienal	-4.9	Volatiles
Nonanal	-4.9	Volatiles
Heptanoic acid	-4.9	Volatiles
Hexyl acetate	-4.9	Volatiles
l-Octen-3-one	-4.9	Volatiles
Citric acid	-4.9	Volatiles
Octanal	-4.8	Volatiles
Heptanol	-4.8	Volatiles
Octanol	-4.8	Volatiles
Nonanol	-4.8	Volatiles
Propyl butanoate	-4.8	Volatiles
Ethyl 2-methylbutanoate	-4.8	Volatiles
Ethyl 3-methylbutanoate	-4.8	Volatiles
Propyl 2-methylpropanoate	-4.8	Volatiles
Ethyl hexanoate	-4.8	Volatiles
3-Octanone	-4.8	Volatiles
Malic acid	-4.8	Volatiles
trans-2-Heptenal	-4.7	Volatiles
trans-2-Hexenol	-4.7	Volatiles
cis-2-Hexenol	-4.7	Volatiles
Lavendulol	-4.7	Volatiles
2-Propylfuran	-4.7	Volatiles
trans-3-Hexenol	-4.6	Volatiles
cis-3-Hexenol	-4.6	Volatiles
Methyl hexanoate	-4.6	Volatiles
3-Propylfuran	-4.6	Volatiles
Heptan-2-one	-4.6	Volatiles
2-Heptanone	-4.6	Volatiles
3-Isopropenylthiophene	-4.6	Volatiles
trans-2-Hexenal	-4.5	Volatiles
Heptanal	-4.5	Volatiles
cis-2-Heptenal	-4.5	Volatiles
Hexanol	-4.5	Volatiles
Hexanoic acid	-4.5	Volatiles
Butyl acetate	-4.5	Volatiles
Ethyl butanoate	-4.5	Volatiles
Methyl pentanoate	-4.5	Volatiles

2-Ethylfuran	-4.5	Volatiles
2,5-Diethylthiophene	-4.5	Volatiles
2,4 Hexadienal	-4.4	Volatiles
cis-2-Hexenal	-4.4	Volatiles
3-Penten-2-ol	-4.4	Volatiles
4-Hexenol	-4.4	Volatiles
3-Methyl-butanoic acid	-4.4	Volatiles
Ethyl isobutyrate	-4.4	Volatiles
Propyl propanoate	-4.4	Volatiles
Ethyl-2-methypropanoate	-4.4	Volatiles
Methyl 3-methylbutanoate	-4.4	Volatiles
4-Methyl-2-pentanone	-4.4	Volatiles
Succinic acid	-4.4	Volatiles
2-Methyl-2-butenal	-4.3	Volatiles
3-Hexanal	-4.3	Volatiles
cis-3-Hexenal	-4.3	Volatiles
3-Methylbutyric acid	-4.3	Volatiles
Pentanoic acid	-4.3	Volatiles
2-Hexanone	-4.3	Volatiles
Hexanal	-4.2	Volatiles
2-Methyl-1-butanol	-4.2	Volatiles
3-Methyl-1-butanol	-4.2	Volatiles
2-Penten-1-ol	-4.2	Volatiles
Methyl butanoate	-4.2	Volatiles
4-Methoxy-2-methyl-2-butanethiol	-4.2	Volatiles
trans-2-Pentenal	-4.1	Volatiles
cis-2-Pentenal	-4.1	Volatiles
2-Methyl-3-butenol	-4.1	Volatiles
Pentanol	-4.1	Volatiles
3-Pentanol	-4.1	Volatiles
1-Penten-3-ol	-4.1	Volatiles
Ethyl propanoate	-4.1	Volatiles
3-Methyl-2-butanone	-4.1	Volatiles
3-Methylbutanal	-4.0	Volatiles
2-Methylbutanal	-4.0	Volatiles
Pentanal	-4.0	Volatiles
Isobutyric acid	-4.0	Volatiles
l-Penten-3-one	-4.0	Volatiles
1-Penten-3-one	-4.0	Volatiles
3-Methyl-2-butenethiol	-4.0	Volatiles
Butanoic acid	-3.9	Volatiles
3-Pentanone	-3.9	Volatiles
Methyl 2-methylbutanoate	-3.8	Volatiles

Oxalic acid	-3.8	Volatiles
Butan-2-ol	-3.7	Volatiles
Butan-1-ol	-3.6	Volatiles
Ethyl acetate	-3.6	Volatiles
Propanal	-3.5	Volatiles
Butan-2-one	-3.5	Volatiles
2-Butanone	-3.5	Volatiles
Propanoic acid	-3.4	Volatiles
Methyl acetate	-3.2	Volatiles
1-Propanol	-3.1	Volatiles
Diethyl ether	-3.1	Volatiles
Acetic acid	-2.9	Volatiles
Ethanol	-2.6	Volatiles
Acetaldehyde	-2.3	Volatiles
Methanol	-2.0	Volatiles

Table S8. Predicted binding affinities of the olive-derived compounds against the representative protein structure for cluster 3 of the GIRK4^{WT} channel.

**Compound**	**Binding Affinity (kcal/mol)**	**OliveNet^{TM} Class**
Geranylgeraniol	-6.3	Aliphatic and aromatic
Tetracosanol	-5.7	Aliphatic and aromatic
Docosanol	-5.6	Aliphatic and aromatic
Tricosanol	-5.4	Aliphatic and aromatic
Hexacosanol	-5.2	Aliphatic and aromatic
Heptacosanol	-5.2	Aliphatic and aromatic
Phytol	-4.9	Aliphatic and aromatic
Eicosanol	-4.8	Aliphatic and aromatic
Octacosanol	-4.8	Aliphatic and aromatic
Pentacosanol	-4.6	Aliphatic and aromatic
Tyrosine	-7.3	Amino acids
Phenylalanine	-6.6	Amino acids
Arginine	-6.5	Amino acids
Glutamine	-5.9	Amino acids
Leucine	-5.5	Amino acids
Glutamic Acid	-5.4	Amino acids
Asparagine	-5.3	Amino acids
Aspartic Acid	-5.2	Amino acids
Isoleucine	-5.1	Amino acids
Valine	-5.0	Amino acids
Serine	-4.9	Amino acids
Alanine	-4.7	Amino acids
Petroselinic	-6.0	Fatty acids
11-cis-vaccenic	-5.9	Fatty acids
Linoelaidic	-5.9	Fatty acids
Gadoleic	-5.9	Fatty acids
Trans-palmitoleic	-5.8	Fatty acids
Cis-10-Heptadecenoic	-5.8	Fatty acids
Lignoceric	-5.8	Fatty acids
Stearic	-5.7	Fatty acids
Methyl linoleate	-5.6	Fatty acids
Oleic	-5.6	Fatty acids
Elaidic	-5.6	Fatty acids
Palmitic	-5.5	Fatty acids
Arachidic	-5.5	Fatty acids
Eicosenoic	-5.5	Fatty acids
Myristic	-5.4	Fatty acids
Palmitoleic	-5.4	Fatty acids
Linoleic	-5.4	Fatty acids

Lauric	-5.4	Fatty acids
Ethyl palmitate	-5.3	Fatty acids
Methyl palmitate	-5.3	Fatty acids
2,3-diacylglycerol	-5.2	Fatty acids
1,3-diacylglycerol	-5.2	Fatty acids
Ethyl linoleate	-5.2	Fatty acids
Methyl oleate	-5.2	Fatty acids
3-monoacylglycerol	-5.2	Fatty acids
Ethyl oleate	-5.1	Fatty acids
Erucic	-5.0	Fatty acids
1,2-diacylglycerol	-4.9	Fatty acids
Ethyl stearate	-4.9	Fatty acids
1-monoacylglycerol	-4.9	Fatty acids
Methyl stearate	-4.8	Fatty acids
Linolenic	-4.8	Fatty acids
Behenic	-4.8	Fatty acids
Methyl heptadecanoate	-4.7	Fatty acids
Margaric acid	-4.7	Fatty acids
2-monoacylglycerol	-4.7	Fatty acids
Calarene	-6.8	Hydrocarbons
α-copaene	-6.2	Hydrocarbons
δ-cadinene	-6.2	Hydrocarbons
β-cubebene	-6.1	Hydrocarbons
(E)-caryophyllene	-6.1	Hydrocarbons
γ-Muurolene	-6.1	Hydrocarbons
β- Sesquiphellandrene	-6.1	Hydrocarbons
Phytyl C22:0	-6.1	Hydrocarbons
Geranylgeranyl C22:0	-6.1	Hydrocarbons
Longicyclene	-6.0	Hydrocarbons
Drima-7,9(11)-diene	-6.0	Hydrocarbons
α-Selinene	-6.0	Hydrocarbons
β-Curcumene	-6.0	Hydrocarbons
(Z)2,(E)4,(E)6-Allofarnesene	-6.0	Hydrocarbons
Cyclosativene	-5.9	Hydrocarbons
α-trans-bergamotene	-5.9	Hydrocarbons
α-Zingiberene	-5.9	Hydrocarbons
Alloaromadendrene	-5.8	Hydrocarbons
γ-curcumene	-5.8	Hydrocarbons
Eremophyllene	-5.8	Hydrocarbons
(E)2,(Z)4,(E)6-Allofarnesene	-5.8	Hydrocarbons
Eremophilone	-5.7	Hydrocarbons
Squalene	-5.7	Hydrocarbons
β-acoradiene	-5.7	Hydrocarbons

Phytyl C20:0	-5.7	Hydrocarbons
β-elemene	-5.6	Hydrocarbons
Geranylgeranyl oleate C18:1	-5.6	Hydrocarbons
6,10-dimethyl-1-undecene	-5.5	Hydrocarbons
9-tricosene	-5.5	Hydrocarbons
(E)-β-farnesene	-5.5	Hydrocarbons
Geranylgeranyl C24:0	-5.5	Hydrocarbons
8-heptdecene	-5.4	Hydrocarbons
9-docosene	-5.4	Hydrocarbons
(Z)-β-farnesene	-5.4	Hydrocarbons
Ethyl oleate	-5.4	Hydrocarbons
Geranylgeranyl C20:1	-5.4	Hydrocarbons
Tricosane	-5.3	Hydrocarbons
Styrene	-5.3	Hydrocarbons
9-hexacosene	-5.3	Hydrocarbons
Wax ester 42:1 (16:1-26:0)	-5.3	Hydrocarbons
Icosane	-5.2	Hydrocarbons
Docosane	-5.2	Hydrocarbons
Tetracosane	-5.2	Hydrocarbons
9-pentacosene	-5.2	Hydrocarbons
6,10-dimethyl-1-undecane	-5.2	Hydrocarbons
Methyl stearate	-5.2	Hydrocarbons
Phytyl oleate C18:1	-5.2	Hydrocarbons
Phytyl C20:1	-5.2	Hydrocarbons
Wax ester 40:0 (16:0-24:0)	-5.2	Hydrocarbons
Wax ester 40:1 (16:1-24:0)	-5.2	Hydrocarbons
Octadecane	-5.1	Hydrocarbons
Methyl oleate	-5.1	Hydrocarbons
Wax ester 40:0 (14:0-26:0)	-5.1	Hydrocarbons
Wax ester 40:1 (18:1-22:0)	-5.1	Hydrocarbons
Wax ester 38:0 (14:0-24:0)	-5.0	Hydrocarbons
Wax ester 42:0 (16:0-26:0)	-5.0	Hydrocarbons
Wax ester 46:0 (16:0-30:0)	-5.0	Hydrocarbons
Wax ester 46:0 (22:0-24:0)	-5.0	Hydrocarbons
Hexacpsane	-4.9	Hydrocarbons
Octacosane	-4.9	Hydrocarbons
Methyl benzene	-4.9	Hydrocarbons
9-tetracosene	-4.9	Hydrocarbons
Methyl palmitate	-4.9	Hydrocarbons
Phytyl oleate C18:0	-4.9	Hydrocarbons
Wax ester 40:0 (18:0-22:0)	-4.9	Hydrocarbons
Wax ester 46:0 (24:0-22:0)	-4.9	Hydrocarbons
Wax ester 46:1 (18:1-28:0)	-4.9	Hydrocarbons

Heptacosane	-4.8	Hydrocarbons
Nonacosane	-4.8	Hydrocarbons
Hentriacontane	-4.8	Hydrocarbons
Tridecane	-4.8	Hydrocarbons
9-heptacosene	-4.8	Hydrocarbons
Wax ester 38:0 (12:0-26:0)	-4.8	Hydrocarbons
Wax ester 38:0 (16:0-22:0)	-4.8	Hydrocarbons
Geranylgeranyl oleate C18:0	-4.7	Hydrocarbons
Phytyl C24:0	-4.7	Hydrocarbons
Wax ester 38:0 (20:0-18:0)	-4.7	Hydrocarbons
Wax ester 46:0 (14:0-32:0)	-4.7	Hydrocarbons
Wax ester 46:0 (18:0-28:0)	-4.7	Hydrocarbons
Wax ester 46:0 (20:0-26:0)	-4.7	Hydrocarbons
Nonadecane	-4.6	Hydrocarbons
Heneicosane	-4.6	Hydrocarbons
Pentacosane	-4.6	Hydrocarbons
Tricontane	-4.6	Hydrocarbons
Geranylgeranyl C20:0	-4.6	Hydrocarbons
Wax ester 40:0 (20:0-20:0)	-4.6	Hydrocarbons
Wax ester 44:0 (22:0-22:0)	-4.6	Hydrocarbons
Wax ester 44:1 (16:0-28:0)	-4.6	Hydrocarbons
Hexadecane	-4.5	Hydrocarbons
Nonane	-4.5	Hydrocarbons
Wax ester 38:0 (18:0-20:0)	-4.5	Hydrocarbons
Wax ester 42:0 (14:0-28:0)	-4.5	Hydrocarbons
Wax ester 42:0 (18:0-24:0)	-4.5	Hydrocarbons
Heptadecane	-4.4	Hydrocarbons
Tritriacontane	-4.4	Hydrocarbons
Octane	-4.4	Hydrocarbons
Wax ester 42:0 (20:0-22:0)	-4.4	Hydrocarbons
Wax ester 44:0 (16:0-28:0)	-4.4	Hydrocarbons
Wax ester 44:1 (18:1-26:0)	-4.4	Hydrocarbons
Tridecane	-4.3	Hydrocarbons
Pentadecane	-4.3	Hydrocarbons
Heptane	-4.3	Hydrocarbons
Wax ester 44:1 (20:1-24:0)	-4.3	Hydrocarbons
Tetratriacontane	-4.2	Hydrocarbons
Tetradecane	-4.1	Hydrocarbons
2-methylpentane	-4.1	Hydrocarbons
Wax ester 42:0 (24:0-18:0)	-4.1	Hydrocarbons
Wax ester 42:1 (18:1-24:0)	-4.1	Hydrocarbons
Wax ester 44:0 (18:0-26:0)	-4.1	Hydrocarbons
Hexene	-4.0	Hydrocarbons

Dodecane	-3.9	Hydrocarbons
Dotriacotane	-3.9	Hydrocarbons
Hexane	-3.9	Hydrocarbons
3-methylpentane	-3.8	Hydrocarbons
2-Methylbutane	-3.5	Hydrocarbons
Poly-unsaturated di-galactoside glycerol diester	-7.1	Other
3-[1-(hydroxymethyl)-(E)-1-propenyl] glutaric acid	-6.1	Other
Halleridone	-5.4	Other
3-(1-Hydroxymethyl-1-propenyl)pentanedioic acid	-5.4	Other
3-[1-(formyl)-(E)-1-propenyl] glutaric acid	-5.4	Other
1,5-anhydroxylitol	-4.8	Other
Luteolin-7-O-rutinoside	-11.3	Phenolic compounds
Isorhoifolin	-11.1	Phenolic compounds
Apigenin-7-O-rutinoside	-10.8	Phenolic compounds
Nüzhenide oleoside	-10.7	Phenolic compounds
Isoacteoside	-10.4	Phenolic compounds
Hesperidin	-10.3	Phenolic compounds
Scolymoside	-10.3	Phenolic compounds
Oxidized isoverbascoside	-10.3	Phenolic compounds
Rutin	-10.1	Phenolic compounds
Luteolin-7-O-glucoside	-10.1	Phenolic compounds
Quercetin 3-O-rutinoside	-10.1	Phenolic compounds
β-Hydroxy verbascoside	-10.1	Phenolic compounds
Oleuropein dimer	-10.1	Phenolic compounds
Verbascoside	-10.1	Phenolic compounds
Isoverbascoside	-10.1	Phenolic compounds
Suspensaside	-10.1	Phenolic compounds
Chrysoeriol-7-O-glucoside	-10.0	Phenolic compounds
Luteolin-3′,7-O-diglucoside	-9.9	Phenolic compounds
Apigenin-7-O-glucoside	-9.9	Phenolic compounds
Jaspolyanoside	-9.9	Phenolic compounds
Luteolin-4'-O-rutinoside	-9.8	Phenolic compounds
Isojaspolyoside A	-9.8	Phenolic compounds
Oxidized verbascoside	-9.8	Phenolic compounds
Luteolin-7,4-O-diglucoside	-9.7	Phenolic compounds
Nüzhenide 11-Methyl oleoside	-9.7	Phenolic compounds
Demethyloleuropein	-9.7	Phenolic compounds

Jaspolyoside	-9.7	Phenolic compounds
Acetoside	-9.7	Phenolic compounds
Hellicoside	-9.6	Phenolic compounds
Orbanchoside	-9.6	Phenolic compounds
Oleuricine A	-9.5	Phenolic compounds
Ligstroside derivative 4	-9.5	Phenolic compounds
Ligstroside derivative 5	-9.5	Phenolic compounds
β-Hydroxy-acetoside	-9.5	Phenolic compounds
4'-O-β-D-Glucosyl-9-O-(6"-deoxysaccharosyl)olivil	-9.5	Phenolic compounds
Quercetin-7-O-glucoside	-9.5	Phenolic compounds
Caffeoyl-6'-secologanoside	-9.5	Phenolic compounds
Comselogoside	-9.4	Phenolic compounds
Luteolin-6-C-glucoside	-9.3	Phenolic compounds
Nüzhenide	-9.3	Phenolic compounds
Oleuroside-10-carboxylic acid	-9.3	Phenolic compounds
Lucidumoside C	-9.2	Phenolic compounds
Delphinidin-3-O-glucoside	-9.2	Phenolic compounds
Neo-nüzhenide	-9.2	Phenolic compounds
10-Hydroxyoleuropein	-9.2	Phenolic compounds
Oleuropein-3'-O-β-D-glucopyranoside	-9.2	Phenolic compounds
7"-S-Hydroxyoleuropein	-9.2	Phenolic compounds
Ligstroside derivative 3	-9.2	Phenolic compounds
Hydroxytyrosol diglucoside	-9.2	Phenolic compounds
Luteolin-4’-O-glucoside	-9.1	Phenolic compounds
Ligstroside	-9.1	Phenolic compounds
Cyanidin-3-O-rutinoside	-9.0	Phenolic compounds
(+)-1-Acetoxypinoresinol-4'-β-D-glucopyranoside-4"-O-methyl ether	-9.0	Phenolic compounds
(+)-Fraxiresinol-1-β-D-glucopyranoside	-9.0	Phenolic compounds
Oleuropein	-9.0	Phenolic compounds
Oleuroside	-9.0	Phenolic compounds
Ligstroside-3'-O-β-D-glucopyranoside	-9.0	Phenolic compounds
Elenolic acid diglucoside	-9.0	Phenolic compounds
Demethylligstroside	-9.0	Phenolic compounds
Cyanidin-3-O-glucoside	-8.9	Phenolic compounds
Quercitrin	-8.9	Phenolic compounds
Oleuropein diglucoside	-8.9	Phenolic compounds
6'-β-D-Glucopyranosyl oleoside	-8.9	Phenolic compounds
Vicenin-2	-8.8	Phenolic compounds
Luteolin-8-C-glucoside	-8.8	Phenolic compounds

Dihydro-oleuropein	-8.8	Phenolic compounds
Chlorogenic acid	-8.7	Phenolic compounds
(+)-1-Hydroxypinoresinol-4''-O-methyl ether	-8.7	Phenolic compounds
(+)-1-Acetoxypinoresinol-4'-β-D-glucopyranoside	-8.7	Phenolic compounds
(+)-1-Hydroxypinoresinol-4'-β-D-glucopyranoside	-8.7	Phenolic compounds
6'-Rhamnopyranosyl oleoside	-8.7	Phenolic compounds
Rosmarinic acid	-8.6	Phenolic compounds
Oleuricine B	-8.6	Phenolic compounds
Wedelosin	-8.6	Phenolic compounds
Quercetin-3-O-glucoside	-8.5	Phenolic compounds
Pinoresinol	-8.5	Phenolic compounds
Quercetin-3-rhamnoside	-8.4	Phenolic compounds
Quercetin	-8.3	Phenolic compounds
Eriodictyol	-8.3	Phenolic compounds
(+)-1-Acetoxypinoresinol-4''-O-methyl ether	-8.3	Phenolic compounds
Oleuropeindial - Lactone (Cannizzaro-like product of oleuropeindial)	-8.3	Phenolic compounds
Oleuropein-3''-Methyl ether	-8.3	Phenolic compounds
Ligstroside derivative 1	-8.3	Phenolic compounds
Scopolin	-8.3	Phenolic compounds
Diosmetin	-8.2	Phenolic compounds
Luteolin	-8.2	Phenolic compounds
Delphinidin	-8.2	Phenolic compounds
Hesperitin	-8.2	Phenolic compounds
Caffeoylglucose	-8.2	Phenolic compounds
Syringaresinol	-8.2	Phenolic compounds
1-Acetoxypinoresinol	-8.2	Phenolic compounds
Hydroxypinoresinol	-8.2	Phenolic compounds
Demethyloleuropein aglycone	-8.2	Phenolic compounds
6'-O-[(2E)-2,6-Dimethyl-8-hydroxy- 2-octenoyloxy]-secologanoside	-8.2	Phenolic compounds
Taxifolin	-8.1	Phenolic compounds
Chrysoeriol	-8.1	Phenolic compounds
Methoxyluteolin	-8.1	Phenolic compounds
Cyanidin (cation)	-8.1	Phenolic compounds
3-Acetyloxy berchemol	-8.1	Phenolic compounds
(-)-Olivil	-8.1	Phenolic compounds
Secologanic acid	-8.1	Phenolic compounds
10-Hydroxy-10-methyl oleuropein aglycone	-8.1	Phenolic compounds
Esculin	-8.1	Phenolic compounds

Oleuropein aglycone (3,4-DHPEA-EA)	-8.0	Phenolic compounds
Apigenin	-7.9	Phenolic compounds
Caftaric acid	-7.9	Phenolic compounds
Berchemol	-7.9	Phenolic compounds
10-Hydroxy oleuropein aglycone decarboxymethyl	-7.9	Phenolic compounds
Ligstroside derivative 2	-7.9	Phenolic compounds
Oleoside dimethylester	-7.8	Phenolic compounds
10-Hydroxy oleuropein aglycone	-7.8	Phenolic compounds
Hydroxytyrosol rhamnoside	-7.8	Phenolic compounds
Hydroxytyrosol-1′-β-glucoside	-7.8	Phenolic compounds
Monoaldehydic form of Oleuropein aglycon	-7.7	Phenolic compounds
Ligstroside aglycone methyl acetal	-7.7	Phenolic compounds
Loganin	-7.7	Phenolic compounds
Verucosin	-7.7	Phenolic compounds
Salidroside	-7.7	Phenolic compounds
Ferulic acid	-7.6	Phenolic compounds
Elenolic acid glucoside	-7.6	Phenolic compounds
Cornoside	-7.6	Phenolic compounds
Caffeic acid	-7.5	Phenolic compounds
Hydroxycaffeic acid	-7.5	Phenolic compounds
Secologanin	-7.5	Phenolic compounds
Oleuropeindial (enol form)	-7.5	Phenolic compounds
Oleoside-11-Methylester	-7.5	Phenolic compounds
Hydroxytyrosol-3-β-glucoside	-7.5	Phenolic compounds
Hydroxytyrosil-elenolate	-7.4	Phenolic compounds
Monoaldehydic form of Ligstroside aglycon	-7.4	Phenolic compounds
Oleuropeindial (Cannizzaro-like product of oleuropeindial)	-7.4	Phenolic compounds
Loganic Acid	-7.4	Phenolic compounds
Oleoside	-7.4	Phenolic compounds
Secologanoside	-7.4	Phenolic compounds
p-Coumaric acid	-7.3	Phenolic compounds
1-(3’-Methoxy-4’-hydroxy)- phenyl-6,7-dihydroxyisochroman	-7.3	Phenolic compounds
7-Deoxyloganic acid	-7.3	Phenolic compounds
Demethyloleuropein aglycone dialdehyde	-7.3	Phenolic compounds
Ligstroside aglycone	-7.3	Phenolic compounds
Hydroxytyrosol-4-β-glucoside	-7.3	Phenolic compounds
Phloretic acid	-7.1	Phenolic compounds
o-Coumaric acid	-7.1	Phenolic compounds
m-Coumaric acid	-7.1	Phenolic compounds

1-Phenyl-6,7-dihydroxyisochroman	-7.1	Phenolic compounds
3,4-DHPEA-DETA	-7.1	Phenolic compounds
demethyloleuropein aglycone (enol form)	-7.1	Phenolic compounds
Oleuropeindial (keto form)	-7.1	Phenolic compounds
(+)-Cycloolivil	-7.1	Phenolic compounds
Dihydro-p-coumaric acid	-7.0	Phenolic compounds
Cinnamic acid	-7.0	Phenolic compounds
Secologanol	-7.0	Phenolic compounds
Hydroxytyrosol acyclodihydroelenolate	-7.0	Phenolic compounds
2,3-dihydrocaffeic acid	-6.9	Phenolic compounds
Oleacein (Dialdehydic form of decarboxymethyl Oleuropein aglycon)	-6.9	Phenolic compounds
3,4-DHPEA-DEDA (acetal)	-6.9	Phenolic compounds
Hemiacetal of dialdehydic oleuropein aglycone decarboxymethyl	-6.9	Phenolic compounds
Sinapic acid	-6.8	Phenolic compounds
Methyl malate-hydroxytyrosol ester	-6.8	Phenolic compounds
Oleocanthal (Dialdehydic form of decarboxymethyl Ligstroside aglycon)	-6.8	Phenolic compounds
Esculetin	-6.8	Phenolic compounds
Tyrosol acetate	-6.7	Phenolic compounds
3,4-DHPEA-EDA (Oleuropein-aglycone di-aldehyde)	-6.7	Phenolic compounds
3,4-DHPEA-DEDA (Oleuropein aglycone decarboxymethyl dialdehyde form)	-6.7	Phenolic compounds
Decarboxymethyl ligstroside aglycone	-6.7	Phenolic compounds
Scopoletin	-6.7	Phenolic compounds
Hydroxytyrosol acetate	-6.6	Phenolic compounds
Hemiacetal of dialdehydic ligstroside aglycone decarboxymethyl	-6.6	Phenolic compounds
4-Hydroxy-3-methoxy-phenylacetic acid	-6.5	Phenolic compounds
Homovanillic acid	-6.5	Phenolic compounds
Isoeugenol	-6.5	Phenolic compounds
3,4-Dihydroxyphenylacetic acid	-6.4	Phenolic compounds
3,4-Dihydroxyphenylglycol	-6.3	Phenolic compounds
Vanillic acid	-6.3	Phenolic compounds
Gentisic acid	-6.3	Phenolic compounds
2,5-Dihydroxyphenylacetic acid	-6.3	Phenolic compounds
p-HPEA-EDA	-6.3	Phenolic compounds
Homovanillin	-6.3	Phenolic compounds
Hydroxytyrosol	-6.2	Phenolic compounds
Homovanillyl alcohol	-6.2	Phenolic compounds
3,4-Dimethoxybenzoic acid	-6.2	Phenolic compounds
Protocatechuic acid	-6.2	Phenolic compounds

D-(+)-Erythro-1-(4-hydroxy-3-methoxy)-214 - phenyl-1,2,3-propantriol	-6.2	Phenolic compounds
Gallic acid	-6.1	Phenolic compounds
p-Hydroxyphenylacetic acid	-6.1	Phenolic compounds
4-hydroxybenzoic acid	-6.0	Phenolic compounds
Demethyl elenolic acid	-6.0	Phenolic compounds
Tyrosol	-5.9	Phenolic compounds
4-Ethylguaiacol	-5.9	Phenolic compounds
4-Vinylguaiacol	-5.9	Phenolic compounds
Syringic acid	-5.9	Phenolic compounds
2,4 dihydroxybenzoic acid	-5.9	Phenolic compounds
Elenolic acid	-5.9	Phenolic compounds
Elenolic acid methylester	-5.9	Phenolic compounds
2-Methoxy-4-vinylphenol	-5.9	Phenolic compounds
Shikimic acid	-5.8	Phenolic compounds
Homoveratric acid	-5.7	Phenolic compounds
DEDA acetal	-5.7	Phenolic compounds
Deoxyloganic acid lauryl ester	-5.7	Phenolic compounds
4-Hydroxybenzaldehyde	-5.6	Phenolic compounds
4-O-methyl-D-glucuronic acid	-5.6	Phenolic compounds
Syringaldehyde	-5.5	Phenolic compounds
4-Ethylphenol	-5.5	Phenolic compounds
4-Vinylphenol	-5.5	Phenolic compounds
3,4,5-Trimethoxybenzoic acid	-5.4	Phenolic compounds
Quinic acid	-5.4	Phenolic compounds
2,6-Dihydroxybenzoic acid	-5.4	Phenolic compounds
Elenolic acid dialdehyde	-5.4	Phenolic compounds
Catechol	-5.3	Phenolic compounds
o-cresol	-5.3	Phenolic compounds
4-Methylcatechol	-5.3	Phenolic compounds
1-oleyltyrosol	-5.3	Phenolic compounds
2,6-Dimethoxybenzoic acid	-5.2	Phenolic compounds
DEDA (Decarboxymethyl elenolic acid dialdehyde)	-5.2	Phenolic compounds
Guaiacol	-5.2	Phenolic compounds
m-cresol	-5.1	Phenolic compounds
p-cresol	-5.0	Phenolic compounds
Phenol	-4.9	Phenolic compounds
Dialdehydic elenolic acid decarboxymethyl	-4.9	Phenolic compounds
Dialdehydic elenolic ester decarboxymethyl	-4.9	Phenolic compounds
Phosphatidylinositol	-6.9	Phospholipids
Lysophosphatidylethanolamine	-6.2	Phospholipids

Lysophosphatidic acid	-6.0	Phospholipids
Phosphatidylcholine	-5.7	Phospholipids
Phosphatidylglycerol	-5.6	Phospholipids
Phosphatidylethanolamine	-5.5	Phospholipids
Phosphatidic acid	-5.1	Phospholipids
Pheophytin α	-10.3	Pigments
Chlorophyllide a	-10.2	Pigments
Chlorophyllide b	-10.1	Pigments
Pheophorbide a	-9.6	Pigments
Pheophorbide b	-9.3	Pigments
Chlorophyll a	-8.9	Pigments
Pheophytin b	-8.5	Pigments
Neoxanthin	-8.1	Pigments
Pyropheophytin α	-8.0	Pigments
Chlorophyll b	-7.9	Pigments
β-Carotene	21.0	Pigments
Mutatoxanthin	25.5	Pigments
β-cryptoxanthin	26.9	Pigments
Antheraxanthin	27.9	Pigments
Lutein	28.6	Pigments
Luteoxanthin	29.1	Pigments
Violaxanthin	34.4	Pigments
Taraxasterol	-9.3	Sterols
β-amyrone	-9.0	Sterols
Germanicol	-9.0	Sterols
β-amyrin	-8.9	Sterols
3-epi-lupeol	-8.8	Sterols
Taraxerol	-8.8	Sterols
Lupenone	-8.8	Sterols
Erythrodiol	-8.8	Sterols
Tirucallol	-8.7	Sterols
28-nor-α-amyrin	-8.7	Sterols
Cyclosadol	-8.6	Sterols
28-nor-β-amyrin	-8.6	Sterols
Ψ-taraxasterol	-8.5	Sterols
Bacchar-12,21-dien-3β-ol	-8.5	Sterols
Ergosterol	-8.4	Sterols
Δ-5,23-Stigmastadienol	-8.3	Sterols
Δ-5,24-Stigmastadienol	-8.3	Sterols
Cycloartenol	-8.3	Sterols
α-amyrin	-8.3	Sterols
Methyl 2α,3β-diacetoxyolean-12-en-28-oate	-8.3	Sterols

Methyl 3β-acetoxyolean-12-en-28-oate	-8.3	Sterols
Uvaol	-8.3	Sterols
24-methyl-(E)-23-dehydrolophenol	-8.2	Sterols
24-methyl-24(25)-dehydrolophenol	-8.2	Sterols
Stigmasterol	-8.2	Sterols
Brassicasterol	-8.2	Sterols
Δ-5-Avenasterol	-8.2	Sterols
7, 24-tirucalladienol	-8.2	Sterols
28-hydroxytaraxerol	-8.2	Sterols
24-methylene-24-dihydrolanosterol	-8.2	Sterols
24-methyl-31-nor-9(11)-lanostenol	-8.1	Sterols
24-methylene-31-nor-9(11)-lanostenol	-8.1	Sterols
24-Methylene-cholesterol	-8.1	Sterols
Δ7,24-Ergostadienol	-8.1	Sterols
Butyrospermol	-8.1	Sterols
24-methylene-cycloartenol	-8.1	Sterols
4,4-dimethyl-5α-stigmasta-7,24Z(241)-dien-3β-ol	-8.1	Sterols
24-Ethyl-E-23-dehydrolophenol	-8.0	Sterols
Cyclobranol	-8.0	Sterols
Parkeol	-8.0	Sterols
3-epi-betulin	-8.0	Sterols
Agrostophyllinol	-8.0	Sterols
δ-amyrin	-8.0	Sterols
Campesterol	-7.9	Sterols
Δ7,22-Ergostadienol	-7.9	Sterols
Lupeol	-7.9	Sterols
(24Z)-24-ethylidene-dihydrolanosterol	-7.9	Sterols
4,4-dimethyl-5α-stigmast-7-en-3β-ol	-7.9	Sterols
24-ethyllophenol	-7.8	Sterols
Cholesterol	-7.8	Sterols
Stigmastanol	-7.8	Sterols
Δ-7-Stigmastenol	-7.8	Sterols
Dammaradienol	-7.8	Sterols
4α,14α-Dimethylstigmasta-8,24(24)-dien-3β-ol	-7.8	Sterols
Citrostadienol	-7.7	Sterols
28-isocitrostadienol	-7.7	Sterols
24-Methylenelophenol	-7.7	Sterols
Campestanol	-7.7	Sterols
24-methylene-24-dihydroparkenol	-7.7	Sterols
Gramisterol	-7.6	Sterols
24-methylene-24-dihydroparkeol	-7.6	Sterols
β-sitosterol	-7.5	Sterols

Δ-7-Avenasterol	-7.5	Sterols
24-Ethylidenelophenol	-7.4	Sterols
22,23- Dihydrobrassicasterol	-7.3	Sterols
Cycloeucalenol	-7.2	Sterols
Obtusifoliol	-7.1	Sterols
Clerosterol	-6.9	Sterols
Linalool	-5.2	Sterols
D-(+)-raffinose	-8.3	Sugars
Mannan	-7.8	Sugars
Galactinol	-7.5	Sugars
Maltotriose	-7.4	Sugars
D-(+)-lactose	-7.1	Sugars
α-Cellulose	-7.0	Sugars
D-(+)-sucrose	-6.6	Sugars
D-glucuronic acid	-5.8	Sugars
Pectin	-5.8	Sugars
Sedoheptulose	-5.7	Sugars
Galacturonan	-5.7	Sugars
D-(+)-galacturonic acid	-5.6	Sugars
myo-inositol	-5.6	Sugars
D-(+)-glucose	-5.5	Sugars
D-(+)-mannose	-5.5	Sugars
D-(-)-fructose	-5.5	Sugars
L-rhamnose	-5.5	Sugars
D-Mannitol	-5.5	Sugars
L-Fucose	-5.5	Sugars
D-(-)-galactose	-5.4	Sugars
1,6-anhydro-β-D-glucose	-5.4	Sugars
Xylitol	-5.4	Sugars
Adonitol	-5.4	Sugars
D-(+)-chiro-inositol	-5.4	Sugars
D-(-)-arabinose	-5.2	Sugars
L-(-)-arabitol	-5.2	Sugars
D-Fucose	-5.2	Sugars
D-(+)-xylose	-5.1	Sugars
α-tocopherol	-7.1	Tocopherols
δ-tocopherol	-6.7	Tocopherols
β-tocopherol	-6.5	Tocopherols
γ-tocopherol	-6.2	Tocopherols
Maslinic acid	-9.0	Triterpenic acids
Oleanolic acid	-8.9	Triterpenic acids
Pomolic acid	-8.9	Triterpenic acids
3-epi-betulinic acid	-8.9	Triterpenic acids

Betulinic acid	-8.8	Triterpenic acids
Oleanolic acid demethyl	-8.7	Triterpenic acids
Ursolic acid	-8.6	Triterpenic acids
Urs-2β,3β-dihydroxy-12-en-28-oic acid	-8.6	Triterpenic acids
Corosolic acid	-8.3	Triterpenic acids
Benzyl acetate	-6.7	Volatiles
trans-β-Damascenone	-6.3	Volatiles
2-Ethylphenyl acetate	-6.1	Volatiles
Ethyl cinnamate	-6.0	Volatiles
Vanillin	-5.8	Volatiles
Phenethyl acetate	-5.8	Volatiles
3,4-methyl-3-pentenyl furan	-5.8	Volatiles
Acetophenone	-5.8	Volatiles
Gluconic acid	-5.8	Volatiles
Phenylacetaldehyde	-5.7	Volatiles
2-Phenylethanol	-5.7	Volatiles
2,4-Decadienal	-5.6	Volatiles
trans,trans-2,4-Decadienal	-5.6	Volatiles
Lavendulol	-5.6	Volatiles
cis-3-Hexenyl acetate	-5.6	Volatiles
Benzaldehyde	-5.5	Volatiles
Octanoic acid	-5.5	Volatiles
trans,trans-2,4-Nonadienal	-5.4	Volatiles
trans-4,5-Epoxy-trans-2-decenal	-5.4	Volatiles
Benzyl alcohol	-5.4	Volatiles
Heptanoic acid	-5.4	Volatiles
2-Methylbutyl acetate	-5.4	Volatiles
Isopentyl acetate	-5.4	Volatiles
2-Hexenyl acetate	-5.4	Volatiles
Hexyl acetate	-5.4	Volatiles
3-Hexenyl acetate	-5.4	Volatiles
Octyl acetate	-5.4	Volatiles
Citric acid	-5.4	Volatiles
cis-2-Nonenal	-5.3	Volatiles
trans-2-Nonenal	-5.3	Volatiles
trans-2-Decenal	-5.3	Volatiles
trans,cis-2,4-Decadienal	-5.3	Volatiles
Terpineol	-5.3	Volatiles
Ethyl cyclohexylcarboxylate	-5.3	Volatiles
3-Methyl-2-pentylfuran	-5.3	Volatiles
6-Methyl-5-hepten-2-one	-5.3	Volatiles
Malic acid	-5.3	Volatiles
2,4-Heptadienal	-5.2	Volatiles

Decanal	-5.2	Volatiles
Hexanoic acid	-5.2	Volatiles
Succinic acid	-5.2	Volatiles
2-Ethyl-5-hexylthiophene	-5.2	Volatiles
trans-2-Heptenal	-5.1	Volatiles
Butyl acetate	-5.1	Volatiles
Methyl octanoate	-5.1	Volatiles
2-Propylfuran	-5.1	Volatiles
3-Propylfuran	-5.1	Volatiles
cis-1,5-Octadien-3-one	-5.1	Volatiles
cis-2-Heptenal	-5.0	Volatiles
trans-2-Octenal	-5.0	Volatiles
Nonanal	-5.0	Volatiles
6-Methyl-5-hepten-3-ol	-5.0	Volatiles
Decanol	-5.0	Volatiles
2-Methylpropyl butanoate	-5.0	Volatiles
Methyl heptanoate	-5.0	Volatiles
2-Octanone	-5.0	Volatiles
Octan-2-one	-5.0	Volatiles
2,4 Hexadienal	-4.9	Volatiles
2-Octanol	-4.9	Volatiles
Nonanol	-4.9	Volatiles
Pentanoic acid	-4.9	Volatiles
3-Octanone	-4.9	Volatiles
l-Octen-3-one	-4.9	Volatiles
2-Nonanone	-4.9	Volatiles
cis-2-Hexenal	-4.8	Volatiles
Heptanal	-4.8	Volatiles
Octanal	-4.8	Volatiles
3-Octenol	-4.8	Volatiles
Methyl hexanoate	-4.8	Volatiles
Ethyl hexanoate	-4.8	Volatiles
Ethyl octanoate	-4.8	Volatiles
Heptan-2-one	-4.8	Volatiles
2-Heptanone	-4.8	Volatiles
cis-3-Hexenal	-4.7	Volatiles
trans-2-Hexenal	-4.7	Volatiles
2-Heptanol	-4.7	Volatiles
trans-3-Hexenol	-4.7	Volatiles
Heptan-2-ol	-4.7	Volatiles
Heptanol	-4.7	Volatiles
Octanol	-4.7	Volatiles
3-Methylbutyric acid	-4.7	Volatiles

3-Methyl-butanoic acid	-4.7	Volatiles
Ethyl 2-methylbutanoate	-4.7	Volatiles
Ethyl 3-methylbutanoate	-4.7	Volatiles
2-Ethylfuran	-4.7	Volatiles
trans-2-Hexenol	-4.6	Volatiles
4-Hexenol	-4.6	Volatiles
Butanoic acid	-4.6	Volatiles
Ethyl butanoate	-4.6	Volatiles
Propyl butanoate	-4.6	Volatiles
Propyl 2-methylpropanoate	-4.6	Volatiles
Methyl pentanoate	-4.6	Volatiles
1,8-Cineole	-4.6	Volatiles
cis-2-Pentenal	-4.5	Volatiles
Hexanal	-4.5	Volatiles
3-Hexanal	-4.5	Volatiles
cis-3-Hexenol	-4.5	Volatiles
cis-2-Hexenol	-4.5	Volatiles
Propyl propanoate	-4.5	Volatiles
Methyl 2-methylbutanoate	-4.5	Volatiles
Methyl 3-methylbutanoate	-4.5	Volatiles
2-Hexanone	-4.5	Volatiles
Oxalic acid	-4.5	Volatiles
2-Methyl-2-butenal	-4.4	Volatiles
trans-2-Pentenal	-4.4	Volatiles
Hexanol	-4.4	Volatiles
Ethyl acetate	-4.4	Volatiles
Ethyl isobutyrate	-4.4	Volatiles
Ethyl-2-methypropanoate	-4.4	Volatiles
3-Penten-2-ol	-4.3	Volatiles
2-Penten-1-ol	-4.3	Volatiles
Isobutyric acid	-4.3	Volatiles
Ethyl propanoate	-4.3	Volatiles
Methyl butanoate	-4.3	Volatiles
2,5-Diethylthiophene	-4.3	Volatiles
Pentanal	-4.2	Volatiles
3-Methylbutanal	-4.1	Volatiles
3-Methyl-1-butanol	-4.1	Volatiles
Pentanol	-4.1	Volatiles
3-Pentanol	-4.1	Volatiles
1-Penten-3-ol	-4.1	Volatiles
Propanoic acid	-4.1	Volatiles
l-Penten-3-one	-4.1	Volatiles
3-Pentanone	-4.1	Volatiles

1-Penten-3-one	-4.1	Volatiles
2-Methyl-3-butenol	-4.0	Volatiles
4-Methyl-2-pentanone	-4.0	Volatiles
3-Isopropenylthiophene	-4.0	Volatiles
2-Methylbutanal	-3.9	Volatiles
Butan-2-ol	-3.9	Volatiles
2-Methyl-1-butanol	-3.9	Volatiles
Methyl acetate	-3.9	Volatiles
3-Methyl-2-butanone	-3.9	Volatiles
Propanal	-3.8	Volatiles
Butan-2-one	-3.8	Volatiles
2-Butanone	-3.8	Volatiles
3-Methyl-2-butenethiol	-3.8	Volatiles
Butan-1-ol	-3.7	Volatiles
4-Methoxy-2-methyl-2-butanethiol	-3.6	Volatiles
Acetic acid	-3.5	Volatiles
Diethyl ether	-3.4	Volatiles
1-Propanol	-3.3	Volatiles
Acetaldehyde	-2.7	Volatiles
Ethanol	-2.6	Volatiles
Methanol	-1.9	Volatiles

Table S9. Predicted binding affinities of the olive-derived compounds against the representative protein structure for cluster 3 of the GIRK4^{G151R} channel.

**Compound**	**Binding Affinity (kcal/mol)**	**Class**
Geranylgeraniol	-6.2	Aliphatic and aromatic
Phytol	-5.3	Aliphatic and aromatic
Docosanol	-4.8	Aliphatic and aromatic
Tricosanol	-4.8	Aliphatic and aromatic
Pentacosanol	-4.8	Aliphatic and aromatic
Hexacosanol	-4.7	Aliphatic and aromatic
Tetracosanol	-4.6	Aliphatic and aromatic
Eicosanol	-4.6	Aliphatic and aromatic
Heptacosanol	-4.4	Aliphatic and aromatic
Octacosanol	-4.4	Aliphatic and aromatic
Tyrosine	-5.5	Amino acids
Arginine	-5.5	Amino acids
Phenylalanine	-5.3	Amino acids
Glutamine	-4.5	Amino acids
Glutamic Acid	-4.4	Amino acids
Asparagine	-4.4	Amino acids
Isoleucine	-4.4	Amino acids
Leucine	-4.3	Amino acids
Serine	-4.3	Amino acids
Valine	-4.2	Amino acids
Aspartic Acid	-4.0	Amino acids
Alanine	-3.6	Amino acids
Linolenic	-5.4	Fatty acids
Petroselinic	-5.2	Fatty acids
11-cis-vaccenic	-5.2	Fatty acids
Methyl linoleate	-5.2	Fatty acids
Ethyl linoleate	-5.2	Fatty acids
Linoelaidic	-5.1	Fatty acids
Gadoleic	-5.1	Fatty acids
Cis-10-Heptadecenoic	-5.1	Fatty acids
Eicosenoic	-5.1	Fatty acids
Linoleic	-5.1	Fatty acids
Erucic	-5.1	Fatty acids
Methyl oleate	-5.0	Fatty acids
Ethyl oleate	-5.0	Fatty acids
Trans-palmitoleic	-4.9	Fatty acids
Oleic	-4.9	Fatty acids
Elaidic	-4.9	Fatty acids
Palmitic	-4.9	Fatty acids

Palmitoleic	-4.9	Fatty acids
Ethyl palmitate	-4.9	Fatty acids
Ethyl stearate	-4.9	Fatty acids
Methyl stearate	-4.9	Fatty acids
Behenic	-4.9	Fatty acids
Margaric acid	-4.9	Fatty acids
Lignoceric	-4.8	Fatty acids
Arachidic	-4.8	Fatty acids
Methyl heptadecanoate	-4.8	Fatty acids
Stearic	-4.7	Fatty acids
Myristic	-4.7	Fatty acids
Lauric	-4.6	Fatty acids
Methyl palmitate	-4.6	Fatty acids
2,3-diacylglycerol	-4.4	Fatty acids
1,3-diacylglycerol	-4.4	Fatty acids
1,2-diacylglycerol	-4.4	Fatty acids
2-monoacylglycerol	-4.3	Fatty acids
3-monoacylglycerol	-4.0	Fatty acids
1-monoacylglycerol	-3.9	Fatty acids
Calarene	-6.6	Hydrocarbons
Drima-7,9(11)-diene	-6.4	Hydrocarbons
Alloaromadendrene	-6.4	Hydrocarbons
(E)-caryophyllene	-6.3	Hydrocarbons
α-trans-bergamotene	-6.3	Hydrocarbons
Geranylgeranyl C20:1	-6.3	Hydrocarbons
α-copaene	-6.2	Hydrocarbons
Geranylgeranyl C22:0	-6.2	Hydrocarbons
Geranylgeranyl C24:0	-6.2	Hydrocarbons
β-cubebene	-6.1	Hydrocarbons
Longicyclene	-6.1	Hydrocarbons
γ-curcumene	-6.1	Hydrocarbons
Eremophilone	-6.1	Hydrocarbons
Squalene	-6.1	Hydrocarbons
Geranylgeranyl oleate C18:0	-6.1	Hydrocarbons
δ-cadinene	-6.0	Hydrocarbons
γ-Muurolene	-6.0	Hydrocarbons
α-Selinene	-6.0	Hydrocarbons
Eremophyllene	-6.0	Hydrocarbons
Phytyl oleate C18:1	-6.0	Hydrocarbons
Cyclosativene	-5.9	Hydrocarbons
Geranylgeranyl oleate C18:1	-5.9	Hydrocarbons
β-acoradiene	-5.8	Hydrocarbons
Phytyl C24:0	-5.8	Hydrocarbons

β-Curcumene	-5.7	Hydrocarbons
α-Zingiberene	-5.7	Hydrocarbons
Wax ester 42:0 (16:0-26:0)	-5.7	Hydrocarbons
Wax ester 40:1 (18:1-22:0)	-5.6	Hydrocarbons
Phytyl oleate C18:0	-5.6	Hydrocarbons
β- Sesquiphellandrene	-5.5	Hydrocarbons
(Z)2,(E)4,(E)6-Allofarnesene	-5.5	Hydrocarbons
Wax ester 42:0 (20:0-22:0)	-5.5	Hydrocarbons
β-elemene	-5.4	Hydrocarbons
Wax ester 40:0 (18:0-22:0)	-5.4	Hydrocarbons
Wax ester 38:0 (20:0-18:0)	-5.4	Hydrocarbons
(E)2,(Z)4,(E)6-Allofarnesene	-5.3	Hydrocarbons
Phytyl C20:0	-5.3	Hydrocarbons
(E)-β-farnesene	-5.3	Hydrocarbons
Wax ester 38:0 (14:0-24:0)	-5.3	Hydrocarbons
Wax ester 44:0 (22:0-22:0)	-5.3	Hydrocarbons
Wax ester 44:1 (16:0-28:0)	-5.3	Hydrocarbons
Wax ester 38:0 (18:0-20:0)	-5.3	Hydrocarbons
Wax ester 44:0 (18:0-26:0)	-5.3	Hydrocarbons
Wax ester 42:1 (16:1-26:0)	-5.2	Hydrocarbons
Wax ester 46:0 (24:0-22:0)	-5.2	Hydrocarbons
Wax ester 44:1 (20:1-24:0)	-5.2	Hydrocarbons
Phytyl C20:1	-5.1	Hydrocarbons
Methyl oleate	-5.1	Hydrocarbons
Wax ester 40:0 (14:0-26:0)	-5.1	Hydrocarbons
Wax ester 46:0 (22:0-24:0)	-5.1	Hydrocarbons
Wax ester 38:0 (16:0-22:0)	-5.1	Hydrocarbons
Wax ester 42:1 (18:1-24:0)	-5.1	Hydrocarbons
Ethyl oleate	-5.0	Hydrocarbons
Wax ester 46:0 (16:0-30:0)	-5.0	Hydrocarbons
Wax ester 46:0 (14:0-32:0)	-5.0	Hydrocarbons
Wax ester 46:0 (20:0-26:0)	-5.0	Hydrocarbons
Geranylgeranyl C20:0	-5.0	Hydrocarbons
Wax ester 42:0 (24:0-18:0)	-5.0	Hydrocarbons
Phytyl C22:0	-4.9	Hydrocarbons
9-tricosene	-4.9	Hydrocarbons
9-pentacosene	-4.9	Hydrocarbons
Methyl stearate	-4.9	Hydrocarbons
Wax ester 40:0 (16:0-24:0)	-4.9	Hydrocarbons
Wax ester 46:1 (18:1-28:0)	-4.9	Hydrocarbons
Wax ester 40:0 (20:0-20:0)	-4.9	Hydrocarbons
Wax ester 42:0 (18:0-24:0)	-4.9	Hydrocarbons
Dotriacotane	-4.9	Hydrocarbons

8-heptdecene	-4.8	Hydrocarbons
9-hexacosene	-4.8	Hydrocarbons
Methyl palmitate	-4.8	Hydrocarbons
9-heptacosene	-4.8	Hydrocarbons
(Z)-β-farnesene	-4.7	Hydrocarbons
Wax ester 38:0 (12:0-26:0)	-4.7	Hydrocarbons
Pentacosane	-4.7	Hydrocarbons
Wax ester 44:1 (18:1-26:0)	-4.7	Hydrocarbons
9-docosene	-4.6	Hydrocarbons
Tricosane	-4.6	Hydrocarbons
Styrene	-4.6	Hydrocarbons
Icosane	-4.6	Hydrocarbons
Wax ester 40:1 (16:1-24:0)	-4.6	Hydrocarbons
Hexacpsane	-4.6	Hydrocarbons
Nonacosane	-4.6	Hydrocarbons
Heneicosane	-4.6	Hydrocarbons
Wax ester 42:0 (14:0-28:0)	-4.6	Hydrocarbons
Wax ester 44:0 (16:0-28:0)	-4.6	Hydrocarbons
Tetratriacontane	-4.6	Hydrocarbons
Octadecane	-4.5	Hydrocarbons
Heptacosane	-4.5	Hydrocarbons
Wax ester 46:0 (18:0-28:0)	-4.5	Hydrocarbons
Tricontane	-4.5	Hydrocarbons
6,10-dimethyl-1-undecene	-4.4	Hydrocarbons
6,10-dimethyl-1-undecane	-4.4	Hydrocarbons
9-tetracosene	-4.4	Hydrocarbons
Nonadecane	-4.4	Hydrocarbons
Tritriacontane	-4.4	Hydrocarbons
Docosane	-4.3	Hydrocarbons
Tetracosane	-4.3	Hydrocarbons
Methyl benzene	-4.3	Hydrocarbons
Hentriacontane	-4.3	Hydrocarbons
Heptadecane	-4.3	Hydrocarbons
Tetradecane	-4.3	Hydrocarbons
Octacosane	-4.2	Hydrocarbons
Tridecane	-4.2	Hydrocarbons
Hexadecane	-4.2	Hydrocarbons
Tridecane	-4.2	Hydrocarbons
Pentadecane	-4.2	Hydrocarbons
Nonane	-4.0	Hydrocarbons
Dodecane	-3.9	Hydrocarbons
Octane	-3.8	Hydrocarbons
Heptane	-3.7	Hydrocarbons

3-methylpentane	-3.5	Hydrocarbons
2-methylpentane	-3.4	Hydrocarbons
Hexene	-3.4	Hydrocarbons
Hexane	-3.4	Hydrocarbons
2-Methylbutane	-3.4	Hydrocarbons
Poly-unsaturated di-galactoside glycerol diester	-7.2	Other
3-[1-(hydroxymethyl)-(E)-1-propenyl] glutaric acid	-5.8	Other
Halleridone	-5.1	Other
3-(1-Hydroxymethyl-1-propenyl)pentanedioic acid	-5.1	Other
3-[1-(formyl)-(E)-1-propenyl] glutaric acid	-5.0	Other
1,5-anhydroxylitol	-4.6	Other
Luteolin-7-O-rutinoside	-9.8	Phenolic compounds
Nüzhenide oleoside	-9.8	Phenolic compounds
Isorhoifolin	-9.7	Phenolic compounds
Apigenin-7-O-rutinoside	-9.7	Phenolic compounds
Hesperidin	-9.7	Phenolic compounds
Nüzhenide 11-Methyl oleoside	-9.7	Phenolic compounds
Oleuropein dimer	-9.6	Phenolic compounds
Luteolin-4'-O-rutinoside	-9.6	Phenolic compounds
Oxidized isoverbascoside	-9.3	Phenolic compounds
Suspensaside	-9.3	Phenolic compounds
Ligstroside derivative 4	-9.3	Phenolic compounds
Luteolin-3′,7-O-diglucoside	-9.2	Phenolic compounds
Scolymoside	-9.1	Phenolic compounds
β-Hydroxy verbascoside	-9.1	Phenolic compounds
Luteolin-7,4-O-diglucoside	-9.1	Phenolic compounds
β-Hydroxy-acetoside	-9.1	Phenolic compounds
Verbascoside	-9.0	Phenolic compounds
Nüzhenide	-9.0	Phenolic compounds
Ligstroside derivative 3	-9.0	Phenolic compounds
Vicenin-2	-9.0	Phenolic compounds
Acetoside	-8.9	Phenolic compounds
Hellicoside	-8.9	Phenolic compounds
Orbanchoside	-8.9	Phenolic compounds
4'-O-β-D-Glucosyl-9-O-(6''-deoxysaccharosyl)olivil	-8.9	Phenolic compounds
Cyanidin-3-O-rutinoside	-8.9	Phenolic compounds
Rutin	-8.8	Phenolic compounds

Luteolin-7-O-glucoside	-8.8	Phenolic compounds
Isojaspolyoside A	-8.8	Phenolic compounds
Luteolin-4’-O-glucoside	-8.8	Phenolic compounds
Jaspolyoside	-8.7	Phenolic compounds
Oleuricine A	-8.7	Phenolic compounds
Oleuropein	-8.7	Phenolic compounds
Quercetin 3-O-rutinoside	-8.6	Phenolic compounds
Chrysoeriol-7-O-glucoside	-8.6	Phenolic compounds
Jaspolyanoside	-8.6	Phenolic compounds
Ligstroside derivative 5	-8.6	Phenolic compounds
Oleuropein diglucoside	-8.6	Phenolic compounds
6'-Rhamnopyranosyl oleoside	-8.6	Phenolic compounds
Wedelosin	-8.6	Phenolic compounds
Apigenin-7-O-glucoside	-8.5	Phenolic compounds
Quercetin-7-O-glucoside	-8.5	Phenolic compounds
Comselogoside	-8.5	Phenolic compounds
Delphinidin-3-O-glucoside	-8.5	Phenolic compounds
10-Hydroxyoleuropein	-8.5	Phenolic compounds
(+)-Fraxiresinol-1-β-D-glucopyranoside	-8.5	Phenolic compounds
6'-β-D-Glucopyranosyl oleoside	-8.5	Phenolic compounds
Demethyloleuropein	-8.4	Phenolic compounds
Luteolin-6-C-glucoside	-8.4	Phenolic compounds
Neo-nüzhenide	-8.4	Phenolic compounds
Elenolic acid diglucoside	-8.4	Phenolic compounds
Eriodictyol	-8.4	Phenolic compounds
Ligstroside	-8.3	Phenolic compounds
Cyanidin-3-O-glucoside	-8.3	Phenolic compounds
Quercitrin	-8.3	Phenolic compounds
Oleuricine B	-8.3	Phenolic compounds
1-Acetoxypinoresinol	-8.3	Phenolic compounds
Isoacteoside	-8.2	Phenolic compounds
Isoverbascoside	-8.2	Phenolic compounds
Caffeoyl-6'-secologanoside	-8.2	Phenolic compounds
7''-S-Hydroxyoleuropein	-8.2	Phenolic compounds
Luteolin-8-C-glucoside	-8.2	Phenolic compounds
(+)-1-Hydroxypinoresinol-4'-β-D-glucopyranoside	-8.2	Phenolic compounds
Quercetin-3-rhamnoside	-8.2	Phenolic compounds
Luteolin	-8.2	Phenolic compounds
Oxidized verbascoside	-8.1	Phenolic compounds

Hydroxytyrosol diglucoside	-8.1	Phenolic compounds
Ligstroside-3'-O-β-D-glucopyranoside	-8.1	Phenolic compounds
Taxifolin	-8.1	Phenolic compounds
Oleuroside	-8.0	Phenolic compounds
Rosmarinic acid	-8.0	Phenolic compounds
Quercetin-3-O-glucoside	-8.0	Phenolic compounds
(+)-1-Hydroxypinoresinol-4"-O-methyl ether	-7.9	Phenolic compounds
Quercetin	-7.9	Phenolic compounds
Loganic Acid	-7.9	Phenolic compounds
Oleuropein-3'-O-β-D-glucopyranoside	-7.8	Phenolic compounds
Dihydro-oleuropein	-7.8	Phenolic compounds
(+)-1-Acetoxypinoresinol-4'-β-D-glucopyranoside	-7.8	Phenolic compounds
Delphinidin	-7.8	Phenolic compounds
Chrysoeriol	-7.8	Phenolic compounds
Apigenin	-7.8	Phenolic compounds
Verucosin	-7.8	Phenolic compounds
(+)-1-Acetoxypinoresinol-4'-β-D-glucopyranoside-4"-O-methyl ether	-7.7	Phenolic compounds
Chlorogenic acid	-7.7	Phenolic compounds
Pinoresinol	-7.7	Phenolic compounds
Ligstroside derivative 1	-7.7	Phenolic compounds
Scopolin	-7.7	Phenolic compounds
Diosmetin	-7.7	Phenolic compounds
Hesperitin	-7.7	Phenolic compounds
3-Acetyloxy berchemol	-7.7	Phenolic compounds
Lucidumoside C	-7.6	Phenolic compounds
(+)-1-Acetoxypinoresinol-4"-O-methyl ether	-7.6	Phenolic compounds
Oleuropein-3"-Methyl ether	-7.6	Phenolic compounds
Hydroxypinoresinol	-7.6	Phenolic compounds
Demethyloleuropein aglycone	-7.6	Phenolic compounds
Methoxyluteolin	-7.6	Phenolic compounds
Berchemol	-7.6	Phenolic compounds
Demethylligstroside	-7.5	Phenolic compounds
Caffeoylglucose	-7.5	Phenolic compounds
(-)-Olivil	-7.5	Phenolic compounds
Esculin	-7.5	Phenolic compounds
Ligstroside derivative 2	-7.5	Phenolic compounds
Oleuroside-10-carboxylic acid	-7.4	Phenolic compounds
Cyanidin (cation)	-7.4	Phenolic compounds
10-Hydroxy oleuropein aglycone	-7.4	Phenolic compounds

Oleoside-11-Methylester	-7.4	Phenolic compounds
Hydroxytyrosil-elenolate	-7.4	Phenolic compounds
Syringaresinol	-7.3	Phenolic compounds
Oleoside dimethylester	-7.3	Phenolic compounds
Oleoside	-7.3	Phenolic compounds
Ligstroside aglycone	-7.3	Phenolic compounds
Loganin	-7.2	Phenolic compounds
Elenolic acid glucoside	-7.2	Phenolic compounds
Secologanin	-7.2	Phenolic compounds
Oleuropeindial - Lactone (Cannizzaro-like product of oleuropeindial)	-7.1	Phenolic compounds
6'-O-[(2E)-2,6-Dimethyl-8-hydroxy-2-octenoyloxy]-secologanoside	-7.1	Phenolic compounds
Oleuropein aglycone (3,4-DHPEA-EA)	-7.1	Phenolic compounds
10-Hydroxy oleuropein aglycone decarboxymethyl	-7.1	Phenolic compounds
Monoaldehydic form of Oleuropein aglycon	-7.1	Phenolic compounds
Oleuropeindial (enol form)	-7.1	Phenolic compounds
Hydroxytyrosol-3-β-glucoside	-7.1	Phenolic compounds
1-(3’-Methoxy-4’-hydroxy)- phenyl-6,7-dihydroxyisochroman	-7.1	Phenolic compounds
Secologanic acid	-7.0	Phenolic compounds
10-Hydroxy-10-methyl oleuropein aglycone	-7.0	Phenolic compounds
Ligstroside aglycone methyl acetal	-7.0	Phenolic compounds
Salidroside	-7.0	Phenolic compounds
Cornoside	-7.0	Phenolic compounds
Secologanoside	-7.0	Phenolic compounds
Secologanol	-7.0	Phenolic compounds
Caftaric acid	-6.9	Phenolic compounds
Hydroxytyrosol-1′-β-glucoside	-6.9	Phenolic compounds
7-Deoxyloganic acid	-6.9	Phenolic compounds
3,4-DHPEA-DETA	-6.9	Phenolic compounds
Hydroxytyrosol rhamnoside	-6.8	Phenolic compounds
Hydroxytyrosol-4-β-glucoside	-6.8	Phenolic compounds
(+)-Cycloolivil	-6.8	Phenolic compounds
Demethyloleuropein aglycone dialdehyde	-6.7	Phenolic compounds
1-Phenyl-6,7-dihydroxyisochroman	-6.7	Phenolic compounds
demethyloleuropein aglycone (enol form)	-6.7	Phenolic compounds
Monoaldehydic form of Ligstroside aglycon	-6.6	Phenolic compounds

Oleuropeindial (keto form)	-6.6	Phenolic compounds
Hydroxytyrosol acyclodihydroelenolate	-6.6	Phenolic compounds
3,4-DHPEA-DEDA (acetal)	-6.6	Phenolic compounds
Hemiacetal of dialdehydic oleuropein aglycone decarboxymethyl	-6.6	Phenolic compounds
Sinapic acid	-6.4	Phenolic compounds
3,4-DHPEA-EDA (Oleuropein-aglycone di-aldehyde)	-6.4	Phenolic compounds
3,4-DHPEA-DEDA (Oleuropein aglycone decarboxymethyl dialdehyde form)	-6.4	Phenolic compounds
Hemiacetal of dialdehydic ligstroside aglycone decarboxymethyl	-6.4	Phenolic compounds
Deoxyloganic acid lauryl ester	-6.4	Phenolic compounds
Oleuropeindial (Cannizzaro-like product of oleuropeindial)	-6.3	Phenolic compounds
Oleacein (Dialdehydic form of decarboxymethyl Oleuropein aglycon)	-6.2	Phenolic compounds
Methyl malate-hydroxytyrosol ester	-6.1	Phenolic compounds
Oleocanthal (Dialdehydic form of decarboxymethyl Ligstroside aglycon)	-6.1	Phenolic compounds
Decarboxymethyl ligstroside aglycone	-6.1	Phenolic compounds
p-HPEA-EDA	-6.1	Phenolic compounds
Caffeic acid	-6.0	Phenolic compounds
Hydroxycaffeic acid	-6.0	Phenolic compounds
D-(+)-Erythro-1-(4-hydroxy-3-methoxy)- 214 - phenyl-1,2,3-propantriol	-6.0	Phenolic compounds
Demethyl elenolic acid	-6.0	Phenolic compounds
Esculetin	-5.9	Phenolic compounds
Ferulic acid	-5.8	Phenolic compounds
m-Coumaric acid	-5.8	Phenolic compounds
Scopoletin	-5.8	Phenolic compounds
Hydroxytyrosol acetate	-5.8	Phenolic compounds
Elenolic acid	-5.8	Phenolic compounds
2,3-dihydrocaffeic acid	-5.7	Phenolic compounds
o-Coumaric acid	-5.6	Phenolic compounds
Elenolic acid methylester	-5.6	Phenolic compounds
Quinic acid	-5.6	Phenolic compounds
Cinnamic acid	-5.5	Phenolic compounds
Tyrosol acetate	-5.5	Phenolic compounds

4-Hydroxy-3-methoxy-phenylacetic acid	-5.5	Phenolic compounds
3,4-Dihydroxyphenylglycol	-5.5	Phenolic compounds
Shikimic acid	-5.5	Phenolic compounds
Isoeugenol	-5.4	Phenolic compounds
Gallic acid	-5.4	Phenolic compounds
Syringic acid	-5.4	Phenolic compounds
4-O-methyl-D-glucuronic acid	-5.4	Phenolic compounds
3,4,5-Trimethoxybenzoic acid	-5.4	Phenolic compounds
1-oleyltyrosol	-5.4	Phenolic compounds
p-Coumaric acid	-5.3	Phenolic compounds
3,4-Dihydroxyphenylacetic acid	-5.3	Phenolic compounds
2,6-Dihydroxybenzoic acid	-5.3	Phenolic compounds
Homovanillic acid	-5.2	Phenolic compounds
Vanillic acid	-5.2	Phenolic compounds
Hydroxytyrosol	-5.2	Phenolic compounds
Homoveratric acid	-5.2	Phenolic compounds
Elenolic acid dialdehyde	-5.2	Phenolic compounds
Phloretic acid	-5.1	Phenolic compounds
Dihydro-p-coumaric acid	-5.1	Phenolic compounds
Gentisic acid	-5.1	Phenolic compounds
Homovanillyl alcohol	-5.1	Phenolic compounds
Protocatechuic acid	-5.1	Phenolic compounds
Syringaldehyde	-5.1	Phenolic compounds
2,5-Dihydroxyphenylacetic acid	-5.0	Phenolic compounds
Homovanillin	-5.0	Phenolic compounds
3,4-Dimethoxybenzoic acid	-5.0	Phenolic compounds
2,4 dihydroxybenzoic acid	-5.0	Phenolic compounds
DEDA acetal	-5.0	Phenolic compounds
p-Hydroxyphenylacetic acid	-4.9	Phenolic compounds
4-Ethylguaiacol	-4.9	Phenolic compounds
4-Vinylguaiacol	-4.9	Phenolic compounds
2-Methoxy-4-vinylphenol	-4.9	Phenolic compounds
2,6-Dimethoxybenzoic acid	-4.9	Phenolic compounds
Dialdehydic elenolic ester decarboxymethyl	-4.9	Phenolic compounds
DEDA (Decarboxymethyl elenolic acid dialdehyde)	-4.8	Phenolic compounds
Guaiacol	-4.8	Phenolic compounds
4-hydroxybenzoic acid	-4.7	Phenolic compounds
Catechol	-4.7	Phenolic compounds
4-Methylcatechol	-4.7	Phenolic compounds
m-cresol	-4.7	Phenolic compounds

Dialdehydic elenolic acid decarboxymethyl	-4.7	Phenolic compounds
Phenol	-4.6	Phenolic compounds
Tyrosol	-4.5	Phenolic compounds
4-Ethylphenol	-4.5	Phenolic compounds
4-Vinylphenol	-4.5	Phenolic compounds
o-cresol	-4.5	Phenolic compounds
4-Hydroxybenzaldehyde	-4.3	Phenolic compounds
p-cresol	-4.3	Phenolic compounds
Phosphatidylinositol	-6.4	Phospholipids
Lysophosphatidylethanolamine	-6.1	Phospholipids
Phosphatidylglycerol	-5.4	Phospholipids
Lysophosphatidic acid	-5.3	Phospholipids
Phosphatidylcholine	-5.3	Phospholipids
Phosphatidylethanolamine	-5.1	Phospholipids
Phosphatidic acid	-4.9	Phospholipids
Chlorophyllide a	-9.5	Pigments
Chlorophyllide b	-9.3	Pigments
Pheophorbide a	-9.2	Pigments
Pheophorbide b	-9.0	Pigments
Pheophytin α	-8.7	Pigments
Chlorophyll b	-8.5	Pigments
Pyropheophytin α	-8.1	Pigments
Chlorophyll a	-8.0	Pigments
Neoxanthin	-8.0	Pigments
Pheophytin b	-7.5	Pigments
β-Carotene	15.2	Pigments
Luteoxanthin	16.4	Pigments
Mutatoxanthin	17.7	Pigments
β-cryptoxanthin	23.8	Pigments
Violaxanthin	26.9	Pigments
Antheraxanthin	30.2	Pigments
Lutein	33.1	Pigments
β-amyrin	-9.5	Sterols
Germanicol	-9.3	Sterols
28-nor-α-amyrin	-9.3	Sterols
β-amyrone	-9.2	Sterols
α-amyrin	-9.2	Sterols
Taraxerol	-9.1	Sterols
Erythrodiol	-9.1	Sterols
Taraxasterol	-9.0	Sterols
Ψ-taraxasterol	-9.0	Sterols
Uvaol	-9.0	Sterols

3-epi-lupeol	-8.8	Sterols
Lupenone	-8.8	Sterols
28-nor-β-amyrin	-8.8	Sterols
28-hydroxytaraxerol	-8.8	Sterols
Bacchar-12,21-dien-3β-ol	-8.6	Sterols
24-methylene-24-dihydrolanosterol	-8.6	Sterols
Methyl 3β-acetoxyolean-12-en-28-oate	-8.5	Sterols
4,4-dimethyl-5α-stigmasta-7,24Z(241)-dien-3β-ol	-8.4	Sterols
Parkeol	-8.4	Sterols
Agrostophyllinol	-8.4	Sterols
δ-amyrin	-8.4	Sterols
4α,14α-Dimethylstigmasta-8,24(24)-dien-3β-ol	-8.4	Sterols
Obtusifoliol	-8.4	Sterols
Tirucallol	-8.3	Sterols
7, 24-tirucalladienol	-8.3	Sterols
Ergosterol	-8.2	Sterols
Brassicasterol	-8.2	Sterols
Lupeol	-8.2	Sterols
Stigmasterol	-8.1	Sterols
24-Ethyl-E-23-dehydrolophenol	-8.1	Sterols
24-methylene-24-dihydroparkeol	-8.1	Sterols
Δ-5,24-Stigmastadienol	-8.0	Sterols
Methyl 2α,3β-diacetoxyolean-12-en-28-oate	-8.0	Sterols
24-methyl-(E)-23-dehydrolophenol	-8.0	Sterols
24-methyl-24(25)-dehydrolophenol	-8.0	Sterols
24-methyl-31-nor-9(11)-lanostenol	-8.0	Sterols
24-Methylene-cholesterol	-8.0	Sterols
3-epi-betulin	-8.0	Sterols
Δ7,22-Ergostadienol	-8.0	Sterols
(24Z)-24-ethylidene-dihydrolanosterol	-8.0	Sterols
4,4-dimethyl-5α-stigmast-7-en-3β-ol	-8.0	Sterols
Citrostadienol	-8.0	Sterols
28-isocitrostadienol	-8.0	Sterols
24-methylene-24-dihydroparkenol	-8.0	Sterols
Δ-5,23-Stigmastadienol	-7.9	Sterols
Δ-5-Avenasterol	-7.9	Sterols
24-methylene-31-nor-9(11)-lanostenol	-7.9	Sterols
Δ7,24-Ergostadienol	-7.9	Sterols
24-ethyllophenol	-7.9	Sterols

Dammaradienol	-7.9	Sterols
Campestanol	-7.9	Sterols
22,23- Dihydrobrassicasterol	-7.9	Sterols
Clerosterol	-7.9	Sterols
Cyclosadol	-7.8	Sterols
Butyrospermol	-7.8	Sterols
24-methylene-cycloartenol	-7.8	Sterols
Campesterol	-7.8	Sterols
Stigmastanol	-7.8	Sterols
24-Methylenelophenol	-7.8	Sterols
Gramisterol	-7.8	Sterols
β-sitosterol	-7.8	Sterols
Δ-7-Avenasterol	-7.8	Sterols
Cycloartenol	-7.7	Sterols
Cyclobranol	-7.7	Sterols
Δ-7-Stigmastenol	-7.6	Sterols
Cholesterol	-7.5	Sterols
24-Ethylidenelophenol	-7.3	Sterols
Cycloeucalenol	-7.2	Sterols
Linalool	-4.8	Sterols
Mannan	-8.4	Sugars
D-(+)-raffinose	-7.6	Sugars
Maltotriose	-7.6	Sugars
Galactinol	-7.4	Sugars
D-(+)-lactose	-6.8	Sugars
α-Cellulose	-6.7	Sugars
D-(+)-sucrose	-6.4	Sugars
Pectin	-5.7	Sugars
Sedoheptulose	-5.6	Sugars
Galacturonan	-5.4	Sugars
D-(+)-galacturonic acid	-5.4	Sugars
D-glucuronic acid	-5.3	Sugars
myo-inositol	-5.2	Sugars
D-(+)-mannose	-5.2	Sugars
D-(-)-fructose	-5.2	Sugars
D-(-)-galactose	-5.2	Sugars
D-(+)-glucose	-5.1	Sugars
L-rhamnose	-5.1	Sugars
D-Mannitol	-5.0	Sugars
D-(+)-chiro-inositol	-5.0	Sugars
1,6-anhydro-β-D-glucose	-4.9	Sugars
D-(-)-arabinose	-4.9	Sugars
D-Fucose	-4.9	Sugars

L-Fucose	-4.8	Sugars
L-(-)-arabitol	-4.7	Sugars
Xylitol	-4.6	Sugars
Adonitol	-4.6	Sugars
D-(+)-xylose	-4.6	Sugars
β-tocopherol	-7.3	Tocopherols
γ-tocopherol	-7.1	Tocopherols
α-tocopherol	-6.7	Tocopherols
δ-tocopherol	-6.5	Tocopherols
Pomolic acid	-9.2	Triterpenic acids
Ursolic acid	-9.0	Triterpenic acids
Oleanolic acid	-8.8	Triterpenic acids
Urs-2β,3β-dihydroxy-12-en-28-oic acid	-8.8	Triterpenic acids
3-epi-betulinic acid	-8.7	Triterpenic acids
Betulinic acid	-8.7	Triterpenic acids
Oleanolic acid demethyl	-8.7	Triterpenic acids
Corosolic acid	-8.7	Triterpenic acids
Maslinic acid	-8.5	Triterpenic acids
trans-β-Damascenone	-5.8	Volatiles
Ethyl cinnamate	-5.7	Volatiles
Benzyl acetate	-5.3	Volatiles
Phenethyl acetate	-5.1	Volatiles
Gluconic acid	-5.1	Volatiles
cis-3-Hexenyl acetate	-5.1	Volatiles
Citric acid	-5.0	Volatiles
Terpineol	-5.0	Volatiles
2-Ethyl-5-hexylthiophene	-5.0	Volatiles
2-Ethylphenyl acetate	-4.9	Volatiles
3,4-methyl-3-pentenyl furan	-4.9	Volatiles
Ethyl cyclohexylcarboxylate	-4.9	Volatiles
1,8-Cineole	-4.9	Volatiles
Vanillin	-4.8	Volatiles
Lavendulol	-4.8	Volatiles
trans,trans-2,4-Decadienal	-4.7	Volatiles
Octan-2-one	-4.7	Volatiles
Acetophenone	-4.6	Volatiles
Phenylacetaldehyde	-4.6	Volatiles
2-Phenylethanol	-4.6	Volatiles
trans,trans-2,4-Nonadienal	-4.6	Volatiles
trans-4,5-Epoxy-trans-2-decenal	-4.6	Volatiles
2-Hexenyl acetate	-4.6	Volatiles
Octyl acetate	-4.6	Volatiles

cis-2-Nonenal	-4.6	Volatiles
trans,cis-2,4-Decadienal	-4.6	Volatiles
6-Methyl-5-hepten-2-one	-4.6	Volatiles
Decanal	-4.6	Volatiles
2-Octanol	-4.6	Volatiles
2,4-Decadienal	-4.5	Volatiles
Benzyl alcohol	-4.5	Volatiles
cis-1,5-Octadien-3-one	-4.5	Volatiles
Decanol	-4.5	Volatiles
Benzaldehyde	-4.4	Volatiles
Octanoic acid	-4.4	Volatiles
3-Hexenyl acetate	-4.4	Volatiles
3-Methyl-2-pentylfuran	-4.4	Volatiles
3-Propylfuran	-4.4	Volatiles
Nonanal	-4.4	Volatiles
6-Methyl-5-hepten-3-ol	-4.4	Volatiles
2-Octanone	-4.4	Volatiles
Nonanol	-4.4	Volatiles
2-Nonanone	-4.4	Volatiles
Isopentyl acetate	-4.3	Volatiles
Malic acid	-4.3	Volatiles
2-Methylpropyl butanoate	-4.3	Volatiles
3-Octanone	-4.3	Volatiles
3-Octenol	-4.3	Volatiles
Ethyl octanoate	-4.3	Volatiles
Heptan-2-one	-4.3	Volatiles
Octanol	-4.3	Volatiles
Heptanoic acid	-4.2	Volatiles
trans-2-Decenal	-4.2	Volatiles
2,4-Heptadienal	-4.2	Volatiles
Hexanoic acid	-4.2	Volatiles
trans-2-Octenal	-4.2	Volatiles
Methyl heptanoate	-4.2	Volatiles
l-Octen-3-one	-4.2	Volatiles
2-Heptanone	-4.2	Volatiles
Heptanol	-4.2	Volatiles
2,5-Diethylthiophene	-4.2	Volatiles
trans-2-Nonenal	-4.1	Volatiles
Succinic acid	-4.1	Volatiles
Methyl octanoate	-4.1	Volatiles
2-Propylfuran	-4.1	Volatiles
cis-2-Heptenal	-4.1	Volatiles
Heptanal	-4.1	Volatiles

Ethyl hexanoate	-4.1	Volatiles
2-Heptanol	-4.1	Volatiles
trans-3-Hexenol	-4.1	Volatiles
Heptan-2-ol	-4.1	Volatiles
2-Ethylfuran	-4.1	Volatiles
trans-2-Hexenol	-4.1	Volatiles
Methyl pentanoate	-4.1	Volatiles
cis-2-Hexenol	-4.1	Volatiles
Hexyl acetate	-4.0	Volatiles
trans-2-Heptenal	-4.0	Volatiles
2,4 Hexadienal	-4.0	Volatiles
cis-2-Hexenal	-4.0	Volatiles
Octanal	-4.0	Volatiles
Methyl hexanoate	-4.0	Volatiles
Ethyl 2-methylbutanoate	-4.0	Volatiles
Ethyl 3-methylbutanoate	-4.0	Volatiles
Propyl 2-methylpropanoate	-4.0	Volatiles
Propyl propanoate	-4.0	Volatiles
Ethyl propanoate	-4.0	Volatiles
2-Methylbutyl acetate	-3.9	Volatiles
cis-3-Hexenal	-3.9	Volatiles
trans-2-Hexenal	-3.9	Volatiles
4-Hexenol	-3.9	Volatiles
Ethyl butanoate	-3.9	Volatiles
Propyl butanoate	-3.9	Volatiles
3-Hexanal	-3.9	Volatiles
cis-3-Hexenol	-3.9	Volatiles
2-Hexanone	-3.9	Volatiles
Hexanol	-3.9	Volatiles
Pentanoic acid	-3.8	Volatiles
cis-2-Pentenal	-3.8	Volatiles
Hexanal	-3.8	Volatiles
Oxalic acid	-3.8	Volatiles
3-Isopropenylthiophene	-3.8	Volatiles
Butyl acetate	-3.7	Volatiles
Methyl 2-methylbutanoate	-3.7	Volatiles
Methyl 3-methylbutanoate	-3.7	Volatiles
Ethyl isobutyrate	-3.7	Volatiles
Ethyl-2-methypropanoate	-3.7	Volatiles
Pentanol	-3.7	Volatiles
Propanoic acid	-3.7	Volatiles
3-Methylbutyric acid	-3.6	Volatiles
3-Methyl-butanoic acid	-3.6	Volatiles

2-Methyl-2-butenal	-3.6	Volatiles
3-Penten-2-ol	-3.6	Volatiles
2-Penten-1-ol	-3.6	Volatiles
Methyl butanoate	-3.6	Volatiles
3-Methylbutanal	-3.6	Volatiles
3-Methyl-1-butanol	-3.6	Volatiles
3-Pentanol	-3.6	Volatiles
1-Penten-3-ol	-3.6	Volatiles
l-Penten-3-one	-3.6	Volatiles
3-Pentanone	-3.6	Volatiles
1-Penten-3-one	-3.6	Volatiles
2-Methyl-1-butanol	-3.6	Volatiles
Butanoic acid	-3.5	Volatiles
trans-2-Pentenal	-3.5	Volatiles
Isobutyric acid	-3.5	Volatiles
Pentanal	-3.5	Volatiles
2-Methyl-3-butenol	-3.5	Volatiles
4-Methyl-2-pentanone	-3.5	Volatiles
Butan-2-ol	-3.5	Volatiles
Butan-1-ol	-3.5	Volatiles
Ethyl acetate	-3.4	Volatiles
2-Methylbutanal	-3.4	Volatiles
3-Methyl-2-butanone	-3.4	Volatiles
Propanal	-3.4	Volatiles
Butan-2-one	-3.4	Volatiles
2-Butanone	-3.4	Volatiles
3-Methyl-2-butenethiol	-3.3	Volatiles
Methyl acetate	-3.2	Volatiles
4-Methoxy-2-methyl-2-butanethiol	-3.2	Volatiles
Acetic acid	-3.2	Volatiles
1-Propanol	-3.1	Volatiles
Diethyl ether	-2.9	Volatiles
Acetaldehyde	-2.5	Volatiles
Ethanol	-2.5	Volatiles
Methanol	-2.1	Volatiles

Table S10. Predicted non-covalent interactions of the top 30 compounds with the strongest binding affinities for the representative protein structure of cluster 1 (GIRK4^{WT} channel).

Cluster 1	Binding Affinity (kcal/mol)	Hydrogen Bonds
β-Hydroxy verbascoside	-10.7	Chain B: T149, Q171<br>Chain C: E147
Acetoside	-10.4	Chain A: E147<br>Chain B: T149, Q171
Verbascoside	-10.2	Chain B: T146, Q171<br>Chain C: E147
Isoverbascoside	-10.1	Chain B: E147<br>Chain C: E147, Q171, A172
Isorhoifolin	-10.0	Chain C: E147
Oxidized verbascoside	-10.0	Chain A: E147<br>Chain B: Q171<br>Chain C: E147
Luteolin-4'-O-rutinoside	-9.8	Chain A: T148, S176
Luteolin-7-O-rutinoside	-9.8	Chain C: Y97, E147, A172<br>Chain D: E147
β-Hydroxy-acetoside	-9.8	Chain C: T146<br>Chain D: E147
4'-O-β-D-Glucosyl-9-O-(6"-deoxysaccharosyl)olivil	-9.8	Chain A: E147<br>Chain B: N179<br>Chain D: T146
Hesperidin	-9.7	Chain B: A172, S176<br>Chain C: N179
Apigenin-7-O-rutinoside	-9.7	Chain B: S176<br>Chain C: A172<br>Chain D: S176
Suspensaside	-9.7	Chain A: E147<br>Chain C: E147
Rutin	-9.6	-
Cyanidin-3-O-rutinoside	-9.6	Chain B: S176<br>Chain D: E147, A172, N179
Scolymoside	-9.6	Chain B: E147<br>Chain C: S176, N179
Orbanchoside	-9.6	Chain D: S176
Quercetin 3-O-rutinoside	-9.5	Chain B: Q171
Nüzhenide oleoside	-9.5	Chain D: T149

Oxidized isoverbascoside	-9.5	Chain A: E147<br>Chain B: Q171
Hellicoside	-9.5	Chain B: E147, T149<br>Chain C: Q171, A172<br>Chain D: E147, S176
Chlorophyllide a	-9.5	-
Ligstroside derivative 5	-9.4	Chain C: E147, A172, N179
Isoacteoside	-9.3	Chain B: E147<br>Chain C: E147, T146
Quercetin-3-rhamnoside	-9.3	Chain B: E147, N179
Oleuricine A	-9.2	-
28-nor-β-amyrin	-9.2	Chain D: E147
Luteolin-3′,7-O-diglucoside	-9.1	Chain B: Y97, A172, N179<br>Chain D: E147
Quercitrin	-9.1	Chain B: N179
Neo-nüzhenide	-9.1	-

Table S11. Predicted non-covalent interactions of the top 30 compounds with the strongest binding affinities for the representative protein structure of cluster 1 (GIRK4^{G151R} channel).

Cluster 1	Binding Affinity (kcal/mol)	Hydrogen Bonds	Salt Bridges
Apigenin-7-O-rutinoside	-10.0	Chain A: T148<br>Chain B: N179, T148	
Isorhoifolin	-9.8	Chain A: N179<br>Chain B: R151<br>Chain D: T149	
Oxidized isoverbascoside	-9.8	Chain B: R151	
Pheophorbide a	-9.8	Chain B: R151	Chain D: R151
Scolymoside	-9.7	Chain A: E147<br>Chain B: Y97, G175<br>Chain C: E147	
Isoverbascoside	-9.7	Chain A: N179<br>Chain C: R151	
Hesperidin	-9.6	Chain A: E147	
Luteolin-7-O-rutinoside	-9.6	Chain B: R151<br>Chain C: N179<br>Chain D: T148, R151, N179	
Luteolin-3′,7-O-diglucoside	-9.6	Chain A: T148<br>Chain C: T149, A172, N179	
Quercetin 3-O-rutinoside	-9.6	Chain A: T148<br>Chain B: E147, R151	
Nüzhenide 11-Methyl oleoside	-9.6	Chain A: T148<br>Chain B: S176<br>Chain C: T149, N179	
4'-O-β-D-Glucosyl-9-O-(6"-deoxysaccharosyl)olivil	-9.6	Chain C: N179<br>Chain D: T149	
Germanicol	-9.6	-	
Lupenone	-9.6	-	
Nüzhenide oleoside	-9.5	Chain B: E147, N179<br>Chain C: N179<br>Chain D: E147	
Pheophorbide b	-9.5	Chain B: R151	Chain D: R151
Neo-nüzhenide	-9.4	Chain B: Y97<br>Chain C: T146, E147, R151<br>Chain D: T149	

Isojaspolyoside A	-9.4	Chain C: R151<br>Chain D: T146, T148, T149
Acetoside	-9.4	Chain A: E147
Corosolic acid	-9.4	Chain B: E147
Urs-2β,3β-dihydroxy-12-en-28-oic acid	-9.4	-
Isoacteoside	-9.3	Chain C: R151, S176
Ψ-taraxasterol	-9.3	-
Chlorophyllide a	-9.3	-
Luteolin-7,4-O-diglucoside	-9.2	Chain B: E147<br>Chain C: T148<br>Chain D: N179
Luteolin-4'-O-rutinoside	-9.2	Chain C: T148, A172
Nüzhenide	-9.2	Chain A: T148<br>Chain C: E147, N179
Oleuricine B	-9.2	Chain B: T146, E147, R151, N179
Verbascoside	-9.2	Chain A: E147
β-amyrin	-9.2	-

Table S12. Predicted non-covalent interactions of the top 30 compounds with the strongest binding affinities for the representative protein structure of cluster 2 (GIRK4^{WT} channel).

Cluster 2	Binding Affinity (kcal/mol)	Hydrogen Bonds
Luteolin-4'-O-rutinoside	-10.6	Chain A: E147, A172<br>Chain B: E147<br>Chain C: E147
Luteolin-7-O-rutinoside	-10.5	Chain A: Q171<br>Chain B: E147
Scolymoside	-10.4	Chain C: T149<br>Chain D: Q171, N179
Oleuropein dimer	-10.4	Chain A: N179<br>Chain C: E147
Hesperidin	-10.3	Chain D: E147, N179
Luteolin-3′,7-O-diglucoside	-10.3	Chain A: T148<br>Chain C: T149
Apigenin-7-O-rutinoside	-10.2	Chain A: N179<br>Chain C: A172, G175<br>Chain D: E147, N179
Verbascoside	-10.2	Chain A: E147<br>Chain B: A172<br>Chain D: E147, N179
Cyanidin-3-O-rutinoside	-10.1	Chain C: E147<br>Chain D: N179
Isorhoifolin	-10.1	Chain B: T146, E147<br>Chain D: N179
Isoverbascoside	-9.9	Chain A: E147<br>Chain B: E147, G175
Oxidized isoverbascoside	-9.9	Chain B: E147<br>Chain D: N179
Quercetin 3-O-rutinoside	-9.7	Chain B: E147, S176
Oxidized verbascoside	-9.7	Chain B: A172, G175<br>Chain C: N179
Isoacteoside	-9.7	Chain A: E147, Q171
Luteolin-7,4-O-diglucoside	-9.6	Chain A: E147<br>Chain B: E147
β-Hydroxy verbascoside	-9.6	Chain A: E147<br>Chain C: E147
Acetoside	-9.6	-
Suspensaside	-9.6	Chain A: G175, N179<br>Chain B: E147
Rutin	-9.5	Chain A: Q171, A172<br>Chain B: S176

Luteolin-7-O-glucoside	-9.5	Chain A: T146, E147
Nüzhenide 11-Methyl oleoside	-9.5	Chain A: E147<br>Chain B: T146, E147
Orbanchoside	-9.5	Chain A: T146, N179<br>Chain B: T149
β-amyrone	-9.5	-
Quercitrin	-9.4	Chain A: E147, N179
Ligstroside derivative 4	-9.4	Chain C: A172
β-Hydroxy-acetoside	-9.4	Chain A: A172<br>Chain C: T148, T149
Chrysoeriol-7-O-glucoside	-9.3	Chain A: E147
Luteolin-8-C-glucoside	-9.3	Chain C: T149<br>Chain D: E147, N179
Jaspolyoside	-9.3	Chain B: E147<br>Chain C: E147<br>Chain D: N179

Table S13. Predicted non-covalent interactions of the top 30 compounds with the strongest binding affinities for the representative protein structure of cluster 2 (GIRK4^{G151R} channel).

Cluster 2	Binding Affinity	Hydrogen Bonds	Salt Bridges
Isorhoifolin	-9.6	Chain A: T146	
		Chain B: Y97	
		Chain C: E147, R151	
Rutin	-9.5	Chain C: N179	
Taraxasterol	-9.5	-	
Hellicoside	-9.4	Chain C: E147, T149	
		Chain D: F142	
4'-O-β-D-Glucosyl-9-O-(6"-deoxysaccharosyl)olivil	-9.4	Chain B: A172	
		Chain C: E147, R151, N179	
Dammaradienol	-9.4	-	
Cyanidin-3-O-rutinoside	-9.3	Chain A: A172	
		Chain B: T148, N179	
Oxidized isoverbascoside	-9.3	-	
Luteolin-7-O-rutinoside	-9.2	Chain C: E147, R151	
Apigenin-7-O-rutinoside	-9.2	Chain D: N179	
Demethylligstroside	-9.2	Chain C: S176, N179	Chain D: R151
Ligstroside derivative 4	-9.2	Chain A: E147	
		Chain B: N179	
		Chain D: N179	
Taraxerol	-9.2	-	
28-nor-β-amyrin	-9.2	-	
Corosolic acid	-9.2		Chain D: R151
Luteolin-3′,7-O-diglucoside	-9.1	Chain A: T146, S176	
		Chain B: E147	
		Chain D: T148	
Nüzhenide 11-Methyl oleoside	-9.1	Chain A: E147	
		Chain C: E147, N179	
Demethyloleuropein	-9.1	Chain A: T148	
		Chain D: R151	
Pheophorbide a	-9.1	Chain B: R151	Chain D: R151
		Chain C: R151	
		Chain D: R151	
Pheophorbide b	-9.1	Chain A: N179	Chain D: R151
		Chain B: R151	
		Chain C: R151	
		Chain D: R151	
Maslinic acid	-9.1		Chain D: R151

Hesperidin	-9	Chain A: E147	
Nüzhenide oleoside	-9	Chain A: E147	
		Chain C: N179	
Oleuropein-3'-O-β-D-glucopyranoside	-9	Chain C: N179	
		Chain D: R151	
β-amyrone	-9	-	
28-hydroxytaraxerol	-9	-	
Chlorophyllide a	-9	Chain C: R151	Chain C: R151
			Chain D: R151
Urs-2β,3β-dihydroxy-12-en-28-oic acid	-9		Chain D: R151
Scolymoside	-8.9	Chain A: A172	
		Chain B: Y97, E147	
Luteolin-4'-O-rutinoside	-8.9	Chain C: A172	

Table S14. Predicted non-covalent interactions of the top 30 compounds with the strongest binding affinities for the representative protein structure of cluster 3 (GIRK4^{WT} channel).

Cluster 3	Binding Affinity (kcal/mol)	Hydrogen Bonds
Luteolin-7-O-rutinoside	-11.3	-
Isorhoifolin	-11.1	Chain C: G175
		Chain D: N179
Apigenin-7-O-rutinoside	-10.8	Chain C: Y97, G175
Nüzhenide oleoside	-10.7	Chain A: A172
		Chain C: T148, G175
Isoacteoside	-10.4	Chain C: T148
		Chain D: N179
Hesperidin	-10.3	Chain A: N179
		Chain C: S176
Scolymoside	-10.3	Chain C: T146
Oxidized isoverbascoside	-10.3	Chain A: E147
		Chain D: S176
Pheophytin α	-10.3	-
Chlorophyllide a	-10.2	-
Rutin	-10.1	Chain C: T149
Luteolin-7-O-glucoside	-10.1	Chain C: Q171
Quercetin 3-O-rutinoside	-10.1	Chain B: E147
β-Hydroxy verbascoside	-10.1	Chain C: A172
		Chain D: S176
Oleuropein dimer	-10.1	Chain B: N179
		Chain C: E147
		Chain D: N179
Verbascoside	-10.1	Chain B: S176
		Chain C: Y97
Isoverbascoside	-10.1	Chain C: E147
Suspensaside	-10.1	-
Chlorophyllide b	-10.1	-
Chrysoeriol-7-O-glucoside	-10	Chain C: E147, T148
Luteolin-3′,7-O-diglucoside	-9.9	Chain C: E147
Apigenin-7-O-glucoside	-9.9	-
Jaspolyanoside	-9.9	Chain A: N179
		Chain B: N179
		Chain C: E147
Luteolin-4'-O-rutinoside	-9.8	Chain B: E147
		Chain D: E147
Isojaspolyoside A	-9.8	Chain D: T146, E147
Oxidized verbascoside	-9.8	Chain A: T149
		Chain C: G175

Luteolin-7,4-O-diglucoside	-9.7	Chain A: E147
		Chain D: E147
Nüzhenide 11-Methyl oleoside	-9.7	Chain C: E147
		Chain D: E147
Demethyloleuropein	-9.7	Chain B: N179
		Chain C: E147
Jaspolyoside	-9.7	Chain A: E147

Table S15. Predicted non-covalent interactions of the top 30 compounds with the strongest binding affinities for the representative protein structure of cluster 3 (GIRK4^{G151R} channel).

Cluster 3	Binding Affinity (kcal/mol)	Hydrogen Bonds	Salt Bridges	Π–Π Cation
Luteolin-7-O-rutinoside	-9.8	Chain C: N179		Chain C: R151
Nüzhenide oleoside	-9.8	Chain B: S176, N179		
		Chain C: E147, S176, N179		
		Chain D: N179		
Isorhoifolin	-9.7	Chain A: T146		
		Chain B: Y97, E147		
Apigenin-7-O-rutinoside	-9.7	Chain B: E147, N179		
Hesperidin	-9.7	Chain C: E147		
Nüzhenide 11-Methyl oleoside	-9.7	Chain B: E147		
		Chain C: E147		
Oleuropein dimer	-9.6	Chain B: N179		
		Chain C: A172		
Luteolin-4'-O-rutinoside	-9.6	Chain C: E147, A172, S176, N179		
		Chain D: S176		
Chlorophyllide a	-9.5	Chain D: R151	Chain D: R151	
β-amyrin	-9.5	-		
Oxidized isoverbascoside	-9.3	Chain B: Y97		
		Chain D: T149		
Suspensaside	-9.3	Chain A: E147		
		Chain D: T148, N179		
Chlorophyllide b	-9.3	-		
Ligstroside derivative 4	-9.3	Chain B: Y97, A172		
		Chain C: N179		
		Chain D: N179		
Germanicol	-9.3	-		
28-nor-α-amyrin	-9.3	Chain D: T146		
Luteolin-3′,7-O-diglucoside	-9.2	Chain B: E147, A172, S176		
Pheophorbide a	-9.2		Chain D: R151	
β-amyrone	-9.2	Chain D: R151		
Pomolic acid	-9.2			
α-amyrin	-9.2	-	Chain D: R151	
Scolymoside	-9.1	-		
β-Hydroxy verbascoside	-9.1	Chain B: A172, S176		
		Chain C: T149		

		Chain D: R151, N179
Luteolin-7,4-O-diglucoside	-9.1	Chain B: Y97, W101, E147
		Chain D: N179
β-Hydroxy-acetoside	-9.1	Chain A: N179
		Chain D: R151
Taraxerol	-9.1	-
Erythrodiol	-9.1	Chain D: T148
Vicenin-2	-9	Chain C: T148
		Chain D: R151
Nüzhenide	-9	Chain B: Y97
Ligstroside derivative 3	-9	Chain A: E147, R151
		Chain B: S176
		Chain D: T149

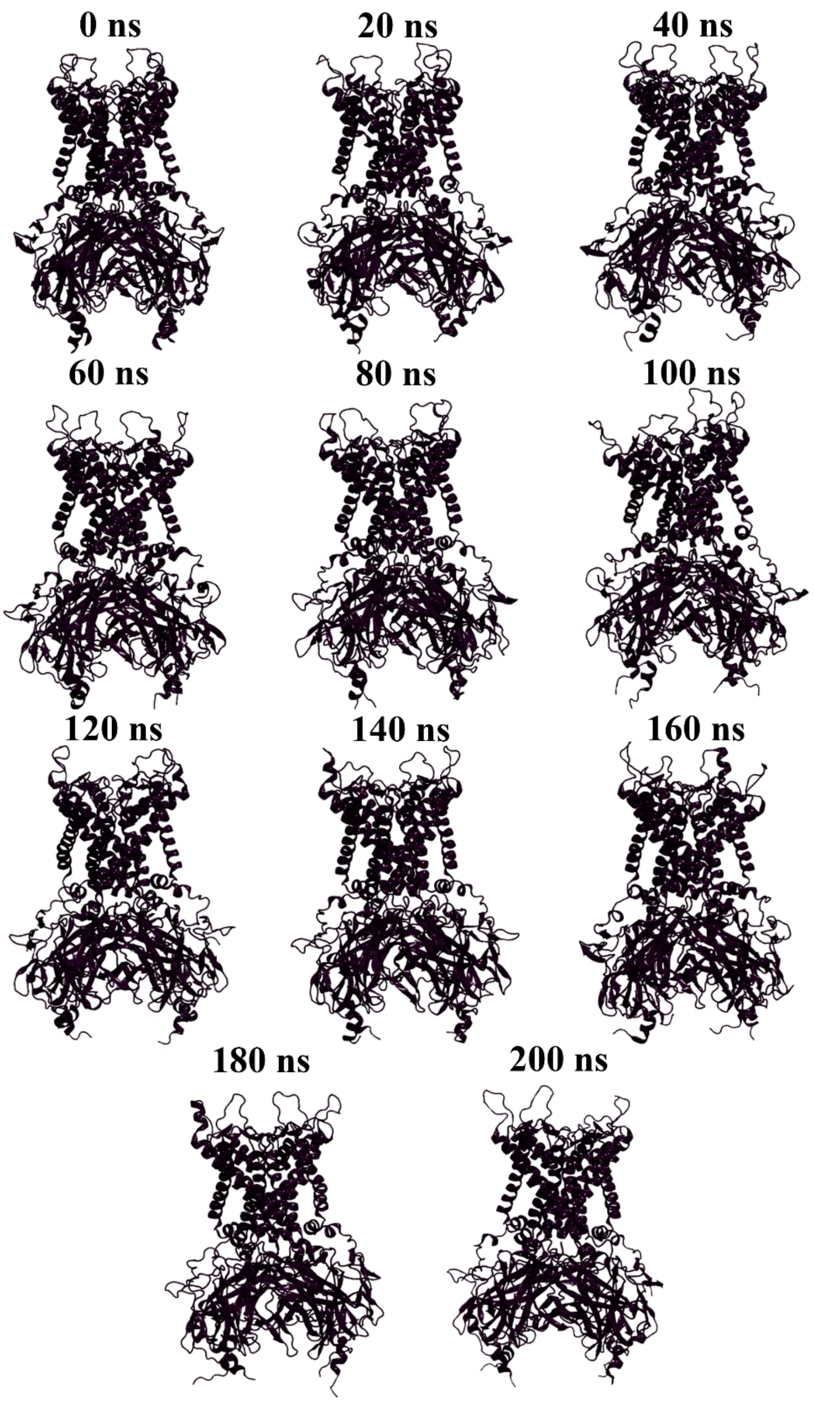

Figure S1. Representative structures of the GIRK4^{WT} channel extracted from molecular dynamics (MD) simulations at 20 ns intervals.

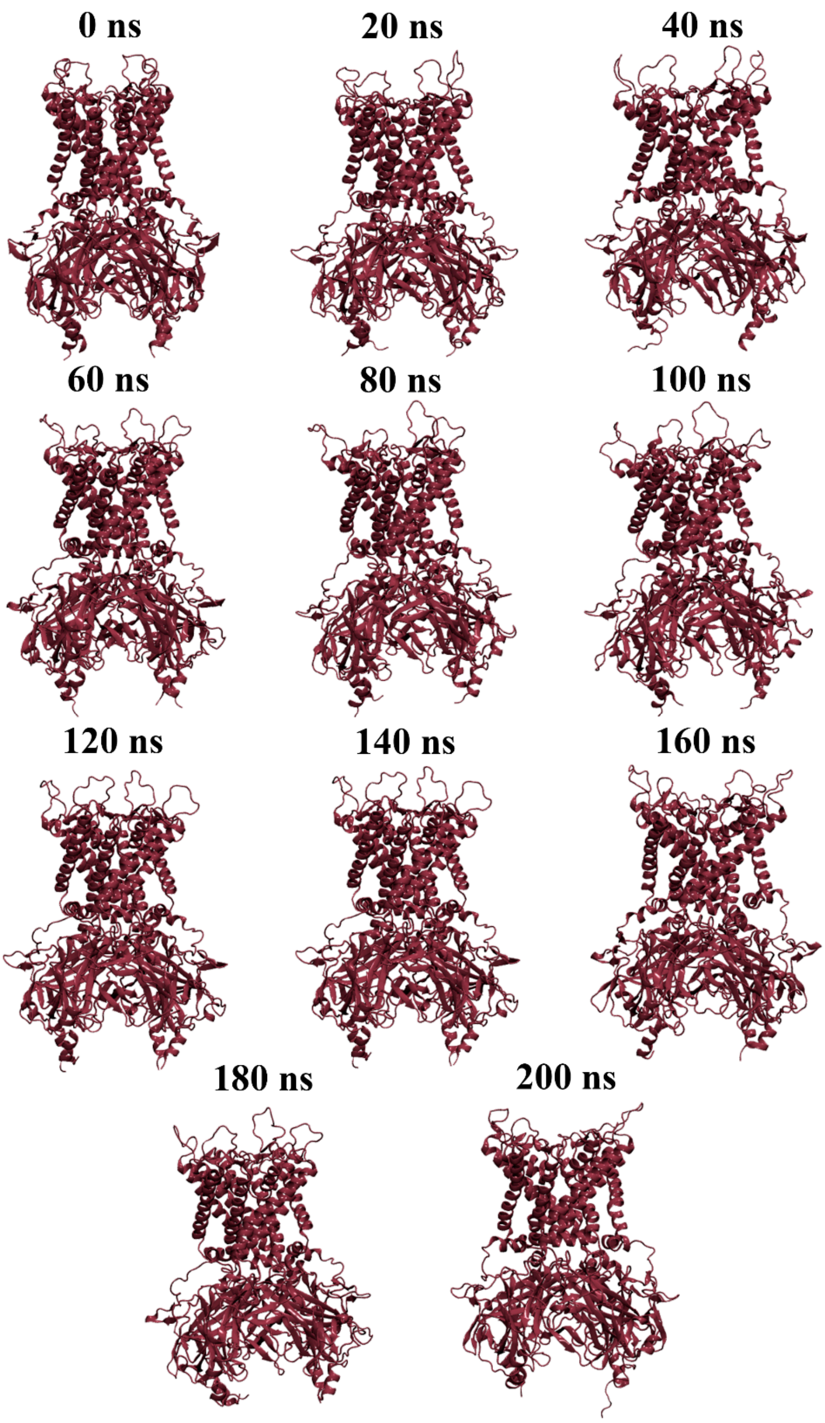

Figure S2. Representative structures of the GIRK4^{G151R} channel extracted from molecular dynamics (MD) simulations at 20 ns intervals.

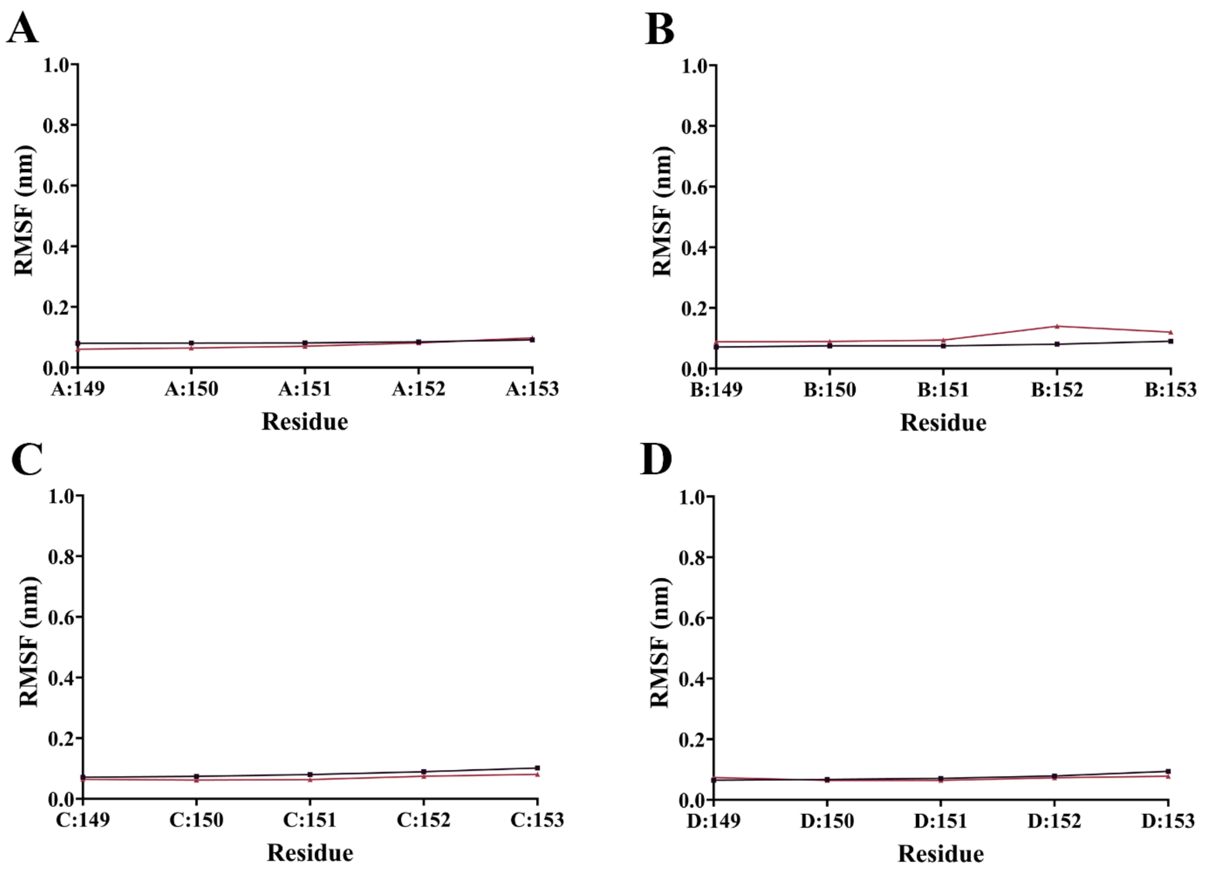

Figure S3. Root mean square fluctuation (RMSF) of protein backbone of WT and G151R GIRK4 following system equilibration. (A-D) The RMSF of the selectivity motif (residues 149-153) for each chain of the homotetrameric complex are shown.

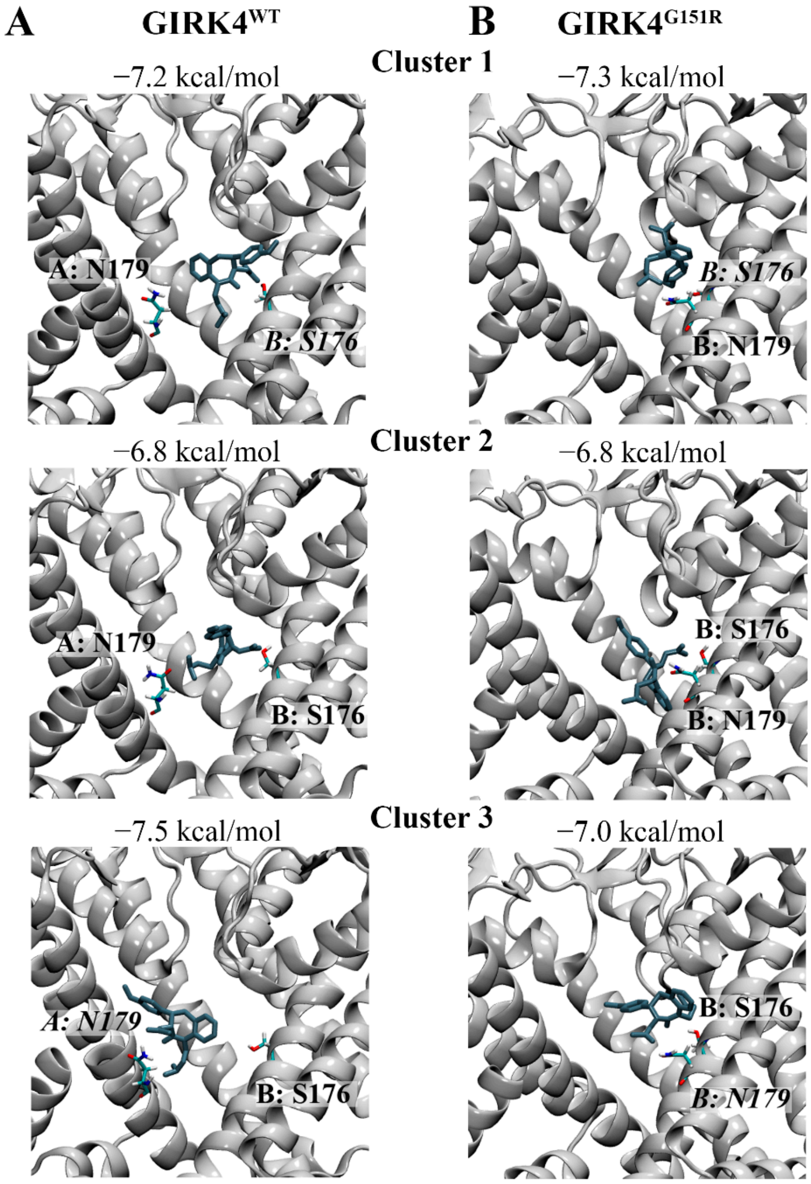

Figure S4. Binding characteristics of diltiazem against the central cavity of GIRK4 channels. The predicted non-covalent interactions of diltiazem with the central cavity of the (A) GIRK4^{WT} and (B) GIRK4^{G151R} channels are provided. Diltiazem is coloured blue. Key residues associated with each chain of the homotetrameric structures are labelled. Residues that were predicted to form hydrogen bonds with diltiazem are italicised.

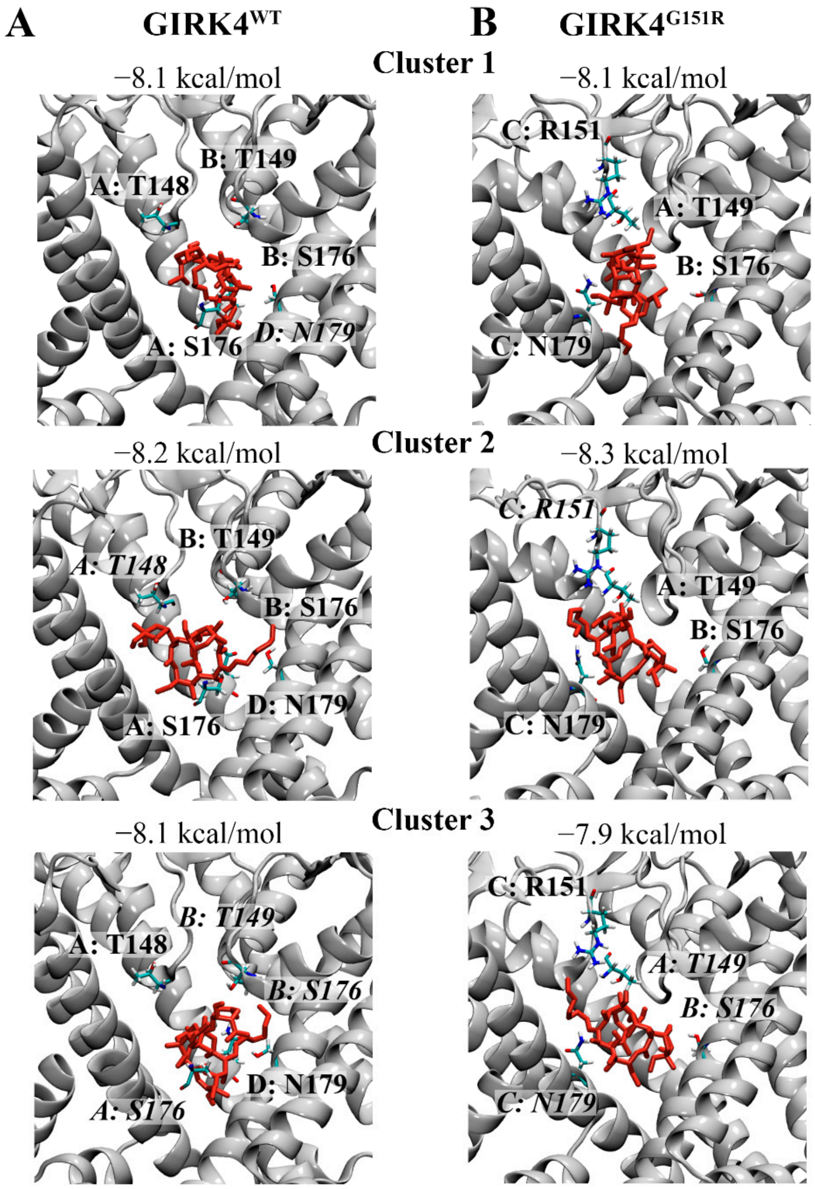

Figure S5. Binding characteristics of roxithromycin against the central cavity of GIRK4 channels. The predicted non-covalent interactions of roxithromycin with the central cavity of the (A) GIRK4^{WT} and (B) GIRK4^{G151R} channels are provided. Roxithromycin is coloured dark red. Key residues associated with each chain of the homotetrameric structures are labelled. Residues that were predicted to form hydrogen bonds with roxithromycin are italicised.
